# Molecular evolution of PCSK family: Analysis of natural selection rate and gene loss

**DOI:** 10.1371/journal.pone.0259085

**Published:** 2021-10-28

**Authors:** Najmeh Parvaz, Zahra Jalali

**Affiliations:** 1 Department of Clinical Biochemistry, School of Medicine, Rafsanjan University of Medical Sciences, Rafsanjan, Iran; 2 Non-Communicable Diseases Research Center, Rafsanjan University of Medical Sciences, Rafsanjan, Iran; Università degli Studi di Milano, ITALY

## Abstract

Proprotein convertases subtilisin kexins are serine endoproteases, playing critical roles in the biological functions, including lipid, glucose, and bile acid metabolism, as well as cell proliferation, migration, and metastasis. Experimental studies have demonstrated the physiological functions of PCSKs and their association with diseases; however, studies on the evolutionary history and diversification of these proteins are missing. In the present research, a bioinformatics study was conducted on the molecular evolution of several PCSKs family members and gene loss events across placental mammalian. In order to detect evolutionary constraints and positive selection, the CodeML program of the PAML package was used. The results showed the positive selection to occur in *PCSK1*, *PCSK3*, *PCSK5*, and *PCSK7*. A decelerated rate of evolution was observed in *PCSK7*, *PCSK3*, and *MBTPS1* in *Carnivores* compared to the rest of phylogeny, and an accelerated evolution of *PCSK1*, *PCSK7*, and *MBTPS1 in Muridae* family of rodents was found. Additionally, our results indicated *pcsk9* gene loss in 12 species comprising *Carnivores* and bats (*Chiroptera*). Future studies are required to evaluate the functional relevance and selective evolutionary advantages associated with these modifications in PCSK proteins during evolution.

## Introduction

Proportion convertases subtilizing kexins (PCSKs) are Ca^+2^ dependent endoproteases belonging to the subtilizing family [1]. These proteases play key roles in a series of biological functions, including lipid, glucose [2], and bile acid metabolism [3], as well as cell proliferation, migration, and metastasis, by converting inactive proteins into their mature forms [4, 5]. Nine members of the PCSK family are divided into two groups, named typical and atypical according to their cleavage site. PC1/3 (PCSK1), PC2 (PCSK2), furin (PCSK3), PC4 (PCSK4), PC5/6 (PCSK5), PACE4 (PCSK6), and PC7 (PCSK7) belong to the typical group, while MBTPS1 [PCSK8] and PCSK9 are members of the atypical group [6]. Furin, PC7, PC5B, and MBTPS1 belong to the class I membrane proteins family, while other PCs are soluble secretory proteins [2, 7–9]. *PCSK1* and *PCSK2* expressions are limited to endocrine and neural tissues. In contrast, furin, PCSK5, PCSK6, and PCSK7 are enzymes widely expressed and target a large number of substrates (e.g., plasma proteins, bacterial toxins, growth factors, and receptors) [2, 10].

**Fig 1:**
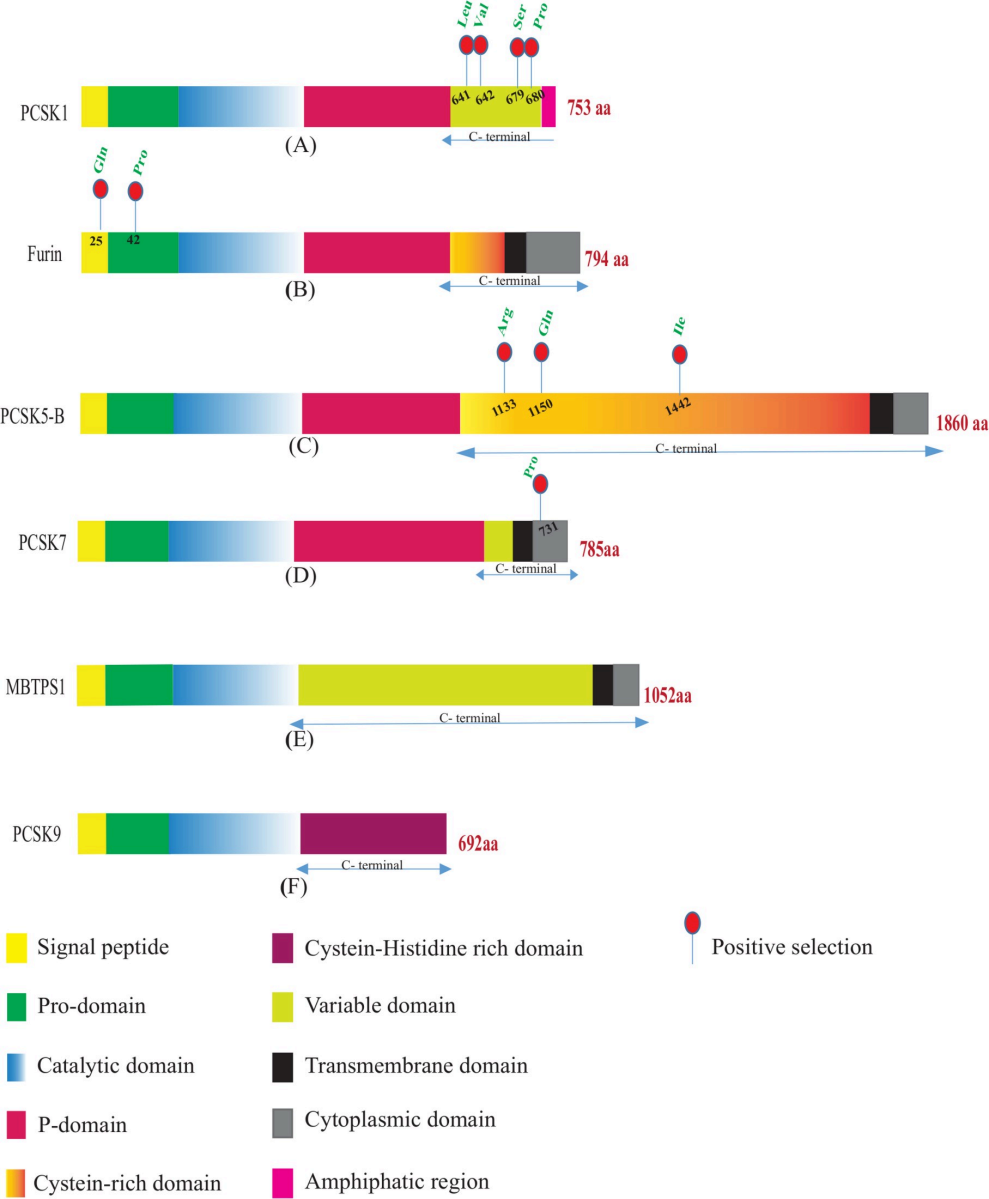
PCSKS structure. Illustration of PCSKS domains and the amino acids which codon has been under positive selection during placental mammalian evolution. Domains are in different colors as indicated.

PCSK proteins consist of several domains, including pro-domain, catalytic domain, p-domain, and C-terminal domain. The pro- and catalytic domains are common among PCSKs, while the C-terminal is unique for each PCSK protein, consisting of several variable subdomains (Fig 1) [10, 11]. The pro-domain acts as a chaperone in protein folding [11, 12]. The p-domain plays a role in the regulation of calcium dependence of PCs and their enzymatic activity [13, 14]. The C-terminal domain is important for subcellular localization and intracellular trafficking [2, 10]. In PCSK7 and MBTPS1, it is divided into three subdomains of variable, transmembrane, and cytoplasmic (Fig 1D and 1E) [2, 8, 15]. In furin, PCSK5, and PCSK6, the C-terminal region consists of a cysteine-rich domain (CRD). The CRD in furin and PCSK5B, is followed by a transmembrane and a cytoplasmic domain (Fig 1B and 1C) [11]. The PCSK9 structure is different from other family members; lacking the p-domain, its catalytic region is followed only by a cysteine-histidine-rich domain (CHRD) [16–19] (Fig 1F).

Members of the PCSK family play diverse critical functions in the body. Here, we selected PCSKs shown to contribute to lipid and glucose metabolism regulation. *PCSK1* is expressed in neuroendocrine and endocrine tissues [10, 20]. One of the most important substrates of this PC is proinsulin [21]. In patients with *PCSK1* deficiency, the level of proinsulin and the risk of obesity increase [22]. Furin is important in lipid metabolism by cleaving lipoprotein lipase (LPL) and endothelial lipase [23]. PCSK5 plays an essential role in lipid metabolism by cleaving two enzymes, including lipoprotein lipase and endothelial lipase [24, 25]. PCSK7 is the transmembrane protease acting in adipocyte differentiation [26] shown to impact obesity and related metabolic ailments, such as insulin resistance [27]. PCSK8 plays an important role in the regulation of lipid and cholesterol metabolism by cleaving sterol regulatory element-binding transcription factors (SREBP-1 and SREBP-2) [28, 29]. PCSK9 is the ninth and last member of the proprotein convertase family mainly expressed in the liver and small intestine; its function is essential in regulation of cholesterol and lipid homeostasis by degradation of LDLR (LDL receptor) [30, 31]. Previous research has shown the gain of function mutations and loss of function mutations in *PCSK9* to be associated with hypercholesterolemia and hypocholesterolemia, respectively [30–33].

The research to date has focused on the structure of PCSKs, their physiological functions, and their association with diseases. However, the evolutionary history and diversification of these proteins have remained unelucidated. The current study aims to analyze gene loss events, the differential rate of evolution, and the sites of positive selection in several *PCSK*s family members, i.e., *PCSK1*, *PCSK3*, *PCSK5*, *PCSK7*, *PCSK8*, and *PCSK9*, across placental mammals.

## Material and methods

### Sequence retrieval and orthologue identification

PCSKs *Homo sapiens* protein sequences (PCSK1: NP_000430.3, PCSK3: NP_001276752.1, PCSK5: NP_001177411.1, PCSK7: NP_004707.2, MBTPS1:NP_003782.1, PCSK9: NP_777596.2) were used as queries to identify orthologous proteins in placental mammals using NCBI blastp (E-values < 1e^−10^). Accession numbers of the identified orthologues were recorded, and the coding and protein sequences were retrieved using ENTREZ-direct and e-fetch tools of NCBI. The complete list of accession numbers for the taxon names and *PCSK* orthologues is provided in (S1 Table). In cases where a PCSK orthologous sequence was not identified in the complete set of protein sequences in a species, we performed tblastn against its genomic sequence using human and camel query sequences. Additionally, for these species, a Trace Archive Nucleotide blastn was undertaken using human and camel coding and genomic sequences as queries against the EST and WGS database, respectively. Finally, Ensembl blast was conducted against the complete protein and nucleotide databases for species with an undetected *PCSK* gene.

### Blat and synteny analysis

A Blat analysis was undertaken to identify remnants of *PCSK9* genes suspected to have been lost in *Ovis aries* (domestic sheep), *Bos taurus* (domestic cattle), *Leptonychotes weddellii* (weddell seal), *Ursus maritimus* (polar bear*)*, *Mustela putorius furo* (european domestic ferret), *Felis catus* (domestic cat), *Rousettus aegyptiacus* (egyptian rousette), *Pteropus vampyrus* (large flying fox), *Eptesicus fuscus* (big brown bat), *Miniopterus natalensis*, *Sorex araneus* (common shrew), and *Erinaceus europaeus* (cape elephant shrew). Human *PCSK9* mRNA and genomic sequences were submitted to Blat search in UCSC and Ensembl genome browsers (http://genome.ucsc.edu/index.html and https://ensembl.org/index.html) against the latest available version of the sequenced genomes of the abovementioned species. Synteny analysis was undertaken to assess the architecture of the genomic region of the detected putative remnant sequences to further determine whether undetected *PCSK9* genes are truly lost.

### Phylogenetic analysis

The MUSCLE algorithm in mega6 software was used to align species coding and protein sequences [34, 35]. Low quality and partial sequences were omitted from the analysis. The distances were estimated, and unrooted phylogenetic trees were constructed in MEGA6 using the maximum likelihood method. For the statistical support, 1000 bootstrap replicates were used to obtain trees. The Tamura-Nei model was used with gamma distributions in tree construction. Species trees were constructed utilizing the ETE toolkit [36]. For constructing phylogenetic trees, marsupial mammals, including *Monodelphis domestica* (gray short-tailed opossum), *Sarcophilus harrisii* (tasmanian devil), and *Vombatus ursinus* (common wombat), were used as out-groups. *Red junglefowl* (gallus gallus) and *Ornithorhynchus anatinus* (platypus) were applied as out-group for *MBTPS*1 and *PCSK7* to obtain the correct topology, respectively.

### Natural selection analysis

CodeML program in the PAML v4.8 package was used to estimate the rate of evolutionary alterations in codons estimated as ω (dN/dS = nonsynonymous/synonymous substitutions) [35]. The likelihood of the model M0 (neutral model with assumption of a fixed value of ω for all codon sites) was compared with the alternative model M3 (discrete model with assumption of different ω values among sites in the 0–1 range) to assess the variation of ω among codon sites. This comparison was made using The Likelihood Ratio Test (LRT). Recurrent positively selected sites were identified by comparing the likelihood of the M7 model (neutral model with beta distribution for ω in the 0–1 range) and the M8 model (selection model allowing ω>1 in a beta distribution). The ω ratio indicated negative purifying selection (0<ω<1), neutral evolution (ω = 1), and positive selection (ω>1) [37]. For *PCSK9*, the PAML analysis was performed on 54 mammalian species, and for the remainder, 45 species were entered in the analysis.

### Branch site model

Branch-site test was performed to detect positively selected sites along specific lineages of the phylogenetic trees. One branch was selected as the foreground while the remainder branches were indicated as background. To compare branch site model against null model, model: 2 and NS sites: 2 with flexible ω value was set in CodeML program in the PAML software for branch site test, and for the null test a similar setting was used with an ω value fixed at 1. The Bayes-empirical Bayes (BEB) method was used to calculate the posterior probability for sites which assumed to undergo positive selection. The sites with ω > 1 and posterior probabilities higher than 95% were determined as positive selection sites.

### Clade C model tests

C model (CmC) test was undertaken to determine divergent evolution in *PCSK*s along selected clades. For this purpose, ω ratio was calculated for selected clades using C model test (model: 3, with NS sites: 2) in CodeML program in the PAML software and was compared against a null model 2a_rel (M2a_rel) (model: 0, NSsite: 22). The target clade was indicated as the foreground clade, while the rest of dataset was presumed as the background. According to previous studies M2a_rel is a more accurate null model than the M1a in Clade C analysis [38].

### LRT test

Likelihood values were used for the statistical comparison of the two models. A log-likelihood (ln L) value for the null and alternative models was estimated by CODEML. Next, twice the difference of the log-likelihood between the alternative and the null model was calculated (2Δℓ). A chi-squared distribution was assumed for 2Δℓ with an appropriate degree of freedom (difference between the parameter number of the alternative and null models) [39].

### Provean analysis

The Protein Variation Effect Analyzer (PROVEAN) [40] and Sorting Tolerant From Intolerant (SIFT) [41] were employed to assess the functional effect of every amino acid change in the putative sites of positive selection. The confidence threshold of − 2.5 (Provean prediction) and 0.05 (SIFT prediction) was used to determine if an amino acid replacement is likely to affect protein function. The human *PCSK* genes sequences were used as a template, and every amino acid replacement present in each species was used as a query.

### 3D model prediction

Homology-modelling servers SWISS-MODEL [42] and I-TASSER [43] were used for homology-based 3D model prediction. This was performed for domains of PCSK1, FURIN, PCSK5 and PCSK7 with unresolved 3D structures (no structure available in PDB) that contained potential sites under positive selection. Successful prediction of 3D structure was only reached for part of PCSK1 C-terminal domain (aa 673–731) and FURIN pro-domain [aa 30–108] due to lack of homology of other submitted domains with the resolved 3D structures of various proteins available in protein structure databases.

## Results and discussion

### The identification of PCSK orthologues in placental mammals and PCSK9 putative loss in species of Artiodactyla, Carnivora, Chiroptera, Soricomorpha, and Erinaceomorpha orders, within conserved syntenic blocks

**Fig 2:**
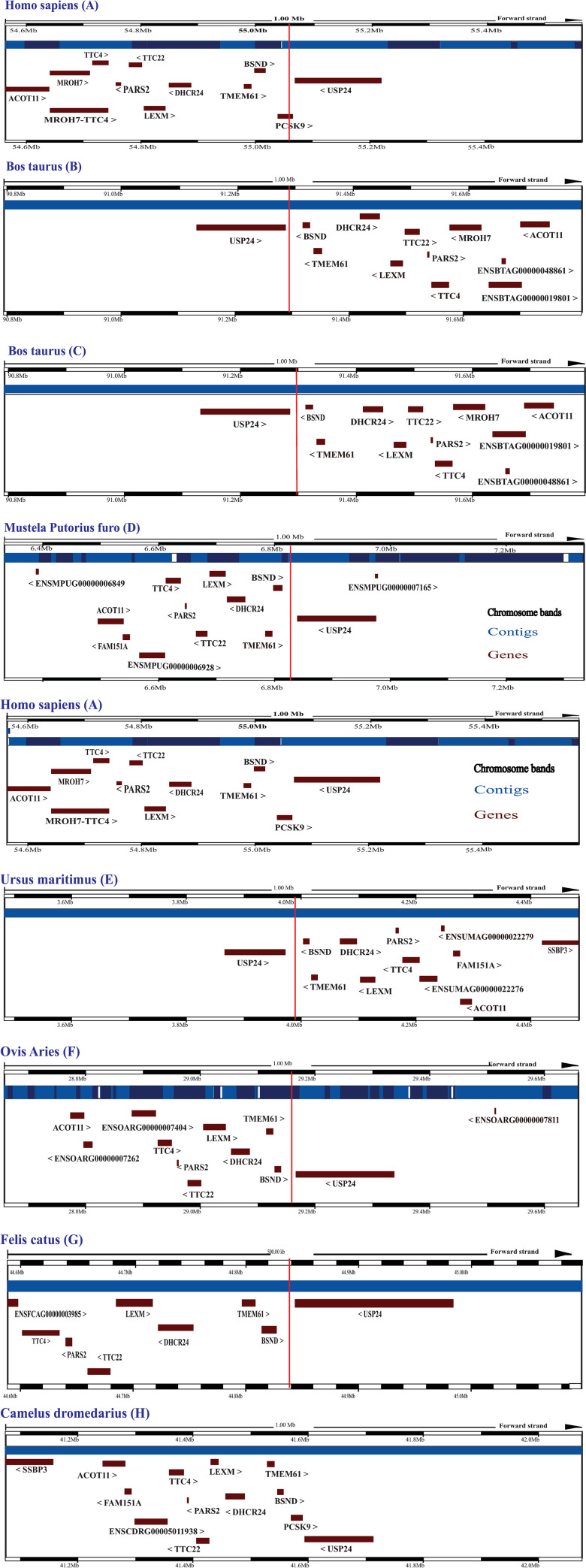
The comparative genomic regions surrounding the PCSK9 gene or remnants in the genome of the species with intact or putatively lost PCSK9. The red vertical lines indicate the genomic location of the PCSK9 remnants.

In all placental mammals analyzed, our NCBI and Ensembl blast identified orthologs for *PCSK1*, *PCSK3*, *PCSK5*, *PCSK7*, *MBTPS1*. In contrast, we did not identify any *PCSK9* gene in 12 species i.e., *Ovis aries*, *Bos Taurus* (*Artiodactyla* order) *Leptonychotes weddellii*, *Ursus maritimus*, *Mustela putorius furo*, *Felis catus* (*Carnivora* order), *Rousettus aegyptiacus*, *Pteropus vampyrus*, *Eptesicus fuscus*, *Miniopterus natalensis* (*Chiroptera* order), *Sorex araneus* (*Soricomorph* order) and *Erinaceus europaeus* (*Erinaceomorpha* order) (S1 Table). We performed blat analysis to identify remnants of *PCSK9* gene sequence in the genome of the species with putatively lost *PCSK9* using human *PCSK9* mRNA (3637 nucleotides (nt)) and genomic sequences. We found two hits on the *Bos taurus* (domestic cattle) chromosome 3 (3: 91294798–91294920, 339 nucleotides, 87.61% identity to *H*. *sapiens PCSK9* and 3:91293072–91293169, 270 nucleotides, 86.67% identity) (Fig 2B and 2C). One hit was identified on the *Mustela putorius furo* (european domestic ferret) genome (GL896928.1:6833708–6833760, 144 nt, 91.67% identity) (Fig 2D). One hit was also observed on the *Ursus maritimus* (polar bear) genome (KK498648.1:3983451–3983508, 162 nt, 87.04% identity) (Fig 2E) and on the *Ovis aries* (domestic sheep) chromosome 1 (1:29158676–29158796, 333 nt, 83.78% identity) (Fig 2F). Finally, on the *Felis catus* (domestic cat) chromosome C1, one hit was identified (C1:44838696–44838745, 135 nt, 88.89% identity) (Fig 2G). Synteny analysis was performed on the arrangement of the adjacent genes of *PCSK9* using human and camel genomic region composition as references, compared to the identified loci for *PCSK9* gene remnants in the abovementioned species. The results indicated conservation of the syntenic region, maintaining a similar composition in species with and without an intact *PCSK9* gene, further supporting the *PCSK9* gene loss suggested by our analysis in *Bos taurus* (domestic cattle), *Mustela putorius furo* (european domestic ferret), *Felis catus* (domestic cat), *Ursus maritimus* (polar bear), and *Ovis aries* (domestic sheep) (Fig 2B–2G, and S2 Table).

**Fig 3:**
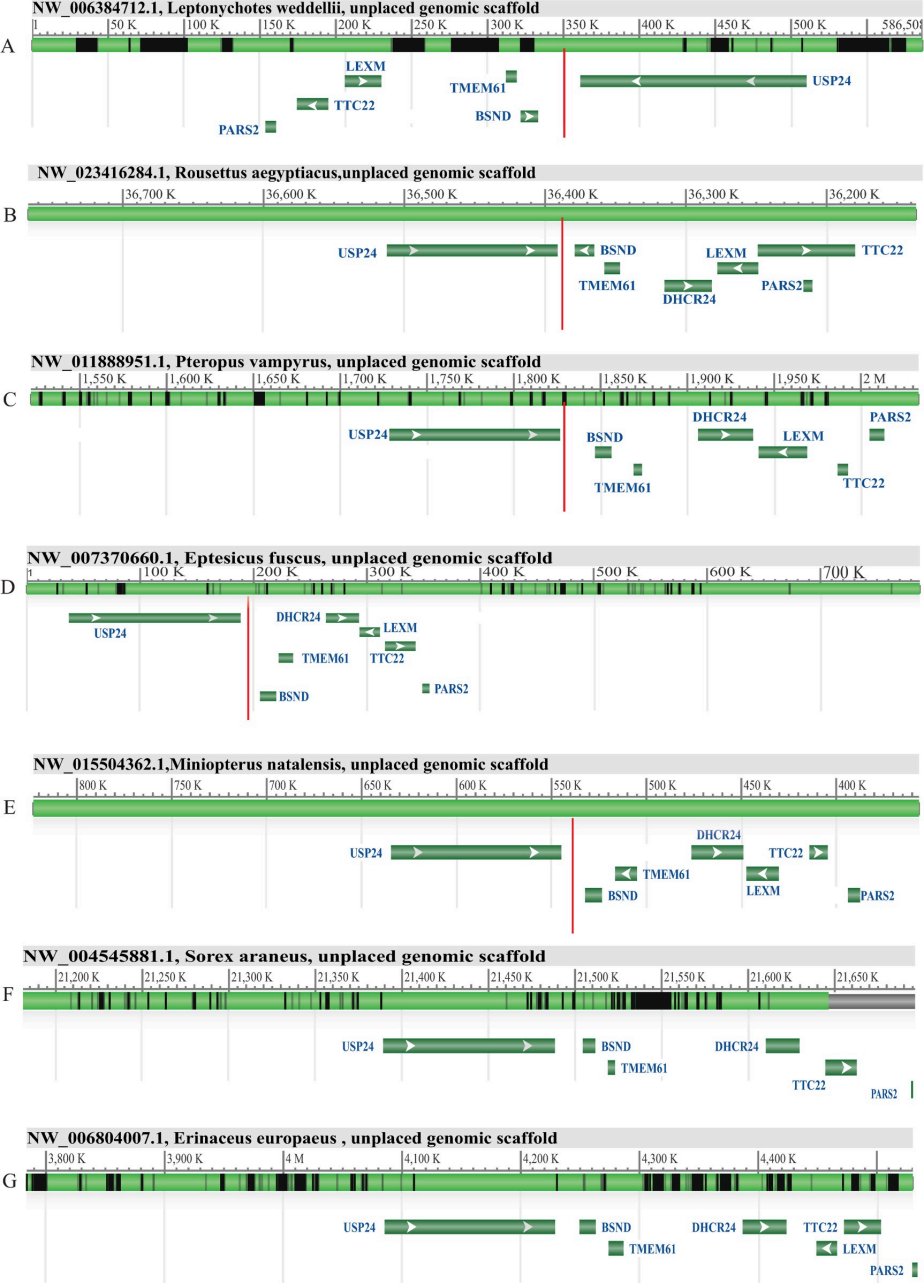
The comparative genomic region around PCSK9 gene remnants in species with putatively lost PCSK9. The red vertical lines indicate the genomic location of the PCSK9 remnants.

For *Leptonychotes weddellii* (weddell seal), *Rousettus aegyptiacus* (egyptian rousette), *Pteropus vampyrus* (large flying fox), *Eptesicus fuscus* (big brown bat), *Miniopterus natalensis* (natal long-fingered bat), *Sorex araneus* (common shrew) and *Erinaceus europaeus* (hedgehog) which genomes were not available in web BLAT tools of Ensembl and UCSC genome browsers, we searched for the two surrounding genes of *PCSK9*, *USP2*4, and *BSND* genes in NCBI (Fig 3A–3F and S3 Table) to find the syntenic block of *PCSK9* adjacent genes. According to the results, this region also displayed similar composition to the corresponding genomic region of *PCSK*9 in our references regarding the arrangement of neighboring genes. Next, to find the remnants of the putatively lost *PCSK9* gene, the genomic sequence of the region between *USP2*4 and *BSND* genes was downloaded for the aforementioned species, and Blastn was performed against this sequence using *H*. *sapiens PCSK9* mRNA sequence as a query (3637 nt). For *Leptonychotes weddellii* (weddell seal), one hit was found with 8 dispersed matching segments as remnants of *PCSK9*, covering 40% of query with 67.13% identity (Fig 3A). For *Rousettus aegyptiacus* (egyptian rousette), one hit was identified with 3 dispersed matching segments as remnants of *PCSK9*, covering 12% of query with 79.90% identity (Fig 3B). Further, for *Pteropus vampyrus* (large flying fox), one hit was identified with 3 dispersed matching segments, covering 16% of the query with 85.79% identity (Fig 3C). In the *Eptesicus fuscus* (big brown bat) genome, one hit with 6 dispersed matching segments was found, covering 14% of query with 75.12% identity (Fig 3D). For *Miniopterus natalensis* (natal long-fingered bat), covering 5% of the query with 75.73% identity (Fig 3E). For *Sorex araneous* (common shrew) and *Erinaceus europaeus* (hedgehog), no significant hit was found in the interval sequence of *USP24* and *BSND* genes (Fig 3F and 3G), although the composition of the adjacent genes was maintained (S4 Table). Additionally, blastn analysis when queried pcsk9 gene sequence from human and camel against latest genome assembly of *Sorex araneous (SorAra2*.*0)* and *Erinaceus europaeus (EriEur2*.*0)*, did not identify a significant homologous region. This could be either due to the complete loss of the pcsk9 gene in these two species or low quality of their genome sequencing and/or assembly.

**Fig 4:**
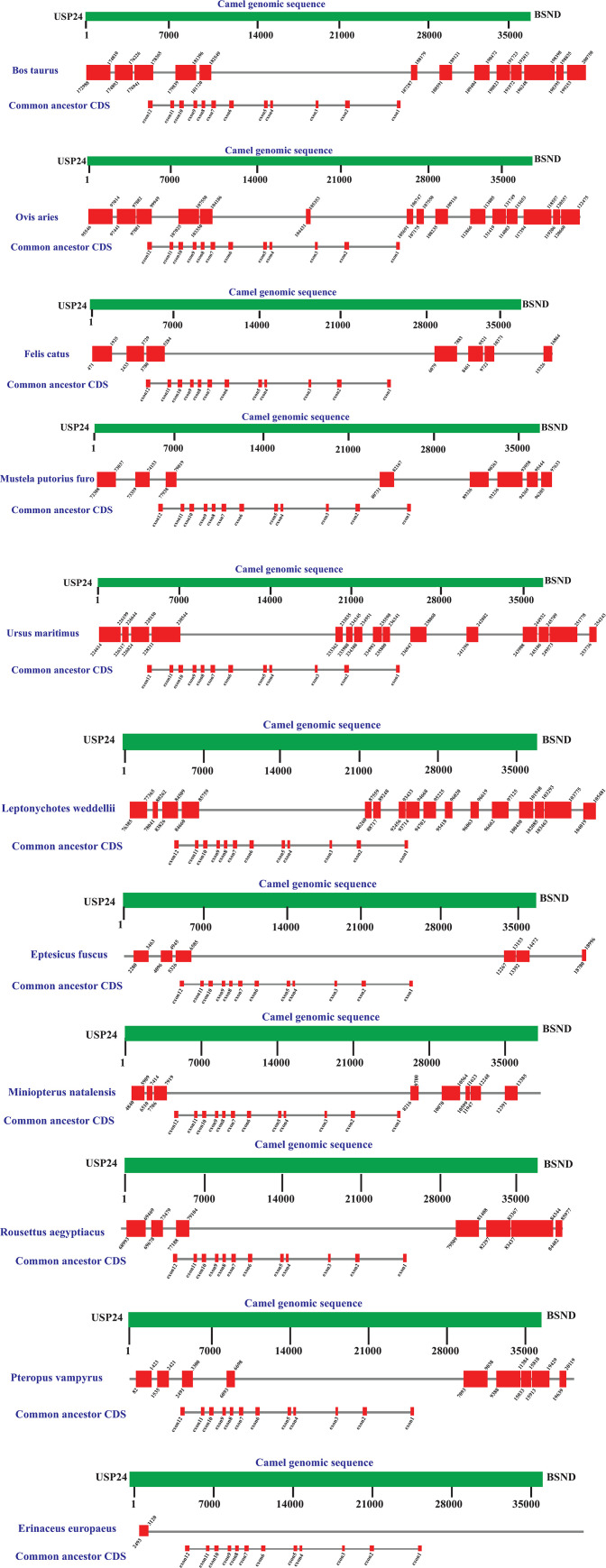
Blast comparison of genomic region between two adjacent genes of pcsk9 (BSND and USP24) in the syntenic block of Camelus Dromedarius and the indicated species with putative loss of pcsk9. Red blocks indicate regions with significant homology to query (intergenic region of USP24 and BSND in Camel). Numbers indicate the start and end position of the subject sequence (intergenic region of USP24 and BSND in the indicated species with putative loss of pcsk9).

In order to understand in detail how the structure of the pcsk9 gene is changed in species with putative loss of pcsk9, we used blastn to query the coding sequence of pcsk9 in the common ancestor of species with lost pcsk9 (obtained by PAML analysis of rate ancestor, which was identical to camel pcsk9 coding sequence (S1 File), against the intergenic region of BSND and USP24 of camel as well as the species with the putative loss of pcsk9 (Fig 4) as subjects. Additionally, we queried the pcsk9 gene sequence of camel against the intergenic region of BSND and USP24 of the species with pcsk9 loss to find homologous regions (S2–S25 Files). Assessing the results of these two blasts, our results indicate that in *Ursus maritimus* (Polar Bear) exons 3 to 10 are deleted (Fig 4), and also the nucleotide corresponding to the A in the start codon ATG is deleted. In *Leptonychotes weddellii* (Weddell seal), exons 3 to 12 are deleted (Fig 4), and also in codon 10 (corresponding camel and common ancestor sequence = TGG) is changed to TGA, leading to introduction of a premature stop codon (S25 File). In bats *Miniopterus natalensis* and *Eptesicus fuscus* all exons are lost, but homologous regions in the upstream and downstream sequences exist between the intergenic region (BSND and USP24) of the bat compared to camel intergenic region, which further supports the synteny conservation of the surrounding region. In bats *Rousettus aegyptiacus* exon 8, and *Pteropus vampyrus* exons 8 and 12 are conserved, while the rest of the exons are deleted. Again, the homology to the surrounding regions suggests a conservation of synteny of surrounding regions with the loss of the pcsk9 gene. In Eutheria animals *Bos taurus* (cow) and *Ovis Aries* (sheep), exons 1 to 7 are lost in addition to exon 11. In cat (*Felis catus*), exons 1 to 11 are all lost, while a significant homology remains in the upstream and downstream regions corresponding to pcsk9 gene between the BSND and USP24 intergenic regions of cat and camel, further supporting the conservation of the synteny of this region while pcsk9 gene is lost in cat. In ferret (*Mustela putorius furo*), all exons are lost except for exon 6, while the synteny and homology of the surrounding regions is existent.

Among the putative *PCSK9* gene loss events found here, lack of an intact *PCSK9* gene in *Bos taurus* has been indicated by a previous study demonstrating lack of expression of PCSK9 protein in this species, and the presence of a premature stop codon in exon 10 in the bovine’s *PCSK9* similar loci on the chromosome 3 is suggested to be a pseudogene of *PCSK9* [44].

### Phylogenetic analysis of PCSK family in mammals

**Fig 5:**
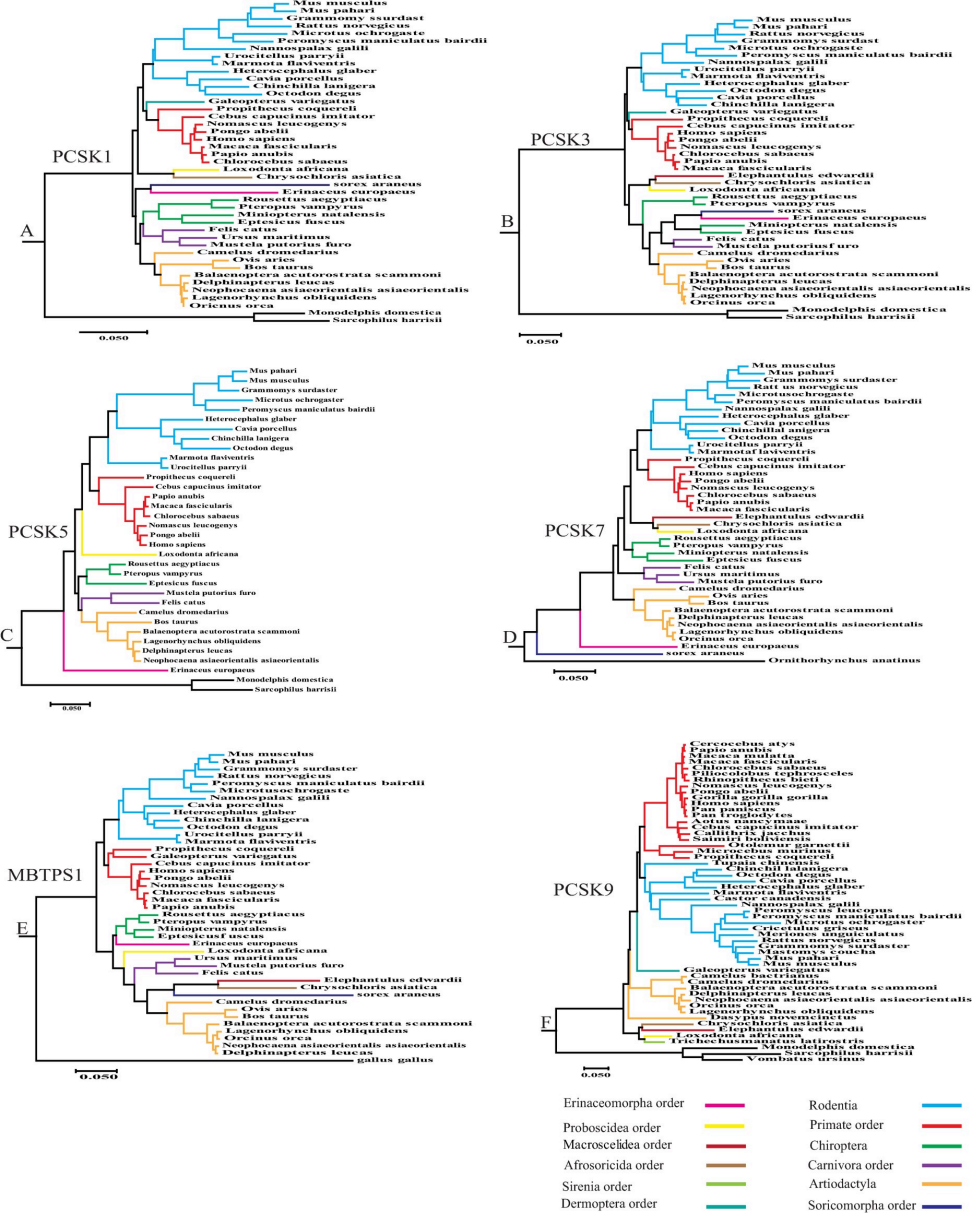
PCSKs Phylogenetic trees. Phylogenetic trees constructed by Maximum Likelihood method analysis of the coding sequences of PCSKS (bootstrap 1000) in mammalian species belonging to major placental mammalian orders. Orders are indicated by different colors. Branch length scale represents number of 0.05 substitutions per site.

The variation in *PCSK*s’ orthologous sequences was investigated among major placental mammalian orders. The results demonstrated that the catalytic domain is the most conserved domain in all *PCSK*s genes and the C-terminal domain is the least conserved domain (S1–S6 Files). The sequences p-distances (the proportion of nucleotide sites at which two sequences being compared are different) [45] were in the range of 0.0027 to 0.3404 for *PCSK1*, 0.0026 to 0.3282 for *PCSK3*, 0.0075 to 0.4335 for *PCSK5*, 0.0039 to 0.4126 for *PCSK7*, 0.0036 to 0.3893 for *MBTPS1*, and 0.0028 to 0.7185 for *PCSK9*. The longest branches in the phylogenetic trees belonged to *Sorex araneus* (common shrew) in *PCSK1* and *MBTPS1*, *Erinaceus europaeus* [hedgehog] in *PCSK3* and *PCSK5*, *Elephantulus edwardii* (cape elephant shrew) in *PCSK7*, and *Dasypus novemcinctus* (the nine-banded armadillo) in *PCSK9* (Fig 5). *PCSK*5 has 2 isoforms; isoform *PCSK5B* was used in our phylogenetic and PAML analysis.

**Fig 6:**
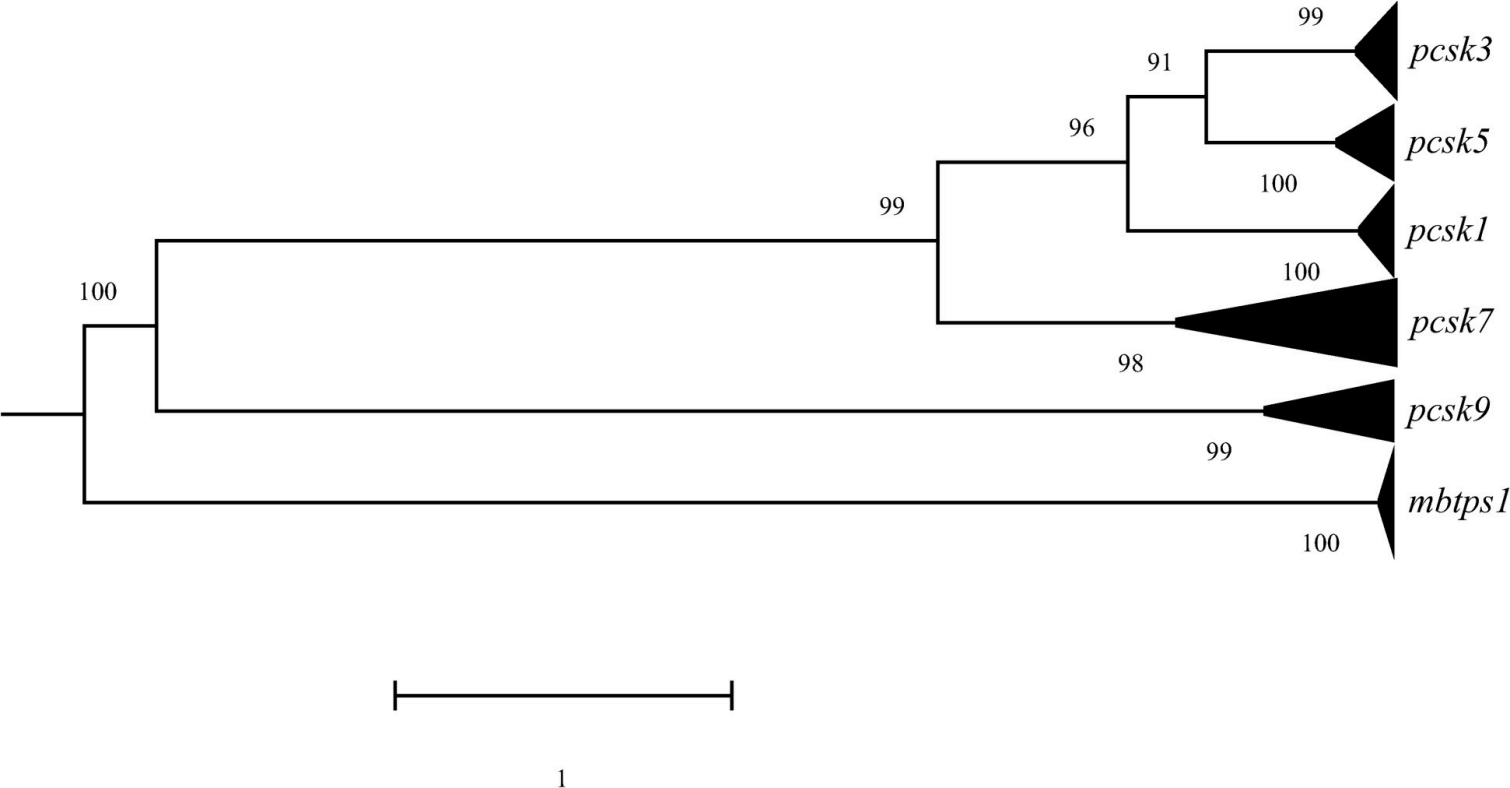
Phylogenetic analysis of six PCSKs. The analysis involves the codon sequences of each PCSK’s members from 14 species. Each branch is marked by its PCSK name on the right side. Branch length scale represents 1 substitution per site. The number written at each node represents the bootstrap value indicating the phylogenetic confidence of the tree topology.

To illustrate the evolutionary relationship between members of the *PCSK* family, codon sequences of 14 species were selected from major orders for each *PCSK* to build phylogenetic trees (Fig 6). The results showed *PCSK1*, *PCSK3*, and *PCSK5* to be the most closely related PCSK genes clustered into one clade with a high bootstrap value; whereas, *PCSK9* and *MBTPS1* were more distant to other *PCSK*s and constituted the periphery branches (Fig 6).

### Natural selection analysis

A site model test determined the rate of evolutionary changes in *PCSK* sites. Results of M0 and M8 models were compared with M3 and M7 models, respectively. The results indicated that the M3 model provided a better fit than that of M0 in all genes, suggesting a variable rate of evolution among codon sites (Table 1). The M8 model showed a significantly higher likelihood than M7 for all genes except *MBTPS1;* it determined sites with ω >1 (positive selection) in *PCSK1*, *PCSK3*, *PCSK5*, and *PCSK7* with a probability higher than 0.95 (Table 1).

**Table 1:** Parameter estimates and LRT test of the site models in pcsk1, pcsk3, pcsk5, pcsk7, mbtps1 and pcs.

Model	np	lnL	k	Parameter Estimates	Null	df	p-value	Corresponding sites of positive selection in *H*. *sapiens* pcsk1 (Probability (BEB))
***PCSK1***								
M0: One ω	85	-18999.494694	3.14174	ω:0.10575				
M3: discrete	93	-18349.383317	3.20776	P_0_:0.65728, P_1_:0.20582, P_2_:0.08867, P_3_: 0.04671, P4:0.00153	M0	8	<0.0005	
				ω_0_:0.00499, ω_1_:0.13276, ω_2_: 0.40954,ω_3_: 1.04662, ω_4_:3.36123				
M7: beta	86	-18387.374331	3.18431	P: 0.14005 q: 0.70068			<0.0005	
M8: beta & ω	88	-18354.600770	3.21008	P: 0.17615, q: 2.06230	M7	2		641 L 0.982*
				P_0_:0.94309, p_1_: 0.05691				642 V 0.972*
				ω:1.03802				679 S 0.999**
								680 P 0.981*
***PCSK3***								
M0: One ω	85	-19813.195649	4.73125	ω:0.04678				
M3: discrete	93	-19349.762353	4.85905	P_0_: 0.11639, P_1_: 0.49350, P_2_: 0.27823, P_3_: 0.09534, P4:0.01655	M0	8	<0.0005	
				ω_0_:0.00104, ω_1_: 0.00108, ω_2_: 0.05882,ω_3_:0.27071, ω_4_: 0.86706				
M7: beta	86	-19384.058249	4.84748	P:0.16658 q:1.81454			<0.0005	
M8: beta & ω	88	-19358.478946	4.86640	P: 0.18402, q:3.07492	M7	2		25Q 0.986*
				P_0_:0.98184, p_1_:0.01816				
				ω:1.00000				42 P 0.951*
***PCSK5***								
M0: One ω	67	-48397.375404	2.92977	ω:0.17426				
M3: discrete	75	-46344.263456	3.15981	P_0_:0.16426, P_1_:0.36429, P_2_:0.16702, P_3_:0.21585, P4:0.08857	M0	8	<0.0005	
				ω_0_:0.00762, ω_1_:0.00767, ω_2_: 0.12416,ω_3_: 0.40916, ω_4_:1.14459				
M7: beta	68	-46379.055122	3.12888	P:0.18072 q:0.59018			<0.0005	
M8: beta & ω	70	-46347.072312	3.16077	P:0.23594, q:1.51063	M7	2		1133 R 0.950*
								1150 Q 0.980*
				P_0_:0.91640, p_1_:0.08360				1442 I 0.966*
				ω:1.16310				
***PCSK7***								
M0: One ω	85	-20969.511919	4.11675	ω:0.11015				
M3: discrete	93	-20253.512133	4.30913	P_0_: 0.04128, P_1_:0.46034, P_2_:0.25730,P_3_:0.19266, P_4_: 0.04842	M0	8	<0.0005	
				ω_0_:0.00000, ω_1_:0.00004, ω_2_:0.07696,ω_3_:0.33788, ω_4_:0.95649				
M7: beta	86	-20271.872466	4.30866	P:0.19053 q:1.03406			<0.0005	
M8: beta & ω	88	-20254.880057	4.31530	P:0.20748, q:1.82683	M7	2		731 P 0.956*
				P_0_:0.95473, p_1_:0.04527				
				ω:1.03802				
***MBTPS1***								
M0: One ω	87	-20993.902777	3.42131	ω:0.03126				
M3: discrete	95	-20746.639592	3.41964	P_0_:0.29416, P_1_:0.48011, P_2_:0.12682, P_3_: 0.05649, P_4_:0.04242	M0	8	<0.0005	
				ω_0_:0.00372, ω_1_:0.00373, ω_2_:0.09131,ω_3_: 0.09131, ω_4_:0.31772				
M7: beta	88	-20758.917788	3.42589	P:0.14584 q:2.93477			NS	
M8: beta & ω	90	-20757.442058	3.43333	P:0.146590, q:3.11420	M7	2		
				P_0_:0.99766, p_1_:0.00234				
				ω:1.00000				
***PCSK9***								
M0: One ω	107	-26328.831432	4.44071	ω:0.17317				
M3: discrete	115	-25218.554105	4.70109	ω_0_:0.18074, P_1_: 0.29957, P_2_: 0.26690, P_3_: 0.19645, P_4_:0.05633	M0	8	<0.0005	
				ω _0_:0.00000, ω _1_: 0.04714, ω_2_: 0.16801, ω_3_:0.42413, ω_4_:1.04324				
M7: beta	108	-25523.641210	4.61356	P:0.39931 q:1.34585			<0.0005	
M8: beta & ω	110	-25500.548814	4.64307	P:0.51201, q: 2.71000	M7	2		
				P_0_:0.93926, p_1_:0.06074				
				ω:1.02291				

np: number of parameters for each model, df: degree of freedom, NS: not significant.

*probability > 0.95

** probability > 0.99.

According to the M3 model, about 13.6%, 30.4%, and 25.2% of sites in *PCSK1*, *PCSK5*, and *PCSK9* showed a relaxation of purifying constraints, respectively (ω> = 0.4, Table 1). In *PCSK3*, only 1% of codon sites displayed ω> 0.4 (ω = 0.8), indicating high purifying pressure on around 90% of codon sites in this protein with ω lower than 0.06. Similarly, in *MBTPS1*, purifying selective pressure can be inferred for approximately 96% of codon sites (ω<0.1). In *PCSK7*, the proportion of sites with ω higher than 0.3 was around 24%. Overall, the natural selection analysis of the present study indicated that the purifying selective pressure (natural selection force to avoid the change of an amino acid residue at a given position) was relatively much higher in *PCSK3* and *MBTPS1* among the studied members of the pc family.

**Fig 7:**
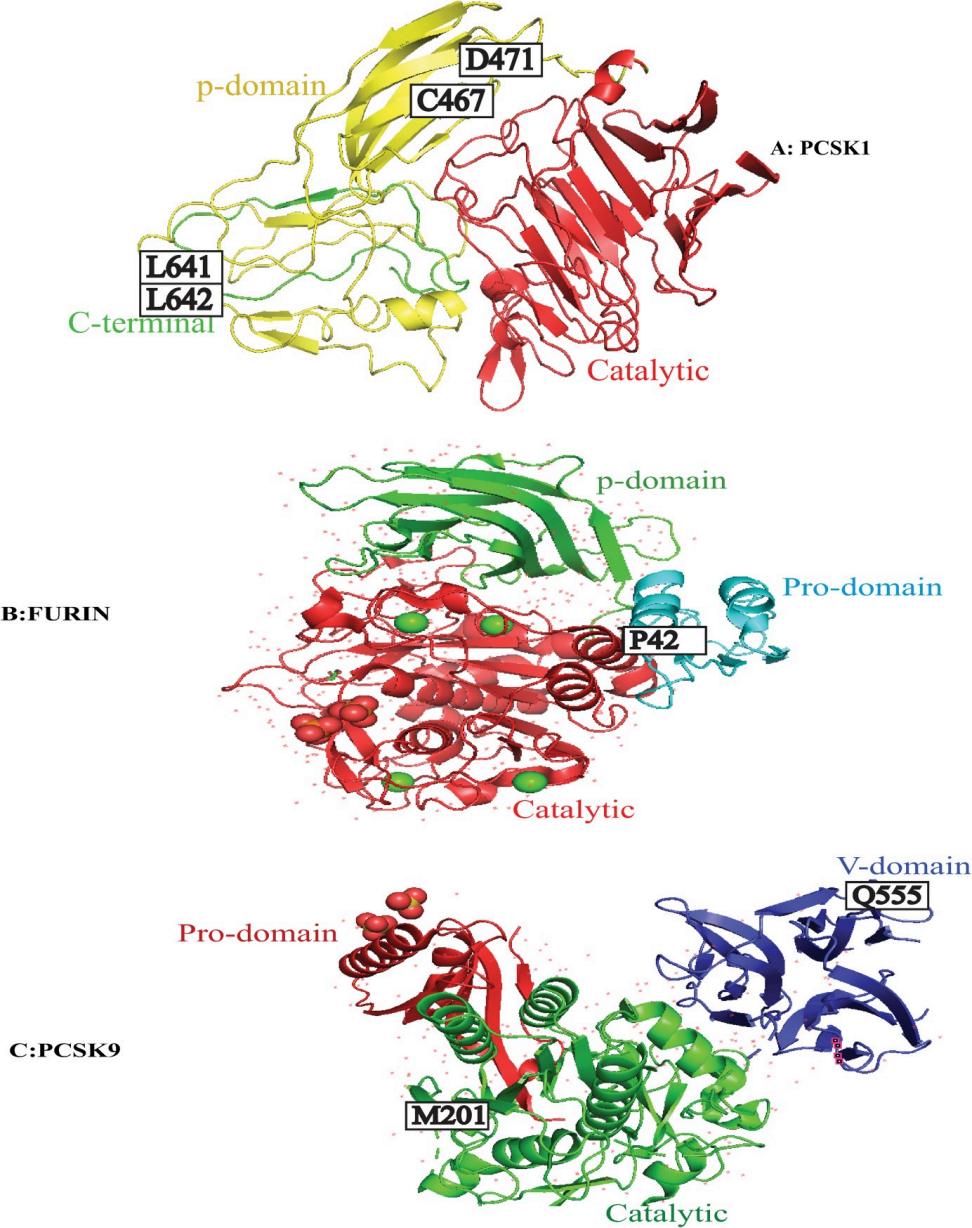
Sites under positive selection in the 3D structure of human PCSK9, PCSK1 and FURIN. A) Homology-based 3D model of PCSK1 catalytic (Red), p-domain (yellow) and part of C-terminal domain (green) (aa 673–731), with sites under positive selection indicated. B) 3D model of PCSK3(FURIN) catalytic (Red) and p-domain (green) (PDB: 4Z2A) and homology-based 3D model of pro-domain (blue) (aa30-108) with sites under positive selection indicated. C) 3D structure of human PCSK9 (PDB: 2PMW) catalytic, pro-domain and v-domain with sites under positive selection indicated.

PCSK1 is highly expressed in neuroendocrine and endocrine tissues, and its mutations are responsible for diseases such as hypoadrenalism, hypogonadism, obesity, malabsorptive diarrhea, and hypoglycemia due to its role in generating metabolically mature hormones and polypeptides, including glucagon, insulin, adrenocorticotropic hormones [46–48]. Positive selections were observed in 4 sites (641L, 642V, 679S, 680P) in the C-terminal domain of *PCSK1* (Fig 1A and Table 1). This domain is involved in PCSK1 sorting in to the secretory granules [49] and is important for PCSK1 oligomerization and stabilization [10, 50]. The corresponding amino acid to *H*. *sapiens* 641 in *PCSK1* was leucine in most species of the current study including *H*. *sapiens*; however, it was converted to proline in *Microtus ochrogaster* (prairie vole) and *Marmota flaviventris* (yellow-bellied marmot), glutamine in *Ovis aries* (domestic sheep) and *Bos taurus* (domestic cattle), as well as valine in *Cebus capucinus imitator* (white-faced capuchin) (Fig 7A). Valine at position 642 was converted to threonine in *Camelus dromedaries* [dromedary], *Rousettus aegyptiacus* (egyptian rousette), *Pteropus vampyrus* (large flying fox), *Eptesicus fuscus* (big brown bat), and *Erinaceus europaeus* (hedgehog), isoleucine in *Mus pahari* (gairdner’s shrewmouse) and *Cavia porcellus* (guinea pig), alanine in *Miniopterus natalensis* (natal long-fingered bat), *Mustela putorius furo* (european domestic ferret), *Sorex araneus* (common shrew), *Galeopterus variegatus* (sunda flying lemur), and *Artiodactyla* order, and methionine in *Felis catus* (domestic cat) and *Propithecus coquereli* (coquerel’s sifaka); the rest of animals had valine at this position (Fig 7A). At position 679, serine was located in the majority of animals; however, it was substituted with proline in *Balaenoptera acutorostrata scammoni* (north Pacific minke whale), *Marmota flaviventris* (yellow-bellied marmot), *Chinchilla lanigera* (long-tailed chinchilla), *Microtus ochrogaster* (prairie vole), *Urocitellus parryii* (arctic ground squirrel), *Chrysochloris asiatica* (cape golden mole), *Sorex araneus* (common shrew), and *Erinaceus europaeus* (hedgehog), leucine in *Galeopterus variegatus* (sunda flying lemur), *Propithecus coquereli* (coquerel’s sifaka), and *Loxodonta africana* (african bush elephant), phenylalanine in *Camelus dromedaries* (dromedary), valine in *Grammomys surdaster*, threonine in *Rattus norvegicus* (norway rat), as well as alanine in *Mus musculus* (house mouse) and *Mus pahari* (gairdner’s shrewmouse). According to sift prediction, the serine substitution at position 679 by phenylalanine was likely to damage protein function (sift score: 0.041) (S5 Table). Amino acid proline at position 680 was conserved in 28 species; however, it was converted to leucine in *Cavia porcellus* (guinea pig), *Octodon degus* (degu), *Chinchilla lanigera* (long-tailed chinchilla), *Grammomys surdaster*, *Mus musculus* (house mouse), *Mus pahari* (gairdner’s shrewmouse), and *Nomascus leucogenys* (white-cheeked gibbon), threonine in *Heterocephalus glaber* (naked mole-rat) and *Ursus maritimus* (polar bear), serine in *Chrysochloris asiatica* (cape golden mole), *Sorex araneus* (common shrew), and *Pongo abelii* (sumatran orangutan), as well as glutamine in *Galeopterus variegatus* (sunda flying lemur) (S26 File). It is not clear how the aforesaid amino acid substitution in 641L, 642V, 679S, and 680P sites of PCSK1 may have affected its function.

Previous studies demonstrated that nonsynonymous mutations in the C-terminal domain of *PCSK1*, such as SNP polymorphisms—Q665E - S690T-, to be associated with an increased risk of obesity [10, 51]. Here, glutamine 665 was found to be only present in *H*. *sapiens*. *In contrast*, in other species, it was glutamic acid (Q665E), except in the *Muridae* family, in which it was converted to lysine (Q665K) (S26 File). *H*. *sapiens* serine at position 690 was completely conserved in all placental mammals studied, except 3 species, including *Peromyscus maniculatus bairdii* (prairie deer mouse), *Microtus ochrogaster* (S690G), and *Chrysochloris asiatica* (S690N) (S26 File)

*Furin* is expressed in many tissues and functions in lipid metabolism, inflammatory response, cytokine secretion, and blood pressure regulation [52, 53]. Two sites corresponding to *H*. *sapiens* 25Q and 42P showed positive selection in *furin* in the analyzed species (Fig 1B and Table 1). Positive selection is the evolutionary driving force to fix advantageous mutations in genes which change their structural and functional properties for better fitness. The amino acids corresponding to *H*. *sapiens* codons 25 and 42 are located in the signal peptide and pro-domain, respectively. Signal peptide at the N-terminal of protein is removed from the pro-domain after translocating the nascent polypeptide chains from the cytoplasm into the endoplasmic reticulum. Pro-domain is responsible for the proper folding of the pro convertase protein [11]. Glutamine at position 25 was not conserved among mammalian species of our study and only 10 species including *Mus musculus* (house mouse), *Chrysochloris asiatica* (cape golden mole), *Eptesicus fuscus* (big brown bat) and *primate* order, had glutamine at this position. The rest of the animals had arginine, histidine, and cysteine at this position. Amino acid proline at position 42 was changed to leucine in *Neophocaena asiaeorientalis asiaeorientalis*, *Galeopterus variegatus* (sunda flying lemur), *Marmota flaviventris* (yellow-bellied marmot), *Cavia porcellus* (guinea pig), *Octodon degus* (degu), *Heterocephalus glaber* [naked mole-rat], and *Urocitellus parryii* (arctic ground squirrel), glutamine in *Elephantulus edwardii* (cape elephant shrew) and *Loxodonta africana* (african bush elephant), and tryptophan *in Peromyscus maniculatus bairdii* [prairie deer mouse], while other animals have proline at position 42 (S27 File and Fig 7B).

The role of PCSK5 in development, diabetes, fertility, lipid metabolism and cardiac complications was studied [54, 55]. *Pc5/B* was used for our PAML analysis and positive selections were observed in three sites of CRD i.e., 1133R, 1150Q, and 1442I (Fig 1C and Table 1). This domain consists of 22 repeated cysteine motifs and is important for protease stabilization [56, 57]. Arginine 1133 was conserved in the majority of species of the current research; however, it was converted to proline in *Eptesicus fuscus* (big brown bat), *Cavia porcellus* (guinea pig), *Mustela putorius furo* (european domestic ferret), and *Camelus dromedarius* (dromedary), glycine in *Microtus ochrogaster* (prairie vole), histidine in *Erinaceus europaeus* (hedgehog), and glutamine in *Bos taurus* (domestic cattle), *Cebus capucinus imitator* (white-faced capuchin) and *Chlorocebus sabaeus* (green monkey). Only *Erinaceus europaeus* (hedgehog), *Loxodonta Africana* (african bush elephant), *Papio Anubis* (olive baboon), and few rodents, such as *Grammomys surdaster*, *Mus musculus* (house mouse), *Mus pahari* (gairdner’s shrewmouse), *Peromyscus maniculatus bairdii* (prairie deer mouse), and *Microtus ochrogaster* (prairie vole), had glutamine at position 1150; and other animals had arginine, proline, leucine, glycine, histidine, and tryptophan at this location. The substitution of glutamine at position 1150 by tryptophan is predicted by SIFT analysis to be likely damaging to the protein function (sift score: 0.014) (S5 Table). At position 1442, only *Pongo abelii* (sumatran orangutan) and *Homo sapiens* had isoleucine and this amino acid is converted to threonine, glutamine, arginine, glutamic acid, lysine, leucine, methionine and tryptophan in other animals (S28 File).

PCSK7 is the most ancient member of this family, and its role in disease such as hypertension, neurological diseases, neoplasia, breast cancer, iron hemostasis, and insulin resistance has been investigated [27, 58, 59]. *PCSK7* showed positive selection in 1 site [731P], belonging to the cytoplasmic domain (Fig 1D and Table 1); this domain is important for pc7 commuting between trans-Golgi network and plasma membrane but also to enter endosomes for maximal activity [60, 61]. The proline 731 lies close to the ExEXXXL725 motif critical for endosomal sorting. This amino acid is converted to leucine in *Marmota flaviventris* (yellow-bellied marmot), *Cavia porcellus* (guinea pig), *Heterocephalus glaber* (naked mole-rat), *Mus musculus* (house mouse), *Mus pahari* (gairdner’s shrewmouse), *Rattus norvegicus* (norway rat), *Urocitellus parryii* (arctic ground squirrel), and *Nannospalax galili* (northern Israeli blind subterranean mole rat), glutamine in *Peromyscus maniculatus bairdii* (prairie deer mouse) and *Microtus ochrogaster* (prairie vole), leucine in *Chinchilla lanigera* (long-tailed chinchilla), alanine in *Sorex araneus* (common shrew), and valine in *Chrysochloris asiatica* (cape golden mole) (S29 File).

No positive selections were observed in *PCSK9* and *MBTPS1* in the studied mammalian species, according to the site-model test results (S30 and S31 Files).

### Carnivores display a divergent lower rate of evolution in *PCSK3*, *PCSK7*, *MBTPS1* genes; *Muridae* and rodents show an accelerated evolution of *PCSK1*, *PCSK7*, *MBTPS1*

For CMC analysis, five main clades were selected for CMC analysis, i.e., *Muridae*, bat, rodent, *Artiodactyla*, and *Carnivora*, as well as four subclades of *Artiodactyl*: *Balaenopteridae*, *Delphinidae*, *Monodontidae*, and *Phocoenidae* families (S1–S6 Figs). The results indicated that when the bat clade or *Muridae* family were chosen as the foreground in *PCSK1* analysis, the estimated ω was significantly higher than the background clades. In bat clade, the ω rate with the proportion of 20% was twice higher than the background (FG:ω_2_ = 0.47080, BG:ω_2_ = 0.22392, p-value <0.005). With a proportion of 21%, ω in *Muridae* clade was higher than the background (FG:ω_2_ = 0.39073, BG:ω_2_ = 0.22201, p-value <0.005) (Table 2).

**Table 2:** Parameter estimates for pcsk1, pcsk3, pcsk7 and mbtps1 Clade model C and the result of LRT tests.

Comparison	Genes name	Model	np	lnL	Model parameters	2 ΔlnL	*P-value*
*Chiroptera* order (bats)	*Pcsk1*	clade	89	-18346.839125	P_0_ = 0.73266, P_1_ = 0.06011, P_2_ = 0.207		
					BG: ω_0_ = 0.00989, ω_1_ = 1.00000, ω_2_ = 0.22392		
					FG: ω_0_ = 0.00989, ω_2_ = 1.00000, ω_2_ = 0.47080		
		M2A_rel	88	-18356.164585	P_0_ = 0.72705, P_1_ = 0.06248, p_2_ = 0.21048	18.65092	<0.0005
					ω_0_ = 0.00949, ω_1_ = 1.00000, ω_2_ = 0.23808		
*Muridae* family	*Pcsk1*	clade	89	-18352.516343	P_0_ = 0.72341, P_1_ = 0.06407, P_2_ = 0.21252		
					BG: ω_0_ = 0.00919, ω_1_ = 1.00000, ω_2_ = 0.22201		
					FG: ω_0_ = 0.00919, ω_2_ = 1.00000, ω_2_ = 0.39073		
		M2A_rel	88	-18356.164585	P_0_ = 0.72705, P_1_ = 0.06248, p_2_ = 0.21048	7.296484	<0.005
					ω_0_ = 0.00949, ω_1_ = 1.00000, ω_2_ = 0.23808		
*Carnivora* order	*Pcsk3*	clade	89	-19360.244607	P_0_ = 0.77600, P_1_ = 0.02033, P_2_ = 0.20367		
					BG: ω_0_ = 0.00714, ω_1_ = 1.00000, ω_2_ = 0.17210		
					FG: ω_0_ = 0.00714, ω_2_ = 1.00000, ω_2_ = 0.09495		
		M2A_rel	88	-19362.445586	P_0_ = 0.77822, P_1_ = 0.01993, p_2_ = 0.20186	4.401958	<0.05
					ω_0_ = 0.00728, ω_1_ = 1.00000, ω_2_ = 0.16951		
*Muridae* family	*Pcsk7*	clade	89	-20256.162247	P_0_ = 0.65950, P_1_ = 0.05691, P_2_ = 0.28359		
					BG: ω_0_ = 0.00919, ω_1_ = 1.00000, ω_2_ = 0.24502		
					FG: ω_0_ = 0.00919, ω_2_ = 1.00000, ω_2_ = 0.48386		
		M2A_rel	88	-20264.328358	P_0_ = 0.66331, P_1_ = 0.05486, p_2_ = 0.28183	442.658732	<0.0005
					ω_0_ = 0.00964, ω_1_ = 1.00000, ω_2_ = 0.26266		
*Carnivora* order	*Pcsk7*	clade	89	-20259.389833	P_0_ = 0.66140, P_1_ = 0.05580, P_2_ = 0.28280		
					BG: ω_0_ = 0.00942, ω_1_ = 1.00000, ω_2_ = 0.27149		
					FG: ω_0_ = 0.00942, ω_2_ = 1.00000, ω_2_ = 0.14542		
		M2A_rel	88	-20264.328358	P_0_ = 0.66331, P_1_ = 0.05486, p_2_ = 0.28183	9.87705	<0.0025
					ω_0_ = 0.00964, ω_1_ = 1.00000, ω_2_ = 0.26266		
*Rodentia* order (rodents)	*Mbtps1*	clade	91	-20744.592954	P_0_ = 0.84739, P_1_ = 0.00194, P_2_ = 0.15067		
					BG: ω_0_ = 0.00673, ω_1_ = 1.00000, ω_2_ = 0.14315		
					FG: ω_0_ = 0.00673, ω_1_ = 1.00000, ω_2_ = 0.25288		
		M2A_rel	90	-20754.044053	P_0_ = 0.84328, P_1_ = 0.00188, P_2_ = 0.15484	18.902198	<0.0005
					ω_0_ = 0.00645, ω_1_ = 1.00000, ω_2_ = 0.16713		
*Carnivora* order	*Mbtps1*	clade	91	-20748.444017	P_0_ = 0.84410, P_1_ = 0.00198, P_2_ = 0.15392		
					BG: ω_0_ = 0.00650, ω_1_ = 1.00000, ω_2_ = 0.17706		
					FG: ω_0_ = 0.00650, ω_1_ = 1.00000, ω_2_ = 0.06792		
		M2A_rel	90	-20754.044053	P_0_ = 0.84328, P_1_ = 0.00188, P_2_ = 0.15484	11.200072	<0.0005
					ω_0_ = 0.00645, ω_1_ = 1.00000, ω_2_ = 0.16713		
*Cercopithecidae* family	*Pcsk9*	clade	111	-25523.740735	P0 = 0.52860, P1 = 0.08834, p2 = 0.38306		
					BG: w0 = 0.03167, w1 = 1.00000, w2 = 0.28212		
					FG: w0 = 0.03167, w1 = 1.00000, w2 = 0.14656		
		M2A_rel	110	-25525.738276	P_0_ = 0.52853, P_1_ = 0.08811, p_2_ = 0.38336	3.995082	<0.05
					w_0_ = 0.03161, w_1_ = 1.00000, w_2_ = 0.27935		

np: number of parameters for each model, NS: not significant (p-value > 0.05).

In *MBTPS1*, rodents displayed a significantly higher ω compared to the rest of the dataset (FG:ω_2_ = 0.25288, BG:ω_2_ = 0.14315, p-value <0.005) (Table 2). The ω rate was approximately twice higher than background clades when the *Muridae* family was selected as foreground in *PCSK7* gene analysis (FG:ω_2_ = 0.48386, BG:ω_2_ = 0.24502, p-value<0.005) (Table 2). These findings suggested that an accelerated evolutionary rate of PCSK genes, including *PCSK1*, *PCSK7*, and *MBTPS1*, in *Muridae* and rodents’ clades occurred during evolution. On the other hand, clade test results, when selecting the species of *Carnivora* order as the foreground in *PCSK3*, *PCSK7*, and *MBTPS1 genes* (No *PCSK9* sequences were found for species of *Carnivora* order), showed the ω rate to be lower than background clades. These findings demonstrated a decelerated evolutionary rate of *PCSK* genes (*PCSK3*, *PCSK7*, *MBTPS1*) in *Carnivora* compared to the rest of the phylogeny (Table 2). Furthermore, the CMC analysis for *PCSK9* showed the ω rate to be twice lower than background clades when *Cercopithecidae* (old world monkey) family was selected as the foreground clade (FG: ω_2_ = 0.14656, BG: ω_2_ = 0.28212, p-value <0.05) (Table 2). The functional relevance of the differential evolutionary rates among the clades mentioned above remains to be studied in the future.

No significant difference in evolutionary rate along Balaenopteridae, *Delphinidae*, *Monodontidae*, and *Phocoenidae* family clades was observed in PCSK genes studied (*PCSK1*, *PCSK3*, *PCSK5*, *PCSK7*, *MBTPS1*, and *PCSK9*) (S6–S11 Tables).

### Evaluation of the selection rate in *PCSK*s along the ancestral branch of diverse mammalian clades

Branch-site model tests were used to detect positive selection along the ancestral branch of various groups of mammalian species in *PCSK* genes (S12–S17 Tables). When the common ancestor of *Carnivora* clade was assigned as the foreground branch, about 0.64% codon sites displayed a significantly higher average rate of evolution compared to the background clades in *PCSK1* gene (P_2a_ = 0.00571, P_2b_ = 0.00074; BG: ω_2a_ = 0.03991, ω_2b_ = 1.00000; FG: ω_2a_ = 8.63637, ω_2b_ = 8.63637) (Table 3). According to the BEB analysis, two sites, including 467C and 471D (*homo sapiens*), in the P-domain of *PCSK1* showed positive selection (Fig 7A). P-domain is responsible for the regulation of calcium dependence of PCs and enzymatic activity [13]. These two sites encoded cysteine and aspartic acid, respectively, in all studied species, except *Ursus maritimus* (polar bear), which were converted to asparagine and leucine, respectively. This species is adapted to live in a cold high-energy demanding climate, justifying the lipid-rich diet of the animal [62]. It remains unclear whether the aforesaid amino acid changes in PCSK1 have contributed to the distinguished metabolic adaptations of a polar bear to extreme cold weather. According to Provean prediction, the serine substitution at position 467 by asparagine in a polar bear is a deleterious change (Provean score:-10.69) and likely to have a damaging (sift score: 0.000) effect on protein function (S5 Table).

**Table 3:** Parameter estimates for pcsk1, pcsk5, pcsk7 and pcsk9 branch site model.

Foreground branches	Genes name	Model	np	lnL	Model parameters	2 ΔlnL	*P-value*	Corresponding sites of Positive selection in *H*. *sapiens* pcsks (Probability (BEB))
*Carnivora* order	*Pcsk1*	null	87	-18463.810809	P_0_ = 0.86199, P_1_ = 0.11307, P_2a_ = 0.02205, P_2b_ = 0.00289	5.396356		
					BG:ω_0_ = 0.03923,ω_1_ = 1.00000, ω_2a_ = 0.03923, ω_2b_ = 1.00000			
					FG:ω_0_ = 0.03923,ω_1_ = 1.00000, ω_2a_ = 1.00000, ω_2b_ = 1.00000			
		Alternative	88	-18461.112631	P_0_ = 0.87933, P_1_ = 0.11421, P_2a_ = 0.00571, P_2b_ = 0.00074		<0.02	467 C 0.951*
					BG:ω_0_ = 0.03991,ω_1_ = 1.00000, ω_2a_ = 0.03991, ω_2b_ = 1.00000			
								471 D 0.998*
					FG:ω_0_ = 0.03991,ω_1_ = 1.00000, ω_2a_ = 8.63637, ω_2b_ = 8.63637			
*Artiodoctyla* order	*Pcsk5*	null	69	-46690.933085	P_0_ = 0.74686, P_1_ = 0.24558, P_2a_ = 0.00569, P_2b_ = 0.00187			
					BG:ω_0_ = 0.05186,ω_1_ = 1.00000, ω_2a_ = 0.05186, ω_2b_ = 1.00000			
					FG:ω_0_ = 0.05186,ω_1_ = 1.00000,ω_2a_ = 1.00000, ω_2b_ = 1.00000			
		Alternative	70	-46685.510459	P_0_ = 0.75182, P_1_ = 0.24625, P_2a_ = 0.00146, P_2b_ = 0.00048	10.845252	<0.001	1263Y 0.980*
					BG:ω_0_ = 0.05227,ω_1_ = 1.00000, ω_2a_ = 0.05227, ω_2b_ = 1.00000			
					FG:ω_0_ = 0.05227,ω_1_ = 1.00000,ω_2a_ = 7.92140, ω_2b_ = 7.92140			
*Muridae* family	*Pcsk7*	null	87	-20455.310597	P_0_ = 0.78358, P_1_ = 0.14097, P_2a_ = 0.06395, P_2b_ = 0.01151	4.536734		
					BG:ω_0_ = 0.04548,ω_1_ = 1.00000, ω_2a_ = 0.04548, ω_2b_ = 1.00000			
					FG:ω_0_ = 0.04548,ω_1_ = 1.00000,ω_2a_ = 1.00000, ω_2b_ = 1.00000			
		Alternative	88	-20453.042230	P_0_ = 0.81322, P_1_ = 0.14610, P_2a_ = 0.03449, P_2b_ = 0.00620		<0.05	598 E 0.982*
								652 I 0.964*
								659 T 0.999**
								662 P 0.976*
					BG:ω_0_ = 0.04591,ω_1_ = 1.00000, ω_2a_ = 0.04591, ω_2b_ = 1.00000			781 E 0.965*
					FG:ω_0_ = 0.04591,ω_1_ = 1.00000, ω_2a_ = 2.24719, ω_2b_ = 2.24719			
*Artiodoctyla* order	*Pcsk9*	null	109	-25784.241834	P_0_ = 0.78754, P_1_ = 0.19724, P_2a_ = 0.01217, P_2b_ = 0.00305	55.823852		
					BG:ω_0_ = 0.10360,ω_1_ = 1.00000, ω_2a_ = 0.10360, ω_2b_ = 1.00000			
					FG:ω_0_ = 0.10360, = 1.00000, ω_2a_ = 1.00000, ω_2b_ = 1.00000			
		Alternative	110	-25756.329908	P_0_ = 0.79521, P_1_ = 0.20138, P_2a_ = 0.00272, P_2b_ = 0.00069		<0.0005	2 G 1.000**
								3 T 1.000**
					BG:ω0 = 0.10411,ω_1_ = 1.00000, ω_2a_ = 0.10411, ω_2b_ = 1.00000			
								555Q 0.996**
					FG:ω0 = 0.10411, ω_1_ = 1.00000,ω_2a_ = 446.22957,ω_2b_ = 446.2297			
*Balaenopteridae*, *delphinida*e, *monodontidae* and *phocoenidae* families from *Artiodoctyla* order	*Pcsk9*	null	109	-25783.486697	P_0_ = 0.78272, P_1_ = 0.19659, P_2a_ = 0.01654, P_2b_ = 0.00415	49.881076		
					BG:ω_0_ = 0.10361,ω_1_ = 1.00000, ω_2a_ = 0.10361, ω_2b_ = 1.00000			
					FG:ω_0_ = 0.10361,ω_1_ = 1.00000, ω_2a_ = 1.00000, ω_2b_ = 1.00000			
		Alternative	110	-25758.546159	P_0_ = 0.79405, P_1_ = 0.19722, P_2a_ = 0.00699, P_2b_ = 0.00174		<0.0005	2 G 1.000**
					BG:ω_0_ = 0.10459,ω_1_ = 1.00000, ω_2a_ = 0.10459, ω_2b_ = 1.00000			3 T 0.996**
								201 M 0.970*
					FG:ω_0_ = 0.10459,ω_1_ = 1.00000, ω_2a_ = 45.95946, ω_2b_ = 45.95946			555Q 1.000**

np: number of parameters for each model, NS: not significant. Positive selection sites are numbered according to the pcsk1 reference sequence in *H*. *sapiens* (NP_000430.3)

*probability >0.95

** probability >0.99.

Selecting the ancestral branch of *Artiodactyla* (cloven-hooved animals) clade as the foreground, the branch-site test showed the ω value to be differentially higher in approximately 0.19% sites of *PCSK5* gene (P_2a_ = 0.00146, P_2b_ = 0.00048; BG: ω_2a_ = 0.05227, ω_2b_ = 1.00000; FG: ω_2a_ = 7.92140, ω_2b_ = 7.92140). Further, positive selection with a probability higher than 95% was observed in codon 1263Y (*homo sapiens*) belonging to the CRD domain of *PCSK5*, which was converted to leucine (lagenorhynchus obliquidens), phenylalanine (balaenoptera acutorostrata scammoni), and serine (camelus dromedarius, bos taurus) (Table 3). According to Provean prediction, the tyrosine substitution by serine was a deleterious change (Provean score:-10.69) (S5 Table). The ω ratio in 4.06% of *PCSK7* codon sites was higher, running the branch-site test along common ancestor of species from *Muridae* family (P_2a_ = 0.03449, P_2b_ = 0.00620; BG: ω_2a_ = 0.04591, ω_2b_ = 1.00000; FG: ω_2a_ = 2.24719, ω_2b_ = 2.24719) (Table 3). The BEB analysis demonstrated that positive selections have occurred in 5 sites of *PCSK7*, including 598E, 652I, 659T, 662P, and 781E (*H*. *sapeins*). The amino acid corresponding to codon 598 is located in the P-domain responsible for structural stability and regulation of the enzymatic activity of PC7 [7]. The 652I, 659T, 662P, and 781E sites are located in the c-terminal domain. The C-terminal domain is divided into three sections named of variable, transmembrane, and cytoplasmic domains [7]. The amino acids corresponding to codons 652, 659, and 662 are located in the variable domain, and amino acid 781 belongs to the cytoplasmic domain. Studies have shown that the cytoplasmic domain plays an important role in the internalization and commuting of pc7 between trans-Golgi network and plasma membrane and endosomes [60, 61]. Amino acids corresponding to codons 598, 652, and 662 were completely conserved in the *Muridae* family, except *Mus pahari* (gairdner’s shrewmouse), in which they were converted to proline, tyrosine, and cysteine, respectively. According to sift prediction, the isoleucine substitution by tyrosine at position 652 (sift score: 0.043) and proline substitution at position 662 by cysteine (sift score: 0.002) were likely damaging to protein function (S5 Table). Threonine at position 659 was substituted by serin in *Rattus norvegicus* (norway rat) and glycine in *Mus pahari* (gairdner’s shrewmouse). Sift prediction showed the threonine substitution by glycine at position 659 to be likely damaging to protein function (sift score: 0.032) (S5 Table). At the corresponding amino acid to codon 781 (*H*. *sapiens*), glutamic acid was converted to aspartic acid in *Rattus norvegicus* (norway rat) *and Grammomys surdaster*, serine in *Mus pahari* (gairdner’s shrewmouse) and *Mus musculus* (house mouse) (S29 File).

When the common ancestor of *Artiodactyla* clade was selected as the foreground for the branch-site model test, the average ω value was differentially higher in 0.34% codon sites of *PCSK9* (P_2a_ = 0.00272, P_2b_ = 0.00069; BG: ω_2a_ = 0.10411, ω_2b_ = 1.00000; FG: ω_2a_ = 446.22957, ω_2b_ = 446.2297), and three sites of *PCSK9*, including 2G, 3T, and 555Q, underwent positive selection (*H*. *sapiens*) (Table 3). Furthermore, 0.87% of *PCSK9* codon sites exhibited significantly higher ω ratio (P_2a_ = 0.00699, P_2b_ = 0.00174; BG: ω_2a_ = 0.10459, ω_2b_ = 1.00000; FG: ω_2a_ = 45.95946, ω_2b_ = 45.95946), selecting the common ancestor of species from *Balaenopteridae*, *Delphinidae*, *Monodontidae*, and *Phocoenidae* families belonging to *Artiodactyla* order. According to the BEB analysis, five sites of *PCSK9*, including 2G, 3T, 201M, and 555Q (*homo sapiens*), revealed positive selection with a probability higher than 95% in the common ancestor of *Artiodactyla* order (Table 3). The amino acids corresponding to codons 2G and 3T (*homo sapiens*) are located in the signal peptide cleaved in the endoplasmic reticulum, and then autocatalytic processing occurs on PCSK9 [17, 63]. Glycine at position 2 was substituated by threonine (balaenoptera acutorostrata scammoni), alanine (neophocaena asiaeorientalis asiaeorientalis), and valine (delphinapterus leucas). According to sift prediction, mentioned substitutions were likely to damage the protein function (sift score: 0.000) (S5 Table). The sift prediction indicated that the threonine substitution at position 3 by histidine (balaenoptera acutorostrata scammoni) and arginine (neophocaena asiaeorientalis asiaeorientalis) was likely to damage protein function (sift score: 0.000) (S5 Table). Amino acid corresponding to codon 201 belongs to the catalytic domain, while the one corresponding to codon 555 is located in the Cys-His-rich domain, which is unique to PCSK9 protein [63] and is important for the regulation of PCSK9 auto-processing [64] (Fig 7C). PCSK9 plays an important role in cholesterol metabolism by degrading the LDL receptor. For this purpose, the pro- and catalytic domains bind to the EGF-A domain of LDLR. Binding increases in acidic pH and is completed following the binding of the c-terminal domain of PCSK9 to the ligand-binding domain of LDLR [65, 66]. Previous research has demonstrated that gain of function mutations in *PCSK9* are associated with hypercholesterolemia [30]. It is still unclear whether the differential evolutionary rate and the positive selections in *PCSK9* in *Artiodactyla* order may have contributed to their environmental or dietary adaptations during evolution.

## Conclusion

Rate of evolution was studied in members of the PCSK family using PAML analyses. The results showed the positive selection to occur in *PCSK1*, *PCSK3*, *PCSK5*, and *PCSK7*. Future studies are recommended to assess the functional relevance and selective evolutionary advantages associated with these modifications in PCSK proteins during evolution. Additionally, the data in this study suggested *PCSK9* gene putative loss in 12 species, including *Carnivores* and bats (*Chiroptera*). Moreover, the decelerated rate of evolution was observed in *PCSK7*, *PCSK3*, and *MBTPS1* in *Carnivores* compared to the rest of phylogeny. *Carnivores* as predator animals fed a high-fat diet. It remains unclear whether the high purifying pressure on the evolution of *PCSK3*, *PCSK7*, and *MBTPS1* genes or the loss of *PCSK9* may have contributed to the evolutionary adaptations of these animals to their high-fat diet. On the other hand, we did not identify an orthologue for *PCSK9* in species of bats (*Chiroptera* order). These animals are known to have undergone evolutionary adaptations modifying their lipid metabolism to increase the capacity of fat storage before hibernation, on which the animal relies for energy during this period [67]. In the future, it will be interesting to investigate whether the lack of the *PCSK9* gene might have had a beneficial impact on the unique metabolic adaptations and hibernating capacity of the *Chiroptera* order.

## Supporting information

S1 Fig PCSK1 species tree. The species tree constructed by ETE toolkit for 41 species belonging to 10 major mammals’ orders: The tested clades for PCSK1 PAML clade and branch-site analyses are indicated by different colors.
(EPS)

S2 Fig PCSK3 species tree. The species tree of furin constructed by ETE toolkit software for 41 species belonging to 11 major placental mammals’ orders. Marsupial mammals are considered as the out-group. Clades tested in the PAML analysis are shown by different colors.
(EPS)

S3 Fig PCSK5 species tree. The species tree of pc5-B, used for PAML tests, constructed by ETE toolkit software for 32 species of seven major mammalian orders: Two marsupial mammals are considered as the out-group. The tested clades are indicated by different colors.
(EPS)

S4 Fig PCSK7 species tree. The species tree of PCSK7 built by ETE toolkit software for 41 species of 10 major mammalian orders: Two marsupial mammals are considered as the out-group. The clades tested in PAML software are indicated by different colors.
(EPS)

S5 Fig MBTPS1 species tree. The species tree of MBTPS1 built by ETE toolkit software for 42 species belonging to 11 major mammalian orders. Two marsupial mammals are considered the out-group. The clades tested in PAML software are indicated by different colors.
(EPS)

S6 Fig PCSK9 species tree. The species tree of PCSK9 built by ETE toolkit software for 51 species of 10 major mammalian orders: Three marsupial mammals are considered the out-group. The clades analyzed in PAML software are indicated by different colors.
(EPS)

S1 File Species tree of indicated species with pcsk9 putative loss and similar species with intact pcsk9 gene. (NWK)

S2 File Blast output querying Camel pcsk9 gene sequence against the intergenic sequence of BSND and USP24 in Bos Taurus. Regions indicating changes in coding sequence or frame are highlighted (if applicable).
(PDF)

S3 File Blast output querying Camel pcsk9 gene sequence against the intergenic sequence of BSND and USP24 in Felis catus. Regions indicating changes in coding sequence or frame are highlighted (if applicable).
(PDF)

S4 File Blast output querying Camel pcsk9 gene sequence against the intergenic sequence of BSND and USP24 in Erinaceus europaeus. Regions indicating changes in coding sequence or frame are highlighted (if applicable).
(PDF)

S5 File Blast output querying Camel pcsk9 gene sequence against the intergenic sequence of BSND and USP24 in Rousettus aegyptiacus. Regions indicating changes in coding sequence or frame are highlighted (if applicable).
(PDF)

S6 File Blast output querying Camel pcsk9 gene sequence against the intergenic sequence of BSND and USP24 in Ovis aries. Regions indicating changes in coding sequence or frame are highlighted (if applicable).
(PDF)

S7 File Blast output querying Camel pcsk9 gene sequence against the intergenic sequence of BSND and USP24 in Miniopterus natalensis. Regions indicating changes in coding sequence or frame are highlighted (if applicable).
(PDF)

S8 File Blast output querying Camel pcsk9 gene sequence against the intergenic sequence of BSND and USP24 in Mustela putorius furo. Regions indicating changes in coding sequence or frame are highlighted (if applicable).
(PDF)

S9 File Blast output querying Camel pcsk9 gene sequence against the intergenic sequence of BSND and USP24 in Eptesicus fuscus. Regions indicating changes in coding sequence or frame are highlighted (if applicable).
(PDF)

S10 File Blast output querying Camel pcsk9 gene sequence against the intergenic sequence of BSND and USP24 in Sorex araneus. Regions indicating changes in coding sequence or frame are highlighted (if applicable).
(PDF)

S11 File Blast output querying Camel pcsk9 gene sequence against the intergenic sequence of BSND and USP24 in Ursus maritimus. Regions indicating changes in coding sequence or frame are highlighted (if applicable).
(PDF)

S12 File Blast output querying Camel pcsk9 gene sequence against the intergenic sequence of BSND and USP24 in Pteropus vampyrus. Regions indicating changes in coding sequence or frame are highlighted (if applicable).
(PDF)

S13 File Blast output querying Camel pcsk9 gene sequence against the intergenic sequence of BSND and USP24 in Leptonychotes weddellii. Regions indicating changes in coding sequence or frame are highlighted (if applicable).
(PDF)

S14 File Pcsk9 gene sequence of camel. Exons are indicated in red. Regions with homology to the intergenic sequence of BSND and USP24 in Bos Taurus are underlined.
(PDF)

S15 File Pcsk9 gene sequence of camel. Exons are indicated in red. Regions with homology to the intergenic sequence of BSND and USP24 in Felis catus are underlined.
(PDF)

S16 File Pcsk9 gene sequence of camel. Exons are indicated in red. Regions with homology to the intergenic sequence of BSND and USP24 in Erinaceus europaeus are underlined.
(PDF)

S17 File Pcsk9 gene sequence of camel. Exons are indicated in red. Regions with homology to the intergenic sequence of BSND and USP24 in Rousettus aegyptiacus are underlined.
(PDF)

S18 File Pcsk9 gene sequence of camel. Exons are indicated in red. Regions with homology to the intergenic sequence of BSND and USP24 in Ovis aries are underlined.
(PDF)

S19 File Pcsk9 gene sequence of camel. Exons are indicated in red. Regions with homology to the intergenic sequence of BSND and USP24 in Miniopterus natalensis are underlined.
(PDF)

S20 File Pcsk9 gene sequence of camel. Exons are indicated in red. Regions with homology to the intergenic sequence of BSND and USP24 in Mustela putorius furo are underlined.
(PDF)

S21 File Pcsk9 gene sequence of camel. Exons are indicated in red. Regions with homology to the intergenic sequence of BSND and USP24 in Eptesicus fuscus are underlined.
(PDF)

S22 File Pcsk9 gene sequence of camel. Exons are indicated in red. Regions with homology to the intergenic sequence of BSND and USP24 in Sorex araneus are underlined.
(PDF)

S23 File Pcsk9 gene sequence of camel. Exons are indicated in red. Regions with homology to the intergenic sequence of BSND and USP24 in Ursus maritimus are underlined.
(PDF)

S24 File Pcsk9 gene sequence of camel. Exons are indicated in red. Regions with homology to the intergenic sequence of BSND and USP24 in Pteropus vampyrus are underlined.
(PDF)

S25 File Pcsk9 gene sequence of camel. Exons are indicated in red. Regions with homology to the intergenic sequence of BSND and USP24 in Leptonychotes weddellii are underlined.
(PDF)

S26 File FAS file including the coding sequence of PCSK1. (FAS)

S27 File FAS file including the coding sequence of PCSK3. (FAS)

S28 File FAS file including the coding sequence of PCSK5. (FAS)

S29 File FAS file including the coding sequence of PCSK7. (FAS)

S30 File FAS file including the coding sequence of MBTPS1. (FAS)

S31 File FAS file including the coding sequence of PCSK9. (FAS)

S1 Table The overall distribution of the proprotein convertases family in placental mammals. A total of 6 members of the proprotein convertases family have been analyzed in 45 species. Proteins accession numbers are shown.
(DOCX)

S2 Table The characteristics of hits found for PCSK9 Blat analysis in the indicated species with putatively lost PCSK9. (DOCX)

S3 Table The genomic positions of Usp24 and BSND genes in the indicated species with putatively lost PCSK9 gene. (DOCX)

S4 Table The characteristics of hits found for PCSK 9 remnants in the indicated species with putatively lost PCSK9, using Blastn of PCSK 9 query against interval genomic sequences of USP24 and BSND genes. (DOCX)

S5 Table Provean and SIFT prediction of deleterious positive selections. Mutations with PROVEAN Score less than -2.5 are predicted to be deleterious. mutations with SIFT score less than 0.05 are predicted to be deleterious, while those greater than 0.05 are neutral.
(DOCX)

S6 Table Parameter estimates for PCSK1 Clade model C and the result of LRT tests. np: number of parameters for each model, NS: not significant (p-value > 0.05).
(DOCX)

S7 Table Parameter estimates for PCSK3 Clade model C and the result of LRT tests. np: number of parameters for each model, NS: not significant (p-value > 0.05).
(DOCX)

S8 Table Parameter estimates for PCSK5 Clade model C and the result of LRT tests. np: number of parameters for each model, NS: not significant (p-value > 0.05).
(DOCX)

S9 Table Parameter estimates for PCSK7 Clade model C and the result of LRT tests. np: number of parameters for each model, NS: not significant (p-value > 0.05).
(DOCX)

S10 Table Parameter estimates for MBTPS1 Clade model C and the result of LRT tests. np: number of parameters for each model, NS: not significant (p-value > 0.05).
(DOCX)

S11 Table Parameter estimates for PCSK9 Clade model C and the result of LRT tests. np: number of parameters for each model, NS: not significant (p-value > 0.05).
(DOCX)

S12 Table Parameter estimates for PCSK1 branch-site model. np: number of parameters for each model, NS: not significant; Positive selection sites are numbered according to the PCSK1 reference sequence in *H*. *sapiens* (NP_000430.3), *probability >0.95, ** probability >0.99.
(DOCX)

S13 Table Parameter estimates for PCSK3 branch-site model. np: number of parameters for each model, NS: not significant; Positive selection sites are numbered according to the PCSK3 reference sequence in *H*. *sapiens* (NM_001289823.1), *probability >0.95, ** probability >0.99.
(DOCX)

S14 Table Parameter estimates for PCSK5 branch-site model. np: number of parameters for each model, NS: not significant; Positive selection sites are numbered according to the PCSK 5 reference sequence in *H*. *sapiens* (NP_001177411.1), *probability >0.95, ** probability >0.99.
(DOCX)

S15 Table Parameter estimates for PCSK7 branch-site model. np: number of parameters for each model, NS: not significant; Positive selection sites are numbered according to the PCSK3 reference sequence in *H*. *sapiens* (NM_001289823.1), *probability >0.95, ** probability >0.99.
(DOCX)

S16 Table Parameter estimates for MBTPS 1 branch-site model. np: number of parameters for each model, NS: not significant; Positive selection sites are numbered according to the MBTPS1 reference sequence in *H*. *sapiens* (NP_001177411.1), *probability >0.95, ** probability >0.99.
(DOCX)

S17 Table Parameter estimates for PCSK 9 branch-site model. np: number of parameters for each model, NS: not significant; Positive selection sites are numbered according to the PCSK 9 reference sequence in *H*. *sapiens* (NM_174936.4), *probability >0.95, ** probability >0.99. NM_174936.4.
(DOCX)
