# Supplementary material for: Molecular evolution of PCSK family: Analysis of natural selection rate and gene loss
Source: PLoS One. 2021 Oct 28;16(10):e0259085. doi: 10.1371/journal.pone.0259085 (PMC8553125; doi:10.1371/journal.pone.0259085)
Supplement: S2 File — Regions indicating changes in coding sequence or frame are highlighted (if applicable). (PDF) [file pone.0259085.s008.pdf]

## COVID-19 Information

Public health information (CDC) | Research information (NIH)

SARS-CoV-2 data (NCBI) | Prevention and treatment information (HHS) | Español

BLAST ® » blastn suite-2sequences » results for RID-H1GDCN1K114

| | |
|---|---|
| Job Title | Nucleotide Sequence ... |
| RID | H1GDCN1K114 Search expires on 08-10 13:48 pm |
| Program | Blast 2 sequences |
| Query ID | lcl\|Query_48003 (dna) |
| Query Descr | None ... |
| Query Length | 20287 |
| Subject ID | lcl\|Query_48005 (dna) |
| Subject Descr | ref\|NC_037330.1\|:91112925-91486939 Bos taurus isolate L1 Dominette 01449 registration number 42190680 breed Hereford chromosome 3, ARS-UCD1.2, whole genome shotgun sequence ... |
| Subject Length | 374015 |

## Descriptions

| Description | Scientific Name | Max Score | Total Score | Query Cover | E value | Per. Ident | Acc. Len | Accession |
|---|---|---|---|---|---|---|---|---|
| ref\|NC_037330.1\|:91112925-91486939 Bos taurus isolate L1 Dominette 01449 registration number 42190680 breed Hereford chromosome 3, ARS-UCD1.2, whole genome shotgun sequence | | 1038 | 3850 | 21% | 0.0 | 75.40% | 374015 | Query_48005 |

»

## Graphic Summary

Distribution of the top 22 Blast Hits on 1 subject sequences

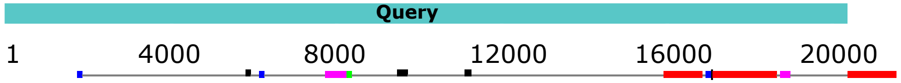


## Alignments

Alignment view Pairwise ☐ CDS feature Restore defaults

ref|NC_037330.1|:91112925-91486939 Bos taurus isolate L1 Dominette 01449 registration number 42190680 breed Hereford chromosome 3, ARS-UCD1.2, whole genome shotgun sequence

Sequence ID: Query_48005 Length: 374015 Number of Matches: 22

Range 1: 366619 to 366686

| Score | Expect | Identities | Gaps | Strand | Frame |
|---|---|---|---|---|---|
| 41.0 bits(44) | 0.004() | 51/69(74%) | 1/69(1%) | Plus/Plus | |

```
Query  1737    AGCCCTGGGGTTGAATCCTGGCTCCATCACCCACTAGCTCTGTGATGCTTGGCTCGTCAC  1796
               || ||| || || ||||||| |||   |||  | |||||||||||     |||  |||||
Sbjct  366619  AGACCTTGGATTCAATCCTGCCTCTGCCACTGAGTAGCTCTGTGACTGAGGGC-AGTCAC  366677

Query  1797    TTAACCTCT  1805
               |||||||||
Sbjct  366678  TTAACCTCT  366686
```

Range 2: 179722 to 179763

| Score | Expect | Identities | Gaps | Strand | Frame |
|---|---|---|---|---|---|
| 39.2 bits(42) | 0.012() | 35/43(81%) | 1/43(2%) | Plus/Plus | |

```
Query  5725    CTTGCCTGAGGTCACACAGCATAGAACTGGCAGGGCCAGAATT  5767
               ||||| || |||||||||||  |  |||||||| ||||| |||
Sbjct  179722  CTTGCGTG-GGTCACACAGCGGATGACTGGCAGAGCCAGGATT  179763
```

Range 3: 368463 to 368496

| Score | Expect | Identities | Gaps | Strand | Frame |
|---|---|---|---|---|---|
| 40.1 bits(43) | 0.012() | 29/34(85%) | 0/34(0%) | Plus/Plus | |

```
Query  5967    CTGTGTGCTGCAGACTCAAGGATGAACCAGACCC  6000
               || |||| ||  ||||||| ||||||||||||||
Sbjct  368463  CTTTGTGTTGGTGACTCAAAGATGAACCAGACCC  368496
```

Range 4: 307407 to 307733

| Score | Expect | Identities | Gaps | Strand | Frame |
|---|---|---|---|---|---|
| 170 bits(188) | 3e-42() | 244/340(72%) | 13/340(3%) | Plus/Plus | |

```
Query  7449    cttttttaatttttatttttttAATTGAAGTATAGTCAGTTCACAGTGTTGTGTAAATTTC  7508
               |||||||||    | | ||||| | ||||   |||  ||| ||||||| |||| ||||||
Sbjct  307407  CTTTTTTAAAAAATTTCTTTTA-TCGAAGACCAGTT-GTTTACAGTGTCGTGTTAATTTC  307464

Query  7509    TGGTGCAAAGCATAATGTTTCGGTCATACACATACATACATATATTCCTTTTCATATTCT  7568
               || || | |||| | ||        |  ||  || ||  |||||||| ||||||||||||
Sbjct  307465  TGCTGTACAGCAAAGTG--------ACCCAACTATATGTATATATTCTTTTTCATATTCT  307516

Query  7569    TTTTCACTATAGGTTATTACAAGCTATTGAATATAGTTCCCCGTGCTACACAGTAGGACC  7628
               |||||| | |   ||||||   | || ||| |||||||||| |||||| |||||||||||
Sbjct  307517  TTTTCATTGTGATTTATTATGGGATACTGAGTATAGTTCCCTGTGCTATACAGTAGGACC  307576

Query  7629    TTGCTGTTAATCTATTTTATATATAGCAGTTTGTATCTGCAAATGCCGATCTCCCAATTT  7688
               ||| |||| |||  || || |||||  |||||| |||| | ||| || | ||| ||||
Sbjct  307577  TTGTTGTTTATCCTTTCTAAATATAATAGTTTGCATCTACTAATTCCAAACTCTCAATCC  307636
```

```
Query  7689    ATCCCTCCATCCTCCTTCCAGCCCCGGGAACCACAAGtttgttttctatgtctgtgagtc  7748
               |||| ||   ||| |  |  |    || |||||||||| |||  ||| ||||||||||
Sbjct  307637  ATCCATCTGCCCTGCCACTCGGTTTGGCAACCACAAGTCTGTCCTCTCTGTCTGTGAG--  307694

Query  7749    tgtttctgtttttttaaataagttcatttgtgtctttttt  7788
               ||||| || |||  || ||||||  || |||||| |||||
Sbjct  307695  TGTTTTTG-TTTCATAGATAAGTGTATATGTGTCATTTTT  307733
```

Range 5: 166920 to 167251

| Score | Expect | Identities | Gaps | Strand | Frame |
|---|---|---|---|---|---|
| 180 bits(199) | 6e-45() | 255/354(72%) | 26/354(7%) | Plus/Plus | |

```
Query  7464    ttttttAATTGAAGTATAGTCAGTTCACAGTGTTGTGTAAATTTCTGGTGCAAAGCATAA  7523
               ||||||||||||||||||||   || ||| | |||||| | ||||  ||| | | || |
Sbjct  166920  TTTTTTAATTGAAGTATAGTTGATTTACAATATTGTGTTAGTTTCAAGTGTATAACAAAG  166979

Query  7524    TGTTTCGGTCATACACATACATACATATATTCCTTTTCATATTCTTTTTCACTATAGGTT  7583
               || ||| || |           | || ||||  ||||||  || || |  | ||||||||
Sbjct  166980  TGATTCAGTTA-----------ATAT-TATT--TTTTCAGGTTGTTGTGTATTATAGGTT  167025

Query  7584    ATTACAAGCTATTGAATATAGTTCCCCGTGCTACACAGTAGGACCTTGCTGTTAATCTAT  7643
                ||||||| || |||||||||||||  ||||| |||||||   ||||| |  || |||||
Sbjct  167026  GTTACAAGATACTGAATATAGTTCCGTGTGCTGCACAGTAAATCCTTGTTTCTAGTCTAT  167085

Query  7644    TTTATATATAGCAGTTTGTATCTGCAAATGCCGATCTC--CCAATTTATCCCTCCAT--C  7699
               || |||||||| ||||||| | ||  ||| ||   |||  | || ||| |||||| |
Sbjct  167086  TTCATATATAGTAGTTTGTGTTTGGTAATCCCATACTCCTCTAACTTACCCCTCCTTTCA  167145

Query  7700    CTCCTTCCAGCCCCGGGAACCACAAGtttgttttctatgtctgtgagtctgtttctgttt  7759
               | |||| |  ||| || ||||| ||||||||||||| |||||||| |||||||| ||  |
Sbjct  167146  CCCCTTTC--CCCTGGTAACCATAAGTTTGTTTTCTGTGTCTGTGTGTCTGTTTTTG-CT  167202

Query  7760    ttttaaataagttcatttgtgtcttttttttttAGATTCCACATATAAGTGATA  7813
               || || |||  ||| ||||     | || ||| ||| ||||||||||| |||||
Sbjct  167203  TTGTATATAGATTCTTTTG-----TATTATTTAAGACTCCACATATAAATGATA  167251
```

Range 6: 176859 to 176937

| Score | Expect | Identities | Gaps | Strand | Frame |
|---|---|---|---|---|---|
| 58.1 bits(63) | 5e-08() | 60/79(76%) | 0/79(0%) | Plus/Plus | |

```
Query  7531    GTCATACACATACATACATATATTCCTTTTCATATTCTTTTTCACTATAGGTTATTACAA  7590
               || || || ||| | |||| | ||  |||| |  || |||| || |||||||||||||||
Sbjct  176859  GTGATTCAGATATACACATGTGTTATTTTTGAAGTTTTTTTCCATTATAGGTTATTACAA  176918

Query  7591    GCTATTGAATATAGTTCCC  7609
                 |||||| |||| |||||
Sbjct  176919  TATATTGACTATAATTCCC  176937
```

Range 7: 92069 to 92144

| Score | Expect | Identities | Gaps | Strand | Frame |
|---|---|---|---|---|---|
| 75.2 bits(82) | 2e-13() | 62/76(82%) | 0/76(0%) | Plus/Plus | |

```
Query  7541   TACATACATATATTCCTTTTCATATTCTTTTTCACTATAGGTTATTACAAGCTATTGAAT  7600
              || |||  ||||||  ||| |||||| |||| || ||||||||||| |||| |||||| |
Sbjct  92069  TATATATGTATATTTTTTTCCATATTGTTTTCCATTATAGGTTATTGCAAGATATTGAGT  92128
```

```
Query  7601   ATAGTTCCCCGTGCTA  7616
              ||| ||||| ||||||
Sbjct  92129  ATAATTCCCTGTGCTA  92144
```

Range 8: 308041 to 308309

| Score | Expect | Identities | Gaps | Strand | Frame |
|---|---|---|---|---|---|
| 171 bits(189) | 3e-42() | 213/288(74%) | 23/288(7%) | Plus/Plus | |

```
Query  7544    ATACATATATTCCTTTTCATATTCTTTTTCACTATAGGTTATTACAAGCTATTGAATATA  7603
               ||| || ||||| ||||||||||| ||| || ||| | |||| ||| | |||||||| ||
Sbjct  308041  ATATATGTATTCTTTTTCATATTCCTTTCCATTATGGTTTATCACAGGATATTGAATGTA  308100

Query  7604    GTTCCCCGTGCTACACAGTAGGACCTTGCTGTTAATCTATTTTATATATAGCAGTTTGTA  7663
               || ||| |||||  ||  ||||||||    | || |||| || | | ||||  |||||| |
Sbjct  308101  GTCCCCTGTGCTGTACCATAGGACCT---TATTTATCTGTTCTGTGTATAATAGTTTGCA  308157

Query  7664    TCTGCAAATGCCGATCTCCCAATTTATCCCTCCATCCTCCTTCCAGCCCCGGGAACCACA  7723
               | ||| ||| || | ||||||    |||| ||| ||| |  ||        | |||||||
Sbjct  308158  TATGCTAATCCCAAGCTCCCA----ATCCTTCCCTCCCCTGTC--------GCAACCACA  308205

Query  7724    AGtttgttttctatgtctgtgagtctg-tttctgtttttttaaataagttcatttgtgtc  7782
               ||| |||| ||| |||||||| ||||| |||||| |||  || ||| ||| |||||||||
Sbjct  308206  AGTCTGTTCTCTGTGTCTGTGGGTCTGTTTTCTG-TTTCATAGATATGTTTATTTGTGTC  308264

Query  7783    ttttttttttAGATTCCACATATAAGTGATAGCAT---GGATTTTTCT  7827
                   |  |||||||||||||||||||||||| |||   | ||||||||
Sbjct  308265  ---ATAATTTAGATTCCACATATAAGTGATATCATATGGTATTTTTCT  308309
```

Range 9: 169119 to 169373

| Score | Expect | Identities | Gaps | Strand | Frame |
|---|---|---|---|---|---|
| 172 bits(190) | 8e-43() | 201/271(74%) | 18/271(6%) | Plus/Minus | |

```
Query  7545    TACATATATTCCTTTTCATATTCTTTTTCACTATAGGTTATTACAAGCTATTGAATATAG  7604
               |||||||| |  |||||  |||||||| || || |||||||||||||  | |||| | ||
Sbjct  169373  TACATATACT--TTTTCTGATTCTTTTCCATTACAGGTTATTACAAGAAACTGAAGACAG  169316

Query  7605    TTCCCCGTGCTACACAGTAGGACCTTGCTGTTAATCTATTTTATATATAGCAGTTTGTAT  7664
               ||| | |||||| |||||||| | ||| |||| ||||||||| || | || |||||| ||
Sbjct  169315  TTCTCTGTGCTATACAGTAGGCCTTTGTTGTTTATCTATTTTGTACACAGTAGTTTGCAT  169256

Query  7665    CTGCAAATGCCGATCTCCCAATTTATCCCTC--CATCCTCCTTCCAGCCCCGGGAACCAC  7722
               |||| |||  |   |||| ||||||||| ||  || ||||||| |  |   || |||||
Sbjct  169255  CTGCTAATCTCACACTCCTAATTTATCCTTCCTCAACCTCCTTTCCACTTTGGTAACCAT  169196

Query  7723    AAGtttgttttctatgtctgtgagtctgtttctgtttttttaaataagttcatttgtgtc  7782
               |||||||||| | |||||| |           | ||||||| ||||||||| ||||| ||
Sbjct  169195  AAGTTTGTTTCCAATGTCTCT-----------TTTTTTTTTTAATAAGTTCTTTTGTATC  169147

Query  7783    ttttttttttAGATTCCACATATAAGTGATA  7813
                      ||||||| ||||||||||||||||
Sbjct  169146  ---ACACTTTAGATCCCACATATAAGTGATA  169119
```

Range 10: 36277 to 36410

| Score | Expect | Identities | Gaps | Strand | Frame |
|---|---|---|---|---|---|
| 57.2 bits(62) | 5e-08() | 98/140(70%) | 7/140(5%) | Plus/Minus | |

```
Query  7725   Gtttgttttctatgtctgtgagtctgtttctgtttttttaaataagttcatttgtgtctt  7784
              |||||||  || | ||||||||||   |||| | || ||| ||    ||| | || | ||
Sbjct  36410  GTTTGTTCACTTTATCTGTGAGTCCACTTCTTTGTTGTTATAT----TCACTAGTTTGTT  36355

Query  7785   tttttttt-AGATTCCACATATAAGTGATAGCATGGATTTTTCTTTCTCTTTCTGGCTTA  7843
              ||  |||| ||||||||||| |||||  || |||  |    | | ||| | |||| ||||
Sbjct  36354  TTGCTTTTCAGATTCCACATTTAAGTAGTATCAT--AACAGTATATCTTTCTCTGACTTA  36297

Query  7844   CTTCACTTGGTATGATGATC  7863
               ||||||| | || ||| ||
Sbjct  36296  TTTCACTTAGCATAATGCTC  36277
```

Range 11: 237795 to 237857

| Score | Expect | Identities | Gaps | Strand | Frame |
|---|---|---|---|---|---|
| 43.7 bits(47) | 0.001() | 49/65(75%) | 2/65(3%) | Plus/Plus | |

```
Query  7792    tAGATTCCACATATAAGTGATAGCATGGATTTTTCTTTCTCTTTCTGGCTTACTTCACTT  7851
               || |||||| ||||| | | ||  ||  |||  |||||||||||||| |||| |||| |
Sbjct  237795  TAAATTCCATATATATGGGTTAATAT--ATTGGTCTTTCTCTTTCTGACTTATTTCATTC  237852

Query  7852    GGTAT  7856
                ||||
Sbjct  237853  TGTAT  237857
```

Range 12: 178609 to 178653

| Score | Expect | Identities | Gaps | Strand | Frame |
|---|---|---|---|---|---|
| 37.4 bits(40) | 0.043() | 35/45(78%) | 0/45(0%) | Plus/Plus | |

```
Query  8975    CAAGCTGTGTGACCTTGGATAAGTCACTGACCGTCTCTGAGCCTC  9019
               || ||||||  ||||| |  ||| || | ||| ||||||||||||
Sbjct  178609  CATGCTGTGCCACCTTAGGAAAGCCATTCACCCTCTCTGAGCCTC  178653
```

Range 13: 196361 to 196405

| Score | Expect | Identities | Gaps | Strand | Frame |
|---|---|---|---|---|---|
| 46.4 bits(50) | 8e-05() | 37/45(82%) | 0/45(0%) | Plus/Minus | |

```
Query  8976    AAGCTGTGTGACCTTGGATAAGTCACTGACCGTCTCTGAGCCTCA  9020
               |||||||||||| |||| || |  ||||||| ||||||  |||||
Sbjct  196405  AAGCTGTGTGACTTTGGTTACGAGACTGACCCTCTCTGGTCCTCA  196361
```

Range 14: 197791 to 197834

| Score | Expect | Identities | Gaps | Strand | Frame |
|---|---|---|---|---|---|
| 49.1 bits(53) | 2e-05() | 37/44(84%) | 0/44(0%) | Plus/Minus | |

```
Query  8978    GCTGTGTGACCTTGGATAAGTCACTGACCGTCTCTGAGCCTCAG  9021
               ||||  ||| |||||||||||||||| || ||||||| | ||||
Sbjct  197834  GCTGGATGAACTTGGATAAGTCACTGCCCCTCTCTGATCTTCAG  197791
```

Range 15: 210213 to 210251

| Score | Expect | Identities | Gaps | Strand | Frame |
|---|---|---|---|---|---|
| 44.6 bits(48) | 3e-04() | 33/39(85%) | 0/39(0%) | Plus/Plus | |

```
Query  8978    GCTGTGTGACCTTGGATAAGTCACTGACCGTCTCTGAGC  9016
               |||||||||||||||| ||| |||| | | |||||| ||
Sbjct  210213  GCTGTGTGACCTTGGAAAAGCCACTCAACCTCTCTGGGC  210251
```

Range 16: 27751 to 27790

| Score | Expect | Identities | Gaps | Strand | Frame |
|---|---|---|---|---|---|
| 37.4 bits(40) | 0.043() | 32/40(80%) | 0/40(0%) | Plus/Plus | |

```
Query  8989   TTGGATAAGTCACTGACCGTCTCTGAGCCTCAGGTTCCTC  9028
              ||||| |||||| |   | |||||||||||||| || |||
Sbjct  27751  TTGGACAAGTCAGTTTGCCTCTCTGAGCCTCAGTTTTCTC  27790
```

Range 17: 218616 to 218675

| Score | Expect | Identities | Gaps | Strand | Frame |
|---|---|---|---|---|---|
| 38.3 bits(41) | 0.043() | 45/60(75%) | 1/60(1%) | Plus/Minus | |

```
Query  10446   ACCAGCTGTGTGACCTTGTGCA-GTTACTTACCCTTTCTGTGCCTCAGTTTCCTTGTCTG  10504
               ||| |||| | ||| |||  || || |||   ||| ||| ||||||||||||||  ||||
Sbjct  218675  ACCCGCTGAGGGACTTTGGACAAGTGACTGTACCTCTCTCTGCCTCAGTTTCCTCATCTG  218616
```

Range 18: 181720 to 182549

| Score | Expect | Identities | Gaps | Strand | Frame |
|---|---|---|---|---|---|
| 695 bits(770) | 0.0() | 679/859(79%) | 47/859(5%) | Plus/Minus | |

```
Query  15138   TGCCGCCCACGTGGCTGGTGAGTTGCTGCCCTACCACCTCAGCCACCGTGATTCTAACCA  15197
               |||||||||| || ||||||||| ||||||||       |||||| | ||||||| ||
Sbjct  182549  TGCCGCCCACATGACTGGTGAGTAGCTGCCCT-------CAGCCATCATGATTCTGACAG  182497

Query  15198   CCCCTTTGGGAGCCAGGATCTGCGCCAGAACCCCATGTGCCAGGCTCTGTGTTGGACACG  15257
               ||| ||||| || |||| |||||  ||| ||||| ||||||||||||||||||||    |
Sbjct  182496  CCCGTTTGGCAGGCAGGGTCTGCAACAGGACCCCTTGTGCCAGGCTCTGTGTTGGGTGTG  182437

Query  15258   GGGGACTAAAGAGGAATCAGACTGATGGTGCCCTCAAAGACTCTCAGTCTGATGGGTGAG  15317
               ||| |||||||| || |||||   ||||||||||||| | | |||||||||||| | |||
Sbjct  182436  GGG-ACTAAAGATGAGTCAGATAAATGGTGCCCTCAAGGTCACTCAGTCTGATGAGGGAG  182378

Query  15318   GCAGGTGCACAAACAGAGTAGCCAGGGCTGTGTGGAAGGGAGCCCAGAG-AGGTACCCAC  15376
               |||| ||||||    |||||||| ||||||||||||||||||||  ||| |||| ||  |
Sbjct  182377  GCAGATGCACA----GAGTAGCCGGGGCTGTGTGGAAGGGAGCCTGGAGGAGGTGCCTGC  182322

Query  15377   CCAGCTTAAAGGTCAGGGAAAGCTTCCTAG--------CATTTTATTTGGGGTTTGGTGG  15428
               |||   |||||||| ||||||  ||||  |        || || || | |||||| ||||
Sbjct  182321  CCACACTAAAGGTCGGGGAAAATTTCCCGGAGGAGGGACAATTCATCTTGGGTTTAGTGG  182262

Query  15429   ATGAATAGGAGTTTACCTGGCAAGCAAAACAGCAATAGTCAAGGCTCAGAGGTATGGGAG  15488
               |||||||  ||||||||||||||  ||||||||||  | |||||||||||||| || ||
Sbjct  182261  ATGAATAAAAGTTTACCTGGCAAATAAAACAGCAACTGCCAAGGCTCAGAGGTGTGAGAA  182202

Query  15489   CAGGATGTAAGATAGTCTTACTCTTTGGCTGTCTTTTAACC-TGGGGTTGCAGGTCTTTT  15547
```

```
                   |||||| ||||| |||  ||||||   ||||| |||||| | || | ||||||||  |||
Sbjct  182201  CAGGAT-TAAGAGAGTTGTACTCTA--GCTGTTTTTTAAGCATGAGATTGCAGGTGGTTT  182145

Query  15548   AACTTCTGAGGAACAGCCTGgtgtgtctctgtgcatgtgtgtgtgtg--tgtgtgtgtgc  15605
               || ||||||  |||||  |||||||||         ||||||   ||  |||||||||||
Sbjct  182144  AATTTCTGA--AACAGTTTGGTGTGTC------CAAGTGTGTACATGCATGTGTGTGTGC  182093

Query  15606   gcgcgcacgcgtgtgtgtACCAAGAGAGGAGTCCCAGATCCGGAAAGAGGGCCAGGCCAC  15665
                 | |     |||||||  ||| ||||||||||||||||| |||| ||||||  ||||||
Sbjct  182092  TTGGGTGTCTGTGTGTGCCCCAGGAGAGGAGTCCCAGATCGGGAAGGAGGGCTGGGCCAC  182033

Query  15666   CACTATCTCTCACTGCCCGTCCCACCACCAGGCATTGTGGCCATGATGCTGACGGCCGAG  15725
               | || |||||||||||| | ||||  ||||||| |||||| ||||||||||||  |||||
Sbjct  182032  CCCTGTCTCTCACTGCCTGCCCCATGACCAGGCGTTGTGGTCATGATGCTGACCTCCGAG  181973

Query  15726   CCGGAGCTCACCCTGGCTGAGCTGAGGCAGAGACTGATCCATTTCTCTGCCAAAGACGTC  15785
               |||||||||||||||||||||||||||||||| ||||||  ||||||||||||||| |||
Sbjct  181972  CCGGAGCTCACCCTGGCTGAGCTGAGGCAGAG-CTGATCTGTTTCTCTGCCAAAGATGTC  181914

Query  15786   ATCAACAAGGCCTGGT--TTCCC-GAAGACCAGCGGGTGCTGACCCCCAACCTGGTGGCC  15842
               ||||||||||||||||  ||||| || |||||||   |   || ||||||||||||||||
Sbjct  181913  ATCAACAAGGCCTGGTTCTTCCCTGAGGACCAGC---TCGGGAACCCCAACCTGGTGGCC  181857

Query  15843   ACACTGC--CCCCCAGAACCTATAAAGCAGGTCAGCAGGGCGGCAAGGTGGGCAGAATCC  15900
               |||| ||  ||| |   ||||||  ||||||||||||||  |||| |||||||||| |||
Sbjct  181856  ACACCGCAACCCTCCACACCTATGGAGCAGGTCAGCAGGATGGCAGGGTGGGCAGAGTCC  181797

Query  15901   AGACTGGGGCTTGGGGGGTCTCGGGAGGTCTGTGTGACCTGGGTAGGCTTGTCCAT-CCT  15959
               || |||||||||||| |||||| || ||||||||||| ||||| |  || | || | |||
Sbjct  181796  AGGCTGGGGCTTGGGAGGTCTCAGG-GGTCTGTGTGA-CTGGGCAATCTGGCCCCTCCCT  181739

Query  15960   CATCTGTGGAGGGAGAATT  15978
               || || ||||||||| |||
Sbjct  181738  CACCTATGGAGGGAGGATT  181720
```

Range 19: 181619 to 181664

| Score | Expect | Identities | Gaps | Strand | Frame |
|---|---|---|---|---|---|
| 44.6 bits(48) | 3e-04() | 38/46(83%) | 1/46(2%) | Plus/Minus | |

```
Query  16052   TGTTTTGATGGAAAAATTGATCATGTTCTTTAAGG-CTGCTCTCCC  16096
               ||||||  ||| |||||||||||  |||||||||| |  |||||||
Sbjct  181664  TGTTTTAGTGGGAAAATTGATCACCTTCTTTAAGGCCCCCTCTCCC  181619
```

Range 20: 179839 to 181396

| Score | Expect | Identities | Gaps | Strand | Frame |
|---|---|---|---|---|---|
| 1038 bits(1150) | 0.0() | 1189/1577(75%) | 81/1577(5%) | Plus/Minus | |

```
Query  16099   ACCAGGAGCCAAAGGTCTGGCGTCCCCTGTGAGCAGAGCCCTGACGGAGGCTCCGCTCCC  16158
               |||||||| ||||  ||||| |||| | |||||||||||||||| ||||||||| |||||
Sbjct  181396  ACCAGGAGTCAAAAATCTGGAGTCCTCCGTGAGCAGAGCCCTGATGGAGGCTCCCCTCCC  181337

Query  16159   CAGCGC-CCCTTCTCACCCCGGGGCCTTGTTGCAGGTGGGACGCTGTTCTGCAGGACCGT  16217
               ||||   |||| |||||||| || |||  ||| |||||||  |||| | || ||||||||
Sbjct  181336  CAGCCATCCCTCCTCACCCCAGGACCTCCTTGAAGGTGGGCAGCTGCTTTGTAGGACCGT  181277

Query  16218   GTGGTCTGCACACTCAGGACCCACGCGGATGGCCACGGCTGAGGCCCGCTGCACAGCCCC  16277
               | |||| |   |||| || |||| ||||||||| | | ||||||| | ||||||  | ||
Sbjct  181276  GAGGTCGGTGTACTCTGGGCCCATGCGGATGGC-ATGACTGAGGCTCACTGCACCTCTCC  181218

Query  16278   TGAGGAGCTTCTGGGCTGCTCCAGCTTCTCCAGGAGCGGGAGGCGGCGGGGCGAGCGCAT  16337
```

```
                ||||||||| ||||||||||||| ||||| |||| || ||||||   ||||| || ||||
Sbjct  181217  -GAGGAGCTGCTGGGCTGCTCCAACTTCTGCAGGGGCAGGAGGCCATGGGGCAAGTGCAT  181159

Query  16338   TGAGGTGACCTGCAGGCCCCCGTCGGAGCCTGAAGTGGGGTTCTCGCTTCCAGGTCCAGA  16397
                |||||||||||    | |||   ||||| ||| | |||||||  ||||| ||| |||||
Sbjct  181158  CGAGGTGACCTGTGCCCTCCCACAGGAGCATGAGG-GGGGTTCGTGCTTC-AGGCCCAGA  181101

Query  16398   TCCGCCTGAGCCCCTTCCTCTGCTGAGCTCCAGGCGCCCGCCTGCAAGTTAAAGCAGGAT  16457
               || ||| |  ||||||||||  |||| ||||||   |||  |||||||||||||    ||
Sbjct  181100  TCTGCCCGGACCCCTTCCTCCTCTGAACTCCAGTACCCCTTCTGCAAGTTAAAGTTAAAT  181041

Query  16458   GGGGCACGTCTCAGTCACATGGCT-GGGTGCTGCTGCAGGGAGCCACACTGAGGTTTCCC  16516
               ||||||  | |||||| | | | | ||||||| ||  | |||||||| ||||||  |||
Sbjct  181040  GGGGCATATTTCAGTCCCTTAGTTTGGGTGCT-CTAGAAGGAGCCACGCTGAGGGGTCCT  180982

Query  16517   AGGAGACT--GCAGGACGGTGGCTAGATGGATTCCAGCGACCGACC-----GTCTGGGG-  16568
                |||||||  | |||| |  |||| |||||||  ||| | | | |      |  |||||
Sbjct  180981  GGGAGACTCTGTAGGATGA-GGCTGGATGGATCGCAGTGCCTGCCTCAAGGGGGTGGGGC  180923

Query  16569   -AGCGGG------AGGG---------CT--GGGCATGGG----CCAG-----------GG  16595
                |||| |      ||||         ||  ||| | |||    ||||           ||
Sbjct  180922  CAGCGTGTGTGCCAGGGACTCACTGCCTTGGGGGAGGGGAGCACCAGATGTGTGCAGGGG  180863

Query  16596   ACTCGCTGCCTCTGGACTCACTGGTCCCCAGGGCTCTTT-CACTCAGATGTTACATAGTT  16654
               | |||||||||||| || || ||||| |||| || |||| | |||||| | | ||| |||
Sbjct  180862  ATTCGCTGCCTCTGCACGCAGTGGTCTCCAGAGCCCTTTTCTCTCAGACGGTCCATGGTT  180803

Query  16655   CCAGCAGCTGAGAAATCTTCTCAAACCAGCAGCAGAGGGGACTTGATATTAAGGCCACAG  16714
               |  |||| ||||||||||   |||| |||||  ||||||| | |||  |||||| || ||
Sbjct  180802  CTGGCAGATGAGAAATCT---CAAATCAGCAAAAGAGGGGGCCTGAGGTTAAGGTCAGAG  180746

Query  16715   AGCCTTACAGAGATGCCAACTGGCCAGGGCGTTTTGGTGGAAGGACAGTGCCTCGGCCAG  16774
               |||||  || |||| | | |||| || ||| ||||||||||||| |||  | | ||||||
Sbjct  180745  AGCCTGGCACAGATACTATCTGGTCATGGCCTTTTGGTGGAAGGGCAGCACTTAGGCCAG  180686

Query  16775   GAGGAC-GGGGTGGGCAGGCATTTCTGCCTGGGAGACGGTGTCTGGGAGTGTGTGTGACC  16833
               |||||| ||||||||||  ||||||||||  |||||| |||||  | ||||||  ||||
Sbjct  180685  GAGGACAGGGGTGGGCAACCATTTCTGCCCAGGAGACAGTGTCCAGCAGTGTGCTTGACT  180626

Query  16834   ATGCACTTGATCCTGCAAGTGAGAGTATGTGGGCGGCGTGGCCGAGAGCAGGTCAGGGCT  16893
                 || ||||| |||||| || || | ||||||| |    |||||||||  || | |||||
Sbjct  180625  GAGCCCTTGAACCTGCAGGTTAGGGGATGTGGGAGA---GGCCGAGAGTGGGGCGGGGCT  180569

Query  16894   GAGGAGGCGGGGGCCTTGCTC-GGGGTCTTAGGTTTCCCTGTATCTGCATTTTATGGTCA  16952
               |||  | | ||| ||| |||| |||||||  | |||||||||||||||||| |   ||||
Sbjct  180568  GAGCCGTCTGGGTCCTGGCTCTGGGGTCTATGATTTCCCTGTATCTGCATTCTGAAGTCA  180509

Query  16953   TGCTTAGAGCCCAGAAGAACTTTATTACA--CACAGCTGCCCATGTGCTGAGCAGTTTG-  17009
                 ||  | | |||||||||||| || | |  ||||||||||||| ||| || |||| ||
Sbjct  180508  CTCTCGGTGTCCAGAAGAACTTGATAATATTCACAGCTGCCCATCTGCAGAACAGTCTGT  180449

Query  17010   ------CAGGAGGGAGGTCCTGGTCCTCAGAGG--GGCAGGCTCCTGGCAGGGACGGTGG  17061
                     ||| || ||| |||| ||| |||| ||  ||| ||| ||| || |||||  |||
Sbjct  180448  CTTGGACAGCAGTGAGTTCCTTGTCTTCAGGGGCTGGCTGGCCCCTTGCTGGGACATTGG  180389

Query  17062   AGATGGTATGAGGGACTGGGACCAGCTGCTTGAGCCTGTCCCTTTCAGCCCCCTCATTCT  17121
               ||| |||  ||||| |||||||||| ||   |||   ||||| | |  ||||||||| ||
Sbjct  180388  AGAGGGTTCGAGGGGCTGGGACCAGGTGTCCGAGTTGGTCCCCTCCCACCCCCTCATCCT  180329

Query  17122   GTGTTTCAAAGCCCTTTCTAAAGCATGTTTCTGTTTCTGTCTTTGGCTTTCAGGCCCGAG  17181
               |||||| ||||||||||||||| || |     |||||||| ||||| | |||||||||||
Sbjct  180328  GTGTTTTAAAGCCCTTTCTAAAACAGG-----GTTTCTGTTTTTGGTTCTCAGGCCCGAG  180274

Query  17182   GGGGCAGGCATGTCTGCCTGGCCCACAATGCGTTTGGGGGTGAGGGTGTCTATGCCGTTG  17241
                || ||| ||  | |||||||||| ||| | ||||||||||||||||||||| || ||||
Sbjct  180273  AGGACAGTCACATGTGCCTGGCCCCCAAAGTGTTTGGGGGTGAGGGTGTCTACGCTGTTG  180214

Query  17242   CCAGATGCTGCCTGCTGCCCCAGGCCAACTGCAGTGTCCACACAGCTCCGCCAGCCAGGG  17301
               |||||||||||||||  |||| ||||||||||||  |||| ||||||  ||||||  ||
Sbjct  180213  CCAGATGCTGCCTGCCTCCCCGGGCCAACTGCAGCATCCATACAGCTTTGCCAGCTGGGC  180154
```

```
Query  17302   CTGGTGTGCTGACCCAAGCCCACTGCCACCAGCAGGGCCACGTCCTCACAGGTAGGAGGC  17361
               ||||||||| |||||| |||   |||| |||||||||| |||| ||||||||||||||||
Sbjct  180153  CTGGTGTGCAGACCCATGCCTGATGCCCCCAGCAGGGCTACGTGCTCACAGGTAGGAGGC  180094

Query  17362   TGGGCCCATCCTGGGGTGAAGAGGCTTCCTTGTCTCCTGGTGCACCTGCTCCCACCTGAC  17421
               ||||| || |||||||||| ||||| |||||| ||||  ||||||| |||  ||  || |
Sbjct  180093  TGGGCTCACCCTGGGGTGAGGAGGCATCCTTGGCTCCCTGTGCACCAGCTTACAGATGCC  180034

Query  17422   TGGTCCCATGCTGGGGCCCAACTGCCTGGTGCGAAGGCCTGTGCTA-CCCTTCCATCCCT  17480
               |||||||| ||||| |  |||||| |||||| ||||||| |||||| |||||||||||||
Sbjct  180033  TGGTCCCACGCTGGAGTGCAACTGTCTGGTGGGAAGGCCAGTGCTACCCCTTCCATCCCT  179974

Query  17481   GTGACCCTGGGTGGGCACCTCATTGGTCTCAGTCTCAGCTTCTTCCTCCCTAAGAAGAAT  17540
               |   ||||| |  | ||||||||  ||||||||||||||||| |||||||||||| |
Sbjct  179973  GGAGCCCTGAGCAGACACCTCATCTGTCTCAGTCTCAGCTTCCTCCTCCCTAAGAGGGGC  179914

Query  17541   GACGGTAGTTCCTGCCTCAATGGGTTGCCATGG---AATGAGTAAGCCCTAGAGCACCAG  17597
                  ||||| |||||| ||| || |||||  |||   |||| ||||  ||||||||||||
Sbjct  179913  CGTGGTAGCTCCTGCTTCACTGCGTTGC--TGGGAAAATGTGTAACACCTAGAGCACCAG  179856

Query  17598   GCCTGGAGCATC-CAGG  17613
               |||  |||| || ||||
Sbjct  179855  GCCCTGAGCTTCGCAGG  179839
```

Range 21: 178403 to 178573

| Score | Expect | Identities | Gaps | Strand | Frame |
|---|---|---|---|---|---|
| 122 bits(134) | 1e-27() | 135/178(76%) | 8/178(4%) | Plus/Minus | |

```
Query  17648   CTCAGGCCAGTGTCTCGTTCCTGCCCTGACTTATTTCTGGGTTTCCCAGCTCCAGCCCCA  17707
               |||||  ||||| | | ||||||||||  ||     |||| |||||||||| |||   ||
Sbjct  178573  CTCAGATCAGTGCCGCTTTCCTGCCCTTTCT-----CTGGATTTCCCAGCTTCAGGATCA  178519

Query  17708   GACCCGAAAGAGATGGAGTCTGAATGGGGTGGG-GAGGACAGACAGATGGTCCCACAGCA  17766
               ||||| ||| |||| ||  ||||   ||  ||  ||||| |||||||  |||||  ||||
Sbjct  178518  GACCCTAAAAAGATAGA--CTGATAAGGACGGAAGAGGAGAGACAGAAAGTCCCTGAGCA  178461

Query  17767   TCCAGGTGTCTGAGCTGGCCCTCCTTTGCCCCAGGCTGCAGCTCCCACTGGGAAGTGG  17824
               |||  |||||||||| || |||||  ||||||||||||| ||||||||||||  ||||
Sbjct  178460  TCCGAGTGTCTGAGCCGGTCCTCCCCTGCCCCAGGCTGCTGCTCCCACTGGGCGGTGG  178403
```

Range 22: 177259 to 178365

| Score | Expect | Identities | Gaps | Strand | Frame |
|---|---|---|---|---|---|
| 646 bits(716) | 0.0() | 839/1151(73%) | 109/1151(9%) | Plus/Minus | |

```
Query  19200   GCCCGGGTGCTGGGGGGTCCGTCAGGGCGGGAGTGTAGAGGATGCTGGAATCTGAAGGAG  19259
               ||||||||| |||||  |  | || |  ||||||||  ||||| ||||   ||||  |||
Sbjct  178365  GCCCGGGTGGTGGGGCTTA-GCCACGGAGGGAGTGTGCAGGATACTGGGCCCTGAGAGAG  178307

Query  19260   GGGCTGCACA--TCTGATGGCCTGGATATTGGGGGAGCAGTGGAGGGGGCG-TCCAAGGG  19316
               | ||  ||||  ||||||||| |||| | | | ||| ||||||||||||| | | | | ||
Sbjct  178306  GAGCC-CACAGCTCTGATGGACTGGCTGTGGCGGGGGCAGTGGAGGGGGGGCTGCCAAGG  178248

Query  19317   TTTTGCTTTGCTCTCGGACGAATGGCATCGCCCCTGACTGGGATGGGAAGGGCTGTGAGA  19376
               ||||  || ||| |               ||||||||  |||||||  || |||| | ||
Sbjct  178247  TTTT--TTCGCTTT---------------GCCCCTGATGGGGATGGTGAGAGCTGCGGGA  178205

Query  19377   GGTCAAGTGTC-GGGGAAGTTGAGGCATTTATGCGGGCCTG---------GCT----CAC  19422
                | |  ||||| |||||||  ||||   |  ||  ||||||         |||    | |
Sbjct  178204  AGCCTGGTGTCTGGGGAAGAAGAGGAGCTCGTGTTGGCCTGCAGACATTCGCTTGTGCCC  178145
```

```
Query  19423   AGCGTGCC-----------GTGCCTTACATGTGC--TTTCTTTTGTCCCCGGGCCCTGGC  19469
                ||| |||           ||||||  |||||||  ||||||| ||||  ||||||||||
Sbjct  178144  TGCGGGCCTAGCTTGCAGAGTGCCT--CATGTGCCCTTTCTTTGGTCCTGGGGCCCTGGC  178087

Query  19470   AGGTCACCGTGGCCTGCAAGGAGGGCTGGACGCTGACCGGCTGCGGGGCCCACCCCGGGG  19529
               ||||||||||||||||  || || ||||||| ||||| ||||||||| | ||||  | ||
Sbjct  178086  AGGTCACCGTGGCCTGGGAG-AGCGCTGGACACTGACTGGCTGCGGGACTCACCTGGAGG  178028

Query  19530   CCTCCCACACCCTGGGGGCCTATGCAGTGGACAACACGTGTGTGGTGAGGGGCCGGGACG  19589
               |||||||||||  |||| ||||||  |||||||||||||||||| || ||||| ||||||
Sbjct  178027  CCTCCCACACCACGGGGACCTATGTGGTGGACAACACGTGTGTGATGTGGGGCTGGGACG  177968

Query  19590   TGGGTGTGCGAGGCAGGACGGGTGAGGAGGCCGCCGTGGCCATTGCCATCTGCTGCAGGA  19649
               |  |||   |||||||| | | ||||| |||| | || ||  ||||||||||||||  ||
Sbjct  177967  TCAGTGCAGGAGGCAGGGCTGCTGAGGTGGCCCCTGTAGCTGTTGCCATCTGCTGCCAGA  177908

Query  19650   GCCGG----TCAGGGGAGCAGGCCTCCCCGGGGACCCAGTGACAGCCCCGCCCAGGATAT  19705
               ||| |    |  || |||||||||||   || | ||||||||| |||| |||||||| | 
Sbjct  177907  GCCAGCTGTTGGGGAGAGCAGGCCTCTTGGGAGGCCCAGTGACTGCCCTGCCCAGGACAC  177848

Query  19706   CTGC--------GTGG-------CTGG-------GGTCCCAGGCCTTGGCTGAGCTTTGA  19743
                |||        | ||       ||||       ||||||||||||||||||||||||||
Sbjct  177847  TTGCACAAGGAAGGGGTCATGTCCTGGATGCCGTGGTCCCAGGCCTTGGCTGAGCTTTGA  177788

Query  19744   AGTGCTTCCTTTTTCCTCCTTCCTCAGCCCTCCTCAGCCTGGGCCCCGGGGGACAGAAGG  19803
               | || |||||| |||| || || |||||||||  |||||||| ||| |||  | ||| | 
Sbjct  177787  ACTGGTTCCTTCTTCCGCCCTCTTCAGCCCTCTCCAGCCTGGACCCTGGGCAA-AGAGGC  177729

Query  19804   CACCTCTTTCTCCTGGAGCTCTGGTGCTGGCACTTGGGGTACACTGGCTCCCTGCCTGGG  19863
                || |||| |||||||||||||||||||||||||  ||||   | |||||||||| ||||
Sbjct  177728  TACTTCTTCCTCCTGGAGCTCTGGTGCTGGCACTCAGGGTGTGCCGGCTCCCTGCTTGGG  177669

Query  19864   AGAACCCCATCTCTTGGCCCGAGTCACCCCTCCCCAGACCCGAGCTGAGTGGGAGGTTGA  19923
               |||||| || ||||||||| |  ||||||| ||||||||| ||||||| | ||||| |||
Sbjct  177668  AGAACCTCAGCTCTTGGCCTGCATCACCCCACCCCAGACCTGAGCTGAAT-GGAGGCTGA  177610

Query  19924   ATGAGCAGGGCCACAGGCGCC-GGCAGCCCCTCCCTCACTGAGGGGCTGTGTCCACATG-  19981
                 ||| |||||  |||||||| ||||| |||||| ||||||  |||  | ||||||||| 
Sbjct  177609  TGGAGTAGGGCTGCAGGCGCCAGGCAGGCCCTCCATCACTGCAGGGTGGGGTCCACATGC  177550

Query  19982   -TCCATCAACAAGGGTCTGGCTGTGCTCAGCTCCCTGTCAGCTGCTCCCAAGTTGCCAGT  20040
                 |||| | ||  || | |||||||||||||| |||| ||||  ||||| || |||||  
Sbjct  177549  CACCATTAGCACAGGCCAGGCTGTGCTCAGCTTCCTGCCAGCCACTCCCCAGATGCCAAC  177490

Query  20041   GCTGTGGGCAGAATTAGCTTTTGTTGAGTTCTTGCTACATGTCAGCCAGGCAGTCAGTCC  20100
                 |||| ||||||| | ||||| | ||| | ||                  ||||| | |
Sbjct  177489  ATTGTGAGCAGAATGACCTTTTATCGAGCTGTT------------------AGTCAATGC  177448

Query  20101   TCAGGCCTCCATGAAGGAGGTGGTAACCCTCCTATGGGGAGGCAAGGAAGCACTTGACGG  20160
               |||||||||||||||||||||||| | |||||| ||||||||||  || || ||||| ||
Sbjct  177447  TCAGGCCTCCATGAAGGAGGTGGTCATCCTCCTGTGGGGAGGCAGTGAGGCGCTTGAAGG  177388

Query  20161   CTGGGAGAGGCCAAATGTTGGTCA-GAGGATGTGAAAGGTGGAAATGGCCCCTCACCTCC  20219
               |||| ||||||||||| |  |||| | ||| ||||||||| || |||||||||||| || 
Sbjct  177387  CTGGAAGAGGCCAAATATCAGTCAGGGGGACGTGAAAGGTAGAGATGGCCCCTCACTTCT  177328

Query  20220   TGCCCACTCTGGGGAGGCCC---GGTTGGGCTCCCTGATTA-TGGA-GATGAGTTTTCCA  20274
                |||| || |||||||||||   |    |||| |||||||| |||| |||  ||||||| 
Sbjct  177327  GGCCCGCTTTGGGGAGGCCCCATGAGGAGGCTTCCTGATTAGTGGAGGAT--GTTTTCCC  177270

Query  20275   TGCCTCTGGGG  20285
               |||||||| ||
Sbjct  177269  TGCCTCTGAGG  177259
```

## Taxonomy

**Reports**

- **Lineage**
- **Organism**
- **Taxonomy**

## Dot Plot

**Plot of lcl|Query_48003 vs lcl|Query_48005**

Top
