# Supplementary material for: Molecular evolution of PCSK family: Analysis of natural selection rate and gene loss
Source: PLoS One. 2021 Oct 28;16(10):e0259085. doi: 10.1371/journal.pone.0259085 (PMC8553125; doi:10.1371/journal.pone.0259085)
Supplement: S3 File — Regions indicating changes in coding sequence or frame are highlighted (if applicable). (PDF) [file pone.0259085.s009.pdf]

COVID-19 Information

Public health information (CDC) | Research information (NIH)

SARS-CoV-2 data (NCBI) | Prevention and treatment information (HHS) | Español

BLAST ® » **blastn suite-2sequences** » **results for RID-HAFXV63H114**

**Job Title**	Nucleotide Sequence ...
**RID**	HAFXV63H114 Search expires on 08-13 23:35 pm
**Program**	Blast 2 sequences
**Query ID**	lcl|Query_60829 (dna)
**Query Descr**	None ...
**Query Length**	20287
**Subject ID**	lcl|Query_60831 (dna)
**Subject Descr**	ref|NC_018730.3|:c44842962-44825408_Felis_catus_isolate_Cinnamon_breed_Abyssinian_chromosome_C1_Felis_catus_9.0_whole_genome_shotgun_sequence ...
**Subject Length**	17555

Descriptions

Description	Scientific Name	Max Score	Total Score	Query Cover	E value	Per. Ident	Acc. Len	Accession
ref|NC_018730.3|:c44842962-44825408_Felis_catus_isolate_Cinnamon_breed_Abyssinian_chromosome_C1_Felis_catus_9.0_whole_genome_shotgun_sequence		516	802	9%	3e-147	68.99%	17555	Query_60831

Graphic Summary

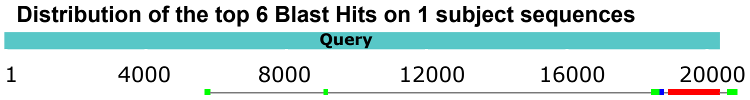

Alignments

Alignment view Pairwise ☐ CDS feature Restore defaults

ref|NC_018730.3|:c44842962-44825408_Felis_catus_isolate_Cinnamon_breed_Abyssinian_chromosome_C1_Felis_catus_9.0_whole_genome_shotgun_sequence
Sequence ID: Query_60831 Length: 17555 Number of Matches: 6
Range 1: 13624 to 13684

Score	Expect	Identities	Gaps	Strand	Frame
52.7 bits(57)	9e-08()	48/61(79%)	0/61(0%)	Plus/Plus	

```
Query  5697   GACACTGAAACCCAGAGTGGGACAGGGTCTTGCCTGAGGTCACACAGCATAGAACTGGCA  5756
              ||||||||  | ||||| ||||| | | |||| |  | |||||||||||  |||||||||
Sbjct  13624  GACACTGAGGCTCAGAGAGGGACGGCGACTTGTCCAAAGTCACACAGCAAGGAACTGGCA  13683
```

```
Query  5757   G  5757
              |
Sbjct  13684  G  13684
```

Range 2: 13628 to 13671

Score	Expect	Identities	Gaps	Strand	Frame
53.6 bits(58)	3e-08()	38/44(86%)	0/44(0%)	Plus/Minus	

```
Query  8978   GCTGTGTGACCTTGGATAAGTCACTGACCGTCTCTGAGCCTCAG  9021
              |||||||||| ||||| ||||| | | || ||||||||||||||
Sbjct  13671  GCTGTGTGACTTTGGACAAGTCGCCGTCCCTCTCTGAGCCTCAG  13628
```

Range 3: 5554 to 5716

Score	Expect	Identities	Gaps	Strand	Frame
59.9 bits(65)	6e-10()	117/167(70%)	6/167(3%)	Plus/Minus	

```
Query  18180  TGTGGCTGGGCCAGGTTTTGCTTTTGTCTAGTTTAGCGAGGTTTGCTCTCTGGGGCACCC  18239
              ||| |||||||||| || ||||||||||    |||| |||  ||||  |||||  |||
Sbjct  5716   TGTAGCTGGGCCAGTTTGTGCTTTTGTCCCTATTAGGGAGCCTTGCAATCTGGA-CACGT  5658

Query  18240  TGCCCCTCCCTTGCAGAGAATATG-ACAAATGTTGCATAAGGAAGATCAGCCCACATGC-  18297
               || || ||  |||||||||||||   || || ||| |||  ||   |||  | | |||
Sbjct  5657   GGCTCCCCC--TGCAGAGAATATGCGTAAGTGCTGCCTAACAAAATGCAGTACTCCTGCG  5600

Query  18298  ATTCACTGGTTCATCCACTCAGCACATCTGCTGGGAGGATGACTCAG  18344
               |||  || || ||||| | | ||  |||||||||| | | ||||||
Sbjct  5599   TTTCCTTGATTAATCCATTTAACAAGTCTGCTGGGACGCT-ACTCAG  5554
```

Range 4: 5482 to 5521

Score	Expect	Identities	Gaps	Strand	Frame
41.9 bits(45)	2e-04()	33/40(83%)	0/40(0%)	Plus/Minus	

```
Query  18335  GATGACTCAGCCGTGACCAAGAGGAGGGGACACCTGAGCT  18374
              || ||||| || ||||||  ||||||||||||  ||||||
Sbjct  5521   GAGGACTCTGCTGTGACCCGGAGGAGGGGACATATGAGCT  5482
```

Range 5: 3870 to 5284

Score	Expect	Identities	Gaps	Strand	Frame
516 bits(571)	3e-147()	1019/1477(69%)	157/1477(10%)	Plus/Minus	

```
Query  18486  CCTTTTAAAAGCCACAGGGAACTTCTTCAAAGGAAGCCCTGCAGAGTTCACTTTTAAA-T  18544
              ||||| ||||||||| |||| |||||| |  ||||||||||  ||||   || ||||| |
Sbjct  5284   CCTTTAAAAAGCCACCGGGAGCTTCTTAATGGGAAGCCCTGGGGAGTCTGCTCTTAAAAT  5225

Query  18545  GAACTG-GAAGAGGTTTTTAAGAGTGTGAGTCTGTGCTGATTGTGT--TCTGCATGCTGC  18601
              | |||  ||||||| |  ||| ||    || ||   |||| ||||   |||| |||  ||
Sbjct  5224   GCACTCCGAAGAGGCTAATAAAAGCACAAGACT---CTGAGTGTGAGGTCTGGATGAAGC  5168

Query  18602  ATTTCTGGAGGGCAAGGGCTGTTCCAGGTCCACTTGCTCAGCAAATGTTGAG----GCCT  18657
              |||||||||||||| || ||||  |||||||| |||||||||   | |||||    | ||
Sbjct  5167   ATTTCTGGAGGGCAGGGACTGTCTCAGGTCCA-TTGCTCAGCCTGTATTGAGCAAGGACT  5109

Query  18658  GTGGCATCCCAGGCAATGTTCCAGGCGGTGGGGATACAAACCCGACTAGCT-TTCTCTCC  18716
              |  || | ||  ||| ||| || ||   |||| || || |||| ||||||  |||| |||
Sbjct  5108   GCAGCGTTCCCAGCACTGT-CCGGGACCTGGGCATGCAGACCCAACTAGCCCTTCTTTCC  5050

Query  18717  TGGCGCGTCCAGTCTAATGGGGGAGAAGGACAGCAAACAAATAAGTAACTATAGAGTAAT  18776
              ||| | ||||| |    |||||||||||| | | ||||||| | ||||| | |||| | |
Sbjct  5049   TGGGGTGTCCATTTCCGTGGGGGAGAAGGGCGGTAAACAAAAACGTAAC-ACAGAGCACT  4991

Query  18777  TAAAACATGCTATAGAGGAAAGTAAAGCAGGGAAGGGAATG-------GGAGGGTCCTTC  18829
              |||||  |||||| ||||| |   |||||||| ||||||||       || | ||  ||
Sbjct  4990   TAAAAGGTGCTATGGAGGAGAAGCAAGCAGGGGAGGGAATGTCGGGTGGGGGCGTTGTTT  4931
```

```
Query  18830  AGGAGAGGCCTCCTTGAGAAGGTGGGGACATCACAGGGAACAGTGTTCAAGGCAGAGGG  18889
               |||||||||||  | ||||||||| ||||||   ||||||||| |||| |  ||| |||
Sbjct  4930   CGGAGAGGCCTCACTCAGAAGGTGGAGGACATTGTAGGGAACAGCGTTCCAC-CAGTGGG  4872

Query  18890  GGTAGCCAGGGCAAAGGCCCTGAGGTGGG-AGTGGGCTTGGAGAGCAAAAGGAAGAGCCA  18948
                 ||||||||  ||| | || |   ||| ||   || ||| | |||  ||||  || ||
Sbjct  4871   AACAGCCAGGGTGAAGACTCTCACACGGGGAGCCTGCCTGGGGGGCAGCAGGAGCAGACA  4812

Query  18949  GAGGGCTGGTGAGGTGGGACCCGAGTGGG--AGGGGGAACCAGAGA-----CAGG----G  18997
              |  |||  ||| | ||||| || |||| |  ||||||| |||||||     ||||    |
Sbjct  4811   GGAGGCCCGTGGGATGGGAACCCAGTGAGTCAGGGGGA-CCAGAGAGGGGACAGGACAAG  4753

Query  18998  TTTAGGTGGGGCCGGAGGGCCACAGGAAGGACTT-GGATTTTTACTGGAGTGAGCTGGGA  19056
               | || ||||||| ||||||||||| |||||||| |||||||  |||     ||||||||
Sbjct  4752   GTCAGATGGGGCCTGAGGGCCACAGTAAGGACTTTGGATTTTGCCTG-----AGCTGGGA  4698

Query  19057  GCCACACAGGGTTCTGAGCCTGGG-TGTGGGG------AGGGGGGTGGGCTATCTGACCT  19109
              ||| | |||||||||||||| ||| ||||||       ||||   |||| | |||||||
Sbjct  4697   GCCCCTCAGGGTTCTGAGCCCGGGGTGTGGGTGTGCTCAGGGAAATGGGATGTCTGACCC  4638

Query  19110  GGGTGTGAGCAGGTTCATTCTGGTCGCTGTGTCGGGAAGACTGCAGGGGACAGGGCGGAA  19169
              ||||  |  |||||||||||||| ||| |     |||||||| | ||||||||||| | |
Sbjct  4637   GGGTTGGCACAGGTTCATTCTGGCCGCGG-----GGAAGACTTCGGGGACAGGGCAGGA  4583

Query  19170  GCAGGGAGGCCCGCTGTAGACGGGTGGA-----------------CAGCCCGGGTGCTGG  19212
              |||||||| || ||||||||| || |||                 ||| ||| |   ||
Sbjct  4582   GCAGGGAGACCAGCTGTAGACAGGCGGACACCCACTGTGACTGTCCAGACCGAGGAGTGA  4523

Query  19213  GGGGTCCGT-CAGGGCGGGAGTGTAGAGGATGCTGGAATCTGAAGGAGG-GGCTGCACAT  19270
              ||  || || |||||  || |||| ||| |  |||||  |||| ||||| | |  || |
Sbjct  4522   GGCCTCTGTACAGGGTCGGGGTGTGGAGCACACTGGAGCCTGAGGGAGGAGCCCTCAGAC  4463

Query  19271  CTGATGGCCTGGATATTGGGGGAGCAGTGGAGGGGGCGTCCAAGGGTTTTGCTTTGCTCT  19330
                ||||| |||||| |  ||||||       ||  |||     |||          | |
Sbjct  4462   TGGATGGGCTGGATGTGCGGGGAG-------GGCTGCG-----GGG---------ACCCC  4424

Query  19331  CGGACGAATGGCATCGCCCCTGACTGGGA-TGGGAAGGGCTGTGAGAGGTCAAGTGTCGG  19389
               || || ||||| | ||  ||||||||||  ||||||| | | | ||||   || |  ||
Sbjct  4423   TGGGCGGATGGCGTTGCTGCTGACTGGGAGAGGGAAGGCCGGAG-GAGGCATAGGGCTGG  4365

Query  19390  GGAAG-----TTG----AGGCAT------------TTATGCGGGCCTGGCTCACAGCGTG  19428
              ||| |     |||     ||| |             | ||| ||||| | ||   | |||
Sbjct  4364   GGACGCGAGCTTGCTACTGGCGTGATGGACCCGTGCTCTGCAGGCCTAGTTCCTCGTGTG  4305

Query  19429  CCG-TGCCTTACATGTGCTTTCTTTTGTCCCCGGGCCCTGGCAGGTCACCGTGGCCTGCA  19487
              | | |||   ||| | | || |||||||||| | |||||||||||||||||||||||||
Sbjct  4304   CTGCTGCTGGACACGCGTTTGCTTTTGTCCCTGAGCCCTGGCAGGTCACCGTGGCCTGCG  4245

Query  19488  AGGAGGGCTGGACGCTGACCGGCTGCGGGG------CCCACCCCGGGGCCTCCCACACCC  19541
              |||  ||||||| |||||| ||||| || |      |||||||| |||||||||||| ||
Sbjct  4244   AGGCAGGCTGGATGCTGACTGGCTGTGGCGTCCTCCCCCACCCC-GGGCCTCCCACATCC  4186

Query  19542  TGGGGGCCTATGCAGTGGACAACACGTGTGTGGTGAGGGGCCGGGACGTGGGTGTGCGAG  19601
              ||||| |||| | || |||||||||  ||||||||||| |||  || || ||||   |||
Sbjct  4185   TGGGGACCTAGGAAGGGGACAACACCCGTGTGGTGAGGAGCCAAGA-GTTGGTGCAGGAG  4127

Query  19602  GCAGGACGGGTGAGGAGGCCGCCGTGGCCATTGC-CATCTGCTGCAGGAGCCGGTCAGGG  19660
              ||  | | |||||||||||| |  |||||   ||   |||  ||| || ||       ||
Sbjct  4126   GC--GGCCGGTGAGGAGGCCAC--TGGCCGAAGCTGGTCT-TTGC-GGGGC-------GG  4080

Query  19661  GAGCAGGCCTCCCCGGGGACCCAGTGACAGCCCCGCCCAGGATATCTGCGTGGCTGGGGT  19720
              | |||||| |||| |||| ||||||| |  |||| ||||||    ||  |  || | |||
Sbjct  4079   GGGCAGGCGTCCCAGGGGTCCCAGTG-CCTCCCCACCCAGG---CCT--GGAGCCGTGGT  4026

Query  19721  CCCAGGCCTTGGCTGAGCTTTGAAGTGCTTCCTTTTT---CCTCCTTCCTCAGCCC----  19773
              |||||||||||||||||||||||  || |||||  ||   ||| ||||| || |||
Sbjct  4025   CCCAGGCCTTGGCTGAGCTTTGAGATGGTTCCTCCTTCCCCCTTCTTCCCCACCCCCCCA  3966

Query  19774  TCCTCAGCCTGGGCCCCGGGGGACAGAAGGCACCTCTTTC-TCCTGGAGCTCTGGTGCTG  19832
               ||||||||||||||||  ||| |||  | ||| |||  | |||||||||| |||  |||
Sbjct  3965   CCCTCAGCCTGGGCCCCAAGGGGCAGGGGACACTTCTAGCTTCCTGGAGCTGTGGCACTG  3906

Query  19833  GCACT--TGGGGTACACTGGCTCCCTGCCTGGGAGAA  19867
              |||||   |||| ||   |||||||  || |||||||
Sbjct  3905   GCACTCAGGGGGAACGTGGGCTCCC-ACCCGGGAGAA  3870
```

Range 6: 3566 to 3729

Score	Expect	Identities	Gaps	Strand	Frame

77.9 bits(85) 2e-15() 128/183(70%) 19/183(10%) Plus/Minus

```
Query  20103  AGGCCTCCATGAAGGAGGTGGTAACCCTCCTATGGGGAGGCAAGGAAGCACTTGACGGCT  20162
              ||| |||| ||||||||||| | | ||||| | |||  ||| |||  |||||||| ||||
Sbjct  3729   AGGGCTCCGTGAAGGAGGTGATCATCCTCCCACGGGTGGGC-AGGGGGCACTTGAGGGCT  3671

Query  20163  GGGAGAGGCCAAATGTTGGTCAGAGGATGTGAAAGGTGGAAATGGCCCCTCACCTCCTGC  20222
              || ||            || ||| |  |||||||   ||| ||||| |||||   |||||
Sbjct  3670   GGAAG------------GGGCAGTGAGTGTGAAACAGGGAGATGGCTCCTCA---CCTGC  3626

Query  20223  CCACTCTGGGGAGGCCCGGTTGGGCTCCCTGATTATGGAGATGAGTTTTCCATGCCTCTG  20282
              |  || ||| || |||||  |||||||||||||||    ||| ||||| ||   ||| ||
Sbjct  3625   CTGCTTTGGAGAAGCCCG--TGGGCTCCCTGATTA-ACCGATCAGTTTCCCGCACCTATG  3569

Query  20283  GGG  20285
              |||
Sbjct  3568   GGG  3566
```

Taxonomy

Reports

- **Lineage**
- **Organism**
- **Taxonomy**

Dot Plot

Plot of lcl|Query_60829 vs lcl|Query_60831

Top
