# Supplementary material for: Molecular evolution of PCSK family: Analysis of natural selection rate and gene loss
Source: PLoS One. 2021 Oct 28;16(10):e0259085. doi: 10.1371/journal.pone.0259085 (PMC8553125; doi:10.1371/journal.pone.0259085)
Supplement: S4 File — Regions indicating changes in coding sequence or frame are highlighted (if applicable). (PDF) [file pone.0259085.s010.pdf]

COVID-19 Information

Public health information (CDC) | Research information (NIH)

SARS-CoV-2 data (NCBI) | Prevention and treatment information (HHS) | Español

BLAST ® » **blastn suite-2sequences** » results for RID-HAEGFE8F114

**Job Title**	Nucleotide Sequence ...
**RID**	HAEGFE8F114 Search expires on 08-13 23:11 pm
**Program**	Blast 2 sequences
**Query ID**	lcl|Query_47935 (dna)
**Query Descr**	None ...
**Query Length**	20287
**Subject ID**	lcl|Query_47937 (dna)
**Subject Descr**	ref|NW_006804007.1|:4227697-4251255_Erinaceus_europaeus_isolate_Erinaceus_europaeus_13Jul2011_unplaced_genomic_scaffold_EriEur2.0_scaffold00084_whole_genome_shotgun_sequence ...
**Subject Length**	23559

Descriptions

Description	Scientific Name	Max Score	Total Score	Query Cover	E value	Per. Ident	Acc. Len	Ac
ref|NW_006804007.1|:4227697-4251255_Erinaceus_europaeus_isolate_Erinaceus_europaeus_13Jul2011_unplaced_genomic_scaffold_EriEur2.0_scaffold00084_whole_genome_shotgun_sequence		36.5	36.5	0%	0.009	95.45%	23559	Que

»

Graphic Summary

Distribution of the top 1 Blast Hits on 1 subject sequences

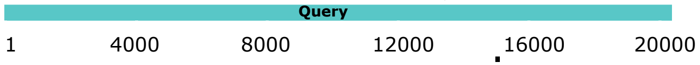

Alignments

Alignment view Pairwise ☐ CDS feature Restore defaults

ref|NW_006804007.1|:4227697-
4251255_Erinaceus_europaeus_isolate_Erinaceus_europaeus_13Jul2011_unplaced_genomic_scaffold_EriEur2.0_scaffold00084_whole_genome_shotgun_sequence
Sequence ID: Query_47937 Length: 23559 Number of Matches: 1
Range 1: 12092 to 12113

Score	Expect	Identities	Gaps	Strand	Frame
36.5 bits(39)	0.009()	21/22(95%)	0/22(0%)	Plus/Plus	

```
Query  14949  CACCCACCCCTCTCCTCTCCAG  14970
              ||||||||||| ||||||||||
Sbjct  12092  CACCCACCCCTGTCCTCTCCAG  12113
```

Taxonomy

Reports

- **Lineage**
- **Organism**
- **Taxonomy**

Dot Plot

Plot of lcl|Query_47935 vs lcl|Query_47937

Top
