# Supplementary material for: Molecular evolution of PCSK family: Analysis of natural selection rate and gene loss
Source: PLoS One. 2021 Oct 28;16(10):e0259085. doi: 10.1371/journal.pone.0259085 (PMC8553125; doi:10.1371/journal.pone.0259085)
Supplement: S5 File — Regions indicating changes in coding sequence or frame are highlighted (if applicable). (PDF) [file pone.0259085.s011.pdf]

COVID-19 Information

Public health information (CDC) | Research information (NIH)

SARS-CoV-2 data (NCBI) | Prevention and treatment information (HHS) | Español

BLAST ® » blastn suite-2sequences » results for RID-J9P041UJ114

Job Title	Nucleotide Sequence ...
RID	J9P041UJ114 Search expires on 08-25 19:29 pm
Program	Blast 2 sequences
Query ID	lcl|Query_34729 (dna)
Query Descr	None ...
Query Length	20287
Subject ID	lcl|Query_34731 (dna)
Subject Descr	ref|NW_023416284.1|:36306287-36597310 Rousettus aegyptiacus isolate mRouAeg1 unplaced genomic scaffold, mRouAeg1.p scaffold_m13_p_1, whole genome shotgun sequence ...
Subject Length	291024

Descriptions

Description	Scientific Name	Max Score	Total Score	Query Cover	E value	Per. Ident	Acc. Len	Accession
ref|NW_023416284.1|:36306287-36597310 Rousettus aegyptiacus isolate mRouAeg1 unplaced genomic scaffold, mRouAeg1.p scaffold_m13_p_1, whole genome shotgun sequence		376	955	6%	3e-104	68.31%	291024	Query_34731

»

Graphic Summary

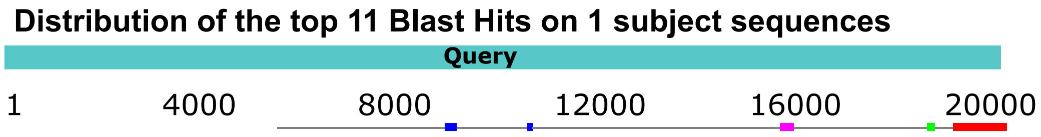

Alignments

Alignment view Pairwise ☐ CDS feature Restore defaults

ref|NW_023416284.1|:36306287-36597310 Rousettus aegyptiacus isolate mRouAeg1 unplaced genomic scaffold, mRouAeg1.p scaffold_m13_p_1, whole genome shotgun sequence

Sequence ID: Query_34731 Length: 291024 Number of Matches: 11

Range 1: 139242 to 139261

Score	Expect	Identities	Gaps	Strand	Frame
37.4 bits(40)	0.034()	20/20(100%)	0/20(0%)	Plus/Minus	

```
Query  5541    AATGCATCTTTaaaaaaaaa  5560
               ||||||||||||||||||||
Sbjct  139261  AATGCATCTTTAAAAAAAAA  139242
```

Range 2: 71704 to 71732

Score	Expect	Identities	Gaps	Strand	Frame
44.6 bits(48)	2e-04()	27/29(93%)	0/29(0%)	Plus/Plus	

```
Query  8978   GCTGTGTGACCTTGGATAAGTCACTGACC  9006
              |||||||||||||||| ||||||||| ||
Sbjct  71704  GCTGTGTGACCTTGGACAAGTCACTGCCC  71732
```

Range 3: 265898 to 265948

Score	Expect	Identities	Gaps	Strand	Frame
43.7 bits(47)	8e-04()	40/51(78%)	0/51(0%)	Plus/Minus	

```
Query  8978    GCTGTGTGACCTTGGATAAGTCACTGACCGTCTCTGAGCCTCAGGTTCCTC  9028
               ||| |||||||||||  ||||| ||   | || ||| ||||||| ||||||
Sbjct  265948  GCTCTGTGACCTTGGGCAAGTCCCTTCTCCTCGCTGGGCCTCAGTTTCCTC  265898
```

Range 4: 265893 to 265948

Score	Expect	Identities	Gaps	Strand	Frame
44.6 bits(48)	2e-04()	44/56(79%)	1/56(1%)	Plus/Minus	

```
Query  10450   GCTGTGTGACCTTGTGCA-GTTACTTACCCTTTCTGTGCCTCAGTTTCCTTGTCTG  10504
               ||| |||||||||| ||| ||  |||  |||  ||| |||||||||||||  ||||
Sbjct  265948  GCTCTGTGACCTTGGGCAAGTCCCTTCTCCTCGCTGGGCCTCAGTTTCCTCATCTG  265893
```

Range 5: 234464 to 234500

Score	Expect	Identities	Gaps	Strand	Frame
41.0 bits(44)	0.003()	31/37(84%)	0/37(0%)	Plus/Minus	

```
Query  10468   GTTACTTACCCTTTCTGTGCCTCAGTTTCCTTGTCTG  10504
               ||| |||| ||| |||| |||||||||||||  ||||
Sbjct  234500  GTTCCTTATCCTCTCTGGGCCTCAGTTTCCTCATCTG  234464
```

Range 6: 60383 to 60417

Score	Expect	Identities	Gaps	Strand	Frame
37.4 bits(40)	0.034()	29/35(83%)	0/35(0%)	Plus/Plus	

```
Query  10476  CCCTTTCTGTGCCTCAGTTTCCTTGTCTGGGCAAT  10510
              |||| |||| |||||||||||||  ||||  ||||
Sbjct  60383  CCCTCTCTGAGCCTCAGTTTCCTCATCTGTACAAT  60417
```

Range 7: 81809 to 82013

Score	Expect	Identities	Gaps	Strand	Frame
184 bits(203)	4e-46()	171/219(78%)	15/219(6%)	Plus/Plus	

```
Query  15538  CAGGTCTTTTAACTTCTG-AGGAACAGCCTGgtgtgtctctgtgcatgtgtgtgtgtgtg  15596
              |||||  ||||||||||| | ||| ||| | ||||||   |||||||||||||||||| |
Sbjct  81809  CAGGTGGTTTAACTTCTGGAAGAATAGCTTAGTGTGTGCATGTGCATGTGTGTGTGTGCG  81868

Query  15597  tgtgtgtgcgcgcgcacgcgtgtgtgtACCAAGAGAGGAGTCCCAGATCCGGAAAGAGGG  15656
              ||||||||              |||  |||| ||||||||| || | |  |||| |||||
Sbjct  81869  TGTGTGTG--------------TGTACACCAGGAGAGGAGTTCCTGGTTGGGAAGGAGGG  81914

Query  15657  CCAGGCCACCACTATCTCTCACTGCCCGTCCCACCACCAGGCATTGTGGCCATGATGCTG  15716
              |||||||||||  || |||||||| ||   |||| || | ||||||||||| ||||||||
Sbjct  81915  CCAGGCCACCAGCATTTCTCACTGTCCTCTCCACTACTATGCATTGTGGCCGTGATGCTG  81974

Query  15717  ACGGCCGAGCCGGAGCTCACCCTGGCTGAGCTGAGGCAG  15755
              || ||||||| ||||||||||||||| ||||||||||||
```

```
Sbjct  81975  ACTGCCGAGCTGGAGCTCACCCTGGCCGAGCTGAGGCAG  82013
```

Range 8: 82017 to 82069

Score	Expect	Identities	Gaps	Strand	Frame
69.8 bits(76)	6e-12()	47/53(89%)	0/53(0%)	Plus/Plus	

```
Query  18474  CTCTGTAAGTCTCCTTTTAAAAGCCACAGGGAACTTCTTCAAAGGAAGCCCTG  18526
              || ||||||||| || ||||||||||||||||  ||||| |||||||||||||
Sbjct  82017  CTTTGTAAGTCTTCTCTTAAAAGCCACAGGGAGATTCTTAAAAGGAAGCCCTG  82069
```

Range 9: 82297 to 83367

Score	Expect	Identities	Gaps	Strand	Frame
376 bits(416)	3e-104()	750/1098(68%)	110/1098(10%)	Plus/Plus	

```
Query  18912  AGGTGGGAGTGGGCTTGGAGAGCAAAAGGAAGAGCCAGAGGGCTGGTGAGGTGGGACC--  18969
              |||||||||   |  |||||  ||  |||||| ||||||  | ||||    |||||| |
Sbjct  82297  AGGTGGGAGCATGTCTGGAGGACACCAGGAACAGCCAGGAGCCTGG----GTGGGAACTA  82352

Query  18970  --CGAGTGGGAGGGGGAACCAGAGA-----CAG---GGTTTAGGTGGGGCCGGAGGGCCA  19019
                ||||||||||    |||||||||     |||   || | || |||| ||  | |||||
Sbjct  82353  GGCGAGTGGGAG----AACCAGAGAGGGGACAGACCGGGTCAGCTGGGACCCCAAGGCCA  82408

Query  19020  CAGGAAGGACTTGGATTTTTACTGGAGTGAGCTGGGAGCCACACAGGGTTCTGAGCCT-G  19078
              ||| |||| | | | ||||| || |||||||||||||| |||   ||||||||||||  |
Sbjct  82409  CAGTAAGGTCATTGGTTTTTGCTCGAGTGAGCTGGGAGGCACTTGGGGTTCTGAGCCCAG  82468

Query  19079  GGT----------GTGGGGAGG-----------GGGGTGGGCTA--------TCTGACCT  19109
              |||          || |||| |           ||||||||  |        |||| |||
Sbjct  82469  GGTAGAGGTAGGGGTAGGGATGAAGGTGGCAGTGGGGTGGGAGAAGGGGGTGTCTGCCCT  82528

Query  19110  GGGTGTGAGCAGGTTCATTCTGGTCGCTGTGTCGGGAAGACTGCAGGGGACAGG-GCGGA  19168
              |||| |||||   |||||||||   |||  |   ||||||||||||||||||   || ||
Sbjct  82529  GGGTCTGAGCGTATTCATTCTGCCTGCTTCGCTAGGAAGACTGCAGGGGACACAAGCAGA  82588

Query  19169  AGCAGGGAGGCCCGCTGTAGACGGGTGGACA------------------GCCCGGGTGCT  19210
              ||||||||| || || || |||  |||  ||                  |||  |||| |
Sbjct  82589  AGCAGGGAGACCTGCCGTTGACAAGTGACCATGTCCTGTGAGTATCCAGGCCTAGGTGGT  82648

Query  19211  GGGGGGTCCGTC-AGGGCGGGAGTGTAGAGGATGCTGGAATCTGAAGGAGGGGCTG-CAC  19268
               |||| || | | |||| |||||||| |||||| ||||||  |||||||   ||   ||
Sbjct  82649  TGGGGCTCAGGCCAGGGTGGGAGTGTGGAGGATACTGGAACTTGAAGGAACAGCCCACAG  82708

Query  19269  ATCTGATGGCCTGGATATTGG-GGGAGCAGTGGAGGGGGCGTCCAAGGGTTTTGCTTTGC  19327
              ||||| ||| ||||   | || ||||| ||||| ||| | |||||| | |||||||| |
Sbjct  82709  ATCTGGTGGGCTGGGCGTGGGCGGGAGGAGTGGGGGGCGGGTCCAAAGTTTTTGCTTCGT  82768

Query  19328  TCTCGGACGAATGGCATCGCCCCTGACTGGGATGGGAAGGGCTGTGAGAGGTCAAG-TGT  19386
              ||  ||||  ||||    |  | |||| |||  ||||| | |||||  ||||   | |||
Sbjct  82769  TCCTGGACCCATGGTGCTGTAC-TGACCGGGTCGGGAATG-CTGTGGAAGGTGTGGGTGT  82826
```

```
Query  19387  CGGGGAAGTTGAGGCATTTATGCGG-GCCTGGCTCACAGCGTGCCGTGCCTTACATGTGC  19445
               ||||||| || ||  || | |    || ||| |||    |||| |||   ||| |||||
Sbjct  82827  TGGGGAAGATGGGGAGTTCAGGATTAGCGTGG-TCAT---GTGCTGTGTACTACGTGTGC  82882

Query  19446  TTTCTTTTGTCCCCGGGCCCTGGCAGGTCACCGTGGCCTGCAAGGAGGGCTGGACGCTGA  19505
              ||||||  ||||| |||||||||| || ||| | || ||| |||||||||||||| ||||
Sbjct  82883  TTTCTTCAGTCCCTGGGCCCTGGCGGGGCACTGAGGTCTGTAAGGAGGGCTGGACACTGA  82942

Query  19506  CCGGCTGCGGGGCCCACCCCGGGGCCTCCCACACCCTGGGGGCCTATGCAGTGGACAACA  19565
              || |||| ||||| | |||||||||||||||||  | ||||||| |  |||||||| |||
Sbjct  82943  CCCGCTGTGGGGCTCTCCCCGGGGCCTCCCACATTCCGGGGGCCCACACAGTGGACGACA  83002

Query  19566  CGTGTGTGGT------GAGGGGCCGGGACGTGGGTGTGCGAGGCAGGACGGGTGAGGAGG  19619
              || |||||||       ||| || ||||| | ||||   |||||||||   |||||||||
Sbjct  83003  CGCGTGTGGTAAGAAGCAGGAGCTGGGACATTGGTGCAGGAGGCAGGATCAGTGAGGAGG  83062

Query  19620  CCGCCGTGGCCATTGCCATCTGCTGCAGGAGCCGG---TCAGGGGAGCAGGCCTCCCCGG  19676
              || | |  ||||  |||||||||||| | || | |   |||||| |||||| | ||| ||
Sbjct  83063  CCACGGCAGCCACAGCCATCTGCTGCGGAAGTCAGCTTTCAGGGAAGCAGGACACCCGGG  83122

Query  19677  GGACCCAGTGACAGCCCCGCCCAGGATATCTGCG---TGGCTGGGGTCCCAGGCCTTGGC  19733
               |  ||||||||||| ||||| |||    | | |    |||||||| ||        || 
Sbjct  83123  AGTTCCAGTGACAGCGCCGCCAAGG---GCGGAGAAAGGGCTGGGGCCC--------GGA  83171

Query  19734  TGAGCTTTGAAGTGCTTCCTTTTTCCTCCTTCCTCAGCCCTCCTCAGCCTGGGCCCCGGG  19793
              |  |||| | | || |||||  ||     |||||||||||  |||||||||||| || ||
Sbjct  83172  T--GCTTCGCAATGGTTCCTCCTTATCTGTTCCTCAGCCCGGCTCAGCCTGGGCTCCAGG  83229

Query  19794  GGACAGAAGGCACCTCTTTC-TCCTGGAGCTCTGGTGCTGGCACT-TGGGGTACACT-GG  19850
               | |  |||  || |||  | | ||| |||| ||||||||||||| |||   |   | ||
Sbjct  83230  AGGCCAAAGATACGTCTACCTTTCTGCAGCTGTGGTGCTGGCACTCTGGCAAAGGGTGGG  83289

Query  19851  CTCCCTGCCTGGGAGAACCCCATCTC--TTGGCCCGAGTCACCCCTCCCCAGACCCGAGC  19908
              ||||||||||| |||||||  |||||     ||| |  |||  ||  |||||||| ||||
Sbjct  83290  CTCCCTGCCTGAGAGAACCTGATCTCAGCCTGCCTGGATCATTCCCTCCCAGACCTGAGC  83349

Query  19909  TGAGTGGGAGGTTGAATG  19926
               || ||||||| ||| ||
Sbjct  83350  CGAATGGGAGGCTGAGTG  83367
```

Range 10: 52706 to 52738

Score	Expect	Identities	Gaps	Strand	Frame
38.3 bits(41)	0.034()	28/33(85%)	0/33(0%)	Plus/Minus	

```
Query  19045  AGTGAGCTGGGAGCCACACAGGGTTCTGAGCCT  19077
              |||||||||||| |||| || | |||| |||||
Sbjct  52738  AGTGAGCTGGGAACCACTCAAGATTCTTAGCCT  52706
```

Range 11: 52620 to 52654

Score	Expect	Identities	Gaps	Strand	Frame

38.3 bits(41)	0.034()	30/35(86%)	1/35(2%)	Plus/Minus

```
Query  19147  AGACTGCAGGGGACA-GGGCGGAAGCAGGGAGGCC  19180
              |||||||||||||||  ||  ||||||||||| ||
Sbjct  52654  AGACTGCAGGGGACACTGGTAGAAGCAGGGAGACC  52620
```

Taxonomy

Reports

- **Lineage**
- **Organism**
- **Taxonomy**

Dot Plot

Plot of lcl|Query_34729 vs lcl|Query_34731

Top
