# Supplementary material for: Molecular evolution of PCSK family: Analysis of natural selection rate and gene loss
Source: PLoS One. 2021 Oct 28;16(10):e0259085. doi: 10.1371/journal.pone.0259085 (PMC8553125; doi:10.1371/journal.pone.0259085)
Supplement: S6 File — Regions indicating changes in coding sequence or frame are highlighted (if applicable). (PDF) [file pone.0259085.s012.pdf]

COVID-19 Information

Public health information (CDC) | Research information (NIH)

SARS-CoV-2 data (NCBI) | Prevention and treatment information (HHS) | Español

BLAST ® » blastn suite-2sequences » results for RID-H20CZTB5114

**Job Title**	Nucleotide Sequence ...
**RID**	H20CZTB5114 Search expires on 08-10 18:21 pm
**Program**	Blast 2 sequences
**Query ID**	lcl|Query_27483 (dna)
**Query Descr**	None ...
**Query Length**	20287
**Subject ID**	lcl|Query_27485 (dna)
**Subject Descr**	ref|NC_040252.1|:30895611-31311082 Ovis aries strain OAR_USU_Benz2616 breed Rambouillet chromosome 1, Oar_rambouillet_v1.0, whole genome shotgun sequence ...
**Subject Length**	415472

Descriptions

Description	Scientific Name	Max Score	Total Score	Query Cover	E value	Per. Ident	Acc. Len	Accession
ref|NC_040252.1|:30895611-31311082 Ovis aries strain OAR_USU_Benz2616 breed Rambouillet chromosome 1, Oar_rambouillet_v1.0, whole genome shotgun sequence		1035	3533	20%	0.0	75.09%	415472	Query_27485

Graphic Summary

Distribution of the top 19 Blast Hits on 1 subject sequences

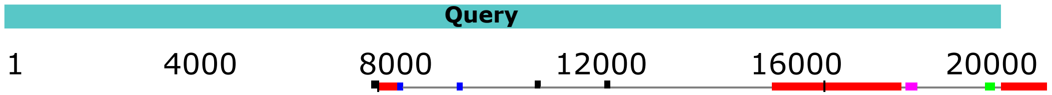

Alignments

Alignment view Pairwise CDS feature Restore defaults

ref|NC_040252.1|:30895611-31311082 Ovis aries strain OAR_USU_Benz2616 breed Rambouillet chromosome 1, Oar_rambouillet_v1.0, whole genome shotgun sequence

Sequence ID: Query_27485 Length: 415472 Number of Matches: 19

Range 1: 114083 to 115653

Score	Expect	Identities	Gaps	Strand	Frame
1035 bits(1147)	0.0()	1197/1594(75%)	84/1594(5%)	Plus/Plus	

```
Query  16087   CTGCTCTCCCTGACCAGGAGCCAAAGGTCTGGCGTCCCCTGTGAGCAGAGCCCTGACGGA  16146
               ||||||||    |||||||||||| |  |||| |||| | | | |||||||||||| |||
Sbjct  114083  CTGCTCTCTTCCACCAGGAGCCAA-GACCTGGAGTCCTCCGCGGGCAGAGCCCTGATGGA  114141

Query  16147   GGCTCCGCTCCCCAGCGC-CCCTTCTCACCCCGGGGCCTTGTTGCAGGTGGGACGCTGTT  16205
               |||||| |||||||||   |||| |||||||| ||||||  ||| ||| |||  |||| |
Sbjct  114142  GGCTCCCCTCCCCAGCCATCCCTCCTCACCCCAGGGCCTCCTTGAAGGCGGGCAGCTGCT  114201

Query  16206   CTGCAGGACCGTGTGGTCTGCACACTCAGGACCCACGCGGATGGCCACGGCTGAGGCCCG  16265
               ||| |||||||||||||| |   |||| || || | || |||||| | | ||||||||| 
Sbjct  114202  CTGTAGGACCGTGTGGTCGGTGTACTCTGGGCC-ATGCAGATGGC-ATGACTGAGGCCCA  114259

Query  16266   CTGCACAGCCCCTGAGGAGCTTCTGGGCTGCTCCAGCTTCTCCAGGAGCGGGAGGCGGCG  16325
               ||||||  ||  ||||||| | ||||||||||||| |||||| |||| || |||||| | |
Sbjct  114260  CTGCACCTCCT-TGAGGAGTTGCTGGGCTGCTCCAACTTCTGCAGGGGCAGGAGGCCGTG  114318

Query  16326   GGGCGAGCGCATTGAGGTGACCTGCAGGCCCCCGTCGGAGCCTGAAGTGGGGTTCTCGCT  16385
               ||||||| |||| |||||||||||    | |||   ||||  ||| | |||||||| ||||
Sbjct  114319  GGGCGAGTGCATCGAGGTGACCTGTGCCCTCCCACAGGAGTATGAGG-GGGGTTCCCGCT  114377

Query  16386   TCCAGGTCCAGATCCGCCTGAGCCCCTTCCTCTGCTGAGCTCCAGGCGCCCGCCTGCAAG  16445
               | |||| ||||||| |||||  ||||||||||||  |||| ||||||   |||  |||||||
Sbjct  114378  TTCAGGCCCAGATCTGCCTGGACCCCTTCCTCCTCTGAACTCCAGTGCCCCTTCTGCAAG  114437

Query  16446   TTAAAGCAGGATGGGGCACGTCTCAGTCACATGGCT-GGGTGCTGCTGCAGGGAGCCACA  16504
               ||||||    ||||||||  |||  ||||| | | | ||||||| ||  | |||||||| 
Sbjct  114438  TTAAAGTTAAATGGGGCATATCT--GTCACTTAGTTTGGGTGCT-CTAGAAGGAGCCACG  114494

Query  16505   CTGAGGTTTCCCAGGAGACT--GCAGGACGGTGGCTAGATGGATTCCAGCGAC-----CG  16557
               ||||||  |||  |||||||  | |||| |  |||| |||||||| ||||  |     ||
Sbjct  114495  CTGAGGGGTCCTGGGAGACTCTGTAGGATGA-GGCTGGATGGATTGCAGCACCTGCCTCG  114553

Query  16558   ACCGTCTGGGG--------------AGCG---------------GGAGGGCTG-----GG  16583
```

```
                 |  |  |||||              || |               ||||||  |     |
Sbjct  114554    AGGGGGTGGGGCCAGTGTGTGTGCCAGGGACTCACTGCCTTAGAGGAGGGGAGCACCAGA  114613

Query  16584     CATGGGCCAGGGACTCGCTGCCTCTGGACTCACTGGTCCCCAGGGCTCTTT-CACTCAGA  16642
                 | || ||  |||| ||| |||||||| || || ||||| ||||||| |||| | ||||||
Sbjct  114614    CGTGTGCAGGGGATTCGTTGCCTCTGCACACAGTGGTCTCCAGGGCCCTTTTCTCTCAGA  114673

Query  16643     TGTTACATAGTTCCAGCAGCTGAGAAATCTTCTCAAACCAGCAGCAGAGGGGACTTGATA  16702
                 || | ||| ||||  |||| ||||||||||   ||||||||||  |||| || | |||
Sbjct  114674    TGGTCCATGGTTCTGGCAGATGAGAAATCT---CAAACCAGCAAAAGAGTGGGCCTGAGG  114730

Query  16703     TTAAGGCCACAGAGCCTTACAGAGATGCCAACTGGCCAGGGCGTTTTGGTGGAAGGACAG  16762
                 ||||||||| |||| ||  || |||||||| |||| || ||| ||||||||||||| |||
Sbjct  114731    TTAAGGCCAGAGAGTCTGGCACAGATGCCATCTGGTCATGGCCTTTTGGTGGAAGGGCAG  114790

Query  16763     TGCCTCGGCCAGGAGGAC-GGGGTGGGCAGGCATTTCTGCCTGGGAGACGGTGTCTGGGA  16821
                   | | |||||||||||| |||| |||||  ||||| |||| ||||||| |||||  | |
Sbjct  114791    CACTTAGGCCAGGAGGACAGGGGCGGGCAATCATTTGTGCCCGGGAGACAGTGTCCAGCA  114850

Query  16822     GTGTGTGTGACCATGCACTTGATCCTGCAAGTGAGAGTATGTGGGCGGCGTGGCCGAGAG  16881
                 |||||  ||||  ||| ||||| |||||| || || | ||||||| |    ||| |||||
Sbjct  114851    GTGTGCTTGACGGTGCCCTTGAACCTGCAGGTTAGGGCATGTGGGAGA---GGCTGAGAG  114907

Query  16882     CAGGTCAGGGCTGAGGAGGCGGGGGCCTTGCTC-GGGGTCTTAGGTTTCCCTGTATCTGC  16940
                   || |||||||||| || | ||| ||| |||| |||||||  | |||||||||||||||
Sbjct  114908    TGGGGCAGGGCTGAGCAGTCTGGGTCCTGGCTCTGGGGTCTATGATTTCCCTGTATCTGC  114967

Query  16941     ATTTTATGGTCATGCTTAGAGCCCAGAAGAACTTTATTACA--CACAGCTGCCCATGTGC  16998
                 ||| |   ||||  ||  | | |||||||||||| || | |  || |||||||||| |||
Sbjct  114968    ATTCTGAAGTCACTCTCGGTGTCCAGAAGAACTTGATAATATTCAGAGCTGCCCATCTGC  115027

Query  16999     TGAGCAGTTTG-------CAGGAGGGAGGTCCTGGTCCTCAGAGG--GGCAGGCTCCTGG  17049
                  || | || ||       ||| || ||| |||| ||| |||| ||  ||| ||| ||  |
Sbjct  115028    AGAACGGTCTGTCTTGGACAGCAGTGAGTTCCTTGTCTTCAGGGGCTGGCTGGCCCCGTG  115087

Query  17050     CAGGGACGGTGGAGATGGTATGAGGGACTGGGACCAGCTGCTTGAGCCTGTCCCTTTCAG  17109
                 | |||||  |||||| |||  ||||| ||||||||   ||  |||    ||||| | |
Sbjct  115088    CTGGGACATTGGAGAGGGTTCGAGGGGCTGGGACCCAGTGTCTGAATTGGTCCCCTCCCA  115147

Query  17110     CCCCCTCATTCTGTGTTTCAAAGCCCTTTCTAAAGCATGTTTCTGTTTCTGTCTTTGGCT  17169
                 |||| |||| |||||||| || |||||||||||  || |     |||||||| |||||||
Sbjct  115148    CCCC-TCATCCTGTGTTTTAACGCCCTTTCTAAGACAGG-----GTTTCTGTTTTTGGCT  115201

Query  17170     TTCAGGCCCGAGGGGGCAGGCATGTCTGCCTGGCCCACAATGCGTTTGGGGGTGAGGGTG  17229
                  |||||||| || |||||| |||||||||||||||||||| ||||||||||| |||||||
Sbjct  115202    CTCAGGCCCTAGAGGGCAGTCATGTCTGCCTGGCCCACAACGCGTTTGGGGGCGAGGGTG  115261

Query  17230     TCTATGCCGTTGCCAGATGCTGCCTGCTGCCCCAGGCCAACTGCAGTGTCCACACAGCTC  17289
                 |||  || |||||||||||||||||||  || | ||||||||||||  |||||||||||
Sbjct  115262    TCTGCGCTGTTGCCAGATGCTGCCTGCCTCCGCGGGCCAACTGCAGCATCCACACAGCTT  115321

Query  17290     CGCCAGCCAGGGCTGGTGTGCTGACCCAAGCCCACTGCCACCAGCAGGGCCACGTCCTCA  17349
                  ||||||  |||||||||||| |||||| | |   |||| |||||||||| ||||  |||
Sbjct  115322    TGCCAGCTGGGGCTGGTGTGCAGACCCATGTCTGATGCCCCCAGCAGGGCTACGTGTTCA  115381

Query  17350     CAGGTAGGAGGCTGGGCCCATCCTGGGGTGAAGAGGCTTCCTTGTCTCCTGGTGCACCTG  17409
                 ||||||||||||||||| || |||||||||| | ||| |||||| ||||  ||||||| |
Sbjct  115382    CAGGTAGGAGGCTGGGCTCACCCTGGGGTGAGGCGGCATCCTTGGCTCCCTGTGCACCAG  115441

Query  17410     CTCCCACCTGACTGGTCCCATGCTGGGGCCCAACTGCCTGGTGCGAAGGCCTGTGCTA-C  17468
```

```
               ||  || ||| ||||||||| ||||| |  |||||| |||||| ||||||| |||||| |
Sbjct  115442  CTTACATCTGGCTGGTCCCACGCTGGAGTGCAACTGTCTGGTGGGAAGGCCAGTGCTACC  115501

Query  17469   CCTTCCATCCCTGTGACCCTGGGTGGGCACCTCATTGGTCTCAGTCTCAGCTTCTTCCTC  17528
               |||||||||||||   ||||| |  ||||||||||  ||||||||||||||||| |||||
Sbjct  115502  CCTTCCATCCCTGGAGCCCTGAGCAGGCACCTCATCTGTCTCAGTCTCAGCTTCCTCCTC  115561

Query  17529   CCTAAGAAGAATGACGGTAGTTCCTGCCTCAATGGGTTGCCATGG---AATGAGTAAGCC  17585
               ||||||| |      ||||| |||||| |||  | |||||  |||   ||||  |||  |
Sbjct  115562  CCTAAGAGGGGCCGTGGTAGCTCCTGCTTCACCGCGTTGC--TGGGAAAATGCATAACAC  115619

Query  17586   CTAGAGCACCAGGCCTGGAGCATCCAGGGCACTT  17619
               ||||||||||| ||||||||| ||   |||||||
Sbjct  115620  CTAGAGCACCAAGCCTGGAGCTTCACAGGCACTT  115653
```

Range 2: 112866 to 113805

Score	Expect	Identities	Gaps	Strand	Frame
692 bits(767)	0.0()	749/976(77%)	55/976(5%)	Plus/Plus	

```
Query  15138   TGCCGCCCACGTGGCTGGTGAGTTGCTGCCCTACCACCTCAGCCACCGTGATTCTAACCA  15197
               |||| |||  ||| ||||||||| ||||||||       |||||| | |||||||  ||
Sbjct  112866  TGCCACCCGTGTGACTGGTGAGTAGCTGCCCT-------CAGCCATCATGATTCTGACAG  112918

Query  15198   CCCCTTTGGGAGCCAGGATCTGCGCCAGAACCCCATGTGCCAGGCTCTGTGTTGGACACG  15257
               ||| ||||  || |||| |||||  ||| ||||| |||||||||||||||| ||| |  |
Sbjct  112919  CCCATTTGTCAGGCAGGGTCTGCAACAGGACCCCTTGTGCCAGGCTCTGTGCTGGGCGTG  112978

Query  15258   GGGGACTAAAGAGGAATCAGACTGATGGTGCCCTCAAAGACTCTCAGTCTGATGGGTGAG  15317
               ||| |||||||| || |||||  |||||||||||||| | | |||||||||||||| |||
Sbjct  112979  GGG-ACTAAAGATGAGTCAGATAGATGGTGCCCTCAAGGTCGCTCAGTCTGATGGGGGAG  113037

Query  15318   GCAGGTGCACAAACAGAGTAGCCAGGGCTGTGTGGAAGGGAGCCCAGAG-AGGTACCCAC  15376
               |||| ||||||    ||||||||||||||||||||||||||||||  || |||| ||  |
Sbjct  113038  GCAGATGCACA----GAGTAGCCAGGGCTGTGTGGAAGGGAGCCCCCAGGAGGTGCCTGC  113093

Query  15377   CCAGCTTAAAGGTCAGGGAAAGCTTCCTAG--------CATTTTATTTGGGGTTTGGTGG  15428
               ||    |||||||| ||||||  |||| ||        ||||| || | |||||| ||||
Sbjct  113094  CCCCACTAAAGGTCGGGGAAATTTTCCCAGAGGAGGGACATTTCATCTTGGGTTTAGTGG  113153

Query  15429   ATGAATAGGAGTTTACCTGGCAAGCAAAACAGCAATAGTCAAGGCTCAGAGGTATGGGAG  15488
               |||||||  ||||||||||||||  ||||||||||  | |||||||||||||| || ||
Sbjct  113154  ATGAATAAAAGTTTACCTGGCAAATAAAACAGCAACTGCCAAGGCTCAGAGGTGTGAGAA  113213

Query  15489   CAGGATGTAAGATAGTCTTACTCTTTGGCTGTCTTTTAACC-TGGGGTTGCAGGTCTTTT  15547
               |||||| ||||| |||| | ||||   ||||| |||||| | || | ||||||||  |||
Sbjct  113214  CAGGAT-TAAGAGAGTCGTGCTCTC--GCTGTTTTTTAAGCATGAGATTGCAGGTGGTTT  113270

Query  15548   AACTTCTGAGGAACAGCCTGgtgtgtctctgtgcatgtgtgtgtgtgtgtgtgtgtgcgc  15607
               || |||||| ||||||  ||||| |||  |||||||   ||  ||||||||||  || |
Sbjct  113271  AATTTCTGAAGAACAGTTTGGTGCGTCCATGTGCATCCATGCATGTGTGTGTGC-TGGG-  113328

Query  15608   gcgcacgcgtgtgtgtACCAAGAGAGGAGTCCCAGATCCGGAAAGAGGGCCAGGCCACCA  15667
                       |||| |||     | |||||||||||||||  ||| ||||||  ||||||||
Sbjct  113329  --------GTGTCTGT-----GTGAGGAGTCCCAGATCGAGAAGGAGGGCTGGGCCACCA  113375
```

```
Query  15668   CTATCTCTCACTGCCCGTCCCACCACCAGGCATTGTGGCCATGATGCTGACGGCCGAGCC  15727
               || |||||||||||| | |||   |||||||   |||| | ||||||||||  |||||||
Sbjct  113376  CTGTCTCTCACTGCCTGCCCCCTGACCAGGCGGCGTGGTCGTGATGCTGACCACCGAGCC  113435

Query  15728   GGAGCTCACCCTGGCTGAGCTGAGGCAGAGACTGATCCATTTCTCTGCCAAAGACGTCAT  15787
                |||||||||||||||| |||||||||||| ||||||  ||||||||||||||| || ||
Sbjct  113436  AGAGCTCACCCTGGCTGCGCTGAGGCAGAG-CTGATCTGTTTCTCTGCCAAAGATGTTAT  113494

Query  15788   CAACAAGGCCTGGT--TTCC-CGAAGACCAGCGGGTGCTGACCCCCAACCTGGTGGCCAC  15844
               ||||||||||||||  ||||  || |||||||   |   || ||||| ||||||||||||
Sbjct  113495  CAACAAGGCCTGGTTCTTCCTTGAGGACCAGC---TCGGGAACCCCAGCCTGGTGGCCAC  113551

Query  15845   ACTGC--CCCCCAGAACCTATAAAGCAGGTCAGCAGGGCGGCAAGGTGGGCAGAATCCAG  15902
               || ||  ||| | | ||||||  ||||||||||||||  |||| |||||||||| |||||
Sbjct  113552  ACCGCAACCCTCCGCACCTATGGAGCAGGTCAGCAGGATGGCAGGGTGGGCAGAGTCCAG  113611

Query  15903   ACTGGGGCTTGGGGGGTCTCGGGAGGTCTGTGTGACCTGGGTAGGCTTGTCCATCCTCAT  15962
                |||||||||||| ||||| ||| ||||||||||| ||||| |  || | || |||
Sbjct  113612  GCTGGGGCTTGGGAGGTCTTGGGTGGTCTGTGTGA-CTGGGCAATCTGGCCCCTCCCTCC  113670

Query  15963   CTGTGGAGGGAGAATTACACCA-GAGGTTCCTAGAAATGGGAGGAGATGCATAGA-AGAG  16020
               || ||||||||| |||   |||   | |||   | | ||||| |||||  | | | |  |
Sbjct  113671  CTATGGAGGGAGGATTCAGCCACCTGCTTCTGGGGACTGGGA-GAGATTGACAAATATGG  113729

Query  16021   GCTCAGAAAGGGCTTGGCAGGGCGTTCATGA--TGTTTTGATGGAAAAATTGATCATGTT  16078
               |||| |||||| ||| |||||||  | ||||  ||||||  ||| |||||||||||  ||
Sbjct  113730  GCTCTGAAAGGACTTTGCAGGGCTCTGATGATGTGTTTTAGTGGGAAAATTGATCACCTT  113789

Query  16079   CTTTAAGGCTGCTCTC  16094
               |||||||||  |||||
Sbjct  113790  CTTTAAGGCCCCTCTC  113805
```

Range 3: 117394 to 118237

Score	Expect	Identities	Gaps	Strand	Frame
554 bits(614)	8e-158()	662/892(74%)	85/892(9%)	Plus/Plus	

```
Query  19431   GTGCCTTACATGTGC--TTTCTTTTGTCCCCGGGCCCTGGCAGGTCACCGTGGCCTGCAA  19488
               ||||||  |||||||  ||||||||||||  ||||||||||||||||||||||||||  |
Sbjct  117394  GTGCCT--CATGTGCCCTTTCTTTTGTCCTGGGGCCCTGGCAGGTCACCGTGGCCTGGGA  117451

Query  19489   GGAGGGCTGGACGCTGACCGGCTGCGGGGCCCACCCCGGGGCCTCCCACACCCTGGGGGC  19548
                 || ||||||| ||||| ||||||||| | ||||| | |||||||||||||  |||| |
Sbjct  117452  C-AGCGCTGGACACTGACTGGCTGCGGGACTCACCCGGAGGCCTCCCACACCACGGGGAC  117510

Query  19549   CTATGCAGTGGACAACACGTGTGTGGTGAGGGGCCGGGACGTGGGTGTGCGAGGCAGGAC  19608
               ||| |  |||||||||||| || || || ||||| ||||||| ||||
Sbjct  117511  CTACGTGGTGGACAACACGCGTCTGATGTGGGGCTGGGACGTTGGTGC------------  117558

Query  19609   GGGTGAGGAGGCCGCCGTGGCCATTGCCATCTGCTGCAGGAGCCGGTCA----GGGGAGC  19664
                    |||||||  | || ||  |  |||||||||||  ||||| | |     || ||||
Sbjct  117559  -----AGGAGGCTCCTGTAGCTGTCACCATCTGCTGCCAGAGCCAGCCGTTGGGGAGAGC  117613

Query  19665   AGGCCTCCCCGGGGACCCAGTGACAGCCCCGCCCAGGATA-----------------TC-  19706
               |||||||  ||| | ||||||||| |||| |||||||| |                 ||
Sbjct  117614  AGGCCTCTTCGGAGGCCCAGTGACTGCCCTGCCCAGGACACTTACACGGGGAAGGGGTCA  117673
```

```
Query  19707   TG-CGTGG---CTGGGGTCCCAGGCCTTGGCTGAGCTTTGAAGTGCTTCCTTTTTCCTCC  19762
               || | |||   ||| ||| |||||||||||||||||||||| || |||||| |||| ||
Sbjct  117674  TGTCCTGGATGCTGTGGTGCCAGGCCTTGGCTGAGCTTTGAACTGGTTCCTTCTTCCCCC  117733

Query  19763   TTCCTCAGCCCTCCTCAGCCTGGGCCCCGGGGGACAGAAGGCACCTCTTTCTCCTGGAGC  19822
                || ||||||||| |||||||||  ||| ||   |||| |   |  ||| ||||||||||
Sbjct  117734  CTCTTCAGCCCTCTTCAGCCTGGAGCCCAGG---CAGAGG---CTACTTCCTCCTGGAGC  117787

Query  19823   TCTGGTGCTGGCACTTGGGGTACACTGGCTCCCTGCCTGGGAGAACCCCATCTCTTGGCC  19882
               |||||||||||||||| |||| | | |||| ||||||||||| |||||||  |||  |||
Sbjct  117788  TCTGGTGCTGGCACTTAGGGTGCGCCGGCT-CCTGCCTGGGAAAACCCCAGGTCTCAGCC  117846

Query  19883   CGAGTCACCCCTCCCCAGACCCGAGCTGAGTGGGAGGTTGAATGAGCAGGGCCACAGGCG  19942
                |  ||||||| |||||| || ||||||| | ||||  |||  ||||| |||  ||||||
Sbjct  117847  TGCATCACCCCGCCCCAGGCCTGAGCTGAAT-GGAGCCTGATGGAGCAAGGCTGCAGGCG  117905

Query  19943   CC-GGCAGCCCCTCCCTCACTGAGGGGCTGTGTCCACATG--TCCATCAACAAGGGTCTG  19999
               || ||||| ||||| |||| ||| | | |||||||||   || ||| ||  || |
Sbjct  117906  CCAGGCAGGCCCTCTCTAACTGCGGGACGGGGTCCACATGCCACCGTCAGCACAGGCCAA  117965

Query  20000   GCTGTGCTCAGCTCCCTGTCAGCTGCTCCCAAGTTGCCAGTGCTGTGGGCAGAATTAGCT  20059
               ||||||||||||| |||| ||||  ||||  || |||||    ||||||||||||| | ||
Sbjct  117966  GCTGTGCTCAGCTTCCTGCCAGCCACTCCGCAGATGCCAACATTGTGGGCAGAATGACCT  118025

Query  20060   TTTGTTGAGTTCTTGCTACATGTCAGCCAGGCAGTCAGTCCTCAGGCCTCCATGAAGGAG  20119
               ||| | |||                  ||| |||||||| ||||||||||||||||||||
Sbjct  118026  TTTATGGAG------------------CAGTCAGTCAGTGCTCAGGCCTCCATGAAGGAG  118067

Query  20120   GTGGTAACCCTCCTATGGGGAGGCAAGGAAGCACTTGACGGCTGGGAGAGGCCAAATGTT  20179
               ||||| | |||||  ||||||||||  || |||||||| |||| | ||||||||||| |
Sbjct  118068  GTGGTCATCCTCCCGTGGGGAGGCAGTGAGGCACTTGAAGGCTAGAAGAGGCCAAATATC  118127

Query  20180   GGTCA-GAGGATGTGAAAGGTGGAAATGGCCCCTCACCTCCTGCCCACTCTGGGGAGGCC  20238
                |||| | ||| ||||||||| || |||||||||||| |||| |||||| ||||||||||
Sbjct  118128  AGTCAGGGGGACGTGAAAGGTAGAGATGGCCCCTCACTTCCTACCCACTTTGGGGAGGCC  118187

Query  20239   CGGT---TGGGCTCCCTGATTA-TGGA-GATGAGTTTTCCATGCCTCTGGGG  20285
               | ||     |||  |||||||| |||| |||  ||||||| |||||||| ||
Sbjct  118188  CCGTGAGGAGGCCTCCTGATTAGTGGAGGAT--GTTTTCCCTGCCTCTGAGG  118237
```

Range 4: 131419 to 131749

Score	Expect	Identities	Gaps	Strand	Frame
204 bits(225)	5e-52()	261/354(74%)	27/354(7%)	Plus/Minus	

```
Query  7464    ttttttAATTGAAGTATAGTCAGTTCACAGTGTTGTGTAAATTTCTGGTGCAAAGCATAA  7523
               ||||||||||||||||||||   || ||| | |||||| | ||||  ||| | | || |
Sbjct  131749  TTTTTTAATTGAAGTATAGTTGATTTACAATATTGTGTTAGTTTCAAGTGTACAACAAAG  131690

Query  7524    TGTTTCGGTCATACACATACATACATATATTCCTTTTCATATTCTTTTTCACTATAGGTT  7583
               || ||| || |         |||   |||||  ||||||  || |||| || ||||||||
Sbjct  131689  TGATTCAGTTA---------ATA---ATATT--TTTTCAGGTTGTTTTCCATTATAGGTT  131644

Query  7584    ATTACAAGCTATTGAATATAGTTCCCCGTGCTACACAGTAGGACCTTGCTGTTAATCTAT  7643
                ||||||| || |||||||||||||| |||||||||||||   ||||| |  ||| ||||
```

```
Sbjct  131643  GTTACAAGATACTGAATATAGTTCCCTGTGCTACACAGTAAATCCTTGTTTCTAACCTAT  131584

Query  7644    TTTATATATAGCAGTTTGTATCTGCAAATGCCGATCTC--CCAATTTATCCCTCCAT--C  7699
               ||  ||||||| ||||||| ||||  ||| ||   |||  | || |||||||||| |
Sbjct  131583  TTCGTATATAGTAGTTTGTGTCTGTTAATCCCATACTCCTCTAACTTATCCCTCCTTTCA  131524

Query  7700    CTCCTTCCAGCCCCGGGAACCACAAGtttgttttctatgtctgtgagtctgtttctgttt  7759
               | |||| |  ||| || ||||| ||||||| ||||| |||||||| |||||||||||  |
Sbjct  131523  CCCCTTTC--CCCTGGTAACCATAAGTTTG-TTTCTGTGTCTGTGTGTCTGTTTCTG-CT  131468

Query  7760    ttttaaataagttcatttgtgtctttttttttttAGATTCCACATATAAGTGATA  7813
               || || |||  ||| ||||     | || ||| |||||||||||||||  ||||
Sbjct  131467  TTGTATATAGATTCTTTTG-----TATTATTTAAGATTCCACATATAAAGGATA  131419
```

Range 5: 129200 to 129466

Score	Expect	Identities	Gaps	Strand	Frame
182 bits(201)	2e-45()	205/272(75%)	7/272(2%)	Plus/Plus	

```
Query  7544    ATACATATATTCCTTTTCATATTCTTTTTCACTATAGGTTATTACAAGCTATTGAATATA  7603
               ||||||||| |  |||||  ||| |||  || || || ||||||||||  | |||| | |
Sbjct  129200  ATACATATACT--TTTTCTGATTGTTTCCCATTACAGATTATTACAAGAAACTGAAGACA  129257

Query  7604    GTTCCCCGTGCTACACAGTAGGACCTTGCTGTTAATCTATTTTATATATAGCAGTTTGTA  7663
               |||| | |||||| |||||||| |||||||| |  |||||||| || |||| |||||| |
Sbjct  129258  GTTCTCTGTGCTATACAGTAGGCCCTTGCTGCTTTTCTATTTTGTACATAGTAGTTTGCA  129317

Query  7664    TCTGCAAATGCCGATCTCCCAATTTATCCCTCC--ATCCTCCTTCCAGCCCCGGGAACCA  7721
               ||||| |||  |   |||| ||||||||| |||  | ||||||| |  |   || |||
Sbjct  129318  TCTGCTAATCTCACACTCCTAATTTATCCTTCCTCAACCTCCTTTCCACTTTGGTAACTC  129377

Query  7722    CAAGtttgttttctatgtctgtgagtctgtttctgtttttttaaataagttcatttgtgt  7781
                |||||||||| |||||||| | | ||| |||   ||||||| ||||||||| |||||
Sbjct  129378  TAAGTTTGTTTCCTATGTCTCTAAATCTTTTTTATTTTTTTTTAATAAGTTCTTTTGTAC  129437

Query  7782    ctttttttttttAGATTCCACATATAAGTGATA  7813
               | |  ||| |   |||||||||||||||||||
Sbjct  129438  CATACTTTAT---ATTCCACATATAAGTGATA  129466
```

Range 6: 116940 to 117117

Score	Expect	Identities	Gaps	Strand	Frame
168 bits(186)	1e-41()	146/179(82%)	3/179(1%)	Plus/Plus	

```
Query  17648   CTCAGGCCAGTGTCTCGTTCCTGCCCTGACTTATTTCTGGGTTTCCCAGCTCCAGCCCCA  17707
               |||||   |||| ||| ||||| || |||||| ||||||| |||||||||| |||   ||
Sbjct  116940  CTCAGCTTAGTGCCTCTTTCCTTCCTTGACTTGTTTCTGGATTTCCCAGCTTCAGAATCA  116999

Query  17708   GACCCGAAAGAGATGGAGTCTGAATGGGGTGGG--GAGGACAGACAGATGGTCCCACAGC  17765
               ||||| ||| |||||||| ||  |||| || ||  ||||| |||||||  |||||  |||
Sbjct  117000  GACCCTAAAAAGATGGAGACT-AATGAGGACGGAAGAGGAGAGACAGAAAGTCCCTGAGC  117058
```

```
Query  17766   ATCCAGGTGTCTGAGCTGGCCCTCCTTTGCCCCAGGCTGCAGCTCCCACTGGGAAGTGG  17824
               ||||  |||||||||| || |||||| |||||||||||||||||||||||||| |||||
Sbjct  117059  ATCCGAGTGTCTGAGCCGGTCCTCCTCTGCCCCAGGCTGCAGCTCCCACTGGGCAGTGG  117117
```

Range 7: 7838 to 8150

Score	Expect	Identities	Gaps	Strand	Frame
138 bits(152)	2e-32()	229/323(71%)	11/323(3%)	Plus/Minus	

```
Query  7466  ttttAATTGAAGTATAGTCAGTTCACAGTGTTGTGTAAATTTCTGGTGCAAAGCATAATG  7525
             ||||| | ||||| ||||  ||| ||| |||||||| |||||||| || | |||| | |
Sbjct  8150  TTTTATTGGAAGTCTAGTT-GTTTACACTGTTGTGTTAATTTCTGCTGTACAGCAAAGTA  8092

Query  7526  TTTCGGTCATACACATACATACATATATTCCTTTTCATATTCTTTTTCACTATAGGTTAT  7585
                | |    | | ||  ||| || |||   | |  ||||||||||||| | |   ||||
Sbjct  8091  CAGCAGA---ATATATGTATAAATGTATATGTAT--ATATTCTTTTTCATTGTGACTTAT  8037

Query  7586  TACAAGCTATTGAATATAGTTCCCCGTGCTACACAGTAGGACCTTGCTGTTAATCTATTT  7645
             ||||   || |||||||||||||| |||||| |||||||||||| | |||  ||| |||
Sbjct  8036  TACAGAATACTGAATATAGTTCCCTGTGCTATACAGTAGGACCTCGTTGTGTATCCATTC  7977

Query  7646  TATATATAGCAGTTTGTATCTGCAAATGCCGATCTCCCAATTTATCCCTCCATCCT-CCT  7704
             || | |||  |||||| |||||| ||| || | ||| || |  |||| ||   ||| ||
Sbjct  7976  TAAAGATAATAGTTTGCATCTGCTAATTCCAAACTCTCAGTCCATCCATCTGCCCTGCCA  7917

Query  7705  TCCAGCCCCGGGAACCACAAGtttgttttctatgtctgtgagtctgtttctgtttttta  7764
               |||    || |||||||||| |||  ||| ||||||||||  ||||| || |||  ||
Sbjct  7916  CTCAG-TTTGGCAACCACAAGTCTGTCCTCTCTGTCTGTGAG--TGTTTTTG-TTTCATA  7861

Query  7765  aataagttcatttgtgtcttttt  7787
              || |||  || |||| | ||||
Sbjct  7860  GATCAGTATATGTGTGCCATTTT  7838
```

Range 8: 117155 to 117285

Score	Expect	Identities	Gaps	Strand	Frame
71.6 bits(78)	2e-12()	100/133(75%)	7/133(5%)	Plus/Plus	

```
Query  19200   GCCCGGGTGCTGGGGGGTCCGTCAGGGCGGGAGTGTAGAGGATGCTGGAATCTGAAGGAG  19259
               |||| |||| |||||  |  | ||||| ||||||||  ||||| ||||   ||||  |||
Sbjct  117155  GCCCTGGTGGTGGGGCTTA-GCCAGGGAGGGAGTGTGCAGGATACTGGGCCCTGAGAGAG  117213

Query  19260   GGGCTGCACA--TCTGATGGCCTGGATATTGGGGGAGCAGTGGA-GGGGGCGTCCAAGG-  19315
               | ||  ||||  |||||||| |||| | | |  || |||||||| ||||||  ||||||
Sbjct  117214  GAGCC-CACAGCTCTGATGGACTGGCTGTGGCAGGGGCAGTGGAGGGGGGCTGCCAAGGT  117272

Query  19316   GTTTT-GCTTTGC  19327
               ||||| |||||||
Sbjct  117273  GTTTTCGCTTTGC  117285
```

Range 9: 118561 to 118639

Score	Expect	Identities	Gaps	Strand	Frame
58.1 bits(63)	5e-08()	60/79(76%)	0/79(0%)	Plus/Minus	

```
Query  7531    GTCATACACATACATACATATATTCCTTTTCATATTCTTTTTCACTATAGGTTATTACAA  7590
               || || || ||| | |||| ||||  |||| | ||  |||| || |||||||||||||||
Sbjct  118639  GTGATTCAGATATACACATGTATTATTTTTGAAATGATTTTCCATTATAGGTTATTACAA  118580

Query  7591    GCTATTGAATATAGTTCCC  7609
                 |||||| |||| || ||
Sbjct  118579  TATATTGACTATAATTGCC  118561
```

Range 10: 256069 to 256139

Score	Expect	Identities	Gaps	Strand	Frame
55.4 bits(60)	2e-07()	55/71(77%)	3/71(4%)	Plus/Minus	

```
Query  7792    tAGATTCCACATATAAGTGATAGCAT---GGATTTTTCTTTCTCTTTCTGGCTTACTTCA  7848
               || |||||| ||||| ||| ||| ||   | |||  | ||| ||||||||||||||||||
Sbjct  256139  TAAATTCCATATATATGTGTTAGTATACTGTATTGGTGTTTTTCTTTCTGGCTTACTTCA  256080

Query  7849    CTTGGTATGAT  7859
               ||  |||| ||
Sbjct  256079  CTCTGTATAAT  256069
```

Range 11: 35733 to 35807

Score	Expect	Identities	Gaps	Strand	Frame
44.6 bits(48)	3e-04()	55/75(73%)	3/75(4%)	Plus/Minus	

```
Query  7788   tttttAGATTCCACATATAAGTGATAGCAT---GGATTTTTCTTTCTCTTTCTGGCTTAC  7844
              ||| || |||| | ||||| |   ||| ||   | |||  | ||| ||||||||||||||
Sbjct  35807  TTTCTAAATTCTATATATATGCATTAGTATACTGTATTGGTGTTTTTCTTTCTGGCTTAC  35748

Query  7845   TTCACTTGGTATGAT  7859
              ||||||  |||| ||
Sbjct  35747  TTCACTCTGTATAAT  35733
```

Range 12: 320555 to 320620

Score	Expect	Identities	Gaps	Strand	Frame
44.6 bits(48)	3e-04()	55/73(75%)	7/73(9%)	Plus/Plus	

```
Query  7786    tttttttAGATTCCACATATAAGTGATAGCATGGATTTTTCTTTCTCTTTCTGGCTTACT  7845
               ||||| ||| |||||||| || |  |||  ||  ||||| ||||    ||||| ||||||
```

```
Sbjct  320555  TTTTTCTAGGTTCCACATGTACGCAATAT-AT--ATTTTCCTTT----TTCTGACTTACT  320607

Query  7846    TCACTTGGTATGA  7858
               |||||  ||||||
Sbjct  320608  TCACTCTGTATGA  320620
```

Range 13: 411034 to 411108

Score	Expect	Identities	Gaps	Strand	Frame
44.6 bits(48)	3e-04()	55/75(73%)	3/75(4%)	Plus/Minus	

```
Query  7788    tttttAGATTCCACATATAAGTGATAGCAT---GGATTTTTCTTTCTCTTTCTGGCTTAC  7844
               ||| || |||||| ||||| |   ||| ||    | |||  | ||| ||||||||||||||
Sbjct  411108  TTTCTAAATTCCATATATATGCATTAGTATACTGTATTGGTGTTTTTCTTTCTGGCTTAC  411049

Query  7845    TTCACTTGGTATGAT  7859
               ||| ||  |||| ||
Sbjct  411048  TTCGCTCTGTATAAT  411034
```

Range 14: 99876 to 99920

Score	Expect	Identities	Gaps	Strand	Frame
41.9 bits(45)	0.004()	36/45(80%)	0/45(0%)	Plus/Plus	

```
Query  8976   AAGCTGTGTGACCTTGGATAAGTCACTGACCGTCTCTGAGCCTCA  9020
              |||||||||||| |||| || |   |||||| ||||||  |||||
Sbjct  99876  AAGCTGTGTGACTTTGGTTACGAGGCTGACCCTCTCTGGTCCTCA  99920
```

Range 15: 116860 to 116904

Score	Expect	Identities	Gaps	Strand	Frame
41.9 bits(45)	0.004()	36/45(80%)	0/45(0%)	Plus/Minus	

```
Query  8975    CAAGCTGTGTGACCTTGGATAAGTCACTGACCGTCTCTGAGCCTC  9019
               || ||||||| ||||| |  ||| || | ||| ||||||||||||
Sbjct  116904  CATGCTGTGTAACCTTAGGAAAGCCATTCACCTTCTCTGAGCCTC  116860
```

Range 16: 86129 to 86167

Score	Expect	Identities	Gaps	Strand	Frame
40.1 bits(43)	0.014()	32/39(82%)	0/39(0%)	Plus/Minus	

```
Query  8978   GCTGTGTGACCTTGGATAAGTCACTGACCGTCTCTGAGC  9016
              |||| ||||||||||| ||| |||| | | |||||| ||
```

```
Sbjct  86167  GCTGCGTGACCTTGGAAAAGCCACTCAACCTCTCTGGGC  86129
```

Range 17: 77526 to 77580

Score	Expect	Identities	Gaps	Strand	Frame
38.3 bits(41)	0.048()	42/55(76%)	1/55(1%)	Plus/Plus	

```
Query  10451  CTGTGTGACCTTGTGCA-GTTACTTACCCTTTCTGTGCCTCAGTTTCCTTGTCTG  10504
              ||| ||||| |||  || || |||   ||| ||| ||||||||||||||  ||||
Sbjct  77526  CTGAGTGACTTTGGACAAGTGACTATACCTCTCTCTGCCTCAGTTTCCTCATCTG  77580
```

Range 18: 77915 to 77972

Score	Expect	Identities	Gaps	Strand	Frame
38.3 bits(41)	0.048()	43/58(74%)	0/58(0%)	Plus/Plus	

```
Query  11808  ACAGTTGATAAAACCAGCCCAGAGAGGGGCGGTGACTTGCCTAGGGTTACACAGCTAG  11865
              |||| ||| ||||| ||||||||||||    || || | || ||||| || | || ||
Sbjct  77915  ACAGATGAGAAAACTAGCCCAGAGAGGTTAAGTAACCTTCCCAGGGTCACTCCGCAAG  77972
```

Range 19: 320184 to 320251

Score	Expect	Identities	Gaps	Strand	Frame
37.4 bits(40)	0.048()	50/68(74%)	4/68(5%)	Plus/Plus	

```
Query  7450    ttttttaat--ttttattttttAA--TTGAAGTATAGTCAGTTCACAGTGTTGTGTAAAT  7505
               ||||| |||  | | |||||||||  ||| ||| || |   ||||||||||||||    |
Sbjct  320184  TTTTTAAATGGTGTAATTTTTTAAAATTGGAGTGTAATTGTTTCACAGTGTTGTGCTGGT  320243

Query  7506    TTCTGGTG  7513
               ||||| ||
Sbjct  320244  TTCTGCTG  320251
```

Taxonomy

Reports

- **Lineage**
- **Organism**
- **Taxonomy**

Dot Plot

Plot of lcl|Query_27483 vs lcl|Query_27485

Top
