# Supplementary material for: Molecular evolution of PCSK family: Analysis of natural selection rate and gene loss
Source: PLoS One. 2021 Oct 28;16(10):e0259085. doi: 10.1371/journal.pone.0259085 (PMC8553125; doi:10.1371/journal.pone.0259085)
Supplement: S7 File — Regions indicating changes in coding sequence or frame are highlighted (if applicable). (PDF) [file pone.0259085.s013.pdf]

COVID-19 Information

Public health information (CDC) | Research information (NIH)

SARS-CoV-2 data (NCBI) | Prevention and treatment information (HHS) | Español

BLAST ® » blastn suite-2sequences » results for RID-J9P4CZNK114

Job Title	Nucleotide Sequence ...
RID	J9P4CZNK114 Search expires on 08-25 19:31 pm
Program	Blast 2 sequences
Query ID	lcl|Query_18275 (dna)
Query Descr	None ...
Query Length	20287
Subject ID	lcl|Query_18277 (dna)
Subject Descr	ref|NW_015504362.1|:531972-545368_Miniopterus_natalensis_isolate_MN2012-01_unplaced_genomic_scaffold_Mnat.v1_scaff262_whole_genome_shotgun_sequence ...
Subject Length	13397

Descriptions

Description	Scientific Name	Max Score	Total Score	Query Cover	E value	Per. Ident	Acc. Len	Accession
ref|NW_015504362.1|:531972-545368_Miniopterus_natalensis_isolate_MN2012-01_unplaced_genomic_scaffold_Mnat.v1_scaff262_whole_genome_shotgun_sequence		49.1	127	0%	9e-07	87.18%	13397	Query_18277

»

Graphic Summary

Distribution of the top 3 Blast Hits on 1 subject sequences

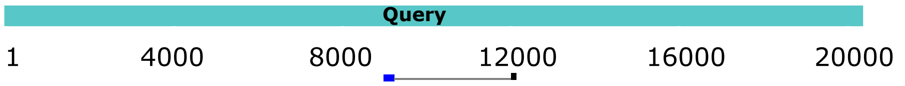

Alignments

Alignment view Pairwise ☐ CDS feature Restore defaults

ref|NW_015504362.1|:531972-545368_Miniopterus_natalensis_isolate_MN2012-01_unplaced_genomic_scaffold_Mnat.v1_scaff262_whole_genome_shotgun_sequence

Sequence ID: Query_18277 Length: 13397 Number of Matches: 3

Range 1: 5335 to 5373

Score	Expect	Identities	Gaps	Strand	Frame
49.1 bits(53)	9e-07()	34/39(87%)	0/39(0%)	Plus/Plus	

```
Query  8980  TGTGTGACCTTGGATAAGTCACTGACCGTCTCTGAGCCT  9018
             |||||||||||| | ||||||||| || ||||||| |||
Sbjct  5335  TGTGTGACCTTGTACAAGTCACTGCCCCTCTCTGATCCT  5373
```

Range 2: 7338 to 7385

Score	Expect	Identities	Gaps	Strand	Frame
42.8 bits(46)	4e-05()	38/48(79%)	0/48(0%)	Plus/Minus	

```
Query  8981  GTGTGACCTTGGATAAGTCACTGACCGTCTCTGAGCCTCAGGTTCCTC  9028
             ||||||||||||  |||| |||| || ||| | ||| |||| || |||
Sbjct  7385  GTGTGACCTTGGGCAAGTTACTGCCCCTCTTTAAGCATCAGTTTACTC  7338
```

Range 3: 5335 to 5368

Score	Expect	Identities	Gaps	Strand	Frame
35.6 bits(38)	0.005()	28/34(82%)	0/34(0%)	Plus/Minus	

```
Query  11827  CAGAGAGGGGCGGTGACTTGCCTAGGGTTACACA  11860
              ||||||||||| ||||||||   | ||| |||||
Sbjct  5368   CAGAGAGGGGCAGTGACTTGTACAAGGTCACACA  5335
```

Taxonomy

Reports

- **Lineage**
- **Organism**
- **Taxonomy**

Dot Plot

Plot of lcl|Query_18275 vs lcl|Query_18277

Top
