# Supplementary material for: Molecular evolution of PCSK family: Analysis of natural selection rate and gene loss
Source: PLoS One. 2021 Oct 28;16(10):e0259085. doi: 10.1371/journal.pone.0259085 (PMC8553125; doi:10.1371/journal.pone.0259085)
Supplement: S8 File — Regions indicating changes in coding sequence or frame are highlighted (if applicable). (PDF) [file pone.0259085.s014.pdf]

COVID-19 Information

Public health information (CDC) | Research information (NIH)

SARS-CoV-2 data (NCBI) | Prevention and treatment information (HHS) | Español

BLAST ® » blastn suite-2sequences » results for RID-H2NP90NX114

**Job Title**	Nucleotide Sequence ...
**RID**	H2NP90NX114 Search expires on 08-11 00:24 am
**Program**	Blast 2 sequences
**Query ID**	lcl|Query_33869 (dna)
**Query Descr**	None ...
**Query Length**	20287
**Subject ID**	lcl|Query_33871 (dna)
**Subject Descr**	ref|NW_004569172.1|:6740699-7071122 Mustela putorius furo isolate ID#1420 breed Sable unplaced genomic scaffold, MusPutFur1.0 scaffold00031, whole genome shotgun sequence ...
**Subject Length**	330424

Descriptions

Description	Scientific Name	Max Score	Total Score	Query Cover	E value	Per. Ident	Acc. Len	Accession
ref|NW_004569172.1|:6740699-7071122 Mustela putorius furo isolate ID#1420 breed Sable unplaced genomic scaffold, MusPutFur1.0 scaffold00031, whole genome shotgun sequence		283	1327	14%	6e-76	70.00%	330424	Query_33871

»
Graphic Summary

Distribution of the top 15 Blast Hits on 1 subject sequences

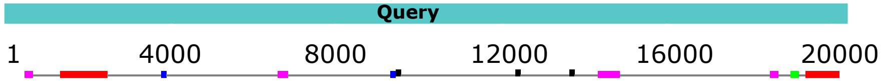

Alignments

Alignment view Pairwise

☐ CDS feature

Restore defaults

ref|NW_004569172.1|:6740699-7071122 Mustela putorius furo isolate ID#1420 breed Sable unplaced genomic scaffold, MusPutFur1.0 scaffold00031, whole genome shotgun sequence

Sequence ID: Query_33871 Length: 330424 Number of Matches: 15

Range 1: 88651 to 88777

Score	Expect	Identities	Gaps	Strand	Frame
93.3 bits(102)	6e-19()	106/136(78%)	10/136(7%)	Plus/Plus	

```
Query  482    GACCCCAGGTCCCGGCGCGCGCCTAGAGCTCCCCACAGCGAGGCACAGTGGCGGCCGGCC  541
              ||||| || ||||| | ||||    | |||| |||| || ||||||||    ||| |||
Sbjct  88651  GACCCGAG-TCCCG-CTCGCGGACGGTGCTCTCCACCGCAAGGCACAG----GGCAGGC-  88703

Query  542    TTGGCCAGCGCGCTGCCCCCGGGTCTCCCCGCC-GAGCGCAAACCTTTCCTCTCCCCGCG  600
               ||| | ||||||  |||| |||| |||||||| | |||||||||| |||||||||||||
Sbjct  88704  -TGGACCGCGCGCCACCCC-GGGTGTCCCCGCCTGGGCGCAAACCTCTCCTCTCCCCGCG  88761

Query  601    ATGGGCGCGGACAGCT  616
              |||||||| | |||||
Sbjct  88762  ATGGGCGCCGGCAGCT  88777
```

Range 2: 89336 to 90263

Score	Expect	Identities	Gaps	Strand	Frame
226 bits(250)	4e-59()	706/1078(65%)	189/1078(17%)	Plus/Plus	

```
Query  1260   gcgg-ggtggggagtgggagtggggatggtcatggggatcttggggaAGGACAGCACTGC  1318
              |||| ||||||  ||| || ||||| |||     |||  ||||| ||  ||| |||| ||
Sbjct  89336  GCGGCGGTGGGCGGTGCGACTGGGGTTGG-----GGG--CTTGGAGAGAGAC-GCACCGC  89387

Query  1319   CGTGGCAGGGGTGGAGTGGGAGGGAAGGCGAATAAT--------GGGACTGGAGGCAATT  1370
               ||||   ||| |||| ||||| || ||||  |           |||   || ||| |||
Sbjct  89388  TGTGGTCCGGGGGGAGAGGGAGAGACGGCGGCTCCCAGGGGCGGGGGTGGGGGGGCGATT  89447

Query  1371   TCTACAGGCCACAAAACT----AGTATTGCATCCTTTTCAGCTGAAGAAAAGAACAGAA-  1425
              ||| || |||||| ||||    ||| ||  ||| |||||||| |||| ||| |||| ||
Sbjct  89448  TCTTCATGCCACAGAACTTTCTAGTTTTCTATCTTTTTCAGCCGAAGGAAACAACAAAAA  89507

Query  1426   CTAAAGGCAAAGGGGCGGAGTTATTCTCAAGGCCCTTTATGG--TCCTTGG--GGTCCTC  1481
              |||||||  |||||||||| || |||| ||| ||| ||| ||  |||||||  || || |
Sbjct  89508  CTAAAGGGGAAGGGGCGGAATTGTTCTGAAG-CCCGTTAGGGGGTCCTTGGCCGGGCCAC  89566

Query  1482   AGGCAAGGAAGGGCTTTGTGGATGCTCATGAGCAGGAGGT-GGGCGCACCTGGTAGCTGG  1540
                |   |||||||||  | || |||||    |||| ||  |||||  ||  ||| ||||
Sbjct  89567  TTG---GGAAGGGCT--GCGGGTGCTCC----CAGGTGGCAGGGCGGGCCAAGTA-CTGG  89616

Query  1541   GA-CAAGGAGGCTGAGCCCTTCAGCCCATGCGCAGGTCCTGCCGGCATAGGCGGGGGTGG  1599
              || |  ||| |||||||||  | |||||    |||  |  ||||   | | |  ||||||
Sbjct  89617  GAACCGGGAAGCTGAGCCCCAC-GCCCA----CAGCGCTCGCCGCGGTCGCCCAGGGTGG  89671

Query  1600   GCAGGGCGAGTTTCCTGAAGATTGATGCCAGCACCTGGCTCTAGGGTTATGGGAGCT-TC  1658
              ||  ||||||||||| ||||    ||||| ||||||||| | |||| ||||||||||   |
Sbjct  89672  GCCTGGCGAGTTTCCCGAAGGCCAATGCCGGCACCTGGCCCCAGGGCTATGGGAGCTCCC  89731

Query  1659   TGCCCAGGGGGACCGCTGG-TCCCTCCAATTATAACCTTCCCAGGACTCGACTGAGGTCC  1717
              ||  | | ||| | ||||| || |||| |||| || ||||||||| || |
Sbjct  89732  TG--CTGAGGGCCGGCTGGTTCTCTCC-ATTACAATCTTCCCAGGCCTTG----------  89778

Query  1718   CAATACAGGACTTGGAGTCAGCCCTGGGGTTGAATCCTGGCTCCATCACCCACTAGCTCT  1777
                     ||  ||||||||||  |||||||||||||| ||||||| | | |||  ||| |
Sbjct  89779  ------GGGCGTTGGAGTCAGAACTGGGGTTGAATCCCGGCTCCAACCCTCACAGGCTGT  89832
```

```
Query  1778   GTGATGCTTGGCTCGTCACTTAACCTCTGAGCCTCCATTTCCTTATCTTCAAAAGGGAGG  1837
              ||| ||||  ||         | |||||| || || |||||||| |||| |||     ||
Sbjct  89833  GTGGTGCTCCGC---------AGCCTCTGGGCTTCTATTTCCTTCTCTTTAAA----GGG  89879

Query  1838   TGACAGTTCTTCCCTAGGGTCTGTTGTGACATTTCAGTGCTGGGCAGATGGAGGAATGAA  1897
               |||||| | ||| |||||   |||||||                     |||||   ||
Sbjct  89880  CGACAGTCCCTCCGTAGGG---GTTGTGA---------------------GAGGA---AA  89912

Query  1898   GGGGAAAGGGCTCTATTGCTCACATGCATGACCTCACCGGGATGTGAGCCAGTGCAGAGA  1957
              ||    ||  ||                ||||||| | |   || ||||||| ||||
Sbjct  89913  GG----AGTTCT---------------GTGACCTC-CTG---TGGGAGCCAGGGCAG---  89946

Query  1958   ACACTGTAGTTATTTCCCTGGCTGCTGTGTGACCTCCCGGTGACATCCTCTTTACTCCAA  2017
                         ||| |  || |||| |  |||||||| |||||| |||||||| |||||
Sbjct  89947  -----------ATTGC--TGTCTGCAG--TGACCTCCTGGTGACTTCCTCTTTGCTCCAC  89991

Query  2018   ACTGCAGCTCCTGGAGCAGAGGGAAAGTTCTA---------GGCTAATAGACACCAGGCC  2068
              ||||||||||||||||||||||| || |||||         ||||||| || ||| || |
Sbjct  89992  ACTGCAGCTCCTGGAGCAGAGGGGAAATTCTACCGCTAATGGGCTAATGGATACCTGGGC  90051

Query  2069   TGC-ACTTCTGCCCCAGCC----CCTCTGCCTAAGTGTGCTAGGGTGG--GGAGGGATGT  2121
               ||  ||||||||||||||    || ||| || ||||||| |||||||  | ||||  ||
Sbjct  90052  AGCGGCTTCTGCCCCAGCCCCAGCCCCTG-CTGAGTGTGCCAGGGTGGGCGAAGGGGCGT  90110

Query  2122   CAGGCCCTTAGTGTTACCTGTGCCTGGTGTCAGTGGTAGTGGGGAGAGACCTCTCTTCTT  2181
                ||||||||| | | |||||||||  | ||               | | |||  |||||
Sbjct  90111  --GGCCCTTAGGGCTGCCTGTGCCT--TTTC-------------TTAAATCTCCTTTCTT  90153

Query  2182   CGGTCTGGGTTTCACAAAAGAGTGACATTTACTTAGCTCAAATCACCCTCTTTCTTGTTC  2241
              | |||| || | | ||         |   |||| ||||| || | ||| ||| |||||||
Sbjct  90154  CAGTCT-GGCTACCCA------CCCCCAGTACTCAGCTCTAAGC-CCCCCTTCCTTGTTC  90205

Query  2242   CCTGAGCCTTTCACCTTCTAGAAGGATGTTG-CTGGGTTGTGGCAAGGATGAGAAAGG  2298
              ||||| ||| |||||||||||||||| ||||    || ||| ||  ||||||||||||
Sbjct  90206  CCTGAACCTCTCACCTTCTAGAAGGACGTTGAGAAGGATGTTGCTGGGATGAGAAAGG  90263
```

Range 3: 70948 to 70989

Score	Expect	Identities	Gaps	Strand	Frame
38.3 bits(41)	0.038()	34/42(81%)	2/42(4%)	Plus/Plus	

```
Query  1801   CCTCTGAGCCTCCA--TTTCCTTATCTTCAAAAGGGAGGTGA  1840
              |||||| | ||||   ||||||||| | | ||||||||||||
Sbjct  70948  CCTCTGGGGCTCCTGCTTTCCTTATATGCCAAAGGGAGGTGA  70989
```

Range 4: 328348 to 328385

Score	Expect	Identities	Gaps	Strand	Frame
42.8 bits(46)	9e-04()	32/38(84%)	0/38(0%)	Plus/Minus	

```
Query  3619    GTCTCAGAGAGGTTGAGTGACTCGCCCGTGGCCACACA  3656
               |||||||||||||| ||||| |||| |   ||||||||
Sbjct  328385  GTCTCAGAGAGGTTCAGTGAGTCGCTCACAGCCACACA  328348
```

Range 5: 92447 to 92482

Score	Expect	Identities	Gaps	Strand	Frame
43.7 bits(47)	9e-04()	31/36(86%)	0/36(0%)	Plus/Plus	

```
Query  3621   CTCAGAGAGGTTGAGTGACTCGCCCGTGGCCACACA  3656
              |||||||||||||| |||||  ||| ||| ||||||
Sbjct  92447  CTCAGAGAGGTTGAATGACTTTCCCATGGTCACACA  92482
```

Range 6: 90320 to 90455

Score	Expect	Identities	Gaps	Strand	Frame
110 bits(121)	7e-24()	108/137(79%)	2/137(1%)	Plus/Plus	

```
Query  6342   AAAT-GGAATGGTCATTTAAGGACTAAATGAGATCGTCAAGTATTTAAGCAGATGCTAAG  6400
              |||| ||||  |||| ||||| |||||  ||||  || |  |||||| |||||  |||||
Sbjct  90320  AAATAGGAACAGTCACTTAAGAACTAAGCGAGAAGGTGACATATTTAGGCAGACACTAAG  90379

Query  6401   CACAGAAACTCACAGAGGTGTGCACAGGTTACGGAAGCCCACGGGAATACTAAGGCACCC  6460
               |||| || ||||||||| ||   ||||||| ||| |||||||||||  || | |||| |
Sbjct  90380  TACAGGAATTCACAGAGGCGTTGGCAGGTTAAGGA-GCCCACGGGAAGCCTGAAGCACGC  90438

Query  6461   AGAGATGAGTTGCTGTG  6477
              |||||||||||||||||
Sbjct  90439  AGAGATGAGTTGCTGTG  90455
```

Range 7: 92437 to 92484

Score	Expect	Identities	Gaps	Strand	Frame
42.8 bits(46)	9e-04()	38/48(79%)	0/48(0%)	Plus/Minus	

```
Query  8978   GCTGTGTGACCTTGGATAAGTCACTGACCGTCTCTGAGCCTCAGGTTC  9025
              ||||||||||| |||  |||||| | | | ||||||||| | || |||
Sbjct  92484  GCTGTGTGACCATGGGAAAGTCATTCAACCTCTCTGAGCTTTAGTTTC  92437
```

Range 8: 72702 to 72744

Score	Expect	Identities	Gaps	Strand	Frame
60.8 bits(66)	3e-09()	39/43(91%)	0/43(0%)	Plus/Plus	

```
Query  8979   CTGTGTGACCTTGGATAAGTCACTGACCGTCTCTGAGCCTCAG  9021
              ||||||||| ||||| ||||||||| || ||||||||||||||
Sbjct  72702  CTGTGTGACTTTGGACAAGTCACTGCCCCTCTCTGAGCCTCAG  72744
```

Range 9: 15911 to 15943

Score	Expect	Identities	Gaps	Strand	Frame
38.3 bits(41)	0.038()	28/33(85%)	0/33(0%)	Plus/Plus	

```
Query  9027   TCTGCAAAAGGGAGGTAATGATAGTTTCTACCT  9059
              |||| | || |  ||||||||||||||||||||
Sbjct  15911  TCTGTACAATGAGGGTAATGATAGTTTCTACCT  15943
```

Range 10: 72702 to 72739

Score	Expect	Identities	Gaps	Strand	Frame

38.3 bits(41)	0.038()	31/38(82%)	0/38(0%)	Plus/Minus	

```
Query  11824  GCCCAGAGAGGGGCGGTGACTTGCCTAGGGTTACACAG  11861
              || ||||||||||| |||||||| | |  || ||||||
Sbjct  72739  GCTCAGAGAGGGGCAGTGACTTGTCCAAAGTCACACAG  72702
```

Range 11: 329366 to 329418

Score	Expect	Identities	Gaps	Strand	Frame
37.4 bits(40)	0.038()	42/54(78%)	2/54(3%)	Plus/Plus	

```
Query  13021   TATTGTGAGGATCATGTAAGTTCCTATATTCAGGCACTTAGAA-GGAGCCTGGC  13073
               |||||||||||| | || ||||   |||| ||||   |||||| |||||| |||
Sbjct  329366  TATTGTGAGGATGAAGTGAGTTAAAATATACAGG-GTTTAGAATGGAGCCAGGC  329418
```

Range 12: 90468 to 90916

Score	Expect	Identities	Gaps	Strand	Frame
85.1 bits(93)	3e-16()	305/467(65%)	44/467(9%)	Plus/Plus	

```
Query  13641  TGTCTCTCAGGCTTGGAGTTTATTCAGAAAAGCCAGCTGGCCCAGCCTGGGGGGCGGTTG  13700
              || |||||| ||| ||||||| ||  | ||||||||||||||  |||    ||  ||  |
Sbjct  90468  TGCCTCTCAAGCT-GGAGTTTCTTTGGGAAAGCCAGCTGGCCTGGCCCACAGGAAGGG-G  90525

Query  13701  GTGGTGCTGCTGCCGCTGGTGGGAGGGTACAGCCGGGCCCTCAACGCCGCCTGCCAGCAC  13760
               ||||   ||||||   |||||| ||||    | || | |||| |   ||| |||||
Sbjct  90526  ATGGTATCGCTGCCCTCGGTGGGTGGGTGTGTCTGGCCTCTCAGCATAGCCCGCCAGTGG  90585

Query  13761  CTGGCGAGGACGGGGGCAGTGCTGGTGGCCGCAGCCGGCAACTTCCGGGACGACGCTTGC  13820
              |||||  || | ||| |  |||  ||||||            ||| |||| ||| | |||
Sbjct  90586  CTGGCAGGGCCTGGGCCCCTGCCTGTGGCC------------TTCTGGGAAGACACCTGC  90633

Query  13821  CTCTACTCCCCAGCCTCGGCTCCCGAGGT--GGGTG------------CTCCAGGA-GTA  13865
              ||| |||| | |||||| | ||||| ||   |||||            | |||| | | |
Sbjct  90634  CTC-ACTCTCTAGCCTCAGGTCCCGGGGCAGGGGTGGACGCCACTACCCCCCAGAATGGA  90692

Query  13866  CGGGAAGGTGG-CAGGTGGGCCC-CTGTGGGCTTCATGGGGTGCACTCC-------TGAA  13916
               |||||||||| ||||   |  | |||||||||||| || ||||  ||||       ||||
Sbjct  90693  GGGGAAGGTGGGCAGGCATGAACACTGTGGGCTTCGTGTGGTGTTCTCCTGGAGGTTGAA  90752

Query  13917  CTAGCCTGGCTTTGCAGGGAGGTGTCAGAGACTCCCAGGGCTGAGCCTGGACAGGGAAAG  13976
              | |||||||||||| |||  |  |||||| || || |     ||||||||    | | | |
Sbjct  90753  C-AGCCTGGCTTTGGAGGATGTCGTCAGACACCCCAACACAGGAGCCTGG-GTGAGGAGG  90810

Query  13977  GGCTTGAACCTTCAGCATTCTCATCTATAAACAGCACCATCCTCAACTCTCTCCCTTCCC  14036
                || |||| | || ||| ||  ||||| ||||||||  |||||| | ||  |||| | |
Sbjct  90811  CACTCGAACTTCCACCATCCTAGTCTATGAACAGCACTGTCCTCAGCACT-ACCCTCCAC  90869

Query  14037  CGCAAAGCAGCCCCGCCCTCACGCCCT-GCCCCTCTCCCT-CTGAAT  14081
                | |   |||| | |||    ||||| | |||||||||| ||||||
Sbjct  90870  TACCACATAGCCACACCCCTGTGCCCTGGTCCCTCTCCCTCCTGAAT  90916
```

Range 13: 92547 to 92691

Score	Expect	Identities	Gaps	Strand	Frame
113 bits(125)	6e-25()	119/155(77%)	10/155(6%)	Plus/Plus	

```
Query  17674  TGACTTATTTCTGGGTTTCCCAGCTCCAGCCCCAGACCCGAAAGAGATGGAGTCTGAATG  17733
              |||||| ||||||| |||  ||||||  ||| ||| ||| |||| ||||||||||||
Sbjct  92547  TGACTTGTTTCTGGATTTTACAGCTCTGGCCTCAGCCCCTAAAGTGATGGAGTCTGA---  92603

Query  17734  GGGTGGGGAGGACAGACAGATGGTCCCACAGCATCCAGGTGTCTGAGCTGGCCCTCCTTT  17793
              ||||||  |||| ||||||| |||||| |||  |||| | |||| |||| || ||||| |
Sbjct  92604  GGGTGGCAAGGAGAGACAGAGGGTCCCCCAGTCTCCAAGGGTCTAAGCT-GCTCTCCTCT  92662

Query  17794  GCCCCAGGCTGCAGCTCCCACTGGGAAGTGGAGGA  17828
              |||||||      |||||||| |||| |  |||||
Sbjct  92663  GCCCCAG------GCTCCCACAGGGAGGCAGAGGA  92691
```

Range 14: 92914 to 93063

Score	Expect	Identities	Gaps	Strand	Frame
72.5 bits(79)	2e-12()	104/150(69%)	14/150(9%)	Plus/Plus	

```
Query  18094  CTCACAGATCTGGGGGGTGGTTTGTGGGCTGGTCGCTGTTGGCGGCTTTTGCAGCTGTGT  18153
              |||| | |||||||  | ||||| ||||  ||  |||    |  ||||||||| |||||
Sbjct  92914  CTCAAATATCTGGGCAGAGGTTTATGGGGAGGATGCTACCTGGAGCTTTTGCATCTGTGG  92973

Query  18154  GGACAGCGTGTGCATGTGTGCTCCTCTGTG--------------GCTGGGCCAGGTTTTG  18199
              |||||| |||||  ||||||    | ||||              |||||||||| |||||
Sbjct  92974  GGACAGTGTGTGTGTGTGTGTGTGTGTGTGTGTGTGTTTGGGTGTGGCTGGGCCAGTTTTTG  93033

Query  18200  CTTTTGTCTAGTTTAGCGAGGTTTGCTCTC  18229
              |||||||| || | || |||||||||| ||
Sbjct  93034  CTTTTGTCCAGATGAGTGAGGTTTGCTGTC  93063
```

Range 15: 93236 to 93958

Score	Expect	Identities	Gaps	Strand	Frame
283 bits(313)	6e-76()	532/760(70%)	73/760(9%)	Plus/Plus	

```
Query  18377  GGAGCAGCTAGCGGGGCCAGAGAGGCAAGGGAGGGTGTGC--AGAGAGGGCGGGAGCCAG  18434
              |||||||| |  | |||| |  |||  |||||||| | ||  ||||||||| ||||   |
Sbjct  93236  GGAGCAGCCAATGTGGCCGGCAAGGTCAGGGAGGGCGAGCTGAGAGAGGGCAGGAGT--G  93293

Query  18435  CTCTCAGAAACCACCCGTGCCAAGTGCAAC--CTGCGGCTTCTCTGTAAGTCTCCTTTTA  18492
              | | ||| || |  | ||||  | ||||||  |   |||| |||| |   |||||||| |
Sbjct  93294  CCCCCAGGAAACCTCAGTGCAGA-TGCAACTCCAATGGCTGCTCTCTCC-TCTCCTTTAA  93351

Query  18493  AAAGCCACAGGGAACTTCTTCAAAGGAAGCCCTGCAGAGTTCACTTTTAAATGAACTG-G  18551
              ||||||||||||| | |||| |||||||||||||    ||   || |||  ||  ||  |
Sbjct  93352  AAAGCCACAGGGAGCCTCTTAAAAGGAAGCCCTGGGCGGTCTGCTCTTATGTGTGCTCAG  93411

Query  18552  AAGAGGTTTTTAAGAGTGTGAGTCTGTGCTGATTGTGTTCTGCATGCTGCATTTCTGGAG  18611
              || |||||||||| ||            | || | || | || |||  ||||||||||||
Sbjct  93412  AAAAGGTTTTTAAAAGCA----------CAGAGTATGGTTTGGATGACGCATTTCTGGAG  93461

Query  18612  GGCAAGGGCTGTTCCAGGTCCACTTGCTCAGCA---AATGT-TGAGGCCTGTGGCATCCC  18667
              ||||||| ||||  ||||||||   |||  |||   | ||  ||||| ||| ||  | ||
Sbjct  93462  GGCAAGGACTGTCTCAGGTCCATAGGCTGGGCACGCATTGAGTGAGGGCTGCGGGGTACC  93521

Query  18668  AGGCAATGTTCCAGGCGGTGGGGATACAAACCCGACTAGCTTTCTCTCCTGGCGCGTCCA  18727
               | || ||| || |||         || ||||| |||     |||  ||||| | |||||
Sbjct  93522  TG-CATTGTCCCCGGC---------ACTAACCCAACT-----TCTTCCCTGGAGTGTCCA  93566

Query  18728  GTCTAATGGGGGAGAAGGACAGCAAACAAATAAGTAACTATAGAGTAATTAAAACATGCT  18787
               | |  ||||||||||   ||| |||||||| | || | ||  ||||| ||||||| || |
Sbjct  93567  TTTTCGTGGGGGAGAA---CAGTAAACAAAAACGTCA-TAGGGAGTACTTAAAAGATACA  93622
```

```
Query  18788  ATAGAGGAAAGTAAAGCAGGGAAGGGAATG------GGAGGGTCCTTCAGGAGAGGCCTC  18841
               | ||| ||| |||||||||||||||||||      ||||||| || |||||||||||||
Sbjct  93623  GTGGAGAAAAATAAAGCAGGGAAGGGAATGTTGGACGGAGGGTGCT-CAGGAGAGGCCTC  93681

Query  18842  CTTGAGAAGGTGGGGGACATCACAGGGAA----CAGTGTTCAAGGCAGAGGGGGTAGCCA  18897
                ||||||||||| || ||||||||||||    |||||||   ||||| |||   |||||
Sbjct  93682  ACTGAGAAGGTGGAGGGCATCACAGGGAAGGAACAGTGTTGT-GGCAGCGGGAACAGCCA  93740

Query  18898  GGGCAAAGGCCCTGA---GGTGGGAGTGGGCTTGGAGAGCAAAAGGAAGAGCCAG-AGGG  18953
              ||| |||| ||||||   ||||||||   |  ||||| |||  ||||  || | | |||
Sbjct  93741  GGGTAAAGACCCTGACGTGGTGGGAGCATGACTGGAGGGCACCAGGAGCAGACGGGAGGC  93800

Query  18954  CTG-GTGAGGTGGGACCCGAGTGG-GAGGGGGAACCAGAGA-----CAGG----GTTTAG  19002
              ||| | |||| || |||| | ||  |  || | || |||||     ||||    | | ||
Sbjct  93801  CTGTGGGAGGGGGAACCC-ATTGAAGCTGGAGGACTAGAGAGGGCACAGGACCAGGTGAG  93859

Query  19003  GTGGGGCCGGAGGGCCACAGGAAGGAC-TTGGATTTTTACTGGAGTGAGCTGGGAGCCAC  19061
              |||||||  |||||||||||   |||| |||||||||  || |||||||||||||||| |
Sbjct  93860  GTGGGGCGTGAGGGCCACAGTCGGGACTTTGGATTTTGCCT-GAGTGAGCTGGGAGCCCC  93918

Query  19062  ACAGGGTTCTGAG-CCTGGGTGTGGGGAGGGGGGTGGGCT  19100
               |||||||||||| ||| |||  |||  |||| ||| |||
Sbjct  93919  GCAGGGTTCTGAGTCCTCGGTCAGGGTGGGGGTGTGTGCT  93958
```

Taxonomy

Reports

- Lineage
- Organism
- Taxonomy

Dot Plot

Plot of lcl|Query_33869 vs lcl|Query_33871
