# Supplementary material for: Molecular evolution of PCSK family: Analysis of natural selection rate and gene loss
Source: PLoS One. 2021 Oct 28;16(10):e0259085. doi: 10.1371/journal.pone.0259085 (PMC8553125; doi:10.1371/journal.pone.0259085)
Supplement: S9 File — Regions indicating changes in coding sequence or frame are highlighted (if applicable). (PDF) [file pone.0259085.s015.pdf]

COVID-19 Information

Public health information (CDC) | Research information (NIH)

SARS-CoV-2 data (NCBI) | Prevention and treatment information (HHS) | Español

BLAST ® » **blastn suite-2sequences** » results for RID-HAHAUXUW11N

Job Title	Nucleotide Sequence ...
RID	HAHAUXUW11N Search expires on 08-13 23:59 pm
Program	Blast 2 sequences
Query ID	lcl|Query_7475 (dna)
Query Descr	None ...
Query Length	20287
Subject ID	lcl|Query_7477 (dna)
Subject Descr	ref|NW_007370660.1|:186955-206584_Eptesicus_fuscus_isolate_BU_THK_EF1_unplaced_genomic_scaffold_EptFus1.0_scaffold00010_whole_genome_shotgun_sequence ...
Subject Length	19630

Descriptions

Description	Scientific Name	Max Score	Total Score	Query Cover	E value	Per. Ident	Acc. Len	Accession
ref|NW_007370660.1|:186955-206584_Eptesicus_fuscus_isolate_BU_THK_EF1_unplaced_genomic_scaffold_EptFus1.0_scaffold00010_whole_genome_shotgun_sequence		242	242	2%	3e-65	70.39%	19630	Query_7477

Graphic Summary

Distribution of the top 1 Blast Hits on 1 subject sequences

Query

1 4000 8000 12000 16000 20000

Alignments

Alignment view Pairwise CDS feature Restore defaults

ref|NW_007370660.1|:186955-206584_Eptesicus_fuscus_isolate_BU_THK_EF1_unplaced_genomic_scaffold_EptFus1.0_scaffold00010_whole_genome_shotgun_sequence

Sequence ID: Query_7477 Length: 19630 Number of Matches: 1

Range 1: 6033 to 6585

Score	Expect	Identities	Gaps	Strand	Frame
242 bits(268)	3e-65()	416/591(70%)	56/591(9%)	Plus/Minus	

```
Query  19713  GCTGGGGTCCCAGGCCTTGGCTGAGCTTTGAAGTGCTTCCTTTTTCCTCCTTCCTCAGCC  19772
              |||| ||||||||| | ||||||||||||||| || |||||  | ||  || |||||||
Sbjct  6585   GCTGTGGTCCCAGGTCCTGGCTGAGCTTTGAAATGGTTCCTCCTCCCCTCTCCCTCAGCT  6526

Query  19773  CTCCTCAGCCTGGGCCCCGGGGGACAGAAGGCACCTCT-TTCTCCTGGAGCTCTGGTGCT  19831
              |||||||||| ||  ||| ||||||||| |  ||||||  | | ||| |||| |||  ||
Sbjct  6525   CTCCTCAGCCCGG--CCCAGGGGACAGAGGATACCTCTACTTTTCTGCAGCTGTGGCTCT  6468
```

```
Query  19832  GGCACTTGGGGTACACTGGCTCCCTGCCTGGGAGAACCCCATCTCTTGGCCCGAGTCA-C  19890
              |||||||| |||     |||||||||  |||||||||   |||||   ||| |  ||| |
Sbjct  6467   GGCACTTGAGGT---GGGGCTCCCTGTGTGGGAGAACTTGATCTC--AGCCTGGATCAGC  6413

Query  19891  CCCTCCCCAGACCCGAGCTGAGTGGGAGGTTGAATG-AGCAGGGCCACAGGCGCCGGCAG  19949
              | ||||||| ||| ||||||||||||||| ||| ||  |||||||  |||| || ||
Sbjct  6412   CTCTCCCCAAACCTGAGCTGAGTGGGAGGCTGAGTGCCGCAGGGCTGCAGGGGCTGGTTT  6353

Query  19950  CCCCTCCCTCACTGAGGGGCT--GTGTCCACATG--TCCATCAACAA---GGGTCTGGCT  20002
              |||||||| || | | || ||   || |   |||  |||  | || |    || |  |||
Sbjct  6352   CCCCTCCCCCA-TCACGGCCTCCCTGCCGGGATGCATCCCACCACTAATGCGGACCAGCT  6294

Query  20003  GTGCTCAGCTCCCTGTCAGCTGCTCCCAAGTTGCCAGTGC-TGTGGGCAGAATTAGCTTT  20061
              |||||||||| |||||||||||||||||||  |||| ||| ||||||| || ||| ||||
Sbjct  6293   GTGCTCAGCTTCCTGTCAGCTGCTCCCAAGAGGCCAATGCTTGTGGGCGGAGTTACCTTT  6234

Query  20062  TGTTGAGTTCTTGCTACATGTCAGCCAGGCAGTCAGTCCTCAGGCCTCCATGAAGGAGGT  20121
                 ||||  |||             | ||          | |||| | ||   | ||||
Sbjct  6233   GACTGAG--CTT-------------CTGG----------TTAGGCTTGCACCGAAGAGGG  6199

Query  20122  GGTAACCCTCCTATGGGGAGGCAAGGAAGCACTTGACGGCTGGGAGAGGCCAAATGTTGG  20181
               || |  ||||   ||  ||||||||| |||||||| ||| || ||     |  |||  |
Sbjct  6198   AGTGATGCTCCCTCGGACAGGCAAGGAGGCACTTGAGGGCAGGAAG----TACCTGTCAG  6143

Query  20182  TCA-GAGGA-----TGTGAAAGGTGGAAATGGCCCCTCACCTCCTGCCCACTCTGGGGAG  20235
              ||| |||||      |||||||||||| |||||||||||||||||||||||| ||| ||
Sbjct  6142   TCAGGAGGAATGAGCGTGAAAGGTGGAGATGGCCCCTCACCTCCTGCCCACTTTGGAGAA  6083

Query  20236  GCCCGGTTGGGCTCCCTGATTATGGAGATGA-GTTTTCCATGCCTCTGGGG  20285
              ||||   ||| ||||||||| |  | || || |||| |||  |||||||||
Sbjct  6082   GCCCCTCTGGTCTCCCTGATCAACG-GAGGATGTTTCCCACACCTCTGGGG  6033
```

Taxonomy

Reports

- **Lineage**
- **Organism**
- **Taxonomy**

Dot Plot

Plot of lcl|Query_7475 vs lcl|Query_7477

Top
