# Supplementary material for: Molecular evolution of PCSK family: Analysis of natural selection rate and gene loss
Source: PLoS One. 2021 Oct 28;16(10):e0259085. doi: 10.1371/journal.pone.0259085 (PMC8553125; doi:10.1371/journal.pone.0259085)
Supplement: S10 File — Regions indicating changes in coding sequence or frame are highlighted (if applicable). (PDF) [file pone.0259085.s016.pdf]

COVID-19 Information

Public health information (CDC) | Research information (NIH)

SARS-CoV-2 data (NCBI) | Prevention and treatment information (HHS) | Español

BLAST ® » blastn suite-2sequences » results for RID-H2V3XGC4114

**Job Title**	Nucleotide Sequence ...
**RID**	H2V3XGC4114 Search expires on 08-11 01:57 am
**Program**	Blast 2 sequences
**Query ID**	lcl|Query_38709 (dna)
**Query Descr**	None ...
**Query Length**	20287
**Subject ID**	lcl|Query_38711 (dna)
**Subject Descr**	ref|NW_004545881.1|:21487817-21505246 Sorex araneus isolate GB8-d unplaced genomic scaffold, SorAra2.0 scaffold00026, whole genome shotgun sequence ...
**Subject Length**	17430

Descriptions

Description	Scientific Name	Max Score	Total Score	Query Cover	E value	Per. Ident	Acc. Len	Accession
ref|NW_004545881.1|:21487817-21505246 Sorex araneus isolate GB8-d unplaced genomic scaffold, SorAra2.0 scaffold00026, whole genome shotgun sequence		53.6	126	0%	3e-08	86.36%	17430	Query_38711

Graphic Summary

Distribution of the top 3 Blast Hits on 1 subject sequences

Query

1 4000 8000 12000 16000 20000

Alignments

Alignment view Pairwise

CDS feature

Restore defaults

ref|NW_004545881.1|:21487817-21505246 Sorex araneus isolate GB8-d unplaced genomic scaffold, SorAra2.0 scaffold00026, whole genome shotgun sequence

Sequence ID: Query_38711 Length: 17430 Number of Matches: 3

Range 1: 13695 to 13742

Score	Expect	Identities	Gaps	Strand	Frame
38.3 bits(41)	0.002()	37/48(77%)	0/48(0%)	Plus/Plus	

```
Query  5698   ACACTGAAACCCAGAGTGGGACAGGGTCTTGCCTGAGGTCACACAGCA  5745
              ||||||   | ||||| | ||||| | |||| |  |||||||||||||
Sbjct  13695  ACACTGGGGCGCAGAGAGAGACAGTGACTTGTCCAAGGTCACACAGCA  13742
```

Range 2: 13698 to 13741

Score	Expect	Identities	Gaps	Strand	Frame
53.6 bits(58)	3e-08()	38/44(86%)	0/44(0%)	Plus/Minus	

```
Query  8978   GCTGTGTGACCTTGGATAAGTCACTGACCGTCTCTGAGCCTCAG  9021
              |||||||||||||||| ||||||||| |  |||||| ||| |||
Sbjct  13741  GCTGTGTGACCTTGGACAAGTCACTGTCTCTCTCTGCGCCCCAG  13698
```

Range 3: 13697 to 13749

Score	Expect	Identities	Gaps	Strand	Frame
34.6 bits(37)	0.024()	40/53(75%)	1/53(1%)	Plus/Minus	

```
Query  10442  CATCACCAGCTGTGTGACCTTGTGC-AGTTACTTACCCTTTCTGTGCCTCAGT  10493
              || |||  ||||||||||||||  | ||| |||  | || |||| ||| ||||
Sbjct  13749  CAGCACTTGCTGTGTGACCTTGGACAAGTCACTGTCTCTCTCTGCGCCCCAGT  13697
```

Taxonomy

Reports

- **Lineage**
- **Organism**
- **Taxonomy**

Dot Plot

Plot of lcl|Query_38709 vs lcl|Query_38711

Top
