# Supplementary material for: Molecular evolution of PCSK family: Analysis of natural selection rate and gene loss
Source: PLoS One. 2021 Oct 28;16(10):e0259085. doi: 10.1371/journal.pone.0259085 (PMC8553125; doi:10.1371/journal.pone.0259085)
Supplement: S11 File — Regions indicating changes in coding sequence or frame are highlighted (if applicable). (PDF) [file pone.0259085.s017.pdf]

COVID-19 Information

Public health information (CDC) | Research information (NIH)

SARS-CoV-2 data (NCBI) | Prevention and treatment information (HHS) | Español

BLAST ® » blastn suite-2sequences » results for RID-HA9FUFRB114

**Job Title**	Nucleotide Sequence ...
**RID**	HA9FUFRB114 Search expires on 08-13 21:45 pm
**Program**	Blast 2 sequences
**Query ID**	lcl|Query_29321 (dna)
**Query Descr**	None ...
**Query Length**	20287
**Subject ID**	lcl|Query_29323 (dna)
**Subject Descr**	ref|NW_024423564.1|:59742916-60061545 Ursus maritimus isolate PB19 unplaced genomic scaffold, ASM1731132v1 scaffold_12, whole genome shotgun sequence ...
**Subject Length**	318630

Descriptions

Description	Scientific Name	Max Score	Total Score	Query Cover	E value	Per. Ident	Acc. Len	Accession
ref|NW_024423564.1|:59742916-60061545 Ursus maritimus isolate PB19 unplaced genomic scaffold, ASM1731132v1 scaffold_12, whole genome shotgun sequence		685	4163	38%	0.0	66.63%	318630	Query_29323

Graphic Summary

Distribution of the top 32 Blast Hits on 1 subject sequences

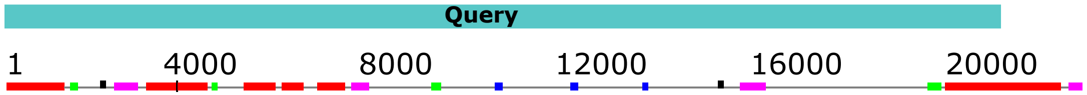

Alignments

Alignment view Pairwise ☐ CDS feature Restore defaults

ref|NW_024423564.1|:59742916-60061545 Ursus maritimus isolate PB19 unplaced genomic scaffold, ASM1731132v1 scaffold_12, whole genome shotgun sequence

Sequence ID: Query_29323 Length: 318630 Number of Matches: 32

Range 1: 236947 to 237967

Score	Expect	Identities	Gaps	Strand	Frame
234 bits(259)	2e-61()	742/1141(65%)	157/1141(13%)	Plus/Minus	

```
Query  29      GTGTCTGTCATCGCACGCAGGGCTCAGGGTGAGGGGCGGAGAGAAGG--CATCTACAGGG  86
               ||||| | || ||| ||||||| ||||| |||||||||| ||| |||  |    | | ||
Sbjct  237967  GTGTCAGGCACCGCTCGCAGGGATCAGGATGAGGGGCGGCGAGGAGGGGCGGTGAGAAGG  237908

Query  87      CACGCCGGGACAGCTTTCCAGCCCAGTTAGCGTTTGGGAtt--tttttCCTCCCTCTGAG  144
               |   |||      | | |||||||| ||||  ||| || ||  | ||| || ||||||||
Sbjct  237907  CTTCCCG------CCTCCCAGCCCAATTAGGATTTAGGGTTGCTATTTACTTCCTCTGAG  237854

Query  145     GGTAATCTGACGTGGTTTGGGAAGGGCGAGGCTGAAACTCGATCCATCAATTCTgggggg  204
                ||||| |||||  ||||| | || || ||||||||||   |||||             |
Sbjct  237853  CGTAATTTGACGCTGTTTGAGGAGAGCAAGGCTGAAACCTAATCCAC------------G  237806

Query  205     tggggggAGCCAGTTAATGTTTAATCAGGTAGGATCATCCGATGGGGCTCGAGTGGCGTG  264
                |||||| | | ||||||||||||||  |||||||| ||| | |||||| | |||||  |
Sbjct  237805  GGGGGGGTGTCCGTTAATGTTTAATCCAGTAGGATCGTCCCAGGGGGCTTGGGTGGCTCG  237746

Query  265     ATCTCCCGGGCCCCGGGCGTCGCGCACCCACACCCCAGCAGGTTTCAGCCTCGGCGTTGA  324
               || || | || || |||||||||||||| | | || || | ||||| |||  | ||  ||
Sbjct  237745  ATTTCGCAGGGCCTGGGCGTCGCGCACCGA-ATCCAAGGAAGTTTCCGCCGGGCCGCGGA  237687

Query  325     GGCGCTCTCGGCTGCAGGCG-GACTC-AGGCTTAGCTCGGGTCCGAGCCCGGGGGAG---  379
               | ||| | | ||||||| || | | |  ||||  ||||| ||| |||||  | | ||
Sbjct  237686  GTCGCGCGCCGCTGCAGACGCGCCGCTGGGCTCTGCTCGAGTCTGAGCCACGAGCAGAGC  237627

Query  380     -GCGAGCCAGACAGTGAGAACTCTCGGGTCCCGTAAGCGTGGCCACGGCGCGGAGCCCCG  438
                | ||||| | ||||||||   |  ||| || | | |||| ||| |  |||| ||||||
Sbjct  237626  AGAGAGCCTGGCAGTGAGACAGCGGGGGCCCGGGACGCGTCGCCGCATCGCGCAGCCCCT  237567

Query  439     AACCCAGAGCCCCAAGGACGGGCCGCGCGGGTGTCCCTGTTGGGACCCCAGGTCCCGGCG  498
               |  || ||||||| |    || ||||||              ||||    |||
Sbjct  237566  A--CCGGAGCCCCGA----GGCCCGCGC------------ACGGAC----GGT-------  237536

Query  499     CGCGCCTAGAGCTCCCCACAGCGAGGCACAGTGGCGGCCGGCCTTGGCCAGCGCG-CTGC  557
```

```
                         ||||||||  || ||||||||  |||| |||||  || | ||||| | ||
Sbjct  237535  ----------GCTCCCCATCGCAAGGCACAGGCGCGGGCGGCC-GGGACCGCGCGCCAGC  237487

Query  558     CCCCGGGTCTCCCCGCC-GAGCGCAAACCTTTCCTCTCCCCGCGATGGGCGCGGACAGCT  616
               ||  | ||| ||| ||| | |||||||     |||||||||||| ||||| |||||||||
Sbjct  237486  CCGGGTGTCCCCCTGCCAGGGCGCAAA-----CCTCTCCCCGCG-TGGGCACGGACAGCT  237433

Query  617     CCTGGCGGCCATGGTGGCCCCCgctgctgctgctgctgctactgctCTTGGGCCCTGGAG  676
                  ||||||| ||                  ||||| ||| |||||| |||||||
Sbjct  237432  GTGGGCGGCCTTG------------------GCTGCCGCTGCTGCTCCTGGGCCC-----  237396

Query  677     GCTCGGGCGTACAGGAGGACGAGGACGGCGACTACGAGGAAATGGTGCTCGCCTTCAGGT  736
                | ||||||  |             | ||       ||||  |||||||| ||||  |||
Sbjct  237395  -CGCGGGCGCTC-------------CCGC-----ATAGGAGCTGGTGCTCCCCTTGCGGT  237355

Query  737     CGGAGGAG-GACGGCCTGACTGACACGACCCAGCACGTGGCCACCGCCAGTTTCCATCGC  795
                |||| ||   | ||| |   ||| || |||||||||||||||||||| | ||||| |||
Sbjct  237354  GGGAGCAGATGCAGCCAG-GAGACGCGGCCCAGCACGTGGCCACCGCCGGCTTCCACCGC  237296

Query  796     TGCGCCAAGGTGCGGGCGC------------CAGGGGCGAA-CCCGC-GTGGGGGCCCCA  841
                ||||||||||||| ||||            | ||||| || ||||| | |||| |||
Sbjct  237295  CGCGCCAAGGTGCGCGCGCCCGAGTGGGAGGCGGGGGCTAACCCCGCAGCGGGGACCCGT  237236

Query  842     GCGGTGGCTGATTCCTCTCCGGCCTCAGTTCTCCCCAGTAAGGGAGAGTCTAGAGAGAAG  901
               | | | || |  ||| |||| |||||||||  |||  ||||||||| | || ||| ||||
Sbjct  237235  GTGCTTGCCG--TCCCCTCCAGCCTCAGTTTCCCCGCGTAAGGGAGGGGCTGGAGTGAAG  237178

Query  902     GTTTCC-AGTGCCTTCTGCTCATCCCAGGACGGGCTTGGCGCAGATCTTGAGGACGGCAG  960
               || ||| || ||| |||||||  |    | | |||| ||||| ||||  | |||| ||||
Sbjct  237177  GTCTCCGAGCGCCCTCTGCTCGCCTGGTGGCCGGCTGGGCGCGGATCCGGGGGACCGCAG  237118

Query  961     GCACTGCGGCAGGGGACCGAGTACAGTAGTTCTTTGGG--------GTGCGCTGTGCTGG  1012
               |    ||||  | |||  |  |  | |  | ||| || |        ||||||||||| |
Sbjct  237117  G---GGCGG--GCGGAAC-CGGCCTGCGGCTCTCTGCGGGCGCGCTGTGCGCTGTGCGGC  237064

Query  1013    GGAAGGCGCACAGGGGTGGGAGACTGGAAGACGTCAGGTAGGGCGAGCAGAGCACCTC-C  1071
                |  || || |  ||||||||| |  |  | ||  |||| |||| |||||||  |  | |
Sbjct  237063  CGGGGGTGCGCCAGGGTGGGAGGC--GGTGGCGCGAGGTCGGGCAAGCAGAGAGCTGCGC  237006

Query  1072    AGGACAGCCTGCGCATATCCCAGACATGCCGCACCACCGAGGCTCTGGTGGGGAAAGGTG  1131
                || | |||||  |   |||||| | ||||||| || || ||||| || |||| |||| |
Sbjct  237005  CGGTC-GCCTG-TCGGGTCCCAGCCGTGCCGCAGCATCGTGGCTCGGGCGGGGGAAGGAG  236948

Query  1132    C  1132
               |
Sbjct  236947  C  236947
```

Range 2: 236780 to 236849

Score	Expect	Identities	Gaps	Strand	Frame
56.3 bits(61)	1e-07()	59/77(77%)	7/77(9%)	Plus/Minus	

```
Query  1264    ggtggggagtgggagtggggatggtcatggggatcttggggaAGGACAGCACTGCCGTGG  1323
               |||||| |||||| ||||||  ||     |||  ||||| ||  ||| ||||||||||||
Sbjct  236849  GGTGGGCAGTGGGCGTGGGGCCGG-----GGG--CTTGGAGAGAGACGGCACTGCCGTGG  236797
```

```
Query  1324    CAGGGGTGGAGTGGGAG  1340
                  ||||||||||||||
Sbjct  236796  TTCGGGTGGAGTGGGAG  236780
```

Range 3: 18900 to 18927

Score	Expect	Identities	Gaps	Strand	Frame
38.3 bits(41)	0.037()	25/28(89%)	0/28(0%)	Plus/Plus	

```
Query  1802   CTCTGAGCCTCCATTTCCTTATCTTCAA  1829
              |||||||||||  |||||| ||||||||
Sbjct  18900  CTCTGAGCCTCGGTTTCCTCATCTTCAA  18927
```

Range 4: 236350 to 236722

Score	Expect	Identities	Gaps	Strand	Frame
177 bits(195)	6e-44()	290/414(70%)	47/414(11%)	Plus/Minus	

```
Query  1986    GTGACCTCCCGGTGACATCCTCTTTACTCCAAACTGCAGCTCCTGGAGCAGA-GGGAAAG  2044
               ||||||||| |||| | ||||| || ||||| ||||||||||||||||| || |||||||
Sbjct  236722  GTGACCTCCTGGTGCCGTCCTCCTTGCTCCACACTGCAGCTCCTGGAGCGGAGGGGAAAG  236663

Query  2045    TTCTA-GGCTAATAGACACCAGGCCTGCACTTCTGCCCCAGCCCCTCTGCCTAAGTGTGC  2103
               |||||   | ||| ||  || | || |||||||||||||||| |||  |||| || ||||
Sbjct  236662  TTCTACCACGAATGGATTCCTGACCCGCACTTCTGCCCCAGCGCCTGGGCCTGAGCGTGC  236603

Query  2104    TAGGGTGG--GGAGGGATGTCAGGCCCTTAGTGTTACCTGTGCCTGGTGTCAGTGGTAGT  2161
                |||||||  ||||||  |    |||||||| ||| ||||  |||  ||    |||
Sbjct  236602  CAGGGTGGGCGGAGGG--GCACAGCCCTTAGGGTTGCCTGAACCTTCTG---CTGG----  236552

Query  2162    GGGGAGAGACCTCTC--TTCTTCGGTCTGGGTTTCACAAAAGAGTGACATTTACTTAGCT  2219
                         |||||  |||||| |||||| |         |  ||||   | || ||||
Sbjct  236551  ----------CTCTCCTTTCTTCCGTCTGGCT-------GCGCCTGAC-CCTCCTCAGCT  236510

Query  2220    CAAATCACCCTCTTTCTTGTTCCCTGAGCCTTTCACCTTCTAGAAGGATGTTGCTGGGTT  2279
               | ||   ||| ||| ||||||| ||||||||||||| ||| |||||||||||||||
Sbjct  236509  CTAA--CCCCGCTTCCTTGTTCTCTGAGCCTTTCACTTTCCAGAAGGATGTTGCTG----  236456

Query  2280    GTGGCAAGGATGAGAAAGGGTGTTTCAAGTCACCACTGTCCCCAAGTAACTATTCTAGGA  2339
                      |||||||||||||  |||||||||||||  |  |||||  | || ||| || |
Sbjct  236455  -------GGATGAGAAAGGGCCTTTCAAGTCACCAGGGCTCCCAA-CAGCTCTTCCAGAA  236404

Query  2340    GTAGTGAGTACTCCATCTTGATAGGTAAGCAGTGACTGGACAACCACCTGAACC  2393
               |||| ||| | |   | | | |  | |||||| | | ||||| |||||||||||
Sbjct  236403  GTAGGGAGCAGTGAGTGTAGGTGAGCAAGCAGAGGCCGGACAGCCACCTGAACC  236350
```

Range 5: 235800 to 236341

Score	Expect	Identities	Gaps	Strand	Frame

444 bits(492)	8e-125()	434/550(79%)	37/550(6%)	Plus/Minus	

```
Query  2553    GAGGGTATTTCTTCCCTGGTGGAAGTTTGAAACAG-ACCCTC--------CAGAAGTTCA  2603
               |||||| ||||| |||| ||| | ||||| ||||| | ||||        |||   ||||
Sbjct  236341  GAGGGTGTTTCTGCCCTCGTGAAGGTTTGGAACAGGAACCTCTGCACGTTCAGCCATTCA  236282

Query  2604    TTGATTCAATGGATATTTTGTGGGGATTGAATTTAGAATGAACAtttttttGGCAGGCAG  2663
               || ||||||||| ||||| ||||| |||||||||||| |||  || ||| ||  ||||||
Sbjct  236281  TTAATTCAATGGGTATTT-GTGGGAATTGAATTTAGAGTGATGATATTTCTGCAAGGCAG  236223

Query  2664    ATAAAGATTTAG-ACCAGTCCTTTTATTTTATT---CATGAGAA------GCCCAGAGAg  2713
               | ||  |||| | || | |||||||||||| ||   ||||||||      ||||||||||
Sbjct  236222  AGAAGAATTTTGGACTAATCCTTTTATTTTTTTACTCATGAGAAACTGAGGCCCAGAGAG  236163

Query  2714    ggggggTCCACCCTCC------TGA--TGCATTAGAACTAGTCTTCCAGGAAAAGTCTCC  2765
                |||||| ||||||||      ||   |||||| |||| ||   || |        ||||
Sbjct  236162  AGGGGGTTCACCCTCCCCCTCCTGCCGTGCATTGGAACCAGAAGTCTACTC-----CTCC  236108

Query  2766    TTCCACTGCACAGAGTGCTCTCCCAATTCATTAGAGTTTCATTTAGTGGAGGG-CATTTT  2824
                ||| ||| ||| ||||| ||||||||| |||||| ||||||||||||||||| ||||||
Sbjct  236107  ATCCTCTGTACAAAGTGCCCTCCCAATTAATTAGATTTTCATTTAGTGGAGGGGCATTTT  236048

Query  2825    AGATGGGCCTTTGAAACATAAATAGGAGTCTAACAAATGAAGGGAACAGGGGAATTTTAT  2884
               ||||||||||||||| ||||||||||||| ||||||||||||||  ||||| | ||||||
Sbjct  236047  AGATGGGCCTTTGAAGCATAAATAGGAGTTTAACAAATGAAGGGGGCAGGGAAGTTTTAT  235988

Query  2885    TCTAGGGGGAGGGGGTAGCATGAACAAAAGCGCAGACCTGGGAAAGCCAGAGATGGAGAA  2944
               |||||||| ||| | |||||||| ||||||| ||||  |||||||||||||| |||||||
Sbjct  235987  TCTAGGGGAAGGAG-TAGCATGAGCAAAAGCACAGAGTTGGGAAAGCCAGAGGTGGAGAA  235929

Query  2945    TGGGAAGCACATGTCCACAGTCCCTTATCCACCTTCTGAAATGTAAAACTGCTCCCCAAA  3004
               | || || ||||||||||||  |||||||  |||||||||||| |||| || |||  |||
Sbjct  235928  TAGGGAGGACATGTCCACAGCTCCTTATCTGCCTTCTGAAATGGAAAA-TGTTCCAAAAA  235870

Query  3005    CCAAAAGGCTTT-TTGTAATTTATTTTGTGGTAACCTGACCTGAACTGACATGAGGTTGT  3063
               |||||||| ||| ||||||||||||| ||||||||||| |||||||||||| ||| || |
Sbjct  235869  CCAAAAGGGTTTCTTGTAATTTATTTGGTGGTAACCTGGCCTGAACTGACACGAGATTTT  235810

Query  3064    TTATAATTTA  3073
               || |  ||||
Sbjct  235809  TTTTTTTTTA  235800
```

Range 6: 234992 to 235598

Score	Expect	Identities	Gaps	Strand	Frame
337 bits(373)	3e-92()	465/639(73%)	60/639(9%)	Plus/Minus	

```
Query  3051    GACATGAGGTTGTTTATAATTTATCCCACTGATATATTCACATTCATATTTATATTAACA  3110
               |||||||| || |||||||||||| |||        || || |   || | |||||  ||
Sbjct  235598  GACATGAGATTATTTATAATTTATTCCA--------TTTACGTGAGTAGTCATATT--CA  235549

Query  3111    GATTTTTTGCTGCATAGATTATAATATGCTGGTCCAGATCCCTCTGAGCGCCCTGACTGC  3170
                || ||||| |||| ||||||||   ||||| ||||||  ||||||||| |||||     
Sbjct  235548  TATATTTTGTTGCAGAGATTATAGCCTGCTGCTCCAGACTCCTCTGAGCACCCTG-----  235494
```

```
Query  3171    CTATTACTACCTTTCTAAAATCCAAATAAGTTACAAATATTGAAACCCATTTGGCCCTAA  3230
               ||     ||||||| |||| ||||||||| |||  ||| |||||||||||||||||||||
Sbjct  235493  CT-----TACCTTTGTAAAGTCCAAATAAATTATGAATCTTGAAACCCATTTGGCCCTAA  235439

Query  3231    GACTTTGGATAAAGGATTGCAGACTCTGTGCTCCTCTCTCTGGTGCGCATACAG-AGATG  3289
               ||||| || |||||| |||  ||| ||   || ||||  |||| || ||    | | ||
Sbjct  235438  GACTTCGG-TAAAGGCTTGTGGACCCTCGTCTACTCTGGCTGGGGCCCACGGTGCACATA  235380

Query  3290    TAGGAGATTAGGCTACAGAGGTAGGTTAGAGAGGGGACCAAGGAGAAGCATGGAGTTTGG  3349
               || || ||  ||     |||||||||| | ||||||| ||||| ||| ||||||||||
Sbjct  235379  TA-GAAATGTGG-----GAGGTAGGTT-GTGGGGGGACC-AGGAGGAGCGTGGAGTTTGG  235328

Query  3350    ACTT----TGTCAGGTTATGGGGAGCCACTGAAGGTTCTTGAGCTCAGGTGTATCTGTTT  3405
               ||||    |||  || | ||||||||| ||||||| || ||||  | ||||| |||||||
Sbjct  235327  ACTTCATCTGTTGGGGTCTGGGGAGCCGCTGAAGG-TCCTGAG--CGGGTGTGTCTGTTT  235271

Query  3406    GAGAGCAGCAGACACAGATAAAAGCTAACTAAGAGCAA-AAAATCTGCTCTGGCAGACCA  3464
               | |||||| |||  |||||   ||||| || |||||||  | ||| || |    ||||||
Sbjct  235270  GCGAGCAGTAGATGCAGATGGGAGCTAGCTGAGAGCAAGCACATCCGCGCCTATAGACCA  235211

Query  3465    -CGACTTGAGTCTTTTCTCCCACTTGAAAAGTGTTGCCTTTGCTCACTCAATCATCCCTT  3523
                 | ||||||||||| |||||| ||| |||||||||| || | |||||||  ||||||||
Sbjct  235210  TGGCCTTGAGTCTTTCCTCCCATTTGGAAAGTGTTGCTTTGGTTCACTCACACATCCCTT  235151

Query  3524    CTGTTTGC-------TAG-AT----------GCTTTACGCAACCACCTTTCCTAGCCTTC  3565
                 ||||||        || ||          || | ||  |||||||| || ||||||||
Sbjct  235150  GGGTTTGCATTTCCACAGCATGCGGCCGGGCGCCTAACAGAACCACCTCTCTTAGCCTTC  235091

Query  3566    CCAGCAGGCCTGTGCCATAGGTATTAC---CCCGACAACATAGAGTTGATGTCTGAGTCT  3622
               |||||||||| ||||||  | ||||||   ||| || | | | |  |  ||| |||||| ||
Sbjct  235090  CCAGCAGGCCGGTGCCAGGGATATTACAGTCCCCACCATACAAGGGAGATATCTGAGGCT  235031

Query  3623    CAGAGAGGTTGAGTGACTCGCCCGTGGCCACACAACCAG  3661
               |||||||||||||||||| |||| |||||||||| ||||
Sbjct  235030  CAGAGAGGTTGAGTGACTTGCCCATGGCCACACAGCCAG  234992
```

Range 7: 291719 to 291753

Score	Expect	Identities	Gaps	Strand	Frame
37.4 bits(40)	0.037()	29/35(83%)	0/35(0%)	Plus/Plus	

```
Query  3622    TCAGAGAGGTTGAGTGACTCGCCCGTGGCCACACA  3656
               ||||||||||| ||| | || |||  |||||||||
Sbjct  291719  TCAGAGAGGTTCAGTAAGTCACCCAAGGCCACACA  291753
```

Range 8: 234943 to 234988

Score	Expect	Identities	Gaps	Strand	Frame
52.7 bits(57)	2e-06()	39/46(85%)	0/46(0%)	Plus/Minus	

```
Query  3700    TGCCTCCAGAGGGGGCCATGGAGGTACTAGAACGGGGAGAAAGTGA  3745
               ||||||  || |||| || ||||||||||||| |||||| ||||||
```

```
Sbjct  234988  TGCCTCTGGAAGGGGTCACGGAGGTACTAGAATGGGGAGGAAGTGA  234943
```

Range 9: 234388 to 234951

Score	Expect	Identities	Gaps	Strand	Frame
355 bits(393)	1e-97()	431/574(75%)	25/574(4%)	Plus/Minus	

```
Query  4277    AGAAAGTGATTGTTTCTAATTGGGGTATGGGGGAGAAGGGTGTAACTAGGAAGGCCTCCA  4336
               || ||||||||  ||||  | ||||| ||||| |||  ||||| || || |||| |||
Sbjct  234951  AGGAAGTGATTTATTCTGGT-GGGGTCTGGGG-AGAGAGGTGTGACCAGAAAGGTCTCTG  234894

Query  4337    GGAGGAGGTGGACTTCTGGCAGGGCCTCCAAGGGTGTCCAGGCCTCAATTAGGCCCACAG  4396
               | ||||||||  |||| | ||||||||||||||||| |||||||    |||||||| |||
Sbjct  234893  GAAGGAGGTGACCTTCAGACAGGGCCTCCAAGGGTGGCCAGGCCAGGGTTAGGCCCGCAG  234834

Query  4397    ACAACCAGGTGCAGGTGCAGAGGAGAACCTCTGTTGACTGTGGCA--GTTCCATTTTTTG  4454
               | | | |||||| |||||||||   | ||  | | || ||   ||  ||||| | |||||
Sbjct  234833  AGAGCTAGGTGC-GGTGCAGAG--AACCCGTTCTCGAGTGGCCCATGGTTCC-TGTTTTG  234778

Query  4455    CCTGACTGCCAAGTTTGAAAGTGT-GTATAAA-----TTAATACTAGTAGTTGGCCTCTG  4508
                |||||  |||| | || |||||| |||||||     ||||| | | |||||| |||||
Sbjct  234777  GCTGACCACCAAATCTGGAAGTGTCGTATAAACTAAGTTAATGCGAATAGTTGCCCTCTA  234718

Query  4509    TGTGGTGTTAGGGGTCCTAATTTGGTAACTTCTGTTTATACCT-CTATACTCGATGGAGT  4567
               |||| | |  ||||||||||||  ||||||||| || | || | ||| |||  || | ||
Sbjct  234717  TGTGTTTTGGGGGGTCCTAATTCAGTAACTTCTCTTGAGACTTCCTAGACTGTATTGTGT  234658

Query  4568    TTCTTTTGCTGTAATTCTAACTTGTAAACAGAGGTGGGCGAGGCACACATAACATTACTA  4627
               ||||||| |||||||||| |  |||     || |||||  ||| ||||||| ||||| ||
Sbjct  234657  TTCTTTTTCTGTAATTCTCATGTGTTTGTGGAAGTGGGTAAGGGACACATAGCATTATTA  234598

Query  4628    TTCtttttttAACGTCATCATGTCA---CTCCTTGCTTGGGGCCAGGACGCCTGGAGGTT  4684
                | ||||||| | |||||| || ||   |||||||||||| ||||||  ||| ||||||
Sbjct  234597  CTATTTTTTTCATGTCATCGTGGCACTCCTCCTTGCTTGGAGCCAGGCTGCCCGGAGGTG  234538

Query  4685    GCCAGGCACCTACATGGTGGTGCTGA---AGGAGACCCACCGCTCGCAGACCGAGCACAC  4741
               ||  |||| || ||||||||||||||   |||||||||| | | ||||||  |||| |||
Sbjct  234537  GCTGGGCATCTTCATGGTGGTGCTGAGGGAGGAGACCCA-CACCCGCAGATGGAGCGCAC  234479

Query  4742    TGCCCGCCGCCTGCAGGCCCGGGCTGCCCGCCGGGGCTACCTCACCAGGATCCTGCACGT  4801
               |||   |||||||| |||||| ||  || || ||||| ||||| || |||||||||| |
Sbjct  234478  TGC---CCGCCTGCGGGCCCGAGCCTCCTGCTGGGGCGACCTCCCCGGGATCCTGCATGC  234422

Query  4802    CTTCCATGACCTCCTCCCTGGCTTCCTGGTGAAG  4835
               ||||||  | ||| ||||||||||||  ||| ||
Sbjct  234421  CTTCCACAAGCTCTTCCCTGGCTTCCCAGTGCAG  234388
```

Range 10: 233988 to 234345

Score	Expect	Identities	Gaps	Strand	Frame
334 bits(370)	1e-91()	292/361(81%)	3/361(0%)	Plus/Minus	

```
Query  4957    AAACATCCATTAAGCACTTACTGAGAGCCCAGCACAGTGGCTCCTGGCCTTCAGTACAGA  5016
               ||||||  ||| ||  |||||||||  || ||||||||| |||||||| ||  ||||||
Sbjct  234345  AAACATTTATTGAGTGCTTACTGAGTACCAAGCACAGTGACTCCTGGCGTTAGGTACAGG  234286

Query  5017    ATGCCCTGTAAGCTTGGCCAGTCCTCAGCGGTACTTCCATCTTCACTTGGAAGATGAGGA  5076
               ||||| |  |||| || |||| | |||  |||||||||||| ||| ||| ||||||||||
Sbjct  234285  ATGCCGTTCAAGCCTGACCAGGCTTCAATGGTACTTCCATCCTCATTTGAAAGATGAGGA  234226

Query  5077    GACCAAGGTTCAGAAGGGACCACCCAGACATCTAGGGGCAGAGCTGGCTTCAAACCCAGT  5136
               ||||||||||||||||||||||| | |||| ||||||||||||||||  ||||| |||||
Sbjct  234225  GACCAAGGTTCAGAAGGGACCACACGGACAGCTAGGGGCAGAGCTGGGATCAAAGCCAGT  234166

Query  5137    GGTGTGTCTGCTAGCTGTCTTCATGCTGATGAACTTGCTGCCTGTGGAAACCCTATAGGG  5196
               ||| || | ||||  |||| |  |||| ||||||||| |||| | || |||| || ||||
Sbjct  234165  GGTCTGCCCGCTA--TGTCCTTGTGCTAATGAACTTGTTGCCCGGGGGAACCTTACAGGG  234108

Query  5197    ACAAGGCCCCATGACATTAGTTGGGCCTGAGTCATTTTATAAAAGCCTGTCTCAAGGATC  5256
               |||||||||    |||| ||| ||||||| |||||||||||  |||  | | | ||| ||
Sbjct  234107  ACAAGGCCCGGCAACATGAGTGGGGCCTGCGTCATTTTATAGGAGCACGACGC-AGGGTC  234049

Query  5257    CAAAATTCCTTTGAAGCTGATGCTATTCAGAAGGTTTCTCCTGTAGGTCAAGGAGGCTCT  5316
               ||||||||||||||||| | ||||||||||||| || |||||| ||| || | |||||||
Sbjct  234048  CAAAATTCCTTTGAAGCGGCTGCTATTCAGAAGTTTCCTCCTGGAGGACAGGTAGGCTCT  233989

Query  5317    T  5317
               |
Sbjct  233988  T  233988
```

Range 11: 233362 to 233835

Score	Expect	Identities	Gaps	Strand	Frame
253 bits(280)	3e-67()	344/475(72%)	15/475(3%)	Plus/Minus	

```
Query  5610    CCCTGTCCTCCTAACTTGATGAGGACACATGGTTCCCATTTTCACACTGATTTTCCATGT  5669
               |||||||||||||||||| |||| || ||  | |||  |||||||||||||| |   | |
Sbjct  233835  CCCTGTCCTCCTAACTTGGTGAGAACGCAAAGCTCCTGTTTTCACACTGATTCTGTGTTT  233776

Query  5670    GCCTAGGGTGTATCACAGCCTCCTTTAGACACTGAAACCCAGAGTGGGACAGGGTCTTGC  5729
               | ||  || |  ||||||||||||  ||||||  | |||||||||||||  |  || |||
Sbjct  233775  GGCTGCGGGGCTTCACAGCCTCCTGCAGACACCAAGACCCAGAGTGGGATCGATTCCTGC  233716

Query  5730    CTGAGGTCACACAGCATAGAACTGGCAGGGCCAGAA-TTGGGCCCAGGGCTTCTTGCTCC  5788
               ||| |||||||||||| ||||| ||| | | ||||| |||||||  || || || || |
Sbjct  233715  CTGCGGTCACACAGCACAGAACAGGCCGAGGCAGAATTTGGGCCGCGGTCTCCTGGCCCT  233656

Query  5789    ACTGCACAACCACTGCATCGTTTAATTCAGCTCAGCACACAGTGGCTGAACA--ACTGGG  5846
                |||||| ||| | |    || ||||||||    ||| |||||||||  |||  || |||
Sbjct  233655  GCTGCACTACCGCCGAGGGGTCTAATTCAGGGTGGCATACAGTGGCTCCACAGTACCGGG  233596

Query  5847    TGTTAAGTCCTGTGGGGACAATGACATGGATTGGACAGTGTCCAATCCCTT-----CA--  5899
                ||||||||||| |||| ||||| || |||| |||||| ||||   |||||     ||
Sbjct  233595  AGTTAAGTCCTGGGGGGGCAATGCCACGGATGGGACAGGGTCCCTGCCCTTGGGGACACT  233536

Query  5900    ---TCTAATAGGGGAAACCTCAAGTTAATG-CTTCCATCAGTCTGCTCACCACACATTTA  5955
                  ||| ||||||| ||  || ||| |||| |  || |||||||||||| ||||||   |
Sbjct  233535  GACTCTGATAGGGG-AAGATCGAGTAAATGCCGCCCGTCAGTCTGCTCATCACACACCGA  233477
```

```
Query  5956    ATCAGCACCTACTGTGTGCTGCAGACTCAAGGATGAACCAGACCCAGCCCTTTCCCTTGA  6015
                 ||||  |||| ||| ||||| |   | |||| | ||| |  ||||||| | |||  ||
Sbjct  233476  GACAGCTACTACCGTGCGCTGCGGGGACGAGGAGGCACCGGGACCAGCCCCTGCCCGCGA  233417

Query  6016    GCTCACAGTTCAGCAGGGGACACTGAGGAGTGATGGGCAGTGCAGTTAACTGGGG  6070
               |||| | |  |||  || ||  ||||| |||| ||| |||||| || ||| ||||
Sbjct  233416  GCTCGCGGCGCAGACGGAGAGCCTGAGAAGTGGTGGCCAGTGCCGTGAACGGGGG  233362
```

Range 12: 251270 to 251330

Score	Expect	Identities	Gaps	Strand	Frame
52.7 bits(57)	2e-06()	48/61(79%)	0/61(0%)	Plus/Plus	

```
Query  5697    GACACTGAAACCCAGAGTGGGACAGGGTCTTGCCTGAGGTCACACAGCATAGAACTGGCA  5756
               ||||||||  | ||||| ||| ||| | |||| |  | |||||||||||  |||||||||
Sbjct  251270  GACACTGAGGCTCAGAGAGGGGCAGTGACTTGTCCAAAGTCACACAGCAAGGAACTGGCA  251329

Query  5757    G  5757
               |
Sbjct  251330  G  251330
```

Range 13: 232627 to 232890

Score	Expect	Identities	Gaps	Strand	Frame
131 bits(144)	2e-30()	198/278(71%)	18/278(6%)	Plus/Minus	

```
Query  6204    AAGAGCTGGGCTTTGGCACCACGCCAGCCTGGCTTCACATCCCAGCTCAGCTTCTCACTA  6263
               |||| ||||||||||||      || |||||| ||||  ||||||||||||  || ||||
Sbjct  232890  AAGAACTGGGCTTTGGCGTTTTACCGGCCTGGGTTCAAGTCCCAGCTCAGCCCCTAACTA  232831

Query  6264    GTTTGTCTAACTGTAGGCAAATTCCTTCACCTCCC--AGTTTC--TCCCCTATCTGTAAT  6319
               || |||  | || | |||| |||| || ||||  |  ||| ||  |||||| ||||  |
Sbjct  232830  GTGTGTG-AGCT-TGGGCACATTCTTTTACCTTGCGGAGTCTCAGTCCCCTGTCTGGAGG  232773

Query  6320    TTGGGTCTAAAAATACAGACCCAAATGGAATGGTCATTTAAGGACTAAATGAGATCGTCA  6379
                ||||  | || |||           ||||  ||||  |||| || | ||||| | || |
Sbjct  232772  CTGGGCATCAACATA-----------GGAACAGTCACGTAAGAACGACATGAGGTGGTGA  232724

Query  6380    AGTATTTAAGCAGATGCTAAGCACAGAAACTCACAGAGGTGTGCACAGGTTACGGAAGCC  6439
               ||||||   |||||||||||| |||||||||||| |||| ||   ||| ||| || ||||
Sbjct  232723  AGTATTCGGGCAGATGCTAAGTACAGAAACTCACGGAGGCGTTGGCAGCTTAAGG-AGCC  232665

Query  6440    CACGGGAATACTAAGGCACCCAGAGATGAGTTGCTGTG  6477
               ||| || |  | ||||||||| |||| |||| ||||||
Sbjct  232664  CACAGGGAGCCGAAGGCACCCGGAGACGAGTCGCTGTG  232627
```

Range 14: 296007 to 296099

Score	Expect	Identities	Gaps	Strand	Frame
53.6 bits(58)	5e-07()	70/96(73%)	6/96(6%)	Plus/Minus	

```
Query  7767    taagttcatttgtgtcttttttttttAGATTCCACATATAAGTGATAGCAT---GGATTT  7823
               || |||||| |||   ||| ||| ||| ||||||||||| ||||| | |||   | | ||
Sbjct  296099  TATGTTCATCTGT---TTTGTTTCTTAAATTCCACATATGAGTGAGATCATATGGTAATT  296043

Query  7824    TTCTTTCTCTTTCTGGCTTACTTCACTTGGTATGAT  7859
                ||||||||   ||| |||| ||| ||| | || ||
Sbjct  296042  GTCTTTCTCAGACTGACTTATTTCGCTTAGCATAAT  296007
```

Range 15: 296926 to 297010

Score	Expect	Identities	Gaps	Strand	Frame
40.1 bits(43)	0.011()	61/85(72%)	3/85(3%)	Plus/Minus	

```
Query  7769    agttcatttgtgtcttttttttttAGATTCCACATATAAG--TGA-TAGCATGGATTTTT  7825
               ||| | ||||| | ||| | | ||||||||||||| ||||   ||  |||  | |||| |
Sbjct  297010  AGTCCGTTTGTCTGTTTGTATGTTAGATTCCACATCTAAGACGGACCAGCCAGTATTTGT  296951

Query  7826    CTTTCTCTTTCTGGCTTACTTCACT  7850
               |||| |   |||| |||| ||| ||
Sbjct  296950  CTTTTTTGATCTGACTTATTTCGCT  296926
```

Range 16: 47472 to 47494

Score	Expect	Identities	Gaps	Strand	Frame
42.8 bits(46)	9e-04()	23/23(100%)	0/23(0%)	Plus/Minus	

```
Query  7789   ttttAGATTCCACATATAAGTGA  7811
              |||||||||||||||||||||||
Sbjct  47494  TTTTAGATTCCACATATAAGTGA  47472
```

Range 17: 298213 to 298265

Score	Expect	Identities	Gaps	Strand	Frame
47.3 bits(51)	7e-05()	42/53(79%)	0/53(0%)	Plus/Minus	

```
Query  8977    AGCTGTGTGACCTTGGATAAGTCACTGACCGTCTCTGAGCCTCAGGTTCCTCT  9029
               ||||  | ||||||||  ||||| ||   | |||||||||||||| |||||||
Sbjct  298265  AGCTAGGAGACCTTGGGCAAGTCGCTTTACCTCTCTGAGCCTCAGTTTCCTCT  298213
```

Range 18: 263555 to 263595

Score	Expect	Identities	Gaps	Strand	Frame
39.2 bits(42)	0.011()	33/41(80%)	0/41(0%)	Plus/Plus	

```
Query  8977    AGCTGTGTGACCTTGGATAAGTCACTGACCGTCTCTGAGCC  9017
               ||||||||||||| ||| ||||  || | | |||||| |||
Sbjct  263555  AGCTGTGTGACCTAGGAAAAGTGGCTCATCCTCTCTGGGCC  263595
```

Range 19: 251274 to 251317

Score	Expect	Identities	Gaps	Strand	Frame
62.6 bits(68)	9e-10()	40/44(91%)	0/44(0%)	Plus/Minus	

```
Query  8978    GCTGTGTGACCTTGGATAAGTCACTGACCGTCTCTGAGCCTCAG  9021
               |||||||||| ||||| ||||||||| || ||||||||||||||
Sbjct  251317  GCTGTGTGACTTTGGACAAGTCACTGCCCCTCTCTGAGCCTCAG  251274
```

Range 20: 234995 to 235038

Score	Expect	Identities	Gaps	Strand	Frame
49.1 bits(53)	2e-05()	37/44(84%)	0/44(0%)	Plus/Plus	

```
Query  8978    GCTGTGTGACCTTGGATAAGTCACTGACCGTCTCTGAGCCTCAG  9021
               |||||||| || |||  |||||||| | | ||||||||||||||
Sbjct  234995  GCTGTGTGGCCATGGGCAAGTCACTCAACCTCTCTGAGCCTCAG  235038
```

Range 21: 20256 to 20284

Score	Expect	Identities	Gaps	Strand	Frame
39.2 bits(42)	0.011()	28/32(88%)	3/32(9%)	Plus/Minus	

```
Query  8997   GTCACTGACCGTCTCTGAGCCTCAGGTTCCTC  9028
              ||||||||||    ||||||||||||||||||
Sbjct  20284  GTCACTGACCC---CTGAGCCTCAGGTTCCTC  20256
```

Range 22: 298209 to 298268

Score	Expect	Identities	Gaps	Strand	Frame
42.8 bits(46)	9e-04()	46/60(77%)	1/60(1%)	Plus/Minus	

```
Query  10446   ACCAGCTGTGTGACCTTGTGCA-GTTACTTACCCTTTCTGTGCCTCAGTTTCCTTGTCTG  10504
               || ||||  | ||||||| ||| ||  |||  ||| |||| |||||||||||||  ||||
Sbjct  298268  ACTAGCTAGGAGACCTTGGGCAAGTCGCTTTACCTCTCTGAGCCTCAGTTTCCTCTTCTG  298209
```

Range 23: 213005 to 213056

Score	Expect	Identities	Gaps	Strand	Frame
41.0 bits(44)	0.003()	43/56(77%)	7/56(12%)	Plus/Minus	

```
Query  10446   ACCAGCTGTGTGACCTTGTG---CAGTTACTTACCCTTTCTGTGCCTCAGTTTCCT  10498
               || |||||||||||   | |   |||| ||||||    ||||||||||||||||||
Sbjct  213056  ACTAGCTGTGTGACACAGGGAGACAGTCACTTAC----TCTGTGCCTCAGTTTCCT  213005
```

Range 24: 256357 to 256412

Score	Expect	Identities	Gaps	Strand	Frame
46.4 bits(50)	7e-05()	45/57(79%)	1/57(1%)	Plus/Minus	

```
Query  10450   GCTGTGTGACCTTGTGCAGTTACTTACCCTTTCTGTGCCTCAGTTTCCTTGTCTGGG  10506
               |||||||  |||||   |||| ||||||||  ||| ||||||||||||  || ||||
Sbjct  256412  GCTGTGTAGCCTTGGCAAGTTTCTTACCCTG-CTGAGCCTCAGTTTCCCCGTGTGGG  256357
```

Range 25: 251279 to 251320

Score	Expect	Identities	Gaps	Strand	Frame
41.0 bits(44)	0.003()	34/42(81%)	0/42(0%)	Plus/Plus	

```
Query  11824   GCCCAGAGAGGGGCGGTGACTTGCCTAGGGTTACACAGCTAG  11865
               || ||||||||||| |||||||| | |  || ||||||| ||
Sbjct  251279  GCTCAGAGAGGGGCAGTGACTTGTCCAAAGTCACACAGCAAG  251320
```

Range 26: 242339 to 242371

Score	Expect	Identities	Gaps	Strand	Frame
39.2 bits(42)	0.011()	29/33(88%)	1/33(3%)	Plus/Plus	

```
Query  13282   GGGCAGGCTGGGAGGCTGGGA-TCTGGCAGGCT  13313
               |||| ||||||||||||||||  |||| |||||
Sbjct  242339  GGGCGGGCTGGGAGGCTGGGAGGCTGGGAGGCT  242371
```

Range 27: 232153 to 232602

Score	Expect	Identities	Gaps	Strand	Frame

146 bits(161)	1e-34()	319/466(68%)	44/466(9%)	Plus/Minus

```
Query  13644   CTCTCAGGCTTGGAGTTTATTCAGAAAAGCCAGCTGGCCCAGCCTGGGGGGCGGTTGGTG  13703
               |||||| || |||||||  ||||||||||||||||||||| ||     ||  ||| |  |
Sbjct  232602  CTCTCAAGCCTGGAGTTCCTTCAGAAAAGCCAGCTGGCCCGGCGCACAGGCAGGTGGACG  232543

Query  13704   GTGCTGCTGCCGCTGGTGGGAGGGTACAGCCGGGCCCTCAACGCCGCCTGCCAGCACCTG  13763
               || |  |||||  ||||||| |||   | | |||| |||||||  ||| ||  |||||||
Sbjct  232542  GTACCACTGCCCTTGGTGGGCGGGGGTATCTGGGCTCTCAACGTTGCCAGCTGGCACCTG  232483

Query  13764   GCGAGGACGGGGGCAGTGCTGGTGGCCGCAGCCGGCAACTTCCGGGACGACGCTTGCCTC  13823
               ||  |||   || | |||| ||||||            |||| |||  ||| | ||||||
Sbjct  232482  GCAGGGAGTAGGCCTGTGCGGGTGGC------------CTTCTGGGCAGACACCTGCCTC  232435

Query  13824   TACTCCCCAGCCTCGGCTCCCGAG------GTGGGTG--------CTCCAGGA-GTACGG  13868
                |||| || || ||||||||| ||      |||| ||        | |||| | | | ||
Sbjct  232434  -ACTCTCCGGCTTCGGCTCCCAAGACAGGTGTGGATGCCACCACCCCCCAGAATGGAGGG  232376

Query  13869   GAAGGT-GGCAGGTG-GGCCCCTGTGGGCTTCATGGGGTGCACTCCTGAA--CT-----A  13919
               |||||| |||||| |    ||| ||||||||| || ||||  |||||| |  ||     |
Sbjct  232375  GAAGGTGGGCAGGAGCAATCCCCGTGGGCTTCCTGTGGTGAGCTCCTGGAGGCTGAGACA  232316

Query  13920   GCCTGGCTTTGCAGGGAGGTGTCAGAGACTCCCAGGGCTG-AGCCTGGACAGGGAAAGGG  13978
               |||||| |||| ||||   ||||||| || |||||  | | |||| || | ||| | ||
Sbjct  232315  GCCTGGATTTGGAGGGCATTGTCAGACAC-CCCAGCACAGAAGCCGGGGC-GGGGAGGGT  232258

Query  13979   CTTGAACCTTCAGCATTCTCATCTATAAACAGCACCATCCTCAACTCTCTCCCT-TCCCC  14037
               ||||||| | || ||| ||  ||||| |||| |||| |||||| | ||  |||| | | |
Sbjct  232257  CTTGAACTTCCACCATCCTAGTCTATGAACAACACCGTCCTCAGCGCTACCCCTCTGCTC  232198

Query  14038   GCAAAGCAGCCCCGCCCTCACGCCCT-GCCCCTCTCCCT-CTGAAT  14081
                || | ||||| | |||    || || |||||||| ||| ||||||
Sbjct  232197  CCACA-CAGCCACACCCCTGTGCACTGGCCCCTCTACCTCCTGAAT  232153
```

Range 28: 231822 to 231999

Score	Expect	Identities	Gaps	Strand	Frame
79.7 bits(87)	1e-14()	135/187(72%)	13/187(6%)	Plus/Minus	

```
Query  17382   GAGGCTTCCTTGTCTCCTGGTGCACCTGCTCCCACCTGACTGGTCCCATGCTGGGGCCCA  17441
               ||||  ||| ||||||||||||| ||||| |||||  | || |||  |  |||| |||
Sbjct  231999  GAGGTGTCCCTGTCTCCTGGTGCCCCTGCACCCACTGGGCTCGTCTGACTCTGGAGCCTG  231940

Query  17442   ACTGCCTGGTGCGAAGGCCTGTGCTACC-CTTCCATCCCTGTGACCCTGG---GTGGGCA  17497
               |||||| |||| ||||||| |  || || ||||| |||| ||||| ||||   ||||
Sbjct  231939  ACTGCCAGGTGTGAAGGCCAGGCCTCCCACTTCCGTCCC-GTGACTCTGGCATGTGG---  231884

Query  17498   CCTCATTGGTCTCAGTCTCAGCTTCTTCCTCCCTAAGAAGAATGACGGTAGTTCCTGCCT  17557
               ||||   | ||||||  || ||  | ||||||||   | |||| |  ||||  |||||||
Sbjct  231883  CCTC--GGCTCTCAGCGTCTGCACCCTCCTCCCT-GCAGGAATCA-AGTAGGACCTGCCT  231828

Query  17558   CAATGGG  17564
               | |||||
Sbjct  231827  C-ATGGG  231822
```

Range 29: 228211 to 230544

Score	Expect	Identities	Gaps	Strand	Frame
685 bits(759)	0.0()	1639/2460(67%)	314/2460(12%)	Plus/Minus	

```
Query  17648   CTCAGGCCAGTGTCTCGTTCCTGCCCTGACTTATTTCTGGGTTTCCCAGCTCCAGCCCCA  17707
               ||||||| ||||||||  |||  || |||||| ||||||| |||||| ||||  ||| ||
Sbjct  230544  CTCAGGCTAGTGTCTC--TCCAACCTTGACTTGTTTCTGGATTTCCCGGCTCTGGCCTCA  230487

Query  17708   GACCCGAAAGAGATGGAGTCTGAATGGGGTGGGGAGGACAGACAGATGGTCCCACAGCAT  17767
               ||||| |||  ||||||||||||    |||||  |||| || |||| |||||| |||| |
Sbjct  230486  GACCCTAAACCGATGGAGTCTGAT---GGTGGCAAGGAGAGCCAGAGGGTCCCCCAGCCT  230430

Query  17768   CCAGGTGTCTGAGCTGGCCCTCCTTTGCCCCAGGCTGCAGCTCCCACTGGGAAGTGG-AG  17826
               ||| ||||||||||   | ||||| || ||||||||||||||||||||| || | || ||
Sbjct  230429  CCAAGTGTCTGAGCCA-CTCTCCTCTGGCCCAGGCTGCAGCTCCCACTGAGAGGCGGGAG  230371

Query  17827   GAATTTGGCACCCATGGGCCACCTGTGCTGAGGCCACGAGGTCAGGCTGATCAGTGTGTG  17886
               ||         ||  ||||| || |   |||  ||||              |||||||||
Sbjct  230370  GA---------CCTCGGGCCCCCAGG--TGACCCCAC--------------CAGTGTGTG  230336

Query  17887   GGCCACGCGGAGGCCAGCGTCCATGCCTCCTGCTGCCACTCGCCAGGTCTGGAGTGCAAA  17946
               |||| || ||||||||| ||||| || |  | | |||    ||| ||         || |
Sbjct  230335  GGCCTCGAGGAGGCCAGGGTCCACGCTTTTTACAGCCCTGTGCCGGG---------CATA  230285

Query  17947   TTCAGGGAGCACGGGATCCCGGGCCCTGCGGAGAAGGTGAGAGGCGTGCTGGGCGGGGGA  18006
                ||||| |||| || | ||||||||||| || ||||| | ||| |  || || |  |||
Sbjct  230284  GTCAGGAAGCATGGAACCCCGGGCCCTGGGG-GAAGGAG-GAGCCC-GCAGGCCCTGGGC  230228

Query  18007   CCGGGACGAGAGCCTGACACCCCAAGCGGTGGCCTGTGTCCCTCCTGTGCCACTTTTCTG  18066
               ||    |||| ||                |||| || |||   |||||||   |||||||
Sbjct  230227  CC----CGAGGGC----------------TGGCGTGCGTCTGCCCTGTGCAGATTTTCTG  230188

Query  18067   TGTCAGCATTTGTGTGCCCACCACACCCTCACAGATCTGGGGGGTGGTTTGTGGGCTGGT  18126
               |||                      |||||||   ||||||| | ||||||||||  |||
Sbjct  230187  TGTGG--------------------CCCTCACGTGTCTGGGGAGAGGTTTGTGGGGAGGT  230148

Query  18127   CGCTGTTGGCGGCTTTTGCAGCTGTGTGGACAGCGTGTGCATGTGTGCTCCTCTGTGGCT  18186
               ||||| |||  |||| |||||||||| ||| ||| ||||  ||| |||     ||| |||
Sbjct  230147  CGCTGCTGGGAGCTTCTGCAGCTGTGGGGATAGCATGTGTGTGTTTGCG----TGTAGCT  230092

Query  18187   GGGCCAGGTTTTGCTTTTGTCTAGTTTAGCGAGGTTTGCTCTCTGGGGCACCCTGCCCCT  18246
               ||||| ||||| ||||||||| || | |||  |||||||| || ||||| | | |||||
Sbjct  230091  GGGCCGGGTTT-GCTTTTGTCCAGATGAGCAGGGTTTGCTGTC-GGGGCCCACGGCCCC-  230035

Query  18247   CCCTTGCAGAGAATATGACAAA-TGTTGCATAAGGAAGATCAGCCCACATGCATTCACTG  18305
                |||||||||||  | | | || |||| ||||| | |   | |  | | ||||  |  ||
Sbjct  230034  -CCTTGCAGAGAGCACGCCCAACTGTTACATAAAGCAATGCGGTACTCGTGCAGGCCTTG  229976

Query  18306   GTTCATCCACTCAGCACATCTGCTGG-----------------GAG--------------  18334
                |    ||| |  ||| | |||||||                 |||
Sbjct  229975  ATGAGCCCATTGGGCAAAGCTGCTGGCGGCGGATCAGGGCCGGGAGCCCCACCGGATTCT  229916

Query  18335   ---------GATGACTCAGCCGTGACCAAGAGGAGGGGACACCTGAGCT---------AG  18376
                        || ||| |||| ||||||  ||||||||||||   |||||         ||
```

```
Sbjct  229915  GGGGACCGAGAAGACCCAGCTGTGACCCGGAGGAGGGGACAAGCGAGCTGAGTGCTGGAG  229856

Query  18377   G-----GAGCAGCTAGCGG---GGCCAGAGAGGCAAGGGAGGGTGTGCAGAGAGGGCGGG  18428
               |     |||||||   ||    |||| |  |||||||| || |  || ||||||||||||
Sbjct  229855  GTGGAGGAGCAGCCGACGACGTGGCCGGCAAGGCAAGGAAGCGCATGGAGAGAGGGCGGG  229796

Query  18429   A--GCCAGCTCTCAGAAA-CCACCCGTGCCAAGTGCAACCTGCGGCTTCTCTGTAAGTCT  18485
                  |||  | | |   || ||  | | ||  |  ||| || || |||   |||    |||
Sbjct  229795  CTCGCCCCCGCCCCCCAAGCCTTCAGCGCAGAC-GCAGCCAGCAGCT---CTGGCC-TCT  229741

Query  18486   CCTTTTAAAAGCCACAGGGAACTTCTTCAAAGGAAGCCCTGCAGAGTTCACTTTTAAATG  18545
               ||||| |||||||||||| | ||||||||||||||||||||   ||    || |||| ||
Sbjct  229740  CCTTTCAAAAGCCACAGGCAGCTTCTTCAAAGGAAGCCCTGGGCAGCCTGCTCTTAAGTG  229681

Query  18546   AACTG-GAAGAGGTTTTTAAGAGTGTGAGTCTGTGCTGATTGTGTTCTGCATGCTGCATT  18604
                |||  | | || | ||||  ||   |||   |    | | ||| |||| |||  |||||
Sbjct  229680  CACTCAGCAAAGATGTTTAGAAGCACGAGCGGG----GTTCGTGGTCTGGATGACGCATT  229625

Query  18605   TCTGGAGGGCAAGGGCTGTTCCAGGTCCACTTGCTCAGCAAA---------TGTTGAG--  18653
               |||||||||| ||| ||||  ||||   |||  |||||  |          ||| |||
Sbjct  229624  TCTGGAGGGCGAGGACTGTCTCAGG---ACTGTCTCAGATAGGTACAGCGCTGTCGAGCG  229568

Query  18654   --GCCTGTGGCATCCCAGGCAATGTTCCAGGCGGTGGGGATACAAACCCGACTAGCT-TT  18710
                 | ||| ||  | || ||||     ||   || || || ||| ||||| || |||| ||
Sbjct  229567  AGGGCTGCGGGGTTCC-GGCATCACCCCCCACGCTGTGGCTACGAACCCCACCAGCTCTT  229509

Query  18711   CTCTCCTGGCGCGTCCAGTCTAATGGGGGAGAAGGACAGCAAACAAATAAGTAACTATAG  18770
               |  |||||| | ||||| | |  |||||||||||||| | ||||||| | | || |||||
Sbjct  229508  CCTTCCTGGAGTGTCCATTTTCGTGGGGGAGAAGGACTGTAAACAAAAATGGAA-TATAG  229450

Query  18771   AGTAATTAAAACATGCTATAGAGGAAAGTAAAGCAGGGAAGGGAATG------GGAGGGT  18824
               || | |||| | ||||||| ||||||| | |||| |||| |||||||      || ||||
Sbjct  229449  AGCACTTAAGAGATGCTATGGAGGAAAATGAAGCCGGGACGGGAATGCTGGGTGGGGGGT  229390

Query  18825   CCTTCAGGAGAGGCCTCCTTGAGAAGGTGGGGGACATCACAGGGAACAGTGTTCAAGGCA  18884
                ||  ||| ||||||||  ||||||||||| || |||||||||||||||||||  || ||
Sbjct  229389  GCTG-AGGCGAGGCCTCACTGAGAAGGTGGAGGGCATCACAGGGAACAGTGTTGCAG-CA  229332

Query  18885   GAGGGGGTAGCCAGGGCAAAGGCCCTGA---GGTGGGAGTGGGCTTGGAGAGCAAAAGGA  18941
               | |||   |||| ||| |||| ||||||   |||||||||| || |||||      ||||
Sbjct  229331  GTGGGAACAGCCGGGGGAAAGACCCTGACGTGGTGGGAGTGTGCCTGGAGGCTGCCAGGA  229272

Query  18942   AGAGCCAGAGGGCTGGTGAGGTGGGACCCGAGTGGG--AGGGGGAACCAGAGA-----CA  18994
                 || |||  |||   ||    |||| |||||||    ||||||| |||||||     ||
Sbjct  229271  GCAGACAGGAGGCCCATGGACGGGGAACCGAGTGAATCAGGGGGA-CCAGAGAGGGGACA  229213

Query  18995   GGGTTT----AGGTGGGGCCGGAGGGCCACAGGAAGGACTTGGATTTTTACTGGAGTGAG  19050
               |||       ||| |||||| |||||||||||   |||||| |  ||||||  |||||||
Sbjct  229212  GGGCCAGGGCAGGCGGGGCCTGAGGGCCACAGTCGGGACTTTGGATTTTACCTGAGTGAG  229153

Query  19051   CTGGGAGCCACACAGGGTTCTGAGCCTGGGTGTGGGGAGGGGGG----------------  19094
               ||||||||| | |||||||||||| |  || |||||| |||| |
Sbjct  229152  CTGGGAGCCCCGCAGGGTTCTGAGTCCAGGGGTGGGGTGGGGTGGGCTGTGCTCAGGGAA  229093

Query  19095   TGGGCTATCTGACCTGGGTGTGAGCAGGTTCATTCTGGTCGCTGTGTCGGGAAGACTGCA  19154
               |||||| || ||||||||||||  |||||||||| ||| |||      ||||||||| ||
Sbjct  229092  TGGGCTGTCCGACCTGGGTGTGGACAGGTTCATTTTGGCCGC-----AGGGAAGACTCCA  229038

Query  19155   GGGGACAGGGCGGAAGCAGGGAGGCCCGCTGTAGACGGGTGGACAGCC----CGGGTG--  19208
               |||| | || | |||||||||||      |||||| | ||||||| ||    ||  ||
```

```
Sbjct  229037  GGGGCGGGACAGAAGCAGGGAG----AGTGTAGATGAGTGGACATCCACTGCGACTGTC  228982

Query  19209   ----CTGGGGGG--------TCCGTCAGGGCGGGAGTGTAGAGGATGCTGGAATCTGAAG  19256
                   ||| ||||        || | |  || |||||||| |    | | |||| | ||||
Sbjct  228981  CCAACTGAGGGGTGGGGCACTCTGCCCAGGTGGGAGTGTGGCACGTACCGGAACCAGAAG  228922

Query  19257   GAGGGG-CTGCACATC--TGATGG------CCTGGATATTGG-GGGAGCAGTGGAGGGGG  19306
               |||||| ||||| | |   | |||       |||| | | || |||||   ||  ||| |
Sbjct  228921  GAGGGGCCTGCAGACCCGGGGTGGGGGGGAGCTGGCTGTGGGCGGGAGGGCTG--GGGTG  228864

Query  19307   CGTCCAAGGGTTTTGCTTTGCTCTCGGACGAATGGCATCGCCCCTGACTGGGATGGGAAG  19366
                 | |||||||| ||||  |||| ||| || |||||   ||| ||| ||||  |||| ||
Sbjct  228863  GTTGCAAGGGTTGTGCTCAGCTC-CGGGCGGATGGCGCTGCCGCTGTCTGGCCTGGGGAG  228805

Query  19367   GGCTGTGAGAGGTCA-AGTGTCGGG-----GAAGTTGAG----GCATTTATGC--GGGCC  19414
               | ||| ||| || || || || |||     |||| || |    || | | | |   ||||
Sbjct  228804  GCCTG-GAGGGGGCACAGGGTTGGGGTGCAGAAGATGGGCATCGCCTGTGTCCTGTGGCC  228746

Query  19415   TGGCTCACAGCGTGC-CGTGCCTTACATGTGCTTTCTTTTGTCCCCGGGCCCTGGCAGGT  19473
               ||||||   | ||||  |||||  |||  ||||| |||||||||| ||| ||  |||| |
Sbjct  228745  TGGCTCCTTGGGTGCTGGTGCCGGACACCTGCTTGCTTTTGTCCCTGGGTCCCTGCAGAT  228686

Query  19474   CACCGTGGCCTGCAAGGAGGGCTGGACGCTGACCGGCTGCGGGGCCCA--------CCCC  19525
               || |  ||||||| || ||||||||| |||||| ||||| |   ||||        ||||
Sbjct  228685  CAGCACGGCCTGCGAGAAGGGCTGGATGCTGACAGGCTGTGCCCCCCACCCCCTGCCCCC  228626

Query  19526   GGGGCCTCCCACACCCTGGGGGCCTATGCAGTGGACAACACGTGTGTGGTGAGGGGCCGG  19585
                ||||||||||||| ||||||  || ||  |||||||||  | ||||||||||| |||||
Sbjct  228625  AGGGCCTCCCACACACTGGGGATCTCTGTGGTGGACAACGTG-GTGTGGTGAGGAGCCGG  228567

Query  19586   GACGTGGGTGTGCGAGGCAGGACGGGTGAGGAGGCCG-CCGTGGCCATTGCCATCTGCTG  19644
               | | | ||||   |||||||| |  |||||||||| |   | |||| |  ||||||||||
Sbjct  228566  G-CATTGGTGCAGGAGGCAGGGCCAGTGAGGAGGCTGTGGGGGGCCGTCACCATCTGCTG  228508

Query  19645   CAGGAGCCGG---TCAGGGGAGCAGGCCTCCCCGGGGACCCAGTGACAGCCCCGCCCAGG  19701
                  ||||| |   ||||||||||||||  ||| |||| ||||||||| | || |||||||
Sbjct  228507  TCAGAGCCAGCCCTCAGGGGAGCAGGCTCCCCAGGGGTCCCAGTGACTGGCCTGCCCAGG  228448

Query  19702   A-TATCTGCGTG-------------------GCTGGGGTCCCAGGCCTTGGCTGAGCTTT  19741
               | | |  | |||                   |||| |||||||||||||||  |||||||
Sbjct  228447  ACTGTACGTGTGGAAGGGGTCAGGGCCTGGAGCTGTGGTCCCAGGCCTTGGGGGAGCTTT  228388

Query  19742   G-AAGTGCTTCCTTTTTCCTCCTTCCTCAGCCCTCCTCAGCCTGGGCCCCGGGGGACAGA  19800
               |  || | || ||  |||| |||||||| |||||||| ||||||||  || ||||||||
Sbjct  228387  GTGAGAG-TTTCTCCTTCCCCCTTCCTCTGCCCTCCTTAGCCTGGGGTCCAGGGGACAGG  228329

Query  19801   AGGCACCTCTTTC-TCCTGGAGCTCTGGTGCTGGCACTTGGGGTACACTGGCTCCCTGCC  19859
                | ||||||| || || ||||||| |||||||||||  ||||||     |||||||||||
Sbjct  228328  GGACACCTCTATCTTCATGGAGCTGTGGTGCTGGCA--TGGGGTGTGTGGGCTCCCTGCC  228271

Query  19860   TGGGAGAACCCCATCTCTTGGCCCGAGTCACCCCTCCCCAGACCCGAGCTGAGTGGGAGG  19919
                 | | |||   | | ||  ||| |  || ||   |||||||| | | ||||| |||||||
Sbjct  228270  CAGAAAAACATGACCACTCAGCCTGGATCGCCTGTCCCCAGAGCTGCGCTGAATGGGAGG  228211
```

Range 30: 264051 to 264080

**Score**	**Expect**	**Identities**	**Gaps**	**Strand**	**Frame**

37.4 bits(40)	0.037()	26/30(87%)	0/30(0%)	Plus/Minus	

```
Query  17755   GGTCCCACAGCATCCAGGTGTCTGAGCTGG  17784
               |||||||||||||| | ||||| ||| |||
Sbjct  264080  GGTCCCACAGCATCTAAGTGTCAGAGTTGG  264051
```

Range 31: 176513 to 176585

Score	Expect	Identities	Gaps	Strand	Frame
38.3 bits(41)	0.037()	56/79(71%)	8/79(10%)	Plus/Minus	

```
Query  19101   ATCTGACCTGGGTGTGAGCAGGTTCATTCTGGTCGCTGTGTCGGGAAG--ACTGCAGGGG  19158
               ||||||| |   | | || |||| || ||||||  |||||| | |||   ||||||
Sbjct  176585  ATCTGACTTACATTTCAGAAGGTCCACTCTGGTTACTGTGTGGAGAATCAACTGCA----  176530

Query  19159   ACAGGGCGGAAGCAGGGAG  19177
                 ||||| |||||||||||
Sbjct  176529  --AGGGCAGAAGCAGGGAG  176513
```

Range 32: 227987 to 228150

Score	Expect	Identities	Gaps	Strand	Frame
85.1 bits(93)	3e-16()	132/185(71%)	23/185(12%)	Plus/Minus	

```
Query  20103   AGGCCTCCATGAAGGAGGTGGTAACCCTCCTATGGGGAGGCAAGGAAGCACTTGACGGCT  20162
               ||| |||| |||||||||  || | |||||  ||||  ||||        ||||| ||||
Sbjct  228150  AGGGCTCCCTGAAGGAGGCAGTCATCCTCCCTTGGGTGGGCA--------CTTGAGGGCT  228099

Query  20163   GGGAGAGGCCAAATGTTGGTCAGAGGATGTGAAAGGTGGAAATGGCCCCTCACCTCCTGC  20222
               ||||||||    | ||      |||  |||||||   ||| ||||  |||||||||||||
Sbjct  228098  GGGAGAGG----AAGT------GAG--TGTGAAACAGGGAGATGGTTCCTCACCTCCTGC  228051

Query  20223   CCACTCTGGGGAGGCCCGGTTGGGCTCCCTGAT-TATGGA-GATGAGTTTTCCATGCCTC  20280
               ||||| |||||| ||||  |||||||| ||| |  || || | | ||||||||||  ||
Sbjct  228050  CCACTTTGGGGAAGCCCCTTTGGGCTCTCTGGTCAATAGAGGGTCAGTTTTCCAT-ACTT  227992

Query  20281   TGGGG  20285
               |||||
Sbjct  227991  TGGGG  227987
```

Taxonomy

Reports

- **Lineage**
- **Organism**
- **Taxonomy**

Dot Plot

Plot of lcl|Query_29321 vs lcl|Query_29323
