# Supplementary material for: Molecular evolution of PCSK family: Analysis of natural selection rate and gene loss
Source: PLoS One. 2021 Oct 28;16(10):e0259085. doi: 10.1371/journal.pone.0259085 (PMC8553125; doi:10.1371/journal.pone.0259085)
Supplement: S12 File — Regions indicating changes in coding sequence or frame are highlighted (if applicable). (PDF) [file pone.0259085.s018.pdf]

COVID-19 Information

Public health information (CDC) | Research information (NIH)

SARS-CoV-2 data (NCBI) | Prevention and treatment information (HHS) | Español

BLAST ® » blastn suite-2sequences » results for RID-HA42JVMA11N

**Job Title**	Nucleotide Sequence ...
**RID**	HA42JVMA11N Search expires on 08-13 20:13 pm
**Program**	Blast 2 sequences
**Query ID**	lcl|Query_18777 (dna)
**Query Descr**	None ...
**Query Length**	20287
**Subject ID**	lcl|Query_18779 (dna)
**Subject Descr**	ref|NW_011888951.1|:1825683-1847668 Pteropus vampyrus isolate Shadow unplaced genomic scaffold, Pvam_2.0 Scaffold170, whole genome shotgun sequence ...
**Subject Length**	21986

Descriptions

Description	Scientific Name	Max Score	Total Score	Query Cover	E value	Per. Ident	Acc. Len	Accession
ref|NW_011888951.1|:1825683-1847668 Pteropus vampyrus isolate Shadow unplaced genomic scaffold, Pvam_2.0 Scaffold170, whole genome shotgun sequence		410	1094	8%	1e-115	75.72%	21986	Query_18779

Graphic Summary

Distribution of the top 10 Blast Hits on 1 subject sequences

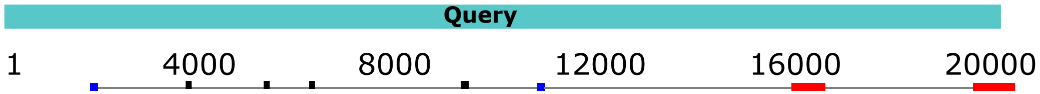

Alignments

Alignment view: Pairwise ☐ CDS feature Restore defaults

ref|NW_011888951.1|:1825683-1847668 Pteropus vampyrus isolate Shadow unplaced genomic scaffold, Pvam_2.0 Scaffold170, whole genome shotgun sequence

Sequence ID: Query_18779 Length: 21986 Number of Matches: 10

Range 1: 5875 to 5954

Score	Expect	Identities	Gaps	Strand	Frame
41.9 bits(45)	2e-04()	57/80(71%)	0/80(0%)	Plus/Minus	

```
Query  1745   GGTTGAATCCTGGCTCCATCACCCACTAGCTCTGTGATGCTTGGCTCGTCACTTAACCTC  1804
              |||| |||||||||||   ||  ||  |||| | |    || |||  || ||||||||||
Sbjct  5954   GGTTTAATCCTGGCTCTGCCAATCAACAGCTGTTTTGACCTGGGCAAGTTACTTAACCTC  5895

Query  1805   TGAGCCTCCATTTCCTTATC  1824
              || |||||   ||||| |||
Sbjct  5894   TGTGCCTCAGGTTCCTCATC  5875
```

Range 2: 7069 to 7098

Score	Expect	Identities	Gaps	Strand	Frame
37.4 bits(40)	0.003()	26/30(87%)	0/30(0%)	Plus/Plus	

```
Query  1802   CTCTGAGCCTCCATTTCCTTATCTTCAAAA  1831
              ||||| || || ||||||||||||| ||||
Sbjct  7069   CTCTGTGCTTCTATTTCCTTATCTTTAAAA  7098
```

Range 3: 16625 to 16655

Score	Expect	Identities	Gaps	Strand	Frame
34.6 bits(37)	0.031()	26/31(84%)	0/31(0%)	Plus/Minus	

```
Query  3615   CTGAGTCTCAGAGAGGTTGAGTGACTCGCCC  3645
              ||||| |||||||||||||| | | | ||||
Sbjct  16655  CTGAGGCTCAGAGAGGTTGAATAATTTGCCC  16625
```

Range 4: 16808 to 16838

Score	Expect	Identities	Gaps	Strand	Frame
35.6 bits(38)	0.009()	27/31(87%)	1/31(3%)	Plus/Minus	

```
Query  5107   TCTAGGGGCAGAGCTGG-CTTCAAACCCAGT  5136
              || ||||||||||||||  ||||||| ||||
Sbjct  16838  TCCAGGGGCAGAGCTGGAGTTCAAACACAGT  16808
```

Range 5: 20085 to 20114

Score	Expect	Identities	Gaps	Strand	Frame
33.7 bits(36)	0.031()	27/32(84%)	2/32(6%)	Plus/Minus	

```
Query  5950   CATTTAATCAGCACCTACTGTGTGCTGCAGAC  5981
              |||||| | |||| |||||||||||  |||||
Sbjct  20114  CATTTACTGAGCATCTACTGTGTGC--CAGAC  20085
```

Range 6: 5878 to 5935

Score	Expect	Identities	Gaps	Strand	Frame
38.3 bits(41)	0.003()	47/61(77%)	4/61(6%)	Plus/Minus	

```
Query  8969  CAATCTCAAGCTGTGT-GACCTTGGATAAGTCACTGACCGTCTCTGAGCCTCAGGTTCCT  9027
             |||||   |||||| | ||||| ||  |||| ||| | |  ||||| |||||||||||||
Sbjct  5935  CAATCAACAGCTGTTTTGACCTGGGC-AAGTTACTTAAC--CTCTGTGCCTCAGGTTCCT  5879

Query  9028  C  9028
             |
Sbjct  5878  C  5878
```

Range 7: 17372 to 17401

Score	Expect	Identities	Gaps	Strand	Frame
46.4 bits(50)	5e-06()	28/30(93%)	0/30(0%)	Plus/Minus	

```
Query  8977   AGCTGTGTGACCTTGGATAAGTCACTGACC  9006
              ||||||||||||||||| ||||||||| ||
Sbjct  17401  AGCTGTGTGACCTTGGACAAGTCACTGCCC  17372
```

Range 8: 5879 to 5933

Score	Expect	Identities	Gaps	Strand	Frame
48.2 bits(52)	1e-06()	46/57(81%)	3/57(5%)	Plus/Minus	

```
Query  10443  ATCACCAGCTGTGT-GACCTTGTGCAGTTACTTACCCTTTCTGTGCCTCAGTTTCCT  10498
              |||| ||||||| | ||||| |   ||||||||| |||  ||||||||||| |||||
Sbjct  5933   ATCAACAGCTGTTTTGACCTGGGCAAGTTACTTAACCT--CTGTGCCTCAGGTTCCT  5879
```

Range 9: 6093 to 6698

Score	Expect	Identities	Gaps	Strand	Frame
410 bits(454)	1e-115()	471/622(76%)	30/622(4%)	Plus/Minus	

```
Query  15545  TTTAACTTCTG-AGGAACAGCCTGgtgtgtctctgtg------catgtgtgtgtgtgtgt  15597
              ||||||||||| | ||| ||| | |||||||||||||      ||||||  | | |||||
Sbjct  6698   TTTAACTTCTGGAAGAATAGCTTAGTGTGTCTCTGTGTGCATACATGTGCATATATGTGT  6639

Query  15598  gtgtgtgcgcgcgcacgcgtgtgtgtACCAAGAGAGGAGTCCCAGATCCGGAAAGAGGGC  15657
              ||||||||           |||||  |||| ||||||||| || | |  | || ||||||
Sbjct  6638   GTGTGTGCA----------TGTGTACACCAGGAGAGGAGTTCCTGGTTGGCAAGGAGGGC  6589

Query  15658  CAGGCCACCACTATCTCTCACTGCCCGTCCCACCACCAGGCATTGTGGCCATGATGCTGA  15717
              ||||||||||  || |||||||| ||   |||||||||||||||||||||||||||||||
Sbjct  6588   CAGGCCACCAGCATTTCTCACTGTCCTCTCCACCACCAGGCATTGTGGCCATGATGCTGA  6529

Query  15718  CGGCCGAGCCGGAGCTCACCCTGGCTGAGCTGAGGCAGAGACTGATCCATTTCTCTGCCA  15777
              | || |||| ||||||||||||||| ||||||||||||||||||| ||| ||||||||||
Sbjct  6528   CTGCAGAGCTGGAGCTCACCCTGGCCGAGCTGAGGCAGAGACTGACCCACTTCTCTGCCA  6469

Query  15778  AAGACGTCATCAACAAGGCCTGGTTTCCCGAAGACCAGCGGGTGCTGACCCCCAACCTGG  15837
              ||| |||| ||||| ||| |||||| || || ||||||||| ||||||||||||||||
Sbjct  6468   AAGGCGTCTTCAACGAGGTCTGGTTCCCTGAGGACCAGCGGCTGCTGACCCCCAACCTCA  6409

Query  15838  TGGCCACACTGCCCCCCAGAACCTATAAAGCAGGTCAGCAGGGCGGCAAGGTGGGCAGAA  15897
              ||||||||||| ||||||| ||  | | | |||||||||||| |||||  |||||||||
Sbjct  6408   TGGCCACACTGTCCCCCAGCACTCAGAGAACAGGTCAGCAGGACGGCAGTGTGGGCAGAG  6349

Query  15898  TCCAGACTGGGGCTTGGGGGGTCTCGGGAGGTCTGTGTGACCTGGGTAGGCTTGTC-CAT  15956
              ||| ||||  |||||||| || || |||||||| ||  || || |  |  ||   | |
Sbjct  6348   TCCGGACT--GGCTTGGGAGGGCTTGGGAGGTCCGTTCGATCTTGCCAATCTCCCCTCTC  6291

Query  15957  CCTCATCTGTGGAGGGAGAATTACACCAGAGGTTCCTAGAAATGGGAGGAGA-TGCAT-A  16014
              |||    |||||| ||||  ||| | ||  |  ||||||||||||  ||||| || || |
Sbjct  6290   CCTGGCTTGTGGAAGGAGGGTTATAGCACTGTCTCCTAGAAATGG--GGAGACTGAATGA  6233

Query  16015  GAAGAGGCTCAGAAAGGGCTTGGCAGGGCGTTCAT--GATGTTTTGAT-GGAAAAATTGA  16071
              ||   |||||  | | ||| | ||    | |||||   ||||||| || | |||||| |
Sbjct  6232   GAGTGGGCTC-CAGACGGCCTTGCTACACTTTCATAACATGTTTTAATAGAAAAAATGGG  6174

Query  16072  TCATGTTCTTTAAGGCTGCTCTCCCTGACCAGGAGCCAAAGGTCTGGCGTCCCCTGTGAG  16131
              ||| ||||||||||     ||||||  | | ||||||||||||| ||| ||   |||| ||
Sbjct  6173   TCACGTTCTTTAAGCACCTTCTCCCCTATCGGGAGCCAAAGGTTTGG-GTTGTCTGTAAG  6115

Query  16132  CAG-AGCCCTGACGGAGGCTCC  16152
```

```
                 ||| | || ||| |||||||||
Sbjct  6114      CAGCACCCTTGATGGAGGCTCC  6093
```

Range 10: 2491 to 3300

Score	Expect	Identities	Gaps	Strand	Frame
368 bits(407)	1e-102()	582/820(71%)	47/820(5%)	Plus/Minus	

```
Query  19144  GGAAGACTGCAGGGGACAGG-GCGGAAGCAGGGAGGCCCGCTGTAGACGGGTGGACAGC-  19201
              ||||||||||||||||||   || |||||| |||| || |||||||||  ||| ||| | 
Sbjct  3300   GGAAGACTGCAGGGGACACAAGCAGAAGCAAGGAGACCTGCTGTAGACAAGTGAACATCT  3241

Query  19202  -CCG----------------GGTGCTGGGGGGTCCGTC-AGGGCGGGAGTGTAGAGGATG  19243
               |||                |||| | |||| || | | |||| |||||||| |||||| 
Sbjct  3240   ACCGTGAGTATCCAGGCCTAGGTGGTTGGGGCTCAGGCCAGGGTGGGAGTGTGGAGGATA  3181

Query  19244  CTGGAATCTGAAGGAGGGGCTG-CACATCTGATGGCCTGGATATTGG-GGGAGCAGTGGA  19301
              ||||||  |||||||   ||   || ||||| ||| |||||  | || ||||| ||| | 
Sbjct  3180   CTGGAACTTGAAGGAACAGCCCACAGATCTGGTGGGCTGGACTTAGGTGGGAGGAGTCGG  3121

Query  19302  GGGGGCGTCCAAGGGTTTTGCTTTGCTCTCGGACGAATGGCATCGCCCCTGACTGGGATG  19361
              ||| | |||||||| |||||||| ||||  ||||  ||||  | |  | |||||||| ||
Sbjct  3120   GGGTGGGTCCAAGGTTTTTGCTTCGCTCCTGGACACATGGTGTTGTAC-TGACTGGGTTG  3062

Query  19362  GGAAGGGCTGTGAGAGGTCAAG-TGTCGGGGAAGTTGAGGCATTTATGCGG-GCCTGGCT  19419
              |||||| |||||  ||||   | ||| ||||||| || ||  || | |    |  ||| |
Sbjct  3061   GGAAGG-CTGTGGAAGGTGTGGGTGTTGGGGAAGATGGGGAGTTCAGGATTAGTATGG-T  3004

Query  19420  CACAGCGTGCCGTGCCTTACATGTGCTTTCTTTTGTCCCCGGGCCCTGGCAGGTCACCGT  19479
              ||    ||||  ||   ||  |||||||||||  ||||| ||||||||||||| ||| | 
Sbjct  3003   CAT---GTGCTATGTACTATGTGTGCTTTCTTCAGTCCCTGGGCCCTGGCAGGGCACTGA  2947

Query  19480  GGCCTGCAAGGAGGGCTGGACGCTGACCGGCTGCGGGGCCCACCCCGGGGCCTCCCACAC  19539
              ||||||||||||||| ||||| |||||| |||| ||||| | ||||| | |||| |||| 
Sbjct  2946   GGCCTGCAAGGAGGGATGGACACTGACCCGCTGTGGGGCTCTCCCCGAGTCCTCTCACAT  2887

Query  19540  CCTGGGGGCCTATGCAGTGGACAACACGTGTGTGGT------GAGGGGCCGGGACGTGGG  19593
               ||||||||| |  ||||||||||||| ||||||||       ||| || ||||| | ||
Sbjct  2886   TCTGGGGGCCCACACAGTGGACAACACATGTGTGGTAAGGAGCAGGAGCTGGGACATTGG  2827

Query  19594  TGTGCGAGGCAGGACGGGTGAGGAGGCCGCCGTGGCCATTGCCATCTGCTGCAGGAGCCG  19653
              ||   ||||||||| | |||||||||||   |  ||||   | ||||||| | | || ||
Sbjct  2826   TGCAGGAGGCAGGATGAGTGAGGAGGCCATAGCAGCCACATCTATCTGCTCCCGAAGTCG  2767

Query  19654  G---TCAGGGGAGCAGGCCTCCCCGGGGACCCAGTGACAGCCCCGCCCAGGATATCTGCG  19710
              |   |||||| |||||| | ||| || |  ||||||||||| || || ||||  | ||| 
Sbjct  2766   GCTTTCAGGGAAGCAGGACACCCAGGAGCTCCAGTGACAGCGCCCCCAAGGACCTATGCA  2707

Query  19711  TGGCTGGGGTCCCAGGCCTTGGCTGAGCTTTGAAGTGCTTCCTTTTTCCTCCTTCCTCAG  19770
              |||     |      || | ||| |  |||||| | || |||||  || |    ||||||| 
Sbjct  2706   TGGAGAAAGGATTGGGGCCTGGAT--GCTTTGCAATGGTTCCTCCTTACCTGTTCCTCAC  2649

Query  19771  CCCTCCTCAGCCTGGGCCCCGGGGGACAGAAGGCACCTCTTTC-TCCTGGAGCTCTGGTG  19829
              ||| ||||||||||||| || || | | ||||  |  |||  | | ||| |||| |||||
Sbjct  2648   CCCGCCTCAGCCTGGGCTCCAGGAGGCGGAAGAAATGTCTACCTTTCTGCAGCTGTGGTG  2589
```

```
Query  19830  CTGGCACTTGGGGTACA---CTGGCTCCCTGCCTGGGAGAACCCCATCTCTTGGCCCGAG  19886
              ||||||||  ||  | |   | ||||||||||||| |||||||  |||||   ||| |
Sbjct  2588   CTGGCACTCTGGCAAAAGGGCAGGCTCCCTGCCTGAGAGAACCTGATCTC--AGCCTGGA  2531

Query  19887  TCACCCCTCCCCAGACCCGAGCTGAGTGGGAGGTTGAATG  19926
              |||  ||  || ||||| ||||||| ||||||| ||| ||
Sbjct  2530   TCATTCCCTCCTAGACCTGAGCTGAATGGGAGGCTGAGTG  2491
```

Taxonomy

Reports

- **Lineage**
- **Organism**
- **Taxonomy**

Dot Plot

Plot of lcl|Query_18777 vs lcl|Query_18779

Top
