# Supplementary material for: Molecular evolution of PCSK family: Analysis of natural selection rate and gene loss
Source: PLoS One. 2021 Oct 28;16(10):e0259085. doi: 10.1371/journal.pone.0259085 (PMC8553125; doi:10.1371/journal.pone.0259085)
Supplement: S13 File — Regions indicating changes in coding sequence or frame are highlighted (if applicable). (PDF) [file pone.0259085.s019.pdf]

COVID-19 Information

Public health information (CDC) | Research information (NIH)

SARS-CoV-2 data (NCBI) | Prevention and treatment information (HHS) | Español

BLAST ® » blastn suite-2sequences » results for RID-HA9KUP0B114

**Job Title**	Nucleotide Sequence ...
**RID**	HA9KUP0B114 Search expires on 08-13 21:47 pm
**Program**	Blast 2 sequences
**Query ID**	lcl|Query_29865 (dna)
**Query Descr**	None ...
**Query Length**	20287
**Subject ID**	lcl|Query_29867 (dna)
**Subject Descr**	ref|NW_006384712.1|:256720-586508 Leptonychotes weddellii isolate WS11-02 unplaced genomic scaffold, LepWed1.0 scaffold01672, whole genome shotgun sequence ...
**Subject Length**	329789

Descriptions

Description	Scientific Name	Max Score	Total Score	Query Cover	E value	Per. Ident	Acc. Len	Accession
ref|NW_006384712.1|:256720-586508 Leptonychotes weddellii isolate WS11-02 unplaced genomic scaffold, LepWed1.0 scaffold01672, whole genome shotgun sequence		557	3775	37%	2e-158	69.01%	329789	Query_29867

Graphic Summary

Distribution of the top 24 Blast Hits on 1 subject sequences

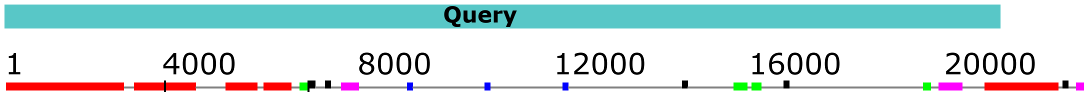

Alignments

Alignment view Pairwise CDS feature Restore defaults

ref|NW_006384712.1|:256720-586508 Leptonychotes weddellii isolate WS11-02 unplaced genomic scaffold, LepWed1.0 scaffold01672, whole genome shotgun sequence

Sequence ID: Query_29867 Length: 329789 Number of Matches: 24

Range 1: 92592 to 94668

Score	Expect	Identities	Gaps	Strand	Frame
499 bits(552)	4e-141()	1538/2359(65%)	339/2359(14%)	Plus/Plus	

```
Query  46     CAGGGCTCAGGGTG------AGGGGCGGAGAGAAGGCATCTACAGGGCACGCCGGGACAG  99
              |||||||||||| |      ||| |||| ||||||||  ||  ||||| ||   || |
Sbjct  92592  CAGGGCTCAGGGCGGAGAGGAGGTGCGGGGAGAAGGCTCCTCGAGGGCGCG---GGCCCA  92648

Query  100    CTTTCCAGCCCAGTTAGCGTTTGGGAtttttttCCTCCCTCTGAGGGTAATCTGACGTGG  159
              | |||||||||||||||  |||||| || ||| ||| |||||||| | |||||||||  |
Sbjct  92649  CCTTCCAGCCCAGTTAGGATTTGGGGTTGTTT-CCTTCCTCTGAGCGCAATCTGACGCTG  92707

Query  160    TTTGGGAAGGGCGAGGCTGAAACTCGATCCATCAATTCTggggggtggggggAGCCAGTT  219
              | |||| | | ||||||||||||  ||||| || | ||  |||| |||||||  || |||
Sbjct  92708  TCTGGGGAAG-CGAGGCTGAAACCTGATCC-TCCAGTCGTGGGGCTGGGGGG--CCCGTT  92763

Query  220    AATGTTTAATCAGGTAGGATCATCCGATGGGGCTCGAGTGGCGTGATCTCCCGGGCCCCG  279
              |||||||||||  |||||||| ||| | | |||||  | |||  ||||||||||| ||||
Sbjct  92764  AATGTTTAATCGAGTAGGATCGTCCCACGCGGCTCCGGGGGCTCGATCTCCCGGGGCCCG  92823

Query  280    GGCGTCGCGCACCCACACCCCAGCAGGTTTCAGCCTCGGCGTTGAGGCGCTCTCGGCTGC  339
              |||||||||||||||  |||||| | ||||| ||   | ||| |||  || | | |||||
Sbjct  92824  GGCGTCGCGCACCCA-GCCCCAGAAAGTTTCCGCGCGGCCGTGGAGTTGCGCGCCGCTGC  92882

Query  340    AGGCGGACTC----AGGCTTAGCT-CGGGTCCGAGCCCGGGGGAGGCGAGCCAGACAGTG  394
              || ||| | |     ||||  ||| ||||||||||||   |||||  ||| |||| || |
Sbjct  92883  AGACGGGCGCCGCTCGGCTCTGCTCCGGGTCCGAGCC---GGGAGCGGAG-CAGAGAGCG  92938

Query  395    AGAACTCTCGGGTCCCGTAAGCGTGGCCACGGCGCGGAGCCCCGAACCCAGAGCCCCAAG  454
               | ||   |||| ||||          || |||||  |||||| |  || | |||||
Sbjct  92939  -GGACAGCCGGG-CCCG----------CATGGCGC--AGCCCCCA--CCGGGGCCCC---  92979

Query  455    GACGGGCCGCGCGGGTGTCCCTGTTGGGACCCCAGGTCCCGGCGCGCGCCTAGAGCTCCC  514
                            | |||||                    |||| ||    | ||||||
Sbjct  92980  --------------GAGTCCC--------------------GCGCACGGACGGTGCTCCC  93005

Query  515    CACAGCGAGGCACAGTGGCGGCCGGCCTTGGCCAGCGCGCTGCCCCCGGGTCTCCCCGCC  574
```

```
              | | || ||||||||  |||| ||||| ||| | ||||||    ||||||| ||||||||
Sbjct  93006  CGCCGCAAGGCACAGGCGCGGGCGGCC-TGGACCGCGCGC-CAGCCCGGGTGTCCCCGCC  93063

Query  575    -GAGCGCAAACCTTTCCTCTCCCCGCGATGGGCGCGGACAGCTCCTGGCGGCCATGGTGG  633
               | |||||||||| |||| |||||||||||||||| | ||||| |  ||||||   |||
Sbjct  93064  AGGGCGCAAACCTCTCCTGTCCCCGCGATGGGCGCTGGCAGCTGCGCGCGGCC---GTGA  93120

Query  634    CCCCCgctgctgctgctgctgctactgctCTTGGGCCCTGGAGGCTCGGGCGTACAGGAG  693
              |  | ||||||| |||| | |     | ||     ||  ||  ||||  |||  ||||
Sbjct  93121  CGGCGGCTGCTGGTGCTCCGG-----GGTC-----CCGAGGGCGCTCCCGCG--CAGGGC  93168

Query  694    GACGAGGACGGCGACTACGAGGAAATGGTGCTCGCCTTCAGGTCGGAGGAGG-ACGGCCT  752
               |||||  ||||    ||| |||  ||| ||||||||    |||| || ||| || |||
Sbjct  93169  CACGAGCGCGGCCCGGACG-GGAGGTGGCGCTCGCCTCGCCGTCGCAGCAGGAACAGCC-  93226

Query  753    GACTGACACGACCCAGCACGTGGCCACCGCCAGTTTCCATCGCTGCGCCAAGGTGC----  808
                |  || || | | |||||||||||||||| | |  |  ||||||||||||||||
Sbjct  93227  -GCCCACCCGGCTCCGCACGTGGCCACCGCCTGCTCGCGCCGCTGCGCCAAGGTGCGCGT  93285

Query  809    --------GGGCGCCAGGGGCGAACCCGC-GTGGGGGCCCCAGCGGTGGCTGATTCCTCT  859
                      ||| | | |||||  |||||| | |||| |||  || || || |   |||||
Sbjct  93286  GCCCGAGGGGGAGGCGGGGGCCGACCCGCAGCGGGGACCCGTGCAGTTGCCGTCCCCTCT  93345

Query  860    CCGGCCTCAGTTCTCCCCAGTAAGGGAGAGTCTAGAGAGAAGGTTTCC-AGTGCCTTCTG  918
              ||||||||||||  |||   ||  |||| | || ||| |||||| ||| || ||| || |
Sbjct  93346  CCGGCCTCAGTTGCCCCGCATACCGGAGGGGCTGGAGCGAAGGTCTCCGAGCGCCCTCCG  93405

Query  919    CTCATCCCAGGACGGGCTTGGCGCAGATCTTGAGGACGGCAGGCACTGCGGCAGGGGACC  978
              |||  |    |||||||| ||||| |||     |  |||  || || ||||  | ||| |
Sbjct  93406  CTCGCCGGGTGACGGGCTGGGCGCGGAT-----GCCCGG--GGGACCGCGG--GCGGAAC  93456

Query  979    GAGTACAGTAGTTCTTTG-GGGTGCGCTGTGC----TG----GGGAAGGCGCACAGGGGT  1029
                | || |  |   || | ||| |||| ||||    ||      | ||| || |  || |
Sbjct  93457  CGGGACGGCGGCCGTTCGCGGGCGCGCCGTGCACCGTGCAGCCCGGAGGTGCGCCAGGCT  93516

Query  1030   GGGAGACTGGAAGACGTCAGGTAGGGCGAGCAGAGCACCTCCAGGACAGCCTGCGCATAT  1089
              ||||| |  | || ||  |||| | ||||||||||| |  || ||   ||| |  |
Sbjct  93517  GGGAGGC--GGAGGCGCGAGGTCGCGCGAGCAGAGCGCTGCCGGGGTCGCC-GGTCGGGG  93573

Query  1090   CCCAGACATGCCGCACCACCGAGGCTCTGGTGGGGAAAGGTGCTAAAGCCTGGACCCCGC  1149
              | ||| |  |||||| ||||| ||||| || |||  |||| ||    | |||     |||
Sbjct  93574  CTCAGCCGCGCCGCAGCACCGTGGCTC-GGCGGGTGAAGGAGC----GGCTG-----CGC  93623

Query  1150   TTAGAACGcccccccccAACCCCTGCACAGAGGAAACAGACTTGCTATTATTATGCATCC  1209
                | | ||||||   ||   ||   ||| ||||     | | |||| |   | ||| | |
Sbjct  93624  GGACAGCGCCCCAAACCGCGCCGCACACGGAGGCCGTCGCCCTGCTGTCGCTGTGCGTTC  93683

Query  1210   TGAAGTGGAtgggggaaatctgggcagtgtagttgtattgtggggagtgtgcggggtggg  1269
              |||   || |  ||||  || ||||   | ||       ||||| |    ||||||||||
Sbjct  93684  TGACCCGGCT-CGGGACCTCCGGGCCCCGGAG-------GTGGGCA----GCGGGGTGGG  93731

Query  1270   gagtgggagtggggatggtcatggggatcttggggaAGGACAGCACTGCCGTGG-CAGG-  1327
              ||       |||||              ||||| ||  ||| |||||| || || | ||
Sbjct  93732  GA-------TGGGG-------------GCTTGGAGAGAGACGGCACTGTCGCGGTCCGGG  93771

Query  1328   -GGTGGAGTGGGAGGGAAGGCGAAT-AATGGGACTGGAGGCAATTTCTACAGGCCACAAA  1385
               || ||||||| || || ||||  | |  ||| | || |||||||||| |  |||||| |
Sbjct  93772  CGGAGGAGTGGCAGAGACGGCGGCTCACAGGGGCGGGGGGCAATTTCTTCGAGCCACAGA  93831

Query  1386   A--------CTAGTATTGCATCCTTTTCAGCTGAAGAAAAGAACAGAACTAAAGGCAAAG  1437
```

```
              |        ||||||| ||| | |||||||| ||   |||||||| ||| |||||  |||
Sbjct  93832  ACTCATATTCTAGTATGGCACCTTTTTCAGCCGAGCGAAAGAACAAAACGAAAGGGGAAG  93891

Query  1438   GGGCGGAGTTATTCT-CAAGGCCCTTTATGGTCCTTGGGGTCCTCAGGC--AAGGAAGGG  1494
               ||||||||| ||||  ||| ||      |||||||||   ||  || |    |||||||
Sbjct  93892  AGGCGGAGTTGTTCTGGAAGCCCGCAGGGGGTCCTTGG---CCCAAGTCCTTGGGAAGGG  93948

Query  1495   CTTTGTGGATGCTCATGAGCAGGAGGTGGGCGCACCTGGTAGCTGGGACAAGGAGGCTGA  1554
              |  || || |||||| | || | |||  ||||| || |||||| || || ||||||||||
Sbjct  93949  C--TGCGGGTGCTCACGGGCGGCAGG-CGGCGCGCCAGGTAGCGGGAACCAGGAGGCTGA  94005

Query  1555   GCCCTTCAGCCCATGCGCAGGTCCTGCCGGCATAGGCGGGGGTGGGCAGGGCGAGTTTCC  1614
              ||||  | |||   ||||| |  | || | |   | |  |||||| |  ||||| |||||
Sbjct  94006  GCCCCAC-GCCGCAGCGCACG--CCGCGGTC--CGCCCAGGGTGGCCCTGGCGACTTTCC  94060

Query  1615   TGAAGATTGATGCCAGCACCTGGCTCTAGGGTTATGGGAGCT-TCTGCCCAGGGGGACCG  1673
               |||| |   |||||||||||||| |   || ||||||||||  |||| |||||   |||
Sbjct  94061  CGAAGGTCAGTGCCAGCACCTGGCCCCTTGGCTATGGGAGCTCCCTGCTCAGGG---CCG  94117

Query  1674   -CTGG-TCCCTCCAATTATAACCTTCCCAGGACT--CGACTGAGGTCCCAAT-ACAGGAC  1728
               |||| ||  ||| |||| |||||||||||| ||   ||||  ||||||  |  |  |
Sbjct  94118  CCTGGTTCTTTCCGATTACAACCTTCCCAGGCCTTGGGACT--GGTCCCGTTGCCCCGGG  94175

Query  1729   TTGGAGTCAGCCCTGGGGTTGAATCCTGGCTCCATCACCCACTAGCTCTGTGATGCTTGG  1788
              ||||||||||  |||||||||||||||||||||||| | |||  |   |||| ||||
Sbjct  94176  TTGGAGTCAGAACTGGGGTTGAATCCTGGCTCCATCGCTCACAGGGGTTGTGGTGCT---  94232

Query  1789   CTCGTCACTTAACCTCTGAGCCTCCATTTCCTTATCTTCAAAAGGGAGGTGACAGTTCTT  1848
                     | ||||||||| || ||||||||||  |||| ||| ||||||||||||| |||
Sbjct  94233  ------CCATAACCTCTGGGCTTCCATTTCCTCCTCTTTAAAGGGGAGGTGACAGTCCTT  94286

Query  1849   CCCTAGGGTCTGTTGTGACATTTCAGTGCTGGGCAGATGGAGGAATGAAGGGGAAAGGGC  1908
              ||||||||  |||||||                               | ||||||||
Sbjct  94287  CCCTAGGGCTTGTTGTG-------------------------------AAGGGAAAGGAG  94315

Query  1909   TCTATTGCTCACATGCATGACCTCACCGGGATGTGAGCCAGTGCAGAGAACACTGTAGTT  1968
              | | |||          ||||  | |||   |||||| ||| |||||||
Sbjct  94316  T-TCTTG----------TGACAGCCCCG---TGTGAGGCAGGGCAGAGA-----------  94350

Query  1969   ATTTCCCTGGCTGCTGTGTGACCTCCCGGTGACATCCTCTTTACTCCAAACTGCAGCTCC  2028
                    ||||||||   ||||||| || ||||||| ||||| |||||| |||||||||||
Sbjct  94351  -----GCTGGCTGC--AGTGACCT-CCTGTGACATACTCTTCACTCCACACTGCAGCTCC  94402

Query  2029   TGGAGCAGAGGGAAAGTTCTAGGCTAATAGACACCAGGCCTGCACTTCTGCCCCAGCCCC  2088
              |||||||                ||||| || ||| || | |  | ||||||||||| ||
Sbjct  94403  TGGAGCA----------------CTAATGGATACCTGG-CGGGGCATCTGCCCCAGCACC  94445

Query  2089   TCTGCCTAAGTGTGCTAGGGTGGGGAGGGATGTCAGGCCCTTAGTGTTACCTGTGCCTGG  2148
                 |||  ||||||| ||||||||  |||      ||||||||| ||| |||||||||
Sbjct  94446  CGGGCCCGAGTGTGCCAGGGTGGGCGGGGGCACTGGGCCCTTAGGGTTGCCTGTGCCTTC  94505

Query  2149   TGTCAGTGGTAGTGGGGAGAGACCTCTCTTCTTCGGTCTGGGTTTCACAAAAGAGTGACA  2208
              ||| |||                |||  |||||| |||||| || | |          |
Sbjct  94506  TGTTAGT---------------TCTCCTTTCTTCAGTCTGGCTTACCC---------GCC  94541

Query  2209   TTTACTTAGCTCAAATCACCCTCTTTCTTGTTCCCTGAGCCTTTCACCTTCTAGAAGGAT  2268
                  |  ||||  || | ||| ||| ||||||||||||| |||||||||||||||||||
Sbjct  94542  CCCCCCCAGCTGTAAGC-CCCCCTTCCTTGTTCCCTGAGGCTTTCACCTTCTAGAAGGAC  94600

Query  2269   GTTGCTGGGTTGTGGCAAGGATGAGAAAGGGTGTTTCAAGTCACCACTGTCCCCAAGTAA  2328
```

```
              |||||||           ||||||||||||||  || |||||||||||||| ||||||   ||
Sbjct  94601  GTTGCTG-----------GGATGAGAAAGGGCATTGCAAGTCACCACTGCCCCCAAACAA  94649

Query  2329   CTATTCTAGGAGTAGTGAG  2347
              || ||| || |||||||||
Sbjct  94650  CTCTTCCAGAAGTAGTGAG  94668
```

Range 2: 16487 to 16526

Score	Expect	Identities	Gaps	Strand	Frame
42.8 bits(46)	9e-04()	34/40(85%)	1/40(2%)	Plus/Plus	

```
Query  1793   TCACTTAACCTCT-GAGCCTCCATTTCCTTATCTTCAAAA  1831
              ||||||||||||| |||| ||| | ||||| |||| ||||
Sbjct  16487  TCACTTAACCTCTGGAGCATCCGTATCCTTGTCTTTAAAA  16526
```

Range 3: 94702 to 95225

Score	Expect	Identities	Gaps	Strand	Frame
488 bits(540)	7e-138()	432/532(81%)	26/532(4%)	Plus/Plus	

```
Query  2560   TTTCTTCCCTGGTGGAAGTTTGAAACAG-ACCCTCCAGAAGTTCA--------TTGATTC  2610
              ||||| |||||||||| ||||| ||||| | ||||   |||||||        || ||||
Sbjct  94702  TTTCTGCCCTGGTGGAGGTTTGGAACAGGAACCTCTGAAAGTTCAGTTATTCATTAATTC  94761

Query  2611   AATGGATATTTTGTGGGGATTGAATTTAGAATGAACAtttttttGGCAGGCAGATAAAGA  2670
              ||||||||||| ||||| |||||||||||| |||   | |||||| |||||||| || ||
Sbjct  94762  AATGGATATTT-GTGGGAATTGAATTTAGAGTGATGGTATTTTTGCCAGGCAGAGAAGGA  94820

Query  2671   TTTAG-ACCAGTCCTTTTATTTTATTCATGAGAAGC------CCAGAGAgggggggTCCA  2723
              ||| | || | ||||||||||||| || ||||||||      |||||||| |||||| ||
Sbjct  94821  TTTTGGACTAATCCTTTTATTTTACTCCTGAGAAGCTGAGGCCCAGAGAGAGGGGGTTCA  94880

Query  2724   CCCTCCTGATGCATTAGAACTAGTCTTCCAGGAAAAGTCTCCTTCCACTGCACAGAGTGC  2783
               || |||| || ||| |||| ||  ||| |       ||||| ||||||||||| |||||
Sbjct  94881  TCCCCCTGGTGTATTGGAACCAGAATTCTACT-----TCTCCATCCACTGCACAAAGTGC  94935

Query  2784   TCTCCCAATTCATTAGAGTTTCATTTAGTGGAGGG-CATTTTAGATGGGCCTTTGAAACA  2842
               |||||||||| ||||| ||||||||||||||||| ||||||||||||||||||||| ||
Sbjct  94936  CCTCCCAATTCGTTAGATTTTCATTTAGTGGAGGGGCATTTTAGATGGGCCTTTGAAGCA  94995

Query  2843   TAAATAGGAGTCTAACAAATGAAGGGAACAGGGGAATTTTATTCTAGGGGGAGGGGGTAG  2902
              ||||||||||| ||| |||| |||| | |||| || |||||||||||||| ||||| |||
Sbjct  94996  TAAATAGGAGTTTAATAAATAAAGGTAGCAGGAGAGTTTTATTCTAGGGGAAGGGG-TAG  95054

Query  2903   CATGAACAAAAGCGCAGACCTGGGAAAGCCAGAGATGGAGAATGGGAAGCACATGTCCAC  2962
               |||||||||||| |||| ||||||| ||||||| |||||||| |||||| |||||||||
Sbjct  95055  TATGAACAAAAGCACAGAGCTGGGAAGGCCAGAGGTGGAGAATAGGAAGCGCATGTCCAC  95114

Query  2963   AGTCCCTTATCCACCTTCTGAAATGTAAAACTGCTCCCCAAACCAAAAGGCTTT-TTGTA  3021
              ||  ||||||   |||||||||||| |||| || |||  ||||||||||| ||| |||||
Sbjct  95115  AGCTCCTTATTTGCCTTCTGAAATGGAAAA-TGTTCCAAAAACCAAAAGGTTTTCTTGTA  95173
```

```
Query  3022   ATTTATTTTGTGGTAACCTGACCTGAACTGACATGAGGTTGTTTATAATTTA  3073
              || ||||| |||||||||||||||||||||||||||  || ||| |  ||||
Sbjct  95174  ATCTATTTGGTGGTAACCTGACCTGAACTGACATGAAATTATTTTTTTTTTA  95225
```

Range 4: 95418 to 96020

Score	Expect	Identities	Gaps	Strand	Frame
327 bits(362)	2e-89()	463/646(72%)	80/646(12%)	Plus/Plus	

```
Query  3051   GACATGAGGTTGTTTATAATTTATCCCACTGATATATTCACATTCATATTTATATTAACA  3110
              |||||||| || |||||||||||| | | |  ||| |    ||||||||| ||||
Sbjct  95418  GACATGAGATTATTTATAATTTATTCTATT--TATGTGAGTATTCATATTCATATA----  95471

Query  3111   GATTTTTTGCTGCATAGATTATAATATGCTGGTCC-AGATCCCTCTGAGCGCCCTGACTG  3169
                  ||||| ||||||||||| | ||||||| ||| || ||| ||||||| || ||| ||
Sbjct  95472  ----TTTTGTTGCATAGATTACAGTATGCTGCTCCCAGCTCC-TCTGAGCACCTTGATTG  95526

Query  3170   CCTATTACTACCTTTCTAAAATCCAAATAAGTTACAAATATTGAAACCCATTTGGCCCTA  3229
              || |||||||||||| |||| ||||||||| |||  ||| || |||||||||||||||||
Sbjct  95527  CCCATTACTACCTTTGTAAAGTCCAAATAAATTATGAATCTTAAAACCCATTTGGCCCTA  95586

Query  3230   AGACTTTGGATAAAGGATTGCAGACTCTGTGCTCCTCTCTCTGG---------TGCGCAT  3280
              |  |||| | |||||||||   ||| ||   || ||||  ||||         ||| |||
Sbjct  95587  A--CTTTAGGTAAAGGATTATGGACCCTACTCTACTCTGGCTGGGGCCCAAGGTGCACAT  95644

Query  3281   ACAGAGATGTAGGAGATTAGGCTACAGAGGTAGGTTAGAGAGGGGACCAAGGAGAAGCAT  3340
              | ||| |||| |||| ||||| |      || |||      || ||||||| || |||||
Sbjct  95645  ATAGAAATGTGGGAGGTTAGGTT------GT-GGT------GGAGACCAAG-AGGAGCAT  95690

Query  3341   GGAGTTTGGACTTTGTCA----GGTTATGGGGAGCCACTGAAGGTTCTTGAGCTCAGGTG  3396
              |||||||| ||||| |||    || | ||  |||||||||||||||| ||| |  |||||
Sbjct  95691  GGAGTTTGAACTTTATCATTTAGGGTCTGAAGAGCCACTGAAGGTTCGTGAAC--AGGTG  95748

Query  3397   TATCTGTTTGAGAGCAGCAGACACAGATAAAAGCTAACTAAGAGCAAAAAATCTGCTCTG  3456
              | ||||||||||||||| |||  ||||||| ||| |       |||      |  || ||
Sbjct  95749  TGTCTGTTTGAGAGCAGTAGAAGCAGATAAGAGCAA-------GCA------CGCCTGTG  95795

Query  3457   GC---AGACCACGACTTGAGTCTTTTCTCCCACTTGAAAAGTGTTGCCTTTGCTCACTCA  3513
               |   |||||  | | |  ||||||||||||| ||||||| |||||| || | |||||||
Sbjct  95796  CCTATAGACCGTGGCCTT-GTCTTTTCTCCCATTTGAAAATTGTTGCTTTAGTTCACTCA  95854

Query  3514   ATCATCCCTTCTGTTTGC-----TAGA------------TGCTTTACGCAACCACCTTTC  3556
                ||||||||  | ||||      |||            ||| | || ||||||||| ||
Sbjct  95855  CGCATCCCTTGGGCTTGCATTTGCAGAGCATGAGGCCGGTGCGTAACACAACCACCTCTC  95914

Query  3557   CTAGCCTTCCCAGCAGGCCTGTGCCATAGGTATTAC---CCCGACAACATAGAGTTGATG  3613
               ||||||||||||||||||||||||| || ||||||    || || | | | ||  |||
Sbjct  95915  TTAGCCTTCCCAGCAGGCCTGTGCCAGAGATATTACGGTTCCCACCATACAAAGGAGATA  95974

Query  3614   TCTGAGTCTCAGAGAGGTTGAGTGACTCGCCCGTGGCCACACAACC  3659
              |||||| ||||||||||||||| |||| |||| |||||||||| ||
Sbjct  95975  TCTGAGGCTCAGAGAGGTTGAGGGACTTGCCCATGGCCACACAGCC  96020
```

Range 5: 96063 to 96619

Score	Expect	Identities	Gaps	Strand	Frame
388 bits(429)	2e-107()	438/575(76%)	34/575(5%)	Plus/Plus	

```
Query  4277   AGAAAGTGATTGTTTCTAATTGGGGTATGGGGGAGAAGGGTGTAACTAGGAAGGCCTCCA  4336
              || ||||||||  |||| || ||||| ||||| |||  ||||||||||| |||| |||
Sbjct  96063  AGGAAGTGATTTATTCTGAT-GGGGTCTGGGG-AGAGAGGTGTAACTAGAAAGGTCTCTG  96120

Query  4337   GGAGGAGGTGGACTTCTGGCAGGGCCTCCAAGGGTGTCCAGGCCTCAATTAGGCCCACAG  4396
              | ||||||||  |||| |||||||||||||||||||  |  |||    || |||||||||
Sbjct  96121  GAAGGAGGTGACCTTCAGGCAGGGCCTCCAAGGGTGGTCCCGCCAGGGTTGGGCCCACAG  96180

Query  4397   ACAACCAGGTGCAGGTGCAGAGGAGAACCTCTGTTGACTGTGGCA--GTTCCATTTTTTG  4454
              | | | ||||||||||||||||     ||  | | || ||   ||  |||||  ||||||
Sbjct  96181  AGAGCTAGGTGCAGGTGCAGAG--CGTCCATTCTCGAGTGGCCCATGGTTCCTGTTTTTG  96238

Query  4455   CCTGACTGCCAAGTTTGAAAGTG-TGTATAAA-----TTAATACTAGTAGTTGGCCTCTG  4508
               ||||||||||| |||||||||| ||||||||     ||||| ||| |||||||||||||
Sbjct  96239  -CTGACTGCCAAATTTGAAAGTGTTGTATAAACTAAGTTAATGCTAATAGTTGGCCTCTG  96297

Query  4509   TGTGGTGTTAGGGGTCCTAATTTGGTAACTTCTGTTTATACCT-CTATACT-CGATGGAG  4566
              |||| | || ||||||||||||||||| ||||| || | |||| ||| |||  ||  | |
Sbjct  96298  TGTGTTTTTTGGGGTCCTAATTTGGTAGCTTCTCTTGAGACCTCCTAGACTGGGACTGTG  96357

Query  4567   TTTCTTTTGCTGTAATTCTAACTTGTAAACAGAGGTGGGCGAGGCACACATAACATTACT  4626
              ||||||||||||||| ||| | |||| |  | |||||||  ||| ||||||| ||||| |
Sbjct  96358  TTTCTTTTGCTGTAACTCTCATTTGTTAGTACAGGTGGGTAAGGGACACATAGCATTATT  96417

Query  4627   ATTCtttttttAACGTCATCATGTCA---CTCCTTGCTTGGGGCCAGGACGCCTGGAGGT  4683
              |||  |||||||| ||| || || ||   |||||||||||| ||||||  ||| || |||
Sbjct  96418  ATT-ATTTTTTAATGTCGTCGTGGCACTCCTCCTTGCTTGGAGCCAGGCTGCCCGGGGGT  96476

Query  4684   TGCCAGGCACCTACATGGTGGTGCTGA---AGGAGACCCACCGCTCGCAGACCGAGCACA  4740
              |||| | || || ||||||||||||||   |||||||||| ||||| ||||  |||  ||
Sbjct  96477  TGCCGGACATCTGCATGGTGGTGCTGAGGGAGGAGACCCA-CGCTCACAGATGGAGTGCA  96535

Query  4741   CTGCCCGCCGCCTGCAGGCCCGGGCTGCCCGCCGGGGCTACCTCACCAGGATCCTGCACG  4800
              ||||||||||||||  |||||| ||  ||  || |||           || |||||||||
Sbjct  96536  CTGCCCGCCGCCTGTGGGCCCGAGCCACCTCCCCGGG-----------GGTTCCTGCACG  96584

Query  4801   TCTTCCATGACCTCCTCCCTGGCTTCCTGGTGAAG  4835
               |||||| ||  || |||||||||| | |||| ||
Sbjct  96585  CCTTCCACGAGTTCTTCCCTGGCTTTCCGGTGCAG  96619
```

Range 6: 96662 to 97125

Score	Expect	Identities	Gaps	Strand	Frame
402 bits(445)	8e-112()	376/470(80%)	10/470(2%)	Plus/Plus	

```
Query  4957   AAACATCCATTAAGCACTTACTGAGAGCCCAGCACAGTGGCTCCTGGCCTTCAGTACAGA  5016
              ||||||| ||| ||||||| |||||  || ||||||||| ||||||||  |  ||||||
Sbjct  96662  AAACATCTATTGAGCACTTCCTGAGTACCAAGCACAGTGACTCCTGGCTCTAGGTACAGG  96721
```

```
Query  5017   ATGCCCTGTAAGCTTGGCCAGTCCTCAGCGGTACTTCCATCTTCACTTGGAAGATGAGGA  5076
              ||||||| | ||| ||||||  || ||  |||| ||||||| ||| ||| ||||||||||
Sbjct  96722  ATGCCCTTTCAGCCTGGCCACGCCCCAAAGGTATTTCCATCCTCATTTGAAAGATGAGGA  96781

Query  5077   GACCAAGGTTCAGAAGGGACCACCCAGACATCTAGGGGCAGAGCTGGCTTCAAACCCAGT  5136
              ||||||||||||||||||||| | ||||||   ||||||||||||||  ||||| |||||
Sbjct  96782  GACCAAGGTTCAGAAGGGACCGCACAGACAATAAGGGGCAGAGCTGGGATCAAAGCCAGT  96841

Query  5137   GGTGTGTCTGCTAGCTGTCTTCATGCTGATGAACTTGCTGCCTGTGGAAACCCTATAGGG  5196
              ||| || |||||||||||| |  |||||||||||||||||||||||| ||||  | ||||
Sbjct  96842  GGTCTGCCTGCTAGCTGTCCTTGTGCTGATGAACTTGCTGCCTGTGGGAACCTCACAGGG  96901

Query  5197   ACAAGGCCCCATGACATTAGTTGGGCCTGAGTCATTTTATAAAAGCCTGTCTCAAGGATC  5256
              || ||||||    |||| ||| |||| || ||||||||| || ||| || |||| || ||
Sbjct  96902  AC-AGGCCCTGCAACATCAGTGGGGCTTGTGTCATTTTAGAAGAGCATGACTCACGG-TC  96959

Query  5257   CAAAATTCCTTTGAAGCTGATGCTATTCAGAAGGTTTCTCCTGTAGGTCAAGGAGGCTCT  5316
              | ||||||||||||||| |||||||||||||||||| |||   |||| || | |||||||
Sbjct  96960  C-AAATTCCTTTGAAGCGGATGCTATTCAGAAGGTTCCTCTCATAGGACAGGTAGGCTCT  97018

Query  5317   -TCTCCCTCCAGCCTGGCCGTGATGTCACGTCTCTGGTGGAGGAGCCTTGAAA---GCAT  5372
                ||||| | ||| | ||| ||| |||||  || ||||| ||| ||   ||||    || |
Sbjct  97019  CACTCCC-CTAGCATAGCCTTGAGGTCACAGCTATGGTGCAGGCGC--AGAAAGAGGCCT  97075

Query  5373   GGGTAGTTGGGAACAGCTGGCCTCCCTTCTCCTCATCCTGGTCCTAGTGC  5422
              |||||| || | ||  ||||||||||||||||||| | ||| |||| |||
Sbjct  97076  GGGTAGCTGAGGACTTCTGGCCTCCCTTCTCCTCACCTTGGCCCTACTGC  97125
```

Range 7: 97192 to 97282

Score	Expect	Identities	Gaps	Strand	Frame
79.7 bits(87)	1e-14()	72/91(79%)	0/91(0%)	Plus/Plus	

```
Query  5609   CCCCTGTCCTCCTAACTTGATGAGGACACATGGTTCCCATTTTCACACTGATTTTCCATG  5668
              ||||||||||||||||||| |||| |||||  |||||  ||||||||||| || || |
Sbjct  97192  CCCCTGTCCTCCTAACTTGGTGAGAACACAAAGTTCCTGTTTTCACACTGCTTCTCTACT  97251

Query  5669   TGCCTAGGGTGTATCACAGCCTCCTTTAGAC  5699
              || ||  || |  ||||||||||||  ||||
Sbjct  97252  TGGCTGAGGAGCTTCACAGCCTCCTCAAGAC  97282
```

Range 8: 78509 to 78569

Score	Expect	Identities	Gaps	Strand	Frame
39.2 bits(42)	0.011()	45/61(74%)	0/61(0%)	Plus/Minus	

```
Query  5697   GACACTGAAACCCAGAGTGGGACAGGGTCTTGCCTGAGGTCACACAGCATAGAACTGGCA  5756
              ||||||||  | | ||| | | ||| | |||| |  | |||||||||||  |||||| ||
Sbjct  78569  GACACTGAGGCTCGGAGAGTGGCAGTGACTTGTCCAAAGTCACACAGCAAGGAACTGACA  78510

Query  5757   G  5757
              |
```

```
Sbjct  78509  G  78509
```

Range 9: 75346 to 75376

Score	Expect	Identities	Gaps	Strand	Frame
39.2 bits(42)	0.011()	27/31(87%)	0/31(0%)	Plus/Plus	

```
Query  5952   TTTAATCAGCACCTACTGTGTGCTGCAGACT  5982
              |||| | |||| ||||||||||||||| |||
Sbjct  75346  TTTACTGAGCATCTACTGTGTGCTGCACACT  75376
```

Range 10: 97369 to 97630

Score	Expect	Identities	Gaps	Strand	Frame
127 bits(140)	3e-29()	197/276(71%)	18/276(6%)	Plus/Plus	

```
Query  6204   AAGAGCTGGGCTTTGGCACCACGCCAGCCTGGCTTCACATCCCAGCTCAGCTTCTCACTA  6263
              |||| || |||||||||      |||| ||   ||||  ||||||||||||  || ||||
Sbjct  97369  AAGAACTAGGCTTTGGCCTTGTACCAGGCTCAGTTCAAGTCCCAGCTCAGCCCCTTACTA  97428

Query  6264   GTTTGTCTAACTGTAGGCAAATTCCTTCACCTCCC--AGT-TTC-TCCCCTATCTGTAAT  6319
              ||   |  | || | ||||||||| || ||||  |  ||| ||| | ||||||||| |
Sbjct  97429  GTCCATA-AGCT-TGGGCAAATTCTTTTACCTTGCTGAGTCTTCGTTCCCTATCTGGAGG  97486

Query  6320   TTGGGTCTAAAAATACAGACCCAAATGGAATGGTCATTTAAGGACTAAATGAGATCGTCA  6379
               ||||  |||| |||           |||||||||||| ||| |||||||||||| || |
Sbjct  97487  CTGGGCATAAACATA-----------GGAATGGTCATTGAAGAACTAAATGAGATGGTGA  97535

Query  6380   AGTATTTAAGCAGATGCTAAGCACAGAAACTCACAGAGGTGTGCACAGGTTACGGAAGCC  6439
               ||| ||| |||||   ||||  ||||||||||||||||  |   ||||||| || ||||
Sbjct  97536  GGTACTTAGGCAGACAGTAAGTGCAGAAACTCACAGAGGCTTTGGCAGGTTAAGG-AGCC  97594

Query  6440   CACGGGAATACTAAGGCACCCAGAGATGAGTTGCTG  6475
              ||| || |  ||  |||||||||||| |||| ||||
Sbjct  97595  CACAGGGAGCCTGGGGCACCCAGAGACGAGTGGCTG  97630
```

Range 11: 134151 to 134180

Score	Expect	Identities	Gaps	Strand	Frame
44.6 bits(48)	3e-04()	29/31(94%)	1/31(3%)	Plus/Plus	

```
Query  7453    tttaattttttatttttttAATTGAAGTATAGT  7483
               |||||||||| ||||||| ||||||||||||||
Sbjct  134151  TTTAATTTTAATTTTTT-AATTGAAGTATAGT  134180
```

Range 12: 95976 to 96019

Score	Expect	Identities	Gaps	Strand	Frame
44.6 bits(48)	3e-04()	36/44(82%)	0/44(0%)	Plus/Minus	

```
Query  8978   GCTGTGTGACCTTGGATAAGTCACTGACCGTCTCTGAGCCTCAG  9021
              |||||||| || |||  ||||| || | | ||||||||||||||
Sbjct  96019  GCTGTGTGGCCATGGGCAAGTCCCTCAACCTCTCTGAGCCTCAG  95976
```

Range 13: 99624 to 99671

Score	Expect	Identities	Gaps	Strand	Frame
41.9 bits(45)	0.003()	40/49(82%)	2/49(4%)	Plus/Minus	

```
Query  8978   GCTGTGTGACCTTGGATAAGTCACT-GACCGTCTCTGAGCCTCAGGTTC  9025
              ||||||||||| |||  ||| |||| |||| ||||||||| | || |||
Sbjct  99671  GCTGTGTGACCGTGGGAAAGCCACTCGACC-TCTCTGAGCTTTAGTTTC  99624
```

Range 14: 80099 to 80148

Score	Expect	Identities	Gaps	Strand	Frame
42.8 bits(46)	9e-04()	40/50(80%)	1/50(2%)	Plus/Plus	

```
Query  10454  TGTGACCTTGTGCA-GTTACTTACCCTTTCTGTGCCTCAGTTTCCTTGTC  10502
              |||||| |||  || |  |||||| |||||||  ||||||||||||| ||
Sbjct  80099  TGTGACTTTGGCCAAGAGACTTACGCTTTCTGGTCCTCAGTTTCCTTATC  80148
```

Range 15: 326945 to 326983

Score	Expect	Identities	Gaps	Strand	Frame
40.1 bits(43)	0.011()	32/39(82%)	0/39(0%)	Plus/Plus	

```
Query  10467   AGTTACTTACCCTTTCTGTGCCTCAGTTTCCTTGTCTGG  10505
               ||||||||   || |||||||||||||||| |  |||||
Sbjct  326945  AGTTACTTGAACTCTCTGTGCCTCAGTTTCTTCATCTGG  326983
```

Range 16: 82245 to 82272

Score	Expect	Identities	Gaps	Strand	Frame
38.3 bits(41)	0.038()	25/28(89%)	0/28(0%)	Plus/Plus	

```
Query  12832  TGGTGGTGGGGCACATGGCTTTGAGTGG  12859
              |||||| ||||||||||||| || ||||
Sbjct  82245  TGGTGGGGGGGCACATGGCTGTGCGTGG  82272
```

Range 17: 97754 to 98009

Score	Expect	Identities	Gaps	Strand	Frame
64.4 bits(70)	3e-10()	170/257(66%)	26/257(10%)	Plus/Plus	

```
Query  13777  CAGTGCTGGTGGCCGCAGCCGGCAAC-TTCCGGGACGACGCTTGCCTCTACTCCCCAGCC  13835
              ||||| || |||||   |||||   | ||| || | ||| | ||||||  ||| ||||||
Sbjct  97754  CAGTGTTGCTGGCCAGGGCCGGTGGCCTTCTGGAAGGACACCTGCCTCG-CTCTCCAGCC  97812

Query  13836  TCGGCTCCCGAGGTGGGTG--------------CTCCAGGA-GTACGGGAAGGT-GGCAG  13879
              |||||||||||||  ||||              | |||||| | | |||| | | |||||
Sbjct  97813  TCGGCTCCCGAGGCAGGTGTGGATGCCCCCAGCCCCCAGGATGGAGGGGAGGATGGGCAG  97872

Query  13880  G-TGGGCCCCTGTGGGCTTCATGGGGTGCACTC-------CTGAACTAGCCTGGCTTTGC  13931
              |   | ||||||||||||||||| |||   |||       |||| |  |||||||| ||
Sbjct  97873  GCCCGACCCCTGTGGGCTTCATGTGGTATGCTCCTGGAGGCTGACCCGGCCTGGCTGTGG  97932

Query  13932  AGGGAGGTGTCAGAGACTCCCAGGGCTGAGCCTGGACAGGGAAAGGGCTTGAACCTTCAG  13991
              |||| |  || ||| || || |     ||||| ||   ||| | || ||||||| | |
Sbjct  97933  AGGGTGTCGTTAGACACCCCAACACAGGAGCCCGGGGTGGGGAGGGTCTTGAACTTCCGC  97992

Query  13992  CATTCTCATCTATAAAC  14008
              ||| ||  ||||| |||
Sbjct  97993  CATCCTAGTCTATGAAC  98009
```

Range 18: 98040 to 98153

Score	Expect	Identities	Gaps	Strand	Frame
56.3 bits(61)	1e-07()	87/119(73%)	8/119(6%)	Plus/Plus	

```
Query  14086  CCTGAGTCCTCCGGCCCCTTCTCCC-CATGCCCATCACCTCCACCTGGCCCCTA--TCTA  14142
              |||||||||  ||| |||| ||||| | ||   ||| ||  | | ||| ||||   |||
Sbjct  98040  CCTGAGTCC--CGGTCCCT-CTCCCTCCTGG--ATCTCCATCCCTTGGGCCCTCCTTCTT  98094

Query  14143  CTCTCCCCTGGGTTGACAACACAGCTCCCTCAGCTTTCTCCTGGCCTCCCTCTGCTCCC  14201
              ||||||||||||  | || ||  || |||||||| |  |||  ||||||| ||||||||
Sbjct  98095  CTCTCCCCTGGGCCGGCACCATCGCACCCTCAGCCTCGTCCCTGCCTCCCCCTGCTCCC  98153
```

Range 19: 236745 to 236795

Score	Expect	Identities	Gaps	Strand	Frame
39.2 bits(42)	0.011()	41/52(79%)	3/52(5%)	Plus/Plus	

```
Query  14634   TCCAGCATGTCCTCCC--CACCTTGCTGGCTCTGTTTTTCTCCACAGCACTT  14683
               |||||||||  | |||  | |||  ||| ||||||||||||| | |||||||
Sbjct  236745  TCCAGCATGCTCGCCCTGCCCCTCCCTG-CTCTGTTTTTCTCTAGAGCACTT  236795
```

Range 20: 98222 to 98325

Score	Expect	Identities	Gaps	Strand	Frame
75.2 bits(82)	2e-13()	81/105(77%)	2/105(1%)	Plus/Plus	

```
Query  17388  TCCTTGTCTCCTGGTGCACCTGCTCCCACCTGACTGGTCCCATGCTGGGGCCCAACTGCC  17447
              ||| || |||||||  | ||||| |||||| | |||| || | ||||| |||  ||||||
Sbjct  98222  TCCCTGGCTCCTGGCACCCCTGCCCCCACCGGGCTGGCCCGACGCTGGAGCCTGACTGCC  98281

Query  17448  TGGTGCGAAGGCCTGTGCTACC-CTTCCATCCCTGTGACCCTGGG  17491
               || | ||||| | || || || ||||| |||| |||||||||||
Sbjct  98282  CGGGGTGAAGGTCAGTTCTCCCACTTCCGTCCC-GTGACCCTGGG  98325
```

Range 21: 99710 to 100080

Score	Expect	Identities	Gaps	Strand	Frame
176 bits(194)	6e-44()	297/428(69%)	63/428(14%)	Plus/Plus	

```
Query  17648   CTCAGGCCAGTGTCTCGTTCCTGCCCTGACTTATTTCTGGGTTTCCCAGCTCCAGCCCCA  17707
               ||||||| ||||||||  |||| || |||||| ||||||| |||||| ||||  ||| ||
Sbjct  99710   CTCAGGCTAGTGTCTC--TCCTACCTTGACTTGTTTCTGGATTTCCCGGCTCTGGCCTCA  99767

Query  17708   GACCCGAAAGAGATGGAGTCTGAATGGGGTGG---GGAGGACAGACAGATGGTCCCACAG  17764
               ||||| |||| ||||||||||||   ||||||   |||||| ||||||| |||||| |||
Sbjct  99768   GACCCTAAAGCGATGGAGTCTGA---GGGTGGCAAGGAGGAGAGACAGAGGGTCCCCCAG  99824

Query  17765   CATCCAGGTGTCTGAGCTGGCCCTCCTTTGCCCCAGGCTGCAGCTCCCACTGGGAAGTGG  17824
               | |||| | ||||| ||   | ||||| |||||||||||||||| |||| ||||| | ||
Sbjct  99825   CCTCCAAGGGTCTGGGC-CACTCTCCTCTGCCCCAGGCTGCAGCCCCCAATGGGAGGCGG  99883

Query  17825   AGGAATTTGGCACCCATGGGCCACCTGTGCTGAGGCCACGAGGTCAGGCTGATCAGTGTG  17884
               ||||  | ||| ||      |||       | |||| |            | |||||||
Sbjct  99884   AGGACCTCGGCCCC------CCA-------GGCGGCCCC------------ACCAGTGTG  99918

Query  17885   TGGGCCACGCGGAGGCCAGCGTCCATGCCTCCTGCTGCCACTCGCCAGGTCTGGAGTGCA  17944
               |||||| || ||||||||| |||||||| || ||| |||   ||||         | |||
Sbjct  99919   TGGGCCTCGAGGAGGCCAGAGTCCATGCTTCTTGCCGCCCTGCGCC---------GGGCA  99969

Query  17945   AATTCAGGGAGCACGGGATCCCGGGCCCTGCGGAGAAGGTGAGA-GGCGTGCTGGGCGGG  18003
                | |||        || ||||| |||||||| | || ||||| | ||| || |||| |
Sbjct  99970   CAGTCA--------GGAATCCCAGGCCCTGCAGCGACGGTGACACGGCCTG-TGGGGGCA  100020

Query  18004   GGACCGGGACGAGAGCCTGACACCCCAAGCGGTGGCCTGTGTCCCT--CCTGTGCCACTT  18061
               |||| || |      || |   ||||||| | |||  ||||| |||  || ||||  |||
Sbjct  100021  GGACAGGCA------CCCGGTGCCCCAAGGGCTGG--TGTGTGCCTGCCCCGTGCAGCTT  100072

Query  18062   TTCTGTGT  18069
               ||||||||
```

```
Sbjct  100073  TTCTGTGT  100080
```

Range 22: 100450 to 101948

Score	Expect	Identities	Gaps	Strand	Frame
557 bits(617)	2e-158()	1069/1549(69%)	164/1549(10%)	Plus/Plus	

```
Query  18483   TCTCCTTTTAAAAGCCACAGGGAACTTCTTCAAAGGAAGCCCTGCAGAGTTCACTTTTAA  18542
               || ||||| ||||||| |||||| |||||| |||||||||||||    ||   || ||||
Sbjct  100450  TCGCCTTTAAAAAGCCGCAGGGAGCTTCTTAAAAGGAAGCCCTGGGTGGTCTGCTCTTAA  100509

Query  18543   ATGAACTG-GAAGAGGTTTTTAAGAGTGTGAGTCTGTGCTGATTGTGTTCTGCATGCTGC  18601
               |||  ||  ||| ||||||||| |||   |||   ||| || ||||| |||| |||  ||
Sbjct  100510  ATGCGCTCCGAAAAGGTTTTTAGGAGCACGAGA--GTGGTGTTTGTGGTCTGGATGACGC  100567

Query  18602   ATTTCTGGAGGGCAAGGGC--TGTTCC-AGGTCCACTTGCTCAGCA---AATGT-TGAGG  18654
               ||||||  ||||||||| |  ||| || || |||||| ||||||||   | ||  ||| |
Sbjct  100568  ATTTCTTTAGGGCAAGGACCCTGTCCCCAGATCCACTGGCTCAGCACGGATTGCGTGATG  100627

Query  18655   CCTGTGGCATCCCAGGCAATGTTCCAGGCGGTGGGGATACAAACCCGACTAGCTTTCTCT  18714
                ||| ||  | || |||  ||| || |||  ||||| ||| |||||||||     ||| |
Sbjct  100628  GCTGGGGGGTTCC-GGCGTTGTCCCCGGCACTGGGG-TACGAACCCGACT-----TCTTT  100680

Query  18715   CCTGGCGCGTCCAGTCTAATGGGGGAGAAGGACAGCAAACAAATAAGTAACTATAGAGTA  18774
               ||||  | ||||  | |  |||||||||||||| | |||  || | |||| |||||||||
Sbjct  100681  CCTGAAGTGTCCGTTTTCGTGGGGGAGAAGGACCGAAAATGAA-ACGTAA-TATAGAGTA  100738

Query  18775   ATTAAAACATGCTATAGAGGAAAGTAAAGCAGGGAAGGGAATG---GGA--GGGTCCTTC  18829
                |||||| || |||| |||  || |||||||||||||||||||   ||   |||| || |
Sbjct  100739  CTTAAAAGATACTATGGAGACAAATAAAGCAGGGAAGGGAATGTTGGGTTGGGGTGCT-C  100797

Query  18830   AGGAGAGGCCTCCTTGAGAAGGTGGGGGACATCACAGGGAACAGTGTTCAAGGCAGAGGG  18889
               ||||| ||||||  |||||||||||  | |||| |||||||||||||   ||| || |||
Sbjct  100798  AGGAGGGGCCTCACTGAGAAGGTGGAAGGCATCGCAGGGAACAGTGTCGCAGG-AGTGGG  100856

Query  18890   GGTAGCCAGGGCAAAGGCCCTGA---GGTGGGAGTGGGCTTGGAGAGCAAAAGGAAGAGC  18946
                  ||||||||  |||  |||||   ||||||||   || ||||| |    ||||  ||
Sbjct  100857  AACAGCCAGGGTGAAGATCCTGACGTGGTGGGAGCATGCCTGGAGGGTGCCAGGAGCAGA  100916

Query  18947   CAGAGGGCTGGTGAGGTGGG-ACCCGAGTG--GGAGGGGGAACCAGAGACAGGGTTTAGG  19003
               |||  |||  ||| | |||| | | |||||  | ||||||| ||||||| |||   |||
Sbjct  100917  CAGGAGGCCTGTGGGATGGGGAACAGAGTGAAGCAGGGGGA-CCAGAGA--GGGGACAGG  100973

Query  19004   TGG--GGCCGGAGGGCCACAGGAAGGACTT-GGATTTTTACTGGAGTGAGCTGGGAGCCA  19060
                    | | || ||| | | |  |||||||  |||||||  ||| ||||| |||||||||
Sbjct  100974  ACCAAGTCAGGTGGGGC-CTG--AGGACTTTGGATTTTGCCTG-AGTGAACTGGGAGCCC  101029

Query  19061   CACAGGGTTCTGAG-CCTGGGTGTGGGGAGGGGGGTG-----------GGCTATCTGACC  19108
               |  ||||||||||| || |||| ||||| |||  |||           || | |||||||
Sbjct  101030  CGTAGGGTTCTGAGTCCAGGGT-TGGGGTGGGCTGTGCTCAGAGAATGGGGTGTCTGACC  101088

Query  19109   TGGGTGTGAGCAGGTTCATTCTGGTCGCTGTGTCGGGAAGACTGCAGGGGACAGGGCGGA  19168
               ||||||||  ||||||||||||||  ||     |||||||||| ||||||||| | | ||
Sbjct  101089  TGGGTGTGGACAGGTTCATTCTGGCTGC-----CGGGAAGACTCCAGGGGACAAGACAGA  101143

Query  19169   AGCAGGGAGGCCCGCTGTAGACGGGTGGACA--------GCCCGGGTGCTGGGG-GGTCC  19219
```

```
               ||||||||| || |||||||||| |||||||        | ||| | | ||||| | ||
Sbjct  101144  AGCAGGGAGACCAGCTGTAGACGAGTGGACATCCACTGTGACCGAGGGGTGGGGAGCTCT  101203

Query  19220   GTCAGGGCGGGAGTGTAGAGGATGCTGGAATCTGAAGGAGG-GGCTGCACATCTGATGGC  19278
               | |  || |||||||| |||  | |||||| | |||||||| | ||||| | |    ||
Sbjct  101204  GCCCAGGTGGGAGTGTGGAGCGTACTGGAACCAGAAGGAGGAGCCTGCAGACCCAGGGGG  101263

Query  19279   CTGGATATTGGGGGAGCAGTGGAGGGGGCGTCCAAGGGTTTTGCTTTGCTCTCGGACGAA  19338
               |||||| |  |||  | || |  ||| |  ||||||| || ||||||||||  || || |
Sbjct  101264  CTGGATGT--GGGTGGGAGGGCTGGGTGGTTCCAAGGTTTGTGCTTTGCTCCTGGGCGGA  101321

Query  19339   TGGCATCGCCCCTGACTGGGATGGGAAGGGCTGTGAGAGGTCAAGTGTCGG--------G  19390
               |||| | ||  |||||||| ||||| |||||||   ||||   || || ||        |
Sbjct  101322  TGGCGTAGCTGCTGACTGGCATGGGGAGGGCTGGAGGAGGCACAGGGTTGGGGTGAGGAG  101381

Query  19391   GAAGTTG----------------AGGCAT--------TTATGCGGGCCTGGCTCACAGCG  19426
               ||  |||                 |||||         |  ||||||||||||||   | |
Sbjct  101382  GAGCTTGGTGTTGGCCTGGCGATGGGCATCCCCTGTGTCCTGCGGGCCTGGCTC-TTGGG  101440

Query  19427   TG-CCGTGCCTTACATGTGCTTTCTTTTGTCCCCGGGCCCTGGCAGGTCACCGTGGCCTG  19485
               || || |||   |||  ||||| |||||||||| ||| || ||||||||||| ||||||
Sbjct  101441  TGCCCATGCAGGACACCTGCTTGCTTTTGTCCCTGGGTCCCGGCAGGTCACCATGGCCTT  101500

Query  19486   CAAGGAGGGCTGGACGCTGACCGGCTGCGGGGCCC--ACCCCGGGGCCTCCCACACCCTG  19543
               | ||||||||||||||||||| |||||||    ||   |||||| ||||||||||| |||
Sbjct  101501  CGAGGAGGGCTGGACGCTGACTGGCTGCGCCCTCCTGCCCCCGGAGCCTCCCACACACTG  101560

Query  19544   GGGGCCTATGCAGTGGACAACACGTGTGTGGTGAGGGGCCGGGACGTGGGTGTGCGAGGC  19603
               |||  |    |  |||||| |||     || |                ||||   |||||
Sbjct  101561  GGGATCCCCACGATGGACAGCAC-----TGCT----------------GGTGCAGGAGGC  101599

Query  19604   AGGACGGGTGAGGAGGCCGCCGTG-GCCATTGCCATCTGCTGCAGGAGCCGG---TCAGG  19659
               ||| |  | |||||||| |  | | ||  |  ||||||||||   ||||| |   || ||
Sbjct  101600  AGGGCCAGCGAGGAGGCTGTGGGGAGCTGTCACCATCTGCTGTCAGAGCCAGCCCTCGGG  101659

Query  19660   GGAGCAGGCCTCCCCGGGGACCCAGTGACAGCCCCGCCCAGGA---TATCTG--------  19708
               |||||||||  ||| |||| ||||||||| |||| ||||||||   ||  ||
Sbjct  101660  GGAGCAGGCACCCCAGGGGTCCCAGTGACTGCCCAGCCCAGGACTGTACGTGGGCAAAAA  101719

Query  19709   ---CGTG-----------GCTGGGGTCCCAGGCCTTGGCTGAGCTTTGAAGTGCTTCCTT  19754
                  | ||           |||| |||||||||||||||  ||||||||    | || ||
Sbjct  101720  TCACATGTCAGGGCCTGAGCTGTGGTCCCAGGCCTTGGGGGAGCTTTGTGAGGGTTTCTC  101779

Query  19755   TTTCCTCCTTCCTCAGCCCTCCTCAGCCTGGGCCCCGGGGGACAGAAGGCACCTCTTTC-  19813
                ||||  ||||||| ||||||||||||||||||||| |||||| |  | |||||||| |
Sbjct  101780  CTTCCCTCTTCCTCTGCCCTCCTCAGCCTGGGCCCCAGGGGACGGGGGACACCTCTTACC  101839

Query  19814   -TCCTGGAGCTCTGGTGCTGGCACTTGGGGTACACT---GGCTCCCTGCCTGGGAGAACC  19869
                ||| |||||| |||  ||||||    |||  |  |   |||||| |||| ||||||||
Sbjct  101840  TTCCCGGAGCTATGGCACTGGCATGGTGGGGGCGGTGTGGGCTCCGTGCCCGGGAGAACG  101899

Query  19870   CCATCTCTTGGCCCGAGTCACCC-CTCCCCAGACCCGAGCTGAGTGGGA  19917
                 | | ||  ||| |  || |||  |||||||||| | ||||| |||||
Sbjct  101900  TGACCGCTCAGCCTGGATCGCCCTGTCCCCAGACCTGTGCTGAATGGGA  101948
```

Range 23: 101975 to 102000

Score	Expect	Identities	Gaps	Strand	Frame
39.2 bits(42)	0.011()	24/26(92%)	0/26(0%)	Plus/Plus	

```
Query  19989   ACAAGGGTCTGGCTGTGCTCAGCTCC  20014
               ||| ||||| ||||||||||||||||
Sbjct  101975  ACACGGGTCAGGCTGTGCTCAGCTCC  102000
```

Range 24: 102085 to 102181

Score	Expect	Identities	Gaps	Strand	Frame
81.5 bits(89)	4e-15()	79/98(81%)	3/98(3%)	Plus/Plus	

```
Query  20190   TGTGAAAGGTGGAAATGGCCCCTCACCTCCTGCCCACTCTGGGGAGGCCCGGTTGGGCTC  20249
               |||||||   ||||||||| ||| ||||||||||| || ||| || ||||  ||||||||
Sbjct  102085  TGTGAAACAGGGAAATGGCTCCTTACCTCCTGCCCTCTTTGGAGAAGCCCCTTTGGGCTC  102144

Query  20250   CCTGAT-TATGGA-GATGAGTTTTCCATGCCTCTGGGG  20285
               ||||||  | ||| ||| ||||||||||  || |||||
Sbjct  102145  CCTGATCAACGGAGGATCAGTTTTCCAT-ACTGTGGGG  102181
```

Taxonomy

Reports

- **Lineage**
- **Organism**
- **Taxonomy**

Dot Plot

Plot of lcl|Query_29865 vs lcl|Query_29867

Top
