# Supplementary material for: Molecular evolution of PCSK family: Analysis of natural selection rate and gene loss
Source: PLoS One. 2021 Oct 28;16(10):e0259085. doi: 10.1371/journal.pone.0259085 (PMC8553125; doi:10.1371/journal.pone.0259085)
Supplement: S15 File — Exons are indicated in red. Regions with homology to the intergenic sequence of BSND and USP24 in Felis catus are underlined. (PDF) [file pone.0259085.s021.pdf]

CAAGACAGAGCCCAGGAACTTTGCGGATGTGTCTGTCATCGCACGCAGGGCTCAGGGTGA
GGGGCGGAGAGAAGGCATCTACAGGGCACGCCGGGACAGCTTTCCAGCCCAGTTAGCGTT
TGGGATTTTTTTCCTCCCTCTGAGGGTAATCTGACGTGGTTTGGGAAGGGCGAGGCTGAA
ACTCGATCCATCAATTCTGGGGGGTGGGGGGAGCCAGTTAATGTTTAATCAGGTAGGATC
ATCCGATGGGGCTCGAGTGGCGTGATCTCCCGGGCCCCGGGCGTCGCGCACCCACACCCC
AGCAGGTTTCAGCCTCGGCGTTGAGGCGCTCTCGGCTGCAGGCGGACTCAGGCTTAGCTC
GGGTCCGAGCCCGGGGGAGGCGAGCCAGACAGTGAGAACTCTCGGGTCCCGTAAGCGTGG
CCACGGCGCGGAGCCCCGAACCCAGAGCCCCAAGGACGGGCCGCGCGGGTGTCCCTGTTG
GGACCCCAGGTCCCGGCGCGCGCCTAGAGCTCCCCACAGCGAGGCACAGTGGCGGCCGGC
CTTGGCCAGCGCGCTGCCCCCGGGTCTCCCCGCCGAGCGCAAACCTTTCCTCTCCCCGCG
ATGGGCGCGGACAGCTCCTGGCGGCCATGGTGGCCCCCGCTGCTGCTGCTGCTGCTACTG
CTCTTGGGCCCTGGAGGCTCGGGCGTACAGGAGGACGAGGACGGCGACTACGAGGAAATG
GTGCTCGCCTTCAGGTCGGAGGAGGACGGCCTGACTGACACGACCCAGCACGTGGCCACC
GCCAGTTTCCATCGCTGCGCCAAGGTGCGGGCGCCAGGGGCGAACCCGCGTGGGGGCCCC
AGCGGTGGCTGATTCCTCTCCGGCCTCAGTTCTCCCCAGTAAGGGAGAGTCTAGAGAGAA
GGTTTCCAGTGCCTTCTGCTCATCCCAGGACGGGCTTGGCGCAGATCTTGAGGACGGCAG
GCACTGCGGCAGGGGACCGAGTACAGTAGTTCTTTGGGGTGCGCTGTGCTGGGGAAGGCG
CACAGGGGTGGGAGACTGGAAGACGTCAGGTAGGGCGAGCAGAGCACCTCCAGGACAGCC
TGCGCATATCCCAGACATGCCGCACCACCGAGGCTCTGGTGGGGAAAGGTGCTAAAGCCT
GGACCCCGCTTAGAACGCCCCCCCCCAACCCCTGCACAGAGGAAACAGACTTGCTATTAT
TATGCATCCTGAAGTGGATGGGGGAAATCTGGGCAGTGTAGTTGTATTGTGGGGAGTGTG
CGGGGTGGGGAGTGGGAGTGGGGATGGTCATGGGGATCTTGGGGAAGGACAGCACTGCCG
TGGCAGGGGTGGAGTGGGAGGGAAGGCGAATAATGGGACTGGAGGCAATTTCTACAGGCC
ACAAAACTAGTATTGCATCCTTTTCAGCTGAAGAAAAGAACAGAACTAAAGGCAAAGGGG
CGGAGTTATTCTCAAGGCCCTTTATGGTCCTTGGGGTCCTCAGGCAAGGAAGGGCTTTGT
GGATGCTCATGAGCAGGAGGTGGGCGCACCTGGTAGCTGGGACAAGGAGGCTGAGCCCTT
CAGCCCATGCGCAGGTCCTGCCGGCATAGGCGGGGGTGGGCAGGGCGAGTTTCCTGAAGA
TTGATGCCAGCACCTGGCTCTAGGGTTATGGGAGCTTCTGCCCAGGGGGACCGCTGGTCC
CTCCAATTATAACCTTCCCAGGACTCGACTGAGGTCCCAATACAGGACTTGGAGTCAGCC
CTGGGGTTGAATCCTGGCTCCATCACCCACTAGCTCTGTGATGCTTGGCTCGTCACTTAA
CCTCTGAGCCTCCATTTCCTTATCTTCAAAAGGGAGGTGACAGTTCTTCCCTAGGGTCTG
TTGTGACATTTCAGTGCTGGGCAGATGGAGGAATGAAGGGGAAAGGGCTCTATTGCTCAC
ATGCATGACCTCACCGGGATGTGAGCCAGTGCAGAGAACACTGTAGTTATTTCCCTGGCT
GCTGTGTGACCTCCCGGTGACATCCTCTTTACTCCAAACTGCAGCTCCTGGAGCAGAGGG
AAAGTTCTAGGCTAATAGACACCAGGCCTGCACTTCTGCCCCAGCCCCTCTGCCTAAGTG
TGCTAGGGTGGGGAGGGATGTCAGGCCCTTAGTGTTACCTGTGCCTGGTGTCAGTGGTAG
TGGGGAGAGACCTCTCTTCTTCGGTCTGGGTTTCACAAAAGAGTGACATTTACTTAGCTC
AAATCACCCTCTTTCTTGTTCCCTGAGCCTTTCACCTTCTAGAAGGATGTTGCTGGGTTG
TGGCAAGGATGAGAAAGGGTGTTTCAAGTCACCACTGTCCCCAAGTAACTATTCTAGGAG
TAGTGAGTACTCCATCTTGATAGGTAAGCAGTGACTGGACAACCACCTGAACCAAATGCT
TGAGAGGGGAGAAGGGTGGCTCAGTGGTAGAGCACATGCTTAGCATACATGAGGTCTTGG
GTTCAATGCCCCATACCTCCATCAAAATTAGTAAACACATAAATAAACCTAATTACCTCC
CCAAAATAAATAAATTAATTAAAAAAGACACTGAGGGTATTTCTTCCCTGGTGGAAGTTT
GAAACAGACCCTCCAGAAGTTCATTGATTCAATGGATATTTTGTGGGGATTGAATTTAGA
ATGAACATTTTTTTGGCAGGCAGATAAAGATTTAGACCAGTCCTTTTATTTTATTCATGA
GAAGCCCAGAGAGGGGGGGTCCACCCTCCTGATGCATTAGAACTAGTCTTCCAGGAAAAG
TCTCCTTCCACTGCACAGAGTGCTCTCCCAATTCATTAGAGTTTCATTTAGTGGAGGGCA
TTTTAGATGGGCCTTTGAAACATAAATAGGAGTCTAACAAATGAAGGGAACAGGGGAATT
TTATTCTAGGGGGAGGGGGTAGCATGAACAAAAGCGCAGACCTGGGAAAGCCAGAGATGG
AGAATGGGAAGCACATGTCCACAGTCCCTTATCCACCTTCTGAAATGTAAAACTGCTCCC
CAAACCAAAAGGCTTTTTGTAATTTATTTTGTGGTAACCTGACCTGAACTGACATGAGGT
TGTTTATAATTTATCCCACTGATATATTCACATTCATATTTATATTAACAGATTTTTTGC
TGCATAGATTATAATATGCTGGTCCAGATCCCTCTGAGCGCCCTGACTGCCTATTACTAC
CTTTCTAAAATCCAAATAAGTTACAAATATTGAAACCCATTTGGCCCTAAGACTTTGGAT
AAAGGATTGCAGACTCTGTGCTCCTCTCTCTGGTGCGCATACAGAGATGTAGGAGATTAG
GCTACAGAGGTAGGTTAGAGAGGGGACCAAGGAGAAGCATGGAGTTTGGACTTTGTCAGG
TTATGGGGAGCCACTGAAGGTTCTTGAGCTCAGGTGTATCTGTTTGAGAGCAGCAGACAC
AGATAAAAGCTAACTAAGAGCAAAAAATCTGCTCTGGCAGACCACGACTTGAGTCTTTTC
TCCCACTTGAAAAGTGTTGCCTTTGCTCACTCAATCATCCCTTCTGTTTGCTAGATGCTT
TACGCAACCACCTTTCCTAGCCTTCCCAGCAGGCCTGTGCCATAGGTATTACCCCGACAA
CATAGAGTTGATGTCTGAGTCTCAGAGAGGTTGAGTGACTCGCCCGTGGCCACACAACCA
GGAAATATTGAGGCTGGGATTCAGTCCACATTTTTGGTCTGCCTCCAGAGGGGGCCATGG
AGGTACTAGAACGGGGAGAAAGTGAGGGTTCTTTGCTTTCTGTTTCTTTCCTGGTCTGGC

GGGTGAGGGAGGGGAGGGGGGAAAAGCACGGGTACGGGCCGGGCAGGGAAGGCGAAGGGA
TAGGGAAGGGACGGGAGGGCGGGAGGGAGGGGAGGGGAGGGGGAGGGGGCGGGATGCGGA
GGGCGAGGGAGGGAGGAAGGGAGGGAGAGAGGGAGGGCGGGGGGAGGTGAGGGAGGGATG
GAGGGAGGGTAGGGAGGGAGGGAGGGAGGGAGGGGGGGGAGGGAGGAGGGAGGGAGGGAG
GGAGGCTGGAGGGAGGGAGGGATCCCGCCTCCTGCGGTTCGACCTACACGCACGTATTTT
CGTCCCGTAAGGTCTGTATGTCTTTCTCCGGCCCGACAATGTGTCTGCTTTCTTTCTTTC
TTTCGTTATTTTTTCTGTATTTCTTTCTTTCTGTCTTTAGTTCTTCCTTCCTATGTTCCT
TCCTCTTTATTTATTTCTTTCTTTCTTTCTTTCTTTCTTTCTTTCTTTCTTTTTCTTTTC
TTTCTTTCTTTTTTAAAGAAAGTGATTGTTTCTAATTGGGGTATGGGGGAGAAGGGTGTA
ACTAGGAAGGCCTCCAGGAGGAGGTGGACTTCTGGCAGGGCCTCCAAGGGTGTCCAGGCC
TCAATTAGGCCCACAGACAACCAGGTGCAGGTGCAGAGGAGAACCTCTGTTGACTGTGGC
AGTTCCATTTTTTGCCTGACTGCCAAGTTTGAAAGTGTGTATAAATTAATACTAGTAGTT
GGCCTCTGTGTGGTGTTAGGGGTCCTAATTTGGTAACTTCTGTTTATACCTCTATACTCG
ATGGAGTTTCTTTTGCTGTAATTCTAACTTGTAAACAGAGGTGGGCGAGGCACACATAAC
ATTACTATTCTTTTTTTAACGTCATCATGTCACTCCTTGCTTGGGGCCAG**GACGCCTGGA**
GGTTGCCAGGCACCTACATGGTGGTGCTGAAGGAGACCCACCGCTCGCAGACCGAGCACA
CTGCCCGCCGCCTGCAGGCCCGGGCTGCCCGCCGGGGCTACCTCACCAGGATCCTGCACG
TCTTCCATGACCTCCTCCCTGGCTTCCTGGTGAAGATGAGTGGCGACCTGCTGGAGCTGG
TGAGCTCCCTCTCTGGTCAGGGTACTTCCTGCCAGGGCTGGGCCACCACATACGTATGGG
GGACAGTCCTTGGTGTGCTGACAATCAGGAGGCAGCAAACATCCATTAAGCACTTACTGA
GAGCCCAGCACAGTGGCTCCTGGCCTTCAGTACAGAATGCCCTGTAAGCTTGGCCAGTCC
TCAGCGGTACTTCCATCTTCACTTGGAAGATGAGGAGACCAAGGTTCAGAAGGGACCACC
CAGACATCTAGGGGCAGAGCTGGCTTCAAACCCAGTGGTGTGTCTGCTAGCTGTCTTCAT
GCTGATGAACTTGCTGCCTGTGGAAACCCTATAGGGACAAGGCCCCATGACATTAGTTGG
GCCTGAGTCATTTTATAAAAGCCTGTCTCAAGGATCCAAAATTCCTTTGAAGCTGATGCT
ATTCAGAAGGTTTCTCCTGTAGGTCAAGGAGGCTCTTCTCCCTCCAGCCTGGCCGTGATG
TCACGTCTCTGGTGGAGGAGCCTTGAAAGCATGGGTAGTTGGGAACAGCTGGCCTCCCTT
CTCCTCATCCTGGTCCTAGTGCTTTAAATGAAAATCCTTTCTTGGCAAGTCTCCCTGCTG
AAGAGAAGGGGGCTCCACTTGAAGCGAGTGATGGATGTAAGATTTGTGGCCTTAATTTAA
AGGCAGAGGAGAGTCCTGAAAATGCATCTTTAAAAAAAAAGTCTTGCTTGTTTTAGCCTC
TGTCCCCTTCTCTCCAACCCACCCCTCTCCCCTGTCCTCCTAACTTGATGAGGACACATG
GTTCCCATTTTCACACTGATTTTCCATGTGCCTAGGGTGTATCACAGCCTCCTTTAGACA
CTGAAACCCAGAGTGGGACAGGGTCTTGCCTGAGGTCACACAGCATAGAACTGGCAGGGC
CAGAATTGGGCCCAGGGCTTCTTGCTCCACTGCACAACCACTGCATCGTTTAATTCAGCT
CAGCACACAGTGGCTGAACAACTGGGTGTTAAGTCCTGTGGGGACAATGACATGGATTGG
ACAGTGTCCAATCCCTTCATCTAATAGGGGAAACCTCAAGTTAATGCTTCCATCAGTCTG
CTCACCACACATTTAATCAGCACCTACTGTGTGCTGCAGACTCAAGGATGAACCAGACCC
AGCCCTTTCCCTTGAGCTCACAGTTCAGCAGGGGACACTGAGGAGTGATGGGCAGTGCAG
TTAACTGGGGAATGGCATCCCCAGTGCAGTGGTGGGGAAGGAATCAGGAACCCACAGAGC
CAGAGGGCAGGTGTGAGCCCCAAGGCTGGGCAGCTTCTCAGAGAAGAGATGCTGCTGACA
GCAGGTCACAGACATTTGCCTTCAAGAGCTGGGCTTTGGCACCACGCCAGCCTGGCTTCA
CATCCCAGCTCAGCTTCTCACTAGTTTGTCTAACTGTAGGCAAATTCCTTCACCTCCCAG
TTTCTCCCCTATCTGTAATTTGGGTCTAAAAATACAGACCCAAATGGAATGGTCATTTAA
GGACTAAATGAGATCGTCAAGTATTTAAGCAGATGCTAAGCACAGAAACTCACAGAGGTG
TGCACAGGTTACGGAAGCCCACGGGAATACTAAGGCACCCAGAGATGAGTTGCTGTGACG
AGTTGATGTGAGAGGGAAAAGTGTCACCTCTGCCAGGTGGGAGCTGGTGCCGTGGCGGGA
TGTGGTAGAGAAGGGGCTGCCCCAAGGAGGCCGTGGTCACCAAAGCTTGTGGCCATTGCA
GGAACCTTATGCCAAAACAGGCTGGGAGTGGAGAAGGCACCCTCATCCCCGAGACTCCTA
CTGGAACTCCCTCTGGCTGAGCCCAGCTGGAAGTCGCTGCAAGGAGGCCTGGGTGCCACA
GTCTGCAGGGTCAGCCTCCACTGCGCAGGACGGAGAAGGGCAGGAATGGATCTGGGGAAA
CAGAATGGCCAGTGCCGGCATCATGATTTGGGCATGGAGTCCAGGTCCAGCCTGCCCGGA
GCCTGGGCACTGCCTGGCTCACCAGATGGCCTATCAAGGCATTCTCTGTGCCAGTTGGTA
TTGGGCTCCCCAGCCTGAGTGAGGAGTGAGGAAACCCAGTGCCAGGATGGGGGCAGGGAG
GGTGCCTGTGTGTGACTCGGGACAGGCTTGATCATGTTGGGTAAGGGCTTAGCTGTGTTT
GTTGTTCACCAAATGGCTTCTGAAGCAGAGCCCCCATCCTCTCCGGCTTCTGCAG**GCCCT**
GAGGTTGCCCCACGTCCAGTACATTGAGGAGGACTCCTTCGTCTTTGCCCAGAGCATCCC
GTGGAACCTGGAGCGAATTCTCCCTGTGCGGCCCCAGGTGGATGAACACCACGCCCCCAG
TAAGCCCCCTGCATCCTGCTCCTCTCCATCCCAACTGAGTCCACATACAGCTCTCTCTTC
CACAGGGATGGTCCATGCCGCTCAGGGGCTTTAGAGCTCAGCACACTCCAATGACCCCAC
CTTTTCTGTCTCATTCCCTCCCCCACTCCAGCTCCCACCTCTGCCTTCCTACTACCTGTA
CAATGCAGGAGTCTTTTTTTTCCCCCCTCCCTCCTTTCCATCATCAAGCAATGCTCTTTT
CTTTTTTTCTTTTTTAATTTTTATTTTTTAATTGAAGTATAGTCAGTTCACAGTGTTGTG
TAAATTTCTGGTGCAAAGCATAATGTTTCGGTCATACACATACATACATATATTCCTTTT

CATATTCTTTTTCACTATAGGTTATTACAAGCTATTGAATATAGTTCCCCGTGCTACACA
GTAGGACCTTGCTGTTAATCTATTTTATATATAGCAGTTTGTATCTGCAAATGCCGATCT
CCCAATTTATCCCTCCATCCTCCTTCCAGCCCCGGGAACCACAAGTTTGTTTTCTATGTC
TGTGAGTCTGTTTCTGTTTTTTTAAATAAGTTCATTTGTGTCTTTTTTTTTTTAGATTCCA
CATATAAGTGATAGCATGGATTTTTCTTTCTCTTTCTGGCTTACTTCACTTGGTATGATG
ATCAGGAGTCTTTTCTTAAATGAGCTCTTCTCCCACTTTCTTGAAGTTCTTGTTTGCCTC
TTCTCTCCTTTGGAATGGCCCAGCAGGCCGCACTTCCATGGCGACAGGGTAAATCTGACC
TTGACACTCCCTAAGGCCCACAGGTCTTGGTGACTCCCAGAGCCCTGAGGACAGGATGGG
ACCCCTTAAGAGAACAAACAAGCCCTGTCCGCTCTGCCCGATCTGGTCTCTGGTCTCCTG
CCTTACCCTGCTCAGCCTTCCTCCAGCATTGCTGGGCTTTCTGGGGCTCTGTGTCGGGGC
CATGCTGTGTGTCCCCTCCAGGCCTCTCCTCTCACTCTTCCGTGTGCCTGAGGCAGCCTG
GCTAGGGCAAGGAGGAGGGGGAGGAGAGACCAAGGATAGTGGCCTGAGTTCCGGCAGGGC
CTTGTAGGTGGGTGGAGGTGGGTTTATTGAGCTGGGGAAGACAGGAAGGGCACCTGGTTT
GGGGAGAGAAGATCAGGGTGCTAGTTGGACCCTGCTGAGTCTGAGGAGCCCATGGGATGA
GGTTTGGAGCGGAAAGATGATGCAATGATATGCCAGGACTCAGCCAAGCCTGGGGACCAG
TTCAGCCTCCATCCCTTACTGGTTCACGTGGAGTCTTGGGAAGCTACTTCCTTCTCTGAG
CCTCCCCTTCTCATATGCAAAATGGGCACAGAGAACCCTGTCTGGTCCTCCTCATAGGGT
GTGTTGAGGCCCCAGTGAGGTGAGGGATGGGCAAATGCTTTGGGAACTGAAAGGCTGGGT
GCTTCCCCAGGCCAGAAGCAGATATGGGACCATTTTCTCCGGCATTGGGATGCCCAGGGA
TTGCCTTACTCCTCTCTTGTTCCCAGTGGTGCTGGGAGGTGGCGGGATGGAAGGCAGGAG
TGTGGAGTCCATCTGGGATCACAGCAGGCTGGATGAGATCCCTGGGAGCTATTGGGTTGG
GGTAGGGCAGAGTGGGCACCATGCAGACAAGTGGAGAGTCAGCTCGCCAAGCCTGGAGCA
GACCCTTCCTTCACAGAGAGGCCACCTGGCACAGGGGTGACAAGCCCTGGCTCAGGAGCC
GACTCCTGCCCTCAAACCCGGACTTCAGCAATCTCAAGCTGTGTGACCTTGGATAAGTCA
CTGACCGTCTCTGAGCCTCAGGTTCCTCTGCAAAAGGGAGGTAATGATAGTTTCTACCTC
AGGGGCCGTGCTGAGGGATAAATGCCCTTCTTGCTGCGGCACGCATCCATCCGTGGCTGG
TATAGAGTGAGGGTGTGTCATTCTCCCCTTCTCCCATCTCTTCTTCAGTCCCACAATAAA
TTCTCAAGCAGCCAGCATGCTCCAGACACTATGCCAAGTGCTGGGGACACAAAGACGAAC
AAGATGGACTTGGTCTCTGCCCCCACAGAGCTTCTGGTGCACAAAGAAGGTTCATCCATT
GCTTAAACAGCTGCATGAGACCAGTTAGTCTCAATGGGGTAGGAGCTCCAAAGCAGTTTG
GACCCGGCTGATGGCTGGGGGGTCAGGAAAGGCTTCCTAGGGGAAGTGACATTCAAGCCA
AGACCTGCAGTGAGGACCATTAGCCATGCCAAGGGGAGGGTGTCCAAGCAAAGGCCCTGA
GGCAGGAAGGAGTTTGGCCTGTGAGGAGGGGCCAAGAAGGTCATGGGCAGGGGCCTCCTG
GGCAGAGATGGAGGGAGAACTTGGCTACCGTCCGAGCTTCTTGGGTGCGGCAGGGGCTGC
CTCATGGGAAGGAGAGAGCTCCCCGCTCCAGAGAGATGCACTGGGCGCCCACCTGCCAGA
GGTCACAGGGCTTTCTGTCCCAGACCAGAGGCTGGATGAGGCCACTCCCAGGTCCCTTTG
CCTCTGAGTGATAACTGCTCTTGAGGTCCCTTTCCCCTCTGCGACATGGGATGACAGTAG
ACCCACCTTGCACAGGGGCTGTGAGGTTGGATCTCTGAAGATTCTGAGAGCAGTGCTGCG
GTCTGGGGCTCGGCCTACCTGACCCTCTTCCTGCCTCTCTGACCACAGGAGTCGCCCCTG
CAGGCTCTCCCTGCTTCATCTTGCCCCCTCCACCTCTGTCCTGGGTAGGCGTGCCACCGA
GAAGTCCCCTGCTGGTTTCATCCCATGTTGGTGCTTCCTTACTGGAGAATCTGAACTGAC
CCAATTAGAAATGATGAAGTGTAGATGGCAGGCGCTTGGTGAATTTCCAACACTGCTGTT
TTCTCTGGGTTGTAACACGTGTCAGTTGGAACCCGTCACTATGAGCCATCCTGGCACCTT
GCGGAGTGGAGAAAGCCTGGGCGTGGAGCCCAGAGGCCAGATCCATGCATCCTCCCCGAG
CCTCAGTCTCCTCTCTGTGAAATGAGCTGGACACTCAGATGGCCAGATGGCCCCGTTAGT
CTCCTTTTATCCTCCAAGCCCTGTTCTGTCTCCTCCTCGGGCTTGGGGAGCTGTGAAAAG
TGTAAAGAGGGGCTTGGCTTATTTTTTCCCATTATATTTATTAGCTTTGAATGTTCGTAT
TGTTATTTCACATTCATATTATGCAGCCAGATTAATATTATGGTTCTCCTGCTGGTTTCA
CCATCACCAGCTGTGTGACCTTGTGCAGTTACTTACCCTTTCTGTGCCTCAGTTTCCTTG
TCTGGGCAATAAAAATATAATAGTATGTACCTCGAGAGGATTTTTTTGACTTAATGTATG
TAAGGTGCTGGGAGCAGGGCCTGGGATGTGGTAAATAGTTTATATGTGTTAATGGTTATA
TTAACCTTAAGGTTATTCTTTCCACTTGAACAAATCTCCCCTTGGAAAG**ATGGAGGCGGC**
CTGGTGGAGGTGTATCTCTTAGACACCAGCATCCAAAGTGGCCACCGGGAACTTGAGGGC
AGGGTCACAGTCACTGACTTCGAGAACGTGCCCGAGGAGGACGGGACACGCTTCCACAGA
CAGGTGAGCCCTTTCCTCAAGCGGGAGGGCGGCCCCGACCCTCTCGCCCCACCTAGAGTG
ACCCCACCCCCGGAGTGTCACAGCTGCGCTCCTGCTGCCCTCCACCCTGCGGCTGCTGCC
CCGATCTTGCCATCAGGTGTGGGTGGGGGCATCTGTCCCGCCACTCGCTGATGTATTTTG
GGGTGGGTGGGCTTTCTCACTTGGGCCTTGTGTTTGTTGAGCAG**GCAAACAAGTGTGACA**
GCCATGGCACCCACCTGGCGGGGGTGGTCAGTGGCCGGGATGCGGGTGTGGCCAAGGGCG
CCAGCCTGCGCAGCTTACGTGTACTCAACTGCCAAGGGAAGGGCACAGTGAGCAGCACCC
TCACAGGTGAGCCATGACTTCGGATGCCTCAGTCTCTGCATCCAGACCTGGCATGGGATG
GAGCTTCAGCCCAGAGAGAACTGACTCCTGACCGACAGGGTCAAGGCAGCCTCTGCCCCA
GAGGCAGAGTCCCAGCGTGTCAGAGAGGGCGGGGTCCCCGGGGGCACAAGTGTAGATGGA

GAAACGGAGGCCCAGAGAGGGGCAGGGCTCAGCCCGGCTTTGACCCCTGGTCTTTCTACA
GTTTCACACTGCTCCCCTTTTCAAAGCCTTTAAATTTGTTGTCTTTTGTGATGTTATTTT
AGATTTGCTTGGGCCCTTGAGGTGATCTAAGCAAACTTTCTCCATCTTCTGTTTGCTTAT
CTCTAACACTAGGGGACTCACTACCTTGCATGACTGATTGGGCCCTGCAGGTCACCCTGT
TCGGGTGGACTTGGTGGGGGAACTGGCAGAGGACTTTTTCCAGGCTCTTGCAGGTTTCTC
TATCTGGTTGCCTCTGGTGAGGTCCAGCTGAGAGCTAGGACCCTGGAGGGGGTCTATGGA
CAGAGAAGAGGGTGAAAGATCTCACTTACTGAGTCCTTCCTGTGGCCAGACCTTGAGCAA
AGGACTTTGTACTCCATACCCTGAGGCTGGTATTGTGATCTTGTTAAACAGTTGATAAAA
CCAGCCCAGAGAGGGGCGGTGACTTGCCTAGGGTTACACAGCTAGAGCCAGTGACCCCAT
TGGGGAAGGTACCAGCTCTGAGTTTGACCTCCACAGCAAGCCCGCAGACCCCCACGTCAG
ACACTGGCTCCTGAGCTGGCAGAGGCAGCCACAGGCCTGTTGAAGGGCTGGGAAGTTCTG
GTGGCACCTGCCTCATGCTTGGTGAGTGAGCTCTGCCCCTATTCTTTCCTGTTTAGAGAA
CAGGTTTTGATGTCCATTTTTCAAGGCAAGAATCAATAATCCCCTGCCCCATCAGGTGAC
CCCTCATGCCTGTCCCACCCCTTTATCGACTGACCTCAGCTCAACAGGCCAGTTCCCAAA
GGTCAGTGGGCAGAGGAGGGGAGACCCGCTGGTGCCATGAAGGGCCTTCCACAGGCCTGG
TGCCCTGGGGTGGACGAGGTCCCCACTTTGGGAAAAGCCCCTAGCACACTACCTGGTGCA
GAGCAGGGGCTCAACAGCAGTAGCTTTTACTTCATGGTCACCGCCAGTTTCCTCTGTAAG
CAGACGTTGGAGCTAAAGTGTGTCAAGTCCCAGCACAGAAATATACATACAGCAGGTGCT
TATAAATGGCAGCTGTCATTGTGGTTATTCTTTACCCCCCATCCCAGTTCTGCTCTCCCC
CCTCCTGGTGTGAGGGGTAGCTGTCTCCTAGGACCCCAACTCCTACCTCTGCTGCAGCCC
CAGGGACATCCCAGATCCAGAATGTTCTGAGAGGTCAGCAGTCCACACCCACATCCGACA
GAGCAGGAGCCGGACATGGTGTTAGAACCCAGGTCTCCGCTGAGCCTGTGAGCTCCCAGG
CTGCACACGGCTCTGGGGCAGAGAAGTACAGCCGGGGTCAGGGAATGACACCCTGAGGGG
GCAGGGTTATCACGTTCCCGGCACCCAGCCCTGGCCCAGTGCCCCCAGCTCCAGGGCATG
GGGTCTTTTGATCATTGCAGCAGTCAGAGCAGCAGTGTTCCTCTTCACACATGGTGGTGG
GGCACATGGCTTTGAGTGGAGGTCAGGACTCCCTGGAGTTTGTTGGAGGGGTGTCTACAC
TGGCCTCAGAGGATGGTGATGGTCAGAGGCAGCACAAAGGGGGCCGTTCCTGTTCCTCTG
AGGACCTTACATATCCTCTTGGTGCCTCAGTTTCCTGGAAAGGGAAAATAATAGTAAGGT
TATTGTGAGGATCATGTAAGTTCCTATATTCAGGCACTTAGAAGGAGCCTGGCACGTCTA
AGAGCAGCCTGGTTTTATCATTGCTGCTGTGGTTAATGTGCTTCCCATGTGTATTAGTCA
GGGTTGTCCAGAGACACAGAACCAATAGGATGTGTGTCTATGTTTACATTTATATCTACA
AATACATACATATACCCCACATAGTGGGATATTTATCCTAAGGAATTTGCTTACATATTG
TGGGGTGGACTGAAATCTGCAGGGCAGGCTGGGAGGCTGGGATCTGGCAGGCTTTGATTT
GATGTCATGGTCTTGAGTATGAAGGCAGTCTAGATGCAGAATTCTTTCTCGGGGGACCGC
CATCTTTTTTTTAAGGCCTTCAACTGATTGAATGAGGCCCACCCCCCATTATAGAGGGTA
ATCTGCTTCACTGAAAATCTATTGATGCAAAAGTTAATCACATCTATCAAGTACTTTCAG
GGCAGCATTTAAACCCATGTCTGAGCAAACACCTGGGCACCGTAGCCTAAACAAATCTAC
ATGTGAAATTAACTCTCACAGGGGCCTCTAGGGTGGGGCTAGGAAAGGGAAGCATATCTC
CTCAGAGGTGACCTTGGCTTTGTCTCTCAG**GCTTGGAGTTTATTCAGAAAAGCCAGCTGG**
CCCAGCCTGGGGGGCGGTTGGTGGTGCTGCTGCCGCTGGTGGGAGGGTACAGCCGGGCCC
TCAACGCCGCCTGCCAGCACCTGGCGAGGACGGGGGCAGTGCTGGTGGCCGCAGCCGGCA
ACTTCCGGGACGACGCTTGCCTCTACTCCCCAGCCTCGGCTCCCGAGGTGGGTGCTCCAG
GAGTACGGGAAGGTGGCAGGTGGGCCCCTGTGGGCTTCATGGGGTGCACTCCTGAACTAG
CCTGGCTTTGCAGGGAGGTGTCAGAGACTCCCAGGGCTGAGCCTGGACAGGGAAAGGGCT
TGAACCTTCAGCATTCTCATCTATAAACAGCACCATCCTCAACTCTCTCCCTTCCCCGCA
AAGCAGCCCCGCCCTCACGCCCTGCCCCTCTCCCTCTGAATGTCTCCTGAGTCCTCCGGC
CCCTTCTCCCCATGCCCATCACCTCCACCTGGCCCCTATCTACTCTCCCCTGGGTTGACA
ACACAGCTCCCTCAGCTTTCTCCTGGCCTCCCTCTGCTCCCTCCCCCAGACCACCCTGTA
AGGGCCTAGGGGCTCTGCCCACATCACTCTCCTGCCTGGTACCCCCAGGGCCTCCCTCCC
CACTATTCCCCTCCCACTCAGAGTTTCCCTGAGGCCTGGGTTGAGGGTCCAGGTGCATCC
CAGGCAGGGGGGCTACGTGAGCACAGAGAAGATGACTCTGACCCCGAGGGGCTGACTCAG
TGGGGCCCATGCCCGTCTATTCCTCTGACCAACATGCGAGTGCACCTACTGGGTGTTGGG
TGATTTGAGCACTGGGGGTACCAAGGGGAAGGAATCTCATCCCACTTCAACGACTTCACA
GTCTTGGGGGGGATGTTGGGGGCAGGGGACTTGTGGGGGCACAGATGTGAGCCTGACAGT
GCTGGGTCACCTTCCCTGACTGGTGGATTTAAAATCACATAAAGCAGGCAAAATCCAGCA
TGTCCTCCCCACCTTGCTGGCTCTGTTTTTCTCCACAGCACTTATAATCGTCTCATGCAC
TGTGTGGTTTACTGTTTGTTTACTGTCTGGGTCCCCCACTAGAATGTAAGCACCTCAGGG
GCTTCAGGAATGGGTCTTGGCCAGTGGTAGGGACAGAGGGCCTCACCAGGGCTGGGAGGG
CCAGGGCTCTGCCTGGGGAGTCAGATTTCCCTCAGGAGGGGTATTTGAATGGGACCCAAG
CAGGTGTGTAGGAGGTAGTCAGCCTGGCCGGCAAGGTCTCAGTCTATTCCTATAATCTCT
TCCCTTGCCACCCACCCCTCTCCTCTCCAG**GTCATTACTGTTGGGGCCACCAATGCCCAA**
GACCAGCCAGTGACCCTGGGGGTCCTGGGGACCAACTTCGGCCGCTGCGTGGACCTCTTT
GCCCCGGGGGACGACATCATTGGTGCCTCCAGCGACTGCAGCACCTGCTTCACGTCACAG

AGTGGGACGTCACAGGCTGCCGCCCACGTGGCTGGTGAGTTGCTGCCCTACCACCTCAGC
CACCGTGATTCTAACCACCCCTTTGGGAGCCAGGATCTGCGCCAGAACCCCATGTGCCAG
GCTCTGTGTTGGACACGGGGGACTAAAGAGGAATCAGACTGATGGTGCCCTCAAAGACTC
TCAGTCTGATGGGTGAGGCAGGTGCACAAACAGAGTAGCCAGGGCTGTGTGGAAGGGAGC
CCAGAGAGGTACCCACCCAGCTTAAAGGTCAGGGAAAGCTTCCTAGCATTTTATTTGGGG
TTTGGTGGATGAATAGGAGTTTACCTGGCAAGCAAAACAGCAATAGTCAAGGCTCAGAGG
TATGGGAGCAGGATGTAAGATAGTCTTACTCTTTGGCTGTCTTTTAACCTGGGGTTGCAG
GTCTTTTAACTTCTGAGGAACAGCCTGGTGTGTCTCTGTGCATGTGTGTGTGTGTGTGTG
TGTGCGCGCGCACGCGTGTGTGTACCAAGAGAGGAGTCCCAGATCCGGAAAGAGGGCCAG
GCCACCACTATCTCTCACTGCCCGTCCCACCACCAG**GCATTGTGGCCATGATGCTGACGG**
CCGAGCCGGAGCTCACCCTGGCTGAGCTGAGGCAGAGACTGATCCATTTCTCTGCCAAAG
ACGTCATCAACAAGGCCTGGTTTCCCGAAGACCAGCGGGTGCTGACCCCCAACCTGGTGG
CCACACTGCCCCCCAGAACCTATAAAGCAGGTCAGCAGGGCGGCAAGGTGGGCAGAATCC
AGACTGGGGCTTGGGGGGTCTCGGGAGGTCTGTGTGACCTGGGTAGGCTTGTCCATCCTC
ATCTGTGGAGGGAGAATTACACCAGAGGTTCCTAGAAATGGGAGGAGATGCATAGAAGAG
GCTCAGAAAGGGCTTGGCAGGGCGTTCATGATGTTTTGATGGAAAAATTGATCATGTTCT
TTAAGGCTGCTCTCCCTGACCAGGAGCCAAAGGTCTGGCGTCCCCTGTGAGCAGAGCCCT
GACGGAGGCTCCGCTCCCCAGCGCCCCTTCTCACCCCGGGGCCTTGTTGCAG**GTGGGACG**
CTGTTCTGCAGGACCGTGTGGTCTGCACACTCAGGACCCACGCGGATGGCCACGGCTGAG
GCCCGCTGCACAGCCCCTGAGGAGCTTCTGGGCTGCTCCAGCTTCTCCAGGAGCGGGAGG
CGGCGGGGCGAGCGCATTGAGGTGACCTGCAGGCCCCCGTCGGAGCCTGAAGTGGGGTTC
TCGCTTCCAGGTCCAGATCCGCCTGAGCCCCTTCCTCTGCTGAGCTCCAGGCGCCCGCCT
GCAAGTTAAAGCAGGATGGGGCACGTCTCAGTCACATGGCTGGGTGCTGCTGCAGGGAGC
CACACTGAGGTTTCCCAGGAGACTGCAGGACGGTGGCTAGATGGATTCCAGCGACCGACC
GTCTGGGGAGCGGGAGGGCTGGGCATGGGCCAGGGACTCGCTGCCTCTGGACTCACTGGT
CCCCAGGGCTCTTTCACTCAGATGTTACATAGTTCCAGCAGCTGAGAAATCTTCTCAAAC
CAGCAGCAGAGGGGACTTGATATTAAGGCCACAGAGCCTTACAGAGATGCCAACTGGCCA
GGGCGTTTTGGTGGAAGGACAGTGCCTCGGCCAGGAGGACGGGGTGGGCAGGCATTTCTG
CCTGGGAGACGGTGTCTGGGAGTGTGTGTGACCATGCACTTGATCCTGCAAGTGAGAGTA
TGTGGGCGGCGTGGCCGAGAGCAGGTCAGGGCTGAGGAGGCGGGGGCCTTGCTCGGGGTC
TTAGGTTTCCCTGTATCTGCATTTTATGGTCATGCTTAGAGCCCAGAAGAACTTTATTAC
ACACAGCTGCCCATGTGCTGAGCAGTTTGCAGGAGGGAGGTCCTGGTCCTCAGAGGGGCA
GGCTCCTGGCAGGGACGGTGGAGATGGTATGAGGGACTGGGACCAGCTGCTTGAGCCTGT
CCCTTTCAGCCCCCTCATTCTGTGTTTCAAAGCCCTTTCTAAAGCATGTTTCTGTTTCTG
TCTTTGGCTTTCAG**GCCCGAGGGGGCAGGCATGTCTGCCTGGCCCACAATGCGTTTGGGG**
GTGAGGGTGTCTATGCCGTTGCCAGATGCTGCCTGCTGCCCCAGGCCAACTGCAGTGTCC
ACACAGCTCCGCCAGCCAGGGCTGGTGTGCTGACCCAAGCCCACTGCCACCAGCAGGGCC
ACGTCCTCACAGGTAGGAGGCTGGGCCCATCCTGGGGTGAAGAGGCTTCCTTGTCTCCTG
GTGCACCTGCTCCCACCTGACTGGTCCCATGCTGGGGCCCAACTGCCTGGTGCGAAGGCC
TGTGCTACCCTTCCATCCCTGTGACCCTGGGTGGGCACCTCATTGGTCTCAGTCTCAGCT
TCTTCCTCCCTAAGAAGAATGACGGTAGTTCCTGCCTCAATGGGTTGCCATGGAATGAGT
AAGCCCTAGAGCACCAGGCCTGGAGCATCCAGGGCACTTTCTGACAGTGTGTGAGGGGCA
GTTCAGGCTCAGGCCAGTGTCTCGTTCCTGCCCTGACTTATTTCTGGGTTTCCCAGCTCC
AGCCCCAGACCCGAAAGAGATGGAGTCTGAATGGGGTGGGGAGGACAGACAGATGGTCCC
ACAGCATCCAGGTGTCTGAGCTGGCCCTCCTTTGCCCCAG**GCTGCAGCTCCCACTGGGAA**
GTGGAGGAATTTGGCACCCATGGGCCACCTGTGCTGAGGCCACGAGGTCAGGCTGATCAG
TGTGTGGGCCACGCGGAGGCCAGCGTCCATGCCTCCTGCTGCCACTCGCCAGGTCTGGAG
TGCAAATTCAGGGAGCACGGGATCCCGGGCCCTGCGGAGAAGGTGAGAGGCGTGCTGGGC
GGGGGACCGGGACGAGAGCCTGACACCCCAAGCGGTGGCCTGTGTCCCTCCTGTGCCACT
TTTCTGTGTCAGCATTTGTGTGCCCACCACACCCTCACAGATCTGGGGGGTGGTTTGTGG
GCTGGTCGCTGTTGGCGGCTTTTGCAGCTGTGTGGACAGCGTGTGCATGTGTGCTCCTCT
GTGGCTGGGCCAGGTTTTGCTTTTGTCTAGTTTAGCGAGGTTTGCTCTCTGGGGCACCCT
GCCCCTCCCTTGCAGAGAATATGACAAATGTTGCATAAGGAAGATCAGCCCACATGCATT
CACTGGTTCATCCACTCAGCACATCTGCTGGGAGGATGACTCAGCCGTGACCAAGAGGAG
GGGACACCTGAGCTAGGGAGCAGCTAGCGGGGCCAGAGAGGCAAGGGAGGGTGTGCAGAG
AGGGCGGGAGCCAGCTCTCAGAAACCACCCGTGCCAAGTGCAACCTGCGGCTTCTCTGTA
AGTCTCCTTTTAAAAGCCACAGGGAACTTCTTCAAAGGAAGCCCTGCAGAGTTCACTTTT
AAATGAACTGGAAGAGGTTTTTAAGAGTGTGAGTCTGTGCTGATTGTGTTCTGCATGCTG
CATTTCTGGAGGGCAAGGGCTGTTCCAGGTCCACTTGCTCAGCAAATGTTGAGGCCTGTG
GCATCCCAGGCAATGTTCCAGGCGGTGGGGATACAAACCCGACTAGCTTTCTCTCCTGGC
GCGTCCAGTCTAATGGGGGAGAAGGACAGCAAACAAATAAGTAACTATAGAGTAATTAAA
ACATGCTATAGAGGAAAGTAAAGCAGGGAAGGGAATGGGAGGGTCCTTCAGGAGAGGCCT
CCTTGAGAAGGTGGGGGACATCACAGGGAACAGTGTTCAAGGCAGAGGGGGTAGCCAGGG

CAAAGGCCCTGAGGTGGGAGTGGGCTTGGAGAGCAAAAGGAAGAGCCAGAGGGCTGGTGA
GGTGGGACCCGAGTGGGAGGGGGAACCAGAGACAGGGTTTAGGTGGGGCCGGAGGGCCAC
AGGAAGGACTTGGATTTTTACTGGAGTGAGCTGGGAGCCACACAGGGTTCTGAGCCTGGG
TGTGGGGAGGGGGGTGGGCTATCTGACCTGGGTGTGAGCAGGTTCATTCTGGTCGCTGTG
TCGGGAAGACTGCAGGGGACAGGGCGGAAGCAGGGAGGCCCGCTGTAGACGGGTGGACAG
CCCGGGTGCTGGGGGGTCCGTCAGGGCGGGAGTGTAGAGGATGCTGGAATCTGAAGGAGG
GGCTGCACATCTGATGGCCTGGATATTGGGGGAGCAGTGGAGGGGGCGTCCAAGGGTTTT
GCTTTGCTCTCGGACGAATGGCATCGCCCCTGACTGGGATGGGAAGGGCTGTGAGAGGTC
AAGTGTCGGGGAAGTTGAGGCATTTATGCGGGCCTGGCTCACAGCGTGCCGTGCCTTACA
TGTGCTTTCTTTTGTCCCCGGGCCCTGGCAG**GTCACCGTGGCCTGCAAGGAGGGCTGGAC**
GCTGACCGGCTGCGGGGCCCACCCCGGGGCCTCCCACACCCTGGGGGCCTATGCAGTGGA
CAACACGTGTGTGGTGAGGGGCCGGGACGTGGGTGTGCGAGGCAGGACGGGTGAGGAGGC
CGCCGTGGCCATTGCCATCTGCTGCAGGAGCCGGTCAGGGGAGCAGGCCTCCCCGGGGAC
CCAGTGACAGCCCCGCCCAGGATATCTGCGTGGCTGGGGTCCCAGGCCTTGGCTGAGCTT
TGAAGTGCTTCCTTTTTCCTCCTTCCTCAGCCCTCCTCAGCCTGGGCCCCGGGGGACAGA
AGGCACCTCTTTCTCCTGGAGCTCTGGTGCTGGCACTTGGGGTACACTGGCTCCCTGCCT
GGGAGAACCCCATCTCTTGGCCCGAGTCACCCCTCCCCAGACCCGAGCTGAGTGGGAGGT
TGAATGAGCAGGGCCACAGGCGCCGGCAGCCCCTCCCTCACTGAGGGGCTGTGTCCACAT
GTCCATCAACAAGGGTCTGGCTGTGCTCAGCTCCCTGTCAGCTGCTCCCAAGTTGCCAGT
GCTGTGGGCAGAATTAGCTTTTGTTGAGTTCTTGCTACATGTCAGCCAGGCAGTCAGTCC
TCAGGCCTCCATGAAGGAGGTGGTAACCCTCCTATGGGGAGGCAAGGAAGCACTTGACGG
CTGGGAGAGGCCAAATGTTGGTCAGAGGATGTGAAAGGTGGAAATGGCCCCTCACCTCCT
GCCCACTCTGGGGAGGCCCGGTTGGGCTCCCTGATTATGGAGATGAGTTTTCCATGCCTC
TGGGGAT
