# Supplementary material for: Molecular evolution of PCSK family: Analysis of natural selection rate and gene loss
Source: PLoS One. 2021 Oct 28;16(10):e0259085. doi: 10.1371/journal.pone.0259085 (PMC8553125; doi:10.1371/journal.pone.0259085)
Supplement: S1 Table — A total of 6 members of the proprotein convertases family have been analyzed in 45 species. Proteins accession numbers are shown. (DOCX) [file pone.0259085.s038.docx]

**S1 Table**. **The overall distribution of the proprotein convertases family in mammalian**

| species | Common name | Order | Family | *Pcsk1* | *Pcsk3* | *Pcsk5* | *Pcsk7* | *Mbtps1* | *Pcsk9* |
| --- | --- | --- | --- | --- | --- | --- | --- | --- | --- |
| *Monodelphis domestica* | Gray short-tailed opossum | *Didelphimorphia* | *Didelphidae* | XP_001364000.1 | XP_007479359.1 | XP_003341693.1 | XP_001380854.1 | XP_003339821.3 | XP_016284974.1 |
| *Sarcophilus harrisii* | Tasmanian devil | *Dasyuromorphia* | *Dasyuridae* | XP_003759876.1 | XP_031811494.1 | XP_031795501.1 | XP_003764243.1 | XP_003758493.1 | XP_023357465.1 |
| *Camelus dromedarius* | Dromedary | *Artiodactyla* | *Camelidae* | XP_010992267.2 | XP_031296449.1 | XP_031305762.1 | XP_010979708.1 | XP_031313843.1 | XP_031321060.1 |
| *Balaenoptera acutorostrata scammoni* | North Pacific minke whale | *Artiodactyla* | *Balaenopteridae* | XP_007197531.1 | XP_007194370.1 | XP_007182377.1 | XP_007196626.1 | XP_007182633.1 | XP_007164449.2 |
| *Lagenorhynchus obliquidens* | Pacific white-sided dolphin | *Artiodactyla* | *Delphinidae* | XP_026956372.1 | XP_026945195.1 | XP_026961465.1 | XP_026938026.1 | XP_026974938.1 | XP_026964210.1 |
| *Orcinus orca* | Killer whale | *Artiodactyla* | *Delphinidae* | XP_004263564.1 | XP_012391653.1 | XP_004276424.1 | XP_004273407.1 | XP_004280142.1 | XP_004273850.1 |
| *Delphinapterus leucas* | Beluga whale | *Artiodactyla* | *Monodontidae* | XP_022448143.1 | XP_022419714.1 | XP_022429193.1 | XP_030615446.1 | XP_022438760.1 | XP_022431480.1 |
| *Neophocaena asiaeorientalis asiaeorientalis* |  | *Artiodactyla* | *Phocoenidae* | XP_024593446.1 | XP_024622254.1 | XP_024594744.1 | XP_024607427.1 | XP_024591387.1 | XP_024595024.1 |
| *Ovis aries* | Domestic sheep or mouflon | *Artiodactyla* | *Bovidae* | XP_004009137.1 | XP_027812787.1 | XP_004004349.1 | XP_027835032.1 | XP_027833477.1 | × |
| *Bos taurus* | Domestic cattle | *Artiodactyla* | *Bovidae* | NP_776837.1 | XP_024837364.1 | XP_024851952.1 | NP_001179907.1 | NP_001020501.2 | × |
| *Galeopterus variegatus* | Sunda flying lemur | *Dermoptera* | *Cynocephalidae* | XP_008567361.1 | XP_008563303.1 | XP_008588460.1 | XP_008592252.1 | XP_008563588.1 | XP_008568363.1 |
| *Marmota flaviventris* | Yellow-bellied marmot | *Rodentia* | *Sciuridae* | XP_027783573.1 | XP_027775791.1 | XP_027795946.1 | XP_027786344.1 | XP_027788809.1 | XP_027800824.1 |
| *Cavia porcellus* | Guinea pig | *Rodentia* | *Caviidae* | XP_003479481.2 | XP_013014060.1 | XP_003472270.2 | XP_013010968.1 | XP_003462008.1 | XP_023418328.1 |
| *Octodon degus* | Degu | *Rodentia* | *Octodontidae* | XP_004625484.1 | XP_004623373.1 | XP_023578735.1 | XP_004636532.1 | XP_004646620.1 | XP_004642652.1 |
| *Chinchilla lanigera* | Long-tailed chinchilla | *Rodentia* | *Chinchillidae* | XP_005382289.1 | XP_005381565.1 | XP_005408366.1 | XP_005378255.1 | XP_005413285.1 | XP_005398562.1 |
| *Heterocephalus glaber* | Naked mole-rat | *Rodentia* | *Bathyergidae* | XP_004841764.1 | XP_012930558.1 | XP_004862586.1 | XP_004856716.1 | XP_004842832.1 | XP_004869692.1 |
| *Grammomys surdaster* |  | *Rodentia* | *Muridae* | XP_028641819.1 | XP_028610617.1 | XP_028625905.1 | XP_028639494.1 | XP_028608241.1 | XP_028641261.1 |
| *Mus musculus* | House mouse | *Rodentia* | *Muridae* | NP_038656.1 | NP_001074923.1 | XP_006526822.1 | NP_001268863.1 | NP_001161382.1 | NP_705793.1 |
| *Mus pahari* | Gairdner's shrewmouse | *Rodentia* | *Muridae* | XP_021064061.1 | XP_021054540.1 | XP_029391286.1 | XP_021063444.1 | XP_021075771.1 | XP_021056523.1 |
| *Rattus norvegicus* | Norway rat | *Rodentia* | *Muridae* | NP_058787.1 | XP_008757777.1 | NP_446275.1 | NP_062119.1 | XP_006255785.1 | NP_954862.2 |
| *Peromyscus maniculatus bairdii* | Prairie deer mouse | *Rodentia* | *Cricetidae* | XP_006986611.1 | XP_006970056.1 | XP_015844862.1 | XP_006989908.1 | XP_015863908.1 | XP_006987259.1 |
| *Microtus ochrogaster* | Prairie vole | *Rodentia* | *Cricetidae* | XP_005356618.1 | XP_005357706.1 | XP_005352083.1 | XP_005347301.1 | XP_005345804.1 | XP_013203947.1 |
| *Urocitellus parryii* | Arctic ground squirrel | *Rodentia* | *Sciuridae* | XP_026241646.1 | XP_026243886.1 | XP_026249805.1 | XP_026252096.1 | XP_026267183.1 | XP_026238164.1 |
| *Nannospalax galili* | Northern Israeli blind subterranean mole rat | *Rodentia* | *Spalacidae* | XP_008826199.1 | XP_008850996.1 | XP_008845451.2 | XP_029410827.1 | XP_029416894.1 | XP_008831203.1 |
| *Cebus capucinus imitator* | white-faced capuchin | *Primates* | *Cebidae* | XP_017364352.1 | XP_017361590.1 | XP_017398739.1 | XP_017378864.1 | XP_017399086.1 | XP_017399475.1 |
| *Nomascus leucogenys* | White-cheeked gibbon | *Primates* | *Hylobatidae* | XP_030673054.1 | XP_012362574.2 | XP_003267477.1 | XP_030650663.1 | XP_003272532.1 | XP_003265178.2 |
| *Homo sapiens* | Human | *Primates* | *Hominidae* | NP_000430.3 | NP_001276752.1 | NP_001177411.1 | NP_004707.2 | NP_003782.1 | NP_777596.2 |
| *Pongo abelii* | Long-tailed macaque | *Primates* | *Cercopithecidae* | NP_001126279.1 | XP_002825875.1 | XP_002819920.2 | XP_024110500.1 | XP_005592718.1 | XP_002810823.2 |
| *Macaca fascicularis* | Sumatran orangutan | *Primates* | *Hominidae* | XP_005557471.1 | XP_005595531.1 | XP_005582010.1 | XP_015291255.1 | NP_001126959.1 | XP_005543314.1 |
| *Papio anubis* | olive baboon | *Primates* | *Cercopithecidae* | XP_003899990.2 | XP_009198206.3 | XP_031509996.1 | XP_003910793.2 | XP_003917293.1 | XP_003891996.2 |
| *Chlorocebus sabaeus* | green monkey | *Primates* | *Cercopithecidae* | XP_007977229.1 | XP_007988647.1 | XP_007967725.1 | XP_008019195.1 | XP_007992399.1 | XP_007976798.1 |
| *Propithecus coquereli* | Coquerel's sifaka | *Primates* | *Indriidae* | XP_012494300.1 | XP_012499190.1 | XP_012506123.1 | XP_012513787.1 | XP_012503632.1 | XP_012494949.1 |
| *Loxodonta africana* | African bush elephant | *Proboscidea* | *Elephantidae* | XP_003405038.1 | XP_003413924.1 | XP_003407430.1 | XP_003418311.1 | XP_003418130.2 | XP_010589541.1 |
| *Chrysochloris asiatica* | Cape golden mole | *Afrosoricida* | *Chrysochloridae* | XP_006867949.1 | XP_006867209.1 | XP_006834991.1 | XP_006833920.1 | XP_006860193.1 | XP_006839877.1 |
| *Leptonychotes weddellii* | Weddell seal | *Carnivora* | *Phocidae* | XP_006733345.1 | XP_006751496.2 | XP_030886696.1 | XP_030877931.1 | XP_030895564.1 | × |
| *Ursus maritimus* | Polar bear | *Carnivora* | *Ursidae* | XP_008699127.1 | XP_008686077.1 | XP_008689256.1 | XP_008688257.1 | XP_008687978.1 | × |
| *Mustela putorius furo* | European domestic ferret | *Carnivora* | *Mustelidae* | XP_004769985.1 | XP_004763759.1 | XP_004772185.1 | XP_004749965.1 | XP_004753962.1 | × |
| *Felis catus* | Domestic cat | *Carnivora* | *Felidae* | XP_003981208.1 | XP_023110662.1 | XP_023099123.1 | XP_023094910.1 | XP_006941834.1 | × |
| *Rousettus aegyptiacus* | Egyptian rousette | *Chiroptera* | *Pteropodidae* | XP_016015271.1 | XP_016007023.1 | XP_015982575.1 | XP_015990398.1 | XP_016021416.1 | × |
| *Pteropus vampyrus* | large flying fox | *Chiroptera* | *Pteropodidae* | XP_023378654.1 | XP_011374454.1 | XP_011376197.1 | XP_011381475.1 | XP_011383704.1 | × |
| *Eptesicus fuscus* | big brown bat | *Chiroptera* | *Vespertilionidae* | XP_008142748.1 | XP_008158267.1 | XP_008140288.1 | XP_008148347.1 | XP_008137936.1 | × |
| *Miniopterus natalensis* | Natal at long-fingered bat | *Chiroptera* | *Miniopterus* | XP_016071637.1 | XP_016072648.1 | XP_016078327.1 | XP_016060220.1 | XP_016052532.1 | × |
| *sorex araneus* | Eurasian shrew | *Soricomorpha* | *Soricidae* | XP_004613144.1 | XP_004617631.1 | XP_004613037.1 | XP_004605122.1 | XP_004600559.1 | × |
| *Elephantulus edwardii* | Cape elephant shrew | *Macroscelidea* | *Macroscelididae* | XP_006885437.1 | XP_006885215.1 | XP_006892053.1 | XP_006890875.1 | XP_006888860.1 | XP_006879886.1 |
| *Erinaceus europaeus* | Western European hedgehog | *Erinaceomorpha* | *Erinaceidae* | XP_007529973.1 | XP_016043802.1 | XP_016049477.1 | XP_007523036.1 | XP_007528419.1 | × |

A total of 6 members of the proprotein convertases family have been analyzed in 45 species. Proteins accession numbers are shown
