# Supplementary material for: Molecular evolution of PCSK family: Analysis of natural selection rate and gene loss
Source: PLoS One. 2021 Oct 28;16(10):e0259085. doi: 10.1371/journal.pone.0259085 (PMC8553125; doi:10.1371/journal.pone.0259085)
Supplement: S2 Table — (DOCX) [file pone.0259085.s039.docx]

**S2 Table.** **The characteristics of hits found for PCSK9 Blat analysis in the indicated species with putatively lost PCSK9**

| **Identity with *homo sapiens* *PCSK9* (%)** | **Length of nucleotides** | **Location** | **Chromosome** | **Species** |
| --- | --- | --- | --- | --- |
| 87.61 | 339 | 91294798-91294920 | 3 | *Bos taurus* |
| 86.67 | 270 | 91293072-91293169 | 3 | *Bos taurus* |
| 91.67 | 144 | 6833708-6833760 | Scaffold GL896928.1 | *Mustela putorius furo* |
| 87.04 | 162 | 3983451-3983508 | Scaffold KK498648.1 | *Ursus maritimus* |
| 83.78 | 333 | 29158676-29158796 | 1 | *Ovis aries* |
| 88.89 | 135 | 44838696-44838745 | C1 | *Felis catus* |
