# Supplementary material for: Molecular evolution of PCSK family: Analysis of natural selection rate and gene loss
Source: PLoS One. 2021 Oct 28;16(10):e0259085. doi: 10.1371/journal.pone.0259085 (PMC8553125; doi:10.1371/journal.pone.0259085)
Supplement: S3 Table — (DOCX) [file pone.0259085.s040.docx]

**S3 Table. The genomic positions of Usp24 and BSND genes in the indicated species with putatively lost PCSK9 gene**

| **chromosome** | **BSND position** | **Usp24 position** | **species** |
| --- | --- | --- | --- |
| Unplaced scaffold | NW_006384712.1  (320710-334462) | NW_006384712.1  (346197-525150) | *Leptonychotes weddellii* |
| Unplaced scaffold | NW_023416284.1  (36363726-36380344) | NW_023416284.1  (36379043-36524554) | *Rousettus aegyptiacus* |
| Unplaced scaffold | NW_011888951.1  (1846892-1856261) | NW_011888951.1  (1728332-1826674) | *Pteropus vampyrus* |
| Unplaced scaffold | NW_007370660.1  (205718-219896) | NW_007370660.1  (36994-188466) | *Eptesicus fuscus* |
| Unplaced scaffold | NW_015504362.1  (523430-532325) | NW_015504362.1  (535861-643504) | *Miniopterus natalensis* |
| Unplaced scaffold | NW_004545881.1  (21504440-21511670) | NW_004545881.1  (21389182-21488287) | *sorex araneus* |
| Unplaced scaffold | NW_006804007.1  (4249472-4263052) | NW_006804007.1  (4070951-4243174) | *Erinaceus europaeus* |
