# Supplementary material for: Molecular evolution of PCSK family: Analysis of natural selection rate and gene loss
Source: PLoS One. 2021 Oct 28;16(10):e0259085. doi: 10.1371/journal.pone.0259085 (PMC8553125; doi:10.1371/journal.pone.0259085)
Supplement: S4 Table — (DOCX) [file pone.0259085.s041.docx]

**S4 Table** . **The characteristics of hits found for PCSK 9 remnants in the indicated species with putatively lost PCSK9, using Blastn of PCSK 9 query against interval genomic sequences of USP24 and BSND genes**

| **Identity to *PCSK9* (%)** | **Quary Cover (%)** | **Numbers of matching segments** | **Numbers of hits** | **species** |
| --- | --- | --- | --- | --- |
| 67.13 | 40 | 8 | 1 | *Leptonychotes weddellii* |
| 79.9 | 12 | 3 | 1 | *Rousettus aegyptiacus* |
| 85.79 | 16 | 3 | 1 | *Pteropus vampyrus* |
| 75.12 | 14 | 6 | 1 | *Eptesicus fuscus* |
| 75.73 | 5 | 1 | 1 | *Miniopterus natalensis* |
| 95 | 0 | 1 | 1 | *sorex araneus* |
| - | - | 0 | 0 | *Erinaceus europaeus* |
