# Supplementary material for: Molecular evolution of PCSK family: Analysis of natural selection rate and gene loss
Source: PLoS One. 2021 Oct 28;16(10):e0259085. doi: 10.1371/journal.pone.0259085 (PMC8553125; doi:10.1371/journal.pone.0259085)
Supplement: S5 Table — Mutations with PROVEAN Score less than -2.5 are predicted to be deleterious. mutations with SIFT score less than 0.05 are predicted to be deleterious, while those greater than 0.05 are neutral. (DOCX) [file pone.0259085.s042.docx]

**S5 Table: Provean and SIFT prediction of deleterious positive selections.**

| VARIATION | | PROTEIN SEQUENCE CHANGE | | | | PROVEAN PREDICTION | | | | SIFT PREDICTION | | | |
| --- | --- | --- | --- | --- | --- | --- | --- | --- | --- | --- | --- | --- | --- |
| ROW_NO. | INPUT | PROTEIN_ID | POSITION | RESIDUE_REF | RESIDUE_ALT | SCORE | PREDICTION (cutoff=-2.5) | #SEQ | #CLUSTER | SCORE | PREDICTION (cutoff=0.05) | MEDIAN_INFO | #SEQ |
| 1 | NP_000430.3,641,L,P | [NP_000430.3](http://www.ncbi.nlm.nih.gov/protein/NP_000430.3) | 641 | L | P | -0.52 | Neutral | [184](http://provean.jcvi.org/view_supporting_seqs.php?pid=NP_000430.3) | 30 | 0.244 | Tolerated | 3.26 | 69 |
| 2 | NP_000430.3,641,L,P | [NP_000430.3](http://www.ncbi.nlm.nih.gov/protein/NP_000430.3) | 641 | L | P | -0.52 | Neutral | [184](http://provean.jcvi.org/view_supporting_seqs.php?pid=NP_000430.3) | 30 | 0.244 | Tolerated | 3.26 | 69 |
| 3 | NP_000430.3,641,L,Q | [NP_000430.3](http://www.ncbi.nlm.nih.gov/protein/NP_000430.3) | 641 | L | Q | 0.05 | Neutral | [184](http://provean.jcvi.org/view_supporting_seqs.php?pid=NP_000430.3) | 30 | 0.333 | Tolerated | 3.26 | 69 |
| 4 | NP_000430.3,641,L,Q | [NP_000430.3](http://www.ncbi.nlm.nih.gov/protein/NP_000430.3) | 641 | L | Q | 0.05 | Neutral | [184](http://provean.jcvi.org/view_supporting_seqs.php?pid=NP_000430.3) | 30 | 0.333 | Tolerated | 3.26 | 69 |
| 5 | NP_000430.3,641,L,V | [NP_000430.3](http://www.ncbi.nlm.nih.gov/protein/NP_000430.3) | 641 | L | V | -0.11 | Neutral | [184](http://provean.jcvi.org/view_supporting_seqs.php?pid=NP_000430.3) | 30 | 0.514 | Tolerated | 3.26 | 69 |
| 6 | NP_000430.3,642,V,T | [NP_000430.3](http://www.ncbi.nlm.nih.gov/protein/NP_000430.3) | 642 | V | T | -0.23 | Neutral | [184](http://provean.jcvi.org/view_supporting_seqs.php?pid=NP_000430.3) | 30 | 0.445 | Tolerated | 3.26 | 70 |
| 7 | NP_000430.3,642,V,I | [NP_000430.3](http://www.ncbi.nlm.nih.gov/protein/NP_000430.3) | 642 | V | I | -0.34 | Neutral | [184](http://provean.jcvi.org/view_supporting_seqs.php?pid=NP_000430.3) | 30 | 0.252 | Tolerated | 3.26 | 70 |
| 8 | NP_000430.3,642,V,A | [NP_000430.3](http://www.ncbi.nlm.nih.gov/protein/NP_000430.3) | 642 | V | A | -0.22 | Neutral | [184](http://provean.jcvi.org/view_supporting_seqs.php?pid=NP_000430.3) | 30 | 0.864 | Tolerated | 3.26 | 70 |
| 9 | NP_000430.3,642,V,M | [NP_000430.3](http://www.ncbi.nlm.nih.gov/protein/NP_000430.3) | 642 | V | M | -0.74 | Neutral | [184](http://provean.jcvi.org/view_supporting_seqs.php?pid=NP_000430.3) | 30 | 0.082 | Tolerated | 3.26 | 70 |
| 10 | NP_000430.3,679,S,P | [NP_000430.3](http://www.ncbi.nlm.nih.gov/protein/NP_000430.3) | 679 | S | P | -0.95 | Neutral | [184](http://provean.jcvi.org/view_supporting_seqs.php?pid=NP_000430.3) | 30 | 0.203 | Tolerated | 3.77 | 42 |
| 11 | NP_000430.3,679,S,L | [NP_000430.3](http://www.ncbi.nlm.nih.gov/protein/NP_000430.3) | 679 | S | L | -1.18 | Neutral | [184](http://provean.jcvi.org/view_supporting_seqs.php?pid=NP_000430.3) | 30 | 0.112 | Tolerated | 3.77 | 42 |
| 12 | NP_000430.3,679,S,F | [NP_000430.3](http://www.ncbi.nlm.nih.gov/protein/NP_000430.3) | 679 | S | F | -0.93 | Neutral | [184](http://provean.jcvi.org/view_supporting_seqs.php?pid=NP_000430.3) | 30 | 0.041 | Damaging | 3.77 | 42 |
| 13 | NP_000430.3,679,S,V | [NP_000430.3](http://www.ncbi.nlm.nih.gov/protein/NP_000430.3) | 679 | S | V | -1.22 | Neutral | [184](http://provean.jcvi.org/view_supporting_seqs.php?pid=NP_000430.3) | 30 | 0.149 | Tolerated | 3.77 | 42 |
| 14 | NP_000430.3,679,S,T | [NP_000430.3](http://www.ncbi.nlm.nih.gov/protein/NP_000430.3) | 679 | S | T | -0.68 | Neutral | [184](http://provean.jcvi.org/view_supporting_seqs.php?pid=NP_000430.3) | 30 | 1.000 | Tolerated | 3.77 | 42 |
| 15 | NP_000430.3,679,S,A | [NP_000430.3](http://www.ncbi.nlm.nih.gov/protein/NP_000430.3) | 679 | S | A | -0.37 | Neutral | [184](http://provean.jcvi.org/view_supporting_seqs.php?pid=NP_000430.3) | 30 | 0.514 | Tolerated | 3.77 | 42 |
| 16 | NP_000430.3,680,P,L | [NP_000430.3](http://www.ncbi.nlm.nih.gov/protein/NP_000430.3) | 680 | P | L | -1.57 | Neutral | [184](http://provean.jcvi.org/view_supporting_seqs.php?pid=NP_000430.3) | 30 | 0.451 | Tolerated | 3.77 | 40 |
| 17 | NP_000430.3,680,P,T | [NP_000430.3](http://www.ncbi.nlm.nih.gov/protein/NP_000430.3) | 680 | P | T | -0.76 | Neutral | [184](http://provean.jcvi.org/view_supporting_seqs.php?pid=NP_000430.3) | 30 | 0.488 | Tolerated | 3.77 | 40 |
| 18 | NP_000430.3,680,P,S | [NP_000430.3](http://www.ncbi.nlm.nih.gov/protein/NP_000430.3) | 680 | P | S | -0.53 | Neutral | [184](http://provean.jcvi.org/view_supporting_seqs.php?pid=NP_000430.3) | 30 | 0.202 | Tolerated | 3.77 | 40 |
| 19 | NP_000430.3,680,P,Q | [NP_000430.3](http://www.ncbi.nlm.nih.gov/protein/NP_000430.3) | 680 | P | Q | -1.02 | Neutral | [184](http://provean.jcvi.org/view_supporting_seqs.php?pid=NP_000430.3) | 30 | 0.077 | Tolerated | 3.77 | 40 |
| 20 | NP_000430.3,665,Q,E | [NP_000430.3](http://www.ncbi.nlm.nih.gov/protein/NP_000430.3) | 665 | Q | E | 0.13 | Neutral | [184](http://provean.jcvi.org/view_supporting_seqs.php?pid=NP_000430.3) | 30 | 1.000 | Tolerated | 3.79 | 42 |
| 21 | NP_000430.3,665,Q,K | [NP_000430.3](http://www.ncbi.nlm.nih.gov/protein/NP_000430.3) | 665 | Q | K | -0.04 | Neutral | [184](http://provean.jcvi.org/view_supporting_seqs.php?pid=NP_000430.3) | 30 | 0.715 | Tolerated | 3.79 | 42 |
| 22 | NP_000430.3,690,S,G | [NP_000430.3](http://www.ncbi.nlm.nih.gov/protein/NP_000430.3) | 690 | S | G | -0.69 | Neutral | [184](http://provean.jcvi.org/view_supporting_seqs.php?pid=NP_000430.3) | 30 | 0.130 | Tolerated | 4.32 | 40 |
| 23 | NP_000430.3,690,S,N | [NP_000430.3](http://www.ncbi.nlm.nih.gov/protein/NP_000430.3) | 690 | S | N | -0.95 | Neutral | [184](http://provean.jcvi.org/view_supporting_seqs.php?pid=NP_000430.3) | 30 | 0.174 | Tolerated | 4.32 | 40 |
| 24 | NP_000430.3,467,C,N | [NP_000430.3](http://www.ncbi.nlm.nih.gov/protein/NP_000430.3) | 467 | C | N | -10.69 | Deleterious | [184](http://provean.jcvi.org/view_supporting_seqs.php?pid=NP_000430.3) | 30 | 0.000 | Damaging | 2.88 | 319 |
| 25 | NP_000430.3,471,D,L | [NP_000430.3](http://www.ncbi.nlm.nih.gov/protein/NP_000430.3) | 471 | D | L | -1.97 | Neutral | [184](http://provean.jcvi.org/view_supporting_seqs.php?pid=NP_000430.3) | 30 | 0.075 | Tolerated | 2.87 | 257 |
| 26 | NP_001177411.1,1133,R,P | [NP_001177411.1](http://www.ncbi.nlm.nih.gov/protein/NP_001177411.1) | 1133 | R | P | -1.81 | Neutral | [139](http://provean.jcvi.org/view_supporting_seqs.php?pid=NP_001177411.1) | 30 | 0.154 | Tolerated | 2.82 | 59 |
| 27 | NP_001177411.1,1133,R,G | [NP_001177411.1](http://www.ncbi.nlm.nih.gov/protein/NP_001177411.1) | 1133 | R | G | -2.42 | Neutral | [139](http://provean.jcvi.org/view_supporting_seqs.php?pid=NP_001177411.1) | 30 | 0.287 | Tolerated | 2.82 | 59 |
| 28 | NP_001177411.1,1133,R,H | [NP_001177411.1](http://www.ncbi.nlm.nih.gov/protein/NP_001177411.1) | 1133 | R | H | -1.81 | Neutral | [139](http://provean.jcvi.org/view_supporting_seqs.php?pid=NP_001177411.1) | 30 | 0.103 | Tolerated | 2.82 | 59 |
| 29 | NP_001177411.1,1133,R,Q | [NP_001177411.1](http://www.ncbi.nlm.nih.gov/protein/NP_001177411.1) | 1133 | R | Q | -1.00 | Neutral | [139](http://provean.jcvi.org/view_supporting_seqs.php?pid=NP_001177411.1) | 30 | 0.320 | Tolerated | 2.82 | 59 |
| 30 | NP_001177411.1,1150,Q,R | [NP_001177411.1](http://www.ncbi.nlm.nih.gov/protein/NP_001177411.1) | 1150 | Q | R | 0.12 | Neutral | [139](http://provean.jcvi.org/view_supporting_seqs.php?pid=NP_001177411.1) | 30 | 0.558 | Tolerated | 2.82 | 59 |
| 31 | NP_001177411.1,1150,Q,P | [NP_001177411.1](http://www.ncbi.nlm.nih.gov/protein/NP_001177411.1) | 1150 | Q | P | -1.19 | Neutral | [139](http://provean.jcvi.org/view_supporting_seqs.php?pid=NP_001177411.1) | 30 | 0.360 | Tolerated | 2.82 | 59 |
| 32 | NP_001177411.1,1150,Q,L | [NP_001177411.1](http://www.ncbi.nlm.nih.gov/protein/NP_001177411.1) | 1150 | Q | L | -1.79 | Neutral | [139](http://provean.jcvi.org/view_supporting_seqs.php?pid=NP_001177411.1) | 30 | 0.304 | Tolerated | 2.82 | 59 |
| 33 | NP_001177411.1,1150,Q,G | [NP_001177411.1](http://www.ncbi.nlm.nih.gov/protein/NP_001177411.1) | 1150 | Q | G | -1.58 | Neutral | [139](http://provean.jcvi.org/view_supporting_seqs.php?pid=NP_001177411.1) | 30 | 0.304 | Tolerated | 2.82 | 59 |
| 34 | NP_001177411.1,1150,Q,H | [NP_001177411.1](http://www.ncbi.nlm.nih.gov/protein/NP_001177411.1) | 1150 | Q | H | -1.12 | Neutral | [139](http://provean.jcvi.org/view_supporting_seqs.php?pid=NP_001177411.1) | 30 | 0.098 | Tolerated | 2.82 | 59 |
| 35 | NP_001177411.1,1150,Q,W | [NP_001177411.1](http://www.ncbi.nlm.nih.gov/protein/NP_001177411.1) | 1150 | Q | W | -2.41 | Neutral | [139](http://provean.jcvi.org/view_supporting_seqs.php?pid=NP_001177411.1) | 30 | 0.014 | Damaging | 2.82 | 59 |
| 36 | NP_001177411.1,1442,I,M | [NP_001177411.1](http://www.ncbi.nlm.nih.gov/protein/NP_001177411.1) | 1442 | I | M | 0.43 | Neutral | [139](http://provean.jcvi.org/view_supporting_seqs.php?pid=NP_001177411.1) | 30 | 0.220 | Tolerated | 2.82 | 39 |
| 37 | NP_001177411.1,1442,I,R | [NP_001177411.1](http://www.ncbi.nlm.nih.gov/protein/NP_001177411.1) | 1442 | I | R | 0.81 | Neutral | [139](http://provean.jcvi.org/view_supporting_seqs.php?pid=NP_001177411.1) | 30 | 0.348 | Tolerated | 2.82 | 39 |
| 38 | NP_001177411.1,1442,I,T | [NP_001177411.1](http://www.ncbi.nlm.nih.gov/protein/NP_001177411.1) | 1442 | I | T | 0.45 | Neutral | [139](http://provean.jcvi.org/view_supporting_seqs.php?pid=NP_001177411.1) | 30 | 0.394 | Tolerated | 2.82 | 39 |
| 39 | NP_001177411.1,1442,I,W | [NP_001177411.1](http://www.ncbi.nlm.nih.gov/protein/NP_001177411.1) | 1442 | I | W | -0.78 | Neutral | [139](http://provean.jcvi.org/view_supporting_seqs.php?pid=NP_001177411.1) | 30 | 0.185 | Tolerated | 2.82 | 39 |
| 40 | NP_001177411.1,1442,I,E | [NP_001177411.1](http://www.ncbi.nlm.nih.gov/protein/NP_001177411.1) | 1442 | I | E | 0.08 | Neutral | [139](http://provean.jcvi.org/view_supporting_seqs.php?pid=NP_001177411.1) | 30 | 0.273 | Tolerated | 2.82 | 39 |
| 41 | NP_001177411.1,1442,I,Q | [NP_001177411.1](http://www.ncbi.nlm.nih.gov/protein/NP_001177411.1) | 1442 | I | Q | 0.40 | Neutral | [139](http://provean.jcvi.org/view_supporting_seqs.php?pid=NP_001177411.1) | 30 | 0.295 | Tolerated | 2.82 | 39 |
| 42 | NP_001177411.1,1442,I,L | [NP_001177411.1](http://www.ncbi.nlm.nih.gov/protein/NP_001177411.1) | 1442 | I | L | 0.06 | Neutral | [139](http://provean.jcvi.org/view_supporting_seqs.php?pid=NP_001177411.1) | 30 | 0.649 | Tolerated | 2.82 | 39 |
| 43 | NP_001177411.1,1442,I,S | [NP_001177411.1](http://www.ncbi.nlm.nih.gov/protein/NP_001177411.1) | 1442 | I | S | 0.32 | Neutral | [139](http://provean.jcvi.org/view_supporting_seqs.php?pid=NP_001177411.1) | 30 | 0.400 | Tolerated | 2.82 | 39 |
| 44 | NP_001177411.1,1442,I,K | [NP_001177411.1](http://www.ncbi.nlm.nih.gov/protein/NP_001177411.1) | 1442 | I | K | 0.62 | Neutral | [139](http://provean.jcvi.org/view_supporting_seqs.php?pid=NP_001177411.1) | 30 | 0.296 | Tolerated | 2.82 | 39 |
| 45 | NP_001177411.1,1263,Y,L | [NP_001177411.1](http://www.ncbi.nlm.nih.gov/protein/NP_001177411.1) | 1263 | Y | L | -1.95 | Neutral | [139](http://provean.jcvi.org/view_supporting_seqs.php?pid=NP_001177411.1) | 30 | 0.726 | Tolerated | 2.82 | 44 |
| 46 | NP_001177411.1,1263,Y,F | [NP_001177411.1](http://www.ncbi.nlm.nih.gov/protein/NP_001177411.1) | 1263 | Y | F | -1.08 | Neutral | [139](http://provean.jcvi.org/view_supporting_seqs.php?pid=NP_001177411.1) | 30 | 0.437 | Tolerated | 2.82 | 44 |
| 47 | NP_001177411.1,1263,Y,S | [NP_001177411.1](http://www.ncbi.nlm.nih.gov/protein/NP_001177411.1) | 1263 | Y | S | -2.59 | Deleterious | [139](http://provean.jcvi.org/view_supporting_seqs.php?pid=NP_001177411.1) | 30 | 0.202 | Tolerated | 2.82 | 44 |
| 48 | NP_004707.2,731,P,L | [NP_004707.2](http://www.ncbi.nlm.nih.gov/protein/NP_004707.2) | 731 | P | L | -0.19 | Neutral | [182](http://provean.jcvi.org/view_supporting_seqs.php?pid=NP_004707.2) | 30 | 0.941 | Tolerated | 3.67 | 30 |
| 49 | NP_004707.2,731,P,Q | [NP_004707.2](http://www.ncbi.nlm.nih.gov/protein/NP_004707.2) | 731 | P | Q | 0.51 | Neutral | [182](http://provean.jcvi.org/view_supporting_seqs.php?pid=NP_004707.2) | 30 | 0.290 | Tolerated | 3.67 | 30 |
| 50 | NP_004707.2,731,P,L | [NP_004707.2](http://www.ncbi.nlm.nih.gov/protein/NP_004707.2) | 731 | P | L | -0.19 | Neutral | [182](http://provean.jcvi.org/view_supporting_seqs.php?pid=NP_004707.2) | 30 | 0.941 | Tolerated | 3.67 | 30 |
| 51 | NP_004707.2,731,P,A | [NP_004707.2](http://www.ncbi.nlm.nih.gov/protein/NP_004707.2) | 731 | P | A | 1.02 | Neutral | [182](http://provean.jcvi.org/view_supporting_seqs.php?pid=NP_004707.2) | 30 | 0.618 | Tolerated | 3.67 | 30 |
| 52 | NP_004707.2,731,P,V | [NP_004707.2](http://www.ncbi.nlm.nih.gov/protein/NP_004707.2) | 731 | P | V | -0.02 | Neutral | [182](http://provean.jcvi.org/view_supporting_seqs.php?pid=NP_004707.2) | 30 | 1.000 | Tolerated | 3.67 | 30 |
| 53 | NP_004707.2,598,E,P | [NP_004707.2](http://www.ncbi.nlm.nih.gov/protein/NP_004707.2) | 598 | E | P | -0.86 | Neutral | [182](http://provean.jcvi.org/view_supporting_seqs.php?pid=NP_004707.2) | 30 | 0.070 | Tolerated | 2.81 | 292 |
| 54 | NP_004707.2,652,I,Y | [NP_004707.2](http://www.ncbi.nlm.nih.gov/protein/NP_004707.2) | 652 | I | Y | -0.59 | Neutral | [182](http://provean.jcvi.org/view_supporting_seqs.php?pid=NP_004707.2) | 30 | 0.043 | Damaging | 3.00 | 109 |
| 55 | NP_004707.2,662,P,C | [NP_004707.2](http://www.ncbi.nlm.nih.gov/protein/NP_004707.2) | 662 | P | C | 1.36 | Neutral | [182](http://provean.jcvi.org/view_supporting_seqs.php?pid=NP_004707.2) | 30 | 0.002 | Damaging | 3.02 | 88 |
| 56 | NP_004707.2,659,T,S | [NP_004707.2](http://www.ncbi.nlm.nih.gov/protein/NP_004707.2) | 659 | T | S | 0.13 | Neutral | [182](http://provean.jcvi.org/view_supporting_seqs.php?pid=NP_004707.2) | 30 | 0.303 | Tolerated | 3.03 | 102 |
| 57 | NP_004707.2,659,T,G | [NP_004707.2](http://www.ncbi.nlm.nih.gov/protein/NP_004707.2) | 659 | T | G | 2.01 | Neutral | [182](http://provean.jcvi.org/view_supporting_seqs.php?pid=NP_004707.2) | 30 | 0.032 | Damaging | 3.03 | 102 |
| 58 | NP_004707.2,781,E,D | [NP_004707.2](http://www.ncbi.nlm.nih.gov/protein/NP_004707.2) | 781 | E | D | -0.62 | Neutral | [182](http://provean.jcvi.org/view_supporting_seqs.php?pid=NP_004707.2) | 30 | 0.204 | Tolerated | 4.32 | 24 |
| 59 | NP_004707.2,781,E,S | [NP_004707.2](http://www.ncbi.nlm.nih.gov/protein/NP_004707.2) | 781 | E | S | -0.10 | Neutral | [182](http://provean.jcvi.org/view_supporting_seqs.php?pid=NP_004707.2) | 30 | 0.213 | Tolerated | 4.32 | 24 |
| 60 | NP_777596.2,2,G,T | [NP_777596.2](http://www.ncbi.nlm.nih.gov/protein/NP_777596.2) | 2 | G | T | -0.52 | Neutral | [84](http://provean.jcvi.org/view_supporting_seqs.php?pid=NP_777596.2) | 30 | 0.000 | Damaging | 4.32 | 20 |
| 61 | NP_777596.2,2,G,V | [NP_777596.2](http://www.ncbi.nlm.nih.gov/protein/NP_777596.2) | 2 | G | V | -0.76 | Neutral | [84](http://provean.jcvi.org/view_supporting_seqs.php?pid=NP_777596.2) | 30 | 0.000 | Damaging | 4.32 | 20 |
| 62 | NP_777596.2,2,G,A | [NP_777596.2](http://www.ncbi.nlm.nih.gov/protein/NP_777596.2) | 2 | G | A | -0.42 | Neutral | [84](http://provean.jcvi.org/view_supporting_seqs.php?pid=NP_777596.2) | 30 | 0.000 | Damaging | 4.32 | 20 |
| 63 | NP_777596.2,3,T,A | [NP_777596.2](http://www.ncbi.nlm.nih.gov/protein/NP_777596.2) | 3 | T | A | -0.34 | Neutral | [84](http://provean.jcvi.org/view_supporting_seqs.php?pid=NP_777596.2) | 30 | 0.181 | Tolerated | 4.32 | 20 |
| 64 | NP_777596.2,3,T,H | [NP_777596.2](http://www.ncbi.nlm.nih.gov/protein/NP_777596.2) | 3 | T | H | -0.26 | Neutral | [84](http://provean.jcvi.org/view_supporting_seqs.php?pid=NP_777596.2) | 30 | 0.005 | Damaging | 4.32 | 20 |
| 65 | NP_777596.2,3,T,R | [NP_777596.2](http://www.ncbi.nlm.nih.gov/protein/NP_777596.2) | 3 | T | R | -0.32 | Neutral | [84](http://provean.jcvi.org/view_supporting_seqs.php?pid=NP_777596.2) | 30 | 0.011 | Damaging | 4.32 | 20 |
| 66 | NP_777596.2,555,Q,R | [NP_777596.2](http://www.ncbi.nlm.nih.gov/protein/NP_777596.2) | 555 | Q | R | -0.79 | Neutral | [84](http://provean.jcvi.org/view_supporting_seqs.php?pid=NP_777596.2) | 30 | 0.262 | Tolerated | 2.99 | 44 |
| 67 | NP_777596.2,555,Q,H | [NP_777596.2](http://www.ncbi.nlm.nih.gov/protein/NP_777596.2) | 555 | Q | H | -0.89 | Neutral | [84](http://provean.jcvi.org/view_supporting_seqs.php?pid=NP_777596.2) | 30 | 0.062 | Tolerated | 2.99 | 44 |
| 68 | NP_777596.2,555,Q,P | [NP_777596.2](http://www.ncbi.nlm.nih.gov/protein/NP_777596.2) | 555 | Q | P | -0.07 | Neutral | [84](http://provean.jcvi.org/view_supporting_seqs.php?pid=NP_777596.2) | 30 | 0.344 | Tolerated | 2.99 | 44 |
| 69 | NP_777596.2,201,M,V | [NP_777596.2](http://www.ncbi.nlm.nih.gov/protein/NP_777596.2) | 201 | M | V | -0.70 | Neutral | [84](http://provean.jcvi.org/view_supporting_seqs.php?pid=NP_777596.2) | 30 | 0.579 | Tolerated | 2.86 | 46 |

Mutations with PROVEAN Score less than -2.5 are predicted to be deleterious. mutations with SIFT score less than 0.05 are predicted to be deleterious, while those greater than 0.05 are neutral.
