# Supplementary material for: Molecular evolution of PCSK family: Analysis of natural selection rate and gene loss
Source: PLoS One. 2021 Oct 28;16(10):e0259085. doi: 10.1371/journal.pone.0259085 (PMC8553125; doi:10.1371/journal.pone.0259085)
Supplement: S6 Table — np: number of parameters for each model, NS: not significant (p-value > 0.05). (DOCX) [file pone.0259085.s043.docx]

**S6 Table. Parameter estimates for PCSK1 Clade model C and the result of LRT tests**

| **Comparison** | **Model** | **np** | **lnL** | **Model parameters** | **2ΔlnL** | ***P*.value** |
| --- | --- | --- | --- | --- | --- | --- |
| *Chiroptera* order (bats) | clade | 89 | -18346.839125 | P_0_=0.73266, P_1_=0.06011, P_2_=0.207  BG: ω_0_=0.00989, ω_1_=1.00000, ω_2_=0.22392  FG: ω_0_=0.00989, ω_2_=1.00000, ω_2_=0.47080 |  |  |
|  | M2A_rel | 88 | -18356.164585 | P_0_=0.72705, P_1_=0.06248, p_2_=0.21048  ω_0_=0.00949, ω_1_=1.00000, ω_2_=0.23808 | 18.65092 | <0.0005 |
| *Rodentia* order (rodents) | clade | 89 | -18355.683415 | P_0_=0.72640, P_1_=0.06242, P_2_=0.21117  BG: ω_0_=0.00943, ω_1_=1.00000, ω_2_=0.24717  FG: ω_0_=0.00943, ω_2_=1.00000, ω_2_=0.21726 |  |  |
|  | M2A_rel | 88 | -18356.164585 | P_0_=0.72705, P_1_=0.06248, p_2_=0.21048  ω_0_=0.00949, ω_1_=1.00000, ω_2_=0.23808 | 0.96234 | NS |
| *Muridae* family | clade | 89 | -18352.516343 | P_0_=0.72341, P_1_=0.06407, P_2_=0.21252  BG: ω_0_=0.00919, ω_1_=1.00000, ω_2_=0.22201  FG: ω_0_=0.00919, ω_2_=1.00000, ω_2_=0.39073 |  |  |
|  | M2A_rel | 88 | -18356.164585 | P_0_=0.72705, P_1_=0.06248, p_2_=0.21048  ω_0_=0.00949, ω_1_=1.00000, ω_2_=0.23808 | 7.296484 | <0.005 |
| *Artiodactyla* order | clade | 89 | -18354.682155 | P_0_=0.73313, P_1_=0.06159, P_2_=0.20528  BG: ω_0_=0.01007, ω_1_=1.00000, ω_2_=0.25192  FG: ω_0_=0.01007, ω_2_=1.00000, ω_2_=0.16573 |  |  |
|  | M2A_rel | 88 | -18356.164585 | P_0_=0.72705, P_1_=0.06248, p_2_=0.21048  ω_0_=0.00949, ω_1_=1.00000, ω_2_=0.23808 | 2.96486 | NS |
| *Balaenopteridae*, *Delphinidae*, *Monodontidae* and *Phocoenidae* families from *Artiodoctyla* order | clade | 89 | -18355.535053 | P_0_=0.72815, P_1_=0.06227, P_2_=0.20957  BG: ω_0_=0.00958, ω_1_=1.00000, ω_2_=0.24149  FG: ω_0_=0.00958, ω_2_=1.00000, ω_2_=0.13561 |  |  |
|  | M2A_rel | 88 | -18356.164585 | P_0_=0.72705, P_1_=0.06248, p_2_=0.21048  ω_0_=0.00949, ω_1_=1.00000, ω_2_=0.23808 | 1.259064 | NS |
| *Carnivora* order | clade | 89 | -18356.145799 | P_0_=0.72765, P_1_=0.06230, P_2_=0.21006  BG: ω_0_=0.00955, ω_1_=1.00000, ω_2_=0.23955  FG: ω_0_=0.00955, ω_2_=1.00000, ω_2_=0.22753 |  |  |
|  | M2A_rel | 88 | -18356.164585 | P_0_=0.72705, P_1_=0.06248, p_2_=0.21048  ω_0_=0.00949, ω_1_=1.00000, ω_2_=0.23808 | 0.037572 | NS |

np: number of parameters for each model, NS: not significant ( p-value > 0.05)
