# Supplementary material for: Molecular evolution of PCSK family: Analysis of natural selection rate and gene loss
Source: PLoS One. 2021 Oct 28;16(10):e0259085. doi: 10.1371/journal.pone.0259085 (PMC8553125; doi:10.1371/journal.pone.0259085)
Supplement: S7 Table — np: number of parameters for each model, NS: not significant (p-value > 0.05). (DOCX) [file pone.0259085.s044.docx]

| **Comparison** | **Model** | **np** | **lnL** | **Model parameters** | **2lnL** | ***P*.value** |
| --- | --- | --- | --- | --- | --- | --- |
| *Chiroptera* order (bats) | clade | 89 | -19362.302145 | P_0_=0.77804, P_1_=0.01996, P_2_=0.20199  BG: ω_0_=0.00727, ω_1_=1.00000, ω_2_=0.17111  FG: ω_0_=0.00727, ω_1_=1.00000, ω_2_=0.15517 |  |  |
|  | M2A_rel | 88 | -19362.445586 | P_0_=0.77822, P_1_=0.01993, p_2_=0.20186  ω_0_=0.00728, ω_1_=1.00000, ω_2_=0.16951 | 0.286882 | NS |
| *Rodentia* order (rodents) | clade | 89 | -19361.328102 | P_0_=0.77920, P_1_=0.02005, P_2_=0.20075  BG: ω_0_=0.00736, ω_1_=1.00000, ω_2_=0.17887  FG: ω_0_=0.00736, ω_2_=1.00000, ω_2_=0.14777 |  |  |
|  | M2A_rel | 88 | -19362.445586 | P_0_=0.77822, P_1_=0.01993, p_2_=0.20186  ω_0_=0.00728, ω_1_=1.00000, ω_2_=0.16951 | 2.234968 | NS |
| *Muridae* family | clade | 89 | -19362.441392 | P_0_=0.77850, P_1_=0.01990, P_2_=0.20160  BG: ω_0_=0.00729, ω_1_=1.00000, ω_2_=0.16996  FG: ω_0_=0.00729, ω_2_=1.00000, ω_2_=0.16629 |  |  |
|  | M2A_rel | 88 | -19362.445586 | P_0_=0.77822, P_1_=0.01993, p_2_=0.20186  ω_0_=0.00728, ω_1_=1.00000, ω_2_=0.16951 | 0.008388 | NS |
| *Artiodactyla* order | clade | 89 | -19361.827784 | P_0_=0.78031, P_1_=0.01945, P_2_=0.20024  BG: ω_0_=0.00740, ω_1_=1.00000, ω_2_=0.16803  FG: ω_0_=0.00740, ω_2_=1.00000, ω_2_=0.20794 |  |  |
|  | M2A_rel | 88 | -19362.445586 | P_0_=0.77822, P_1_=0.01993, p_2_=0.20186  ω_0_=0.00728, ω_1_=1.00000, ω_2_=0.16951 | 1.235604 | NS |
| *Balaenopteridae*, *Delphinidae*, *Monodontidae* and *Phocoenidae* families from *Artiodoctyla* order | clade | 89 | -19361.019633 | P_0_=0.77912, P_1_=0.01968, P_2_=0.20120  BG: ω_0_=0.00732, ω_1_=1.00000, ω_2_=0.16783  FG: ω_0_=0.00732, ω_2_=1.00000, ω_2_=0.29764 |  |  |
|  | M2A_rel | 88 | -19362.445586 | P_0_=0.77822, P_1_=0.01993, p_2_=0.20186  ω_0_=0.00728, ω_1_=1.00000, ω_2_=0.16951 | 2.8519006 | NS |
| *Carnivora* order | clade | 89 | -19360.244607 | P_0_=0.77600, P_1_=0.02033, P_2_=0.20367  BG: ω_0_=0.00714, ω_1_=1.00000, ω_2_=0.17210  FG: ω_0_=0.00714, ω_2_=1.00000, ω_2_=0.09495 |  |  |
|  | M2A_rel | 88 | -19362.445586 | P_0_=0.77822, P_1_=0.01993, p_2_=0.20186  ω_0_=0.00728, ω_1_=1.00000, ω_2_=0.16951 | 4.401958 | <0.05 |

**S7 Table. Parameter estimates for PCSK3 Clade model C and the result of LRT tests**

np: number of parameters for each model, NS: not significant ( p-value > 0.05)
