# Supplementary material for: Molecular evolution of PCSK family: Analysis of natural selection rate and gene loss
Source: PLoS One. 2021 Oct 28;16(10):e0259085. doi: 10.1371/journal.pone.0259085 (PMC8553125; doi:10.1371/journal.pone.0259085)
Supplement: S8 Table — np: number of parameters for each model, NS: not significant (p-value > 0.05). (DOCX) [file pone.0259085.s045.docx]

**S8 Table. Parameter estimates for PCSK5 Clade model C and the result of LRT tests**

| **Comparison** | **Model** | **np** | **lnL** | **Model parameters** | **2lnL** | ***P*.value** |
| --- | --- | --- | --- | --- | --- | --- |
| *Chiroptera* order (bats) | clade | 71 | -46352.522027 | P_0_=0.60391, P_1_=0.11579, P_2_=0.28030  BG: ω_0_=0.01354, ω_1_=1.00000, ω_2_=0.30629  FG: ω_0_=0.01354, ω_1_=1.00000, ω_2_=0.27730 |  |  |
|  | M2A_rel | 70 | -46352.819636 | P_0_=0.60412, P_1_=0.11550, P_2_=0.28039  ω_0_=0.01356, ω_1_=1.00000, ω_2_=0.30452 | 0.595218 | NS |
| *Rodentia* order (rodents) | clade | 71 | -46179.305186 | P_0_=0.60091, P_1_=0.12048, P_2_=0.27860  BG: ω_0_=0.01454, ω_1_=1.00000, ω_2_=0.32292  FG: ω_0_=0.01454, ω_1_=1.00000, ω_2_=0.33351 |  |  |
|  | M2A_rel | 70 | -46352.819636 | P_0_=0.60412, P_1_=0.11550, P_2_=0.28039  ω_0_=0.01356, ω_1_=1.00000, ω_2_=0.30452 | 347.0289 | <0.0005 |
| *Muridae* family | clade | 71 | -46351.475329 | P_0_=0.60347, P_1_=0.11570, P_2_=0.28083  BG: ω_0_=0.01348, ω_1_=1.00000, ω_2_=0.30813  FG: ω_0_=0.01348, ω_1_=1.00000, ω_2_=0.24206 |  |  |
|  | M2A_rel | 70 | -46352.819636 | P_0_=0.60412, P_1_=0.11550, P_2_=0.28039  ω_0_=0.01356, ω_1_=1.00000, ω_2_=0.30452 | 680.861034 | NS |
| *Artiodactyla* order | clade | 71 | -46350.886507 | P_0_=0.60396, P_1_=0.11602, P_2_=0.28003  BG: ω_0_=0.01355, ω_1_=1.00000, ω_2_=0.31105  FG: ω_0_=0.01355, ω_1_=1.00000, ω_2_=0.24926 |  |  |
|  | M2A_rel | 70 | -46352.819636 | P_0_=0.60412, P_1_=0.11550, P_2_=0.28039  ω_0_=0.01356, ω_1_=1.00000, ω_2_=0.30452 | 3.866258 | NS |
| *Balaenopteridae*, *Delphinidae*, *Monodontidae* and *Phocoenidae* families from *Artiodoctyla* order | clade | 71 | -46352.775870 | P_0_=0.60407, P_1_=0.11543, P_2_=0.28050  BG: ω_0_=0.01356, ω_1_=1.00000, ω_2_=0.30387  FG: ω_0_=0.01356, ω_1_=1.00000, ω_2_=0.32017 |  |  |
|  | M2A_rel | 70 | -46352.819636 | P_0_=0.60412, P_1_=0.11550, P_2_=0.28039  ω_0_=0.01356, ω_1_=1.00000, ω_2_=0.30452 | 0.087532 | NS |
| *Carnivora* order | clade | 71 | -46352.668573 | P_0_=0.60392, P_1_=0.11564, P_2_=0.28044  BG: ω_0_=0.01354, ω_1_=1.00000, ω_2_=0.30265  FG: ω_0_=0.01354, ω_1_=1.00000, ω_2_=0.30265 |  |  |
|  | M2A_rel | 70 | -46352.819636 | P_0_=0.60412, P_1_=0.11550, P_2_=0.28039  ω_0_=0.01356, ω_1_=1.00000, ω_2_=0.30452 | 0.302126 | NS |

np: number of parameters for each model, NS: not significant ( p-value > 0.05)
