# Supplementary material for: Molecular evolution of PCSK family: Analysis of natural selection rate and gene loss
Source: PLoS One. 2021 Oct 28;16(10):e0259085. doi: 10.1371/journal.pone.0259085 (PMC8553125; doi:10.1371/journal.pone.0259085)
Supplement: S9 Table — np: number of parameters for each model, NS: not significant (p-value > 0.05). (DOCX) [file pone.0259085.s046.docx]

**S9 Table. Parameter estimates for PCSK7 Clade model C and the result of LRT tests**

| **Comparison** | **Model** | **np** | **lnL** | **Model parameters** | **2lnL** | ***P*.value** |
| --- | --- | --- | --- | --- | --- | --- |
| *Chiroptera* order (bats) | clade | 89 | -20262.624527 | P_0_=0.66267, P_1_=0.05373, P_2_=0.28360  BG: ω_0_=0.00955, ω_1_=1.00000, ω_2_=0.25616  FG: ω_0_=0.00955, ω_2_=1.00000, ω_2_=0.34580 |  |  |
|  | M2A_rel | 88 | -20264.328358 | P_0_=0.66331, P_1_=0.05486, p_2_=0.28183  ω_0_=0.00964, ω_1_=1.00000, ω_2_=0.26266 | 3.407662 | NS |
| *Rodentia* order (rodents) | clade | 89 | -20262.743966 | P_0_=0.66277, P_1_=0.05497, P_2_=0.28226  BG: ω_0_=0.00956, ω_1_=1.00000, ω_2_=0.24850  FG: ω_0_=0.00956, ω_2_=1.00000, ω_2_=0.29854 |  |  |
|  | M2A_rel | 88 | -20264.328358 | P_0_=0.66331, P_1_=0.05486, p_2_=0.28183  ω_0_=0.00964, ω_1_=1.00000, ω_2_=0.26266 | 3.168784 | NS |
| *Muridae* family | clade | 89 | -20256.162247 | P_0_=0.65950, P_1_=0.05691, P_2_=0.28359  BG: ω_0_=0.00919, ω_1_=1.00000, ω_2_=0.24502  FG: ω_0_=0.00919, ω_2_=1.00000, ω_2_=0.48386 |  |  |
|  | M2A_rel | 88 | -20264.328358 | P_0_=0.66331, P_1_=0.05486, p_2_=0.28183  ω_0_=0.00964, ω_1_=1.00000, ω_2_=0.26266 | 442.658732 | <0.0005 |
| *Artiodactyla* order | clade | 89 | -20264.328282 | P_0_=0.66332, P_1_=0.05486, P_2_=0.28182  BG: ω_0_=0.00964, ω_1_=1.00000, ω_2_=0.26261  FG: ω_0_=0.00964, ω_2_=1.00000, ω_2_=0.26308 |  |  |
|  | M2A_rel | 88 | -20264.328358 | P_0_=0.66331, P_1_=0.05486, p_2_=0.28183  ω_0_=0.00964, ω_1_=1.00000, ω_2_=0.26266 | 0.000152 | NS |
| *Balaenopteridae*, *Delphinidae*, *Monodontidae* and *Phocoenidae* families from *Artiodoctyla* order | clade | 89 | -20262.535679 | P_0_=0.66220, P_1_=0.05603, P_2_=0.28177  BG: ω_0_=0.00955, ω_1_=1.00000, ω_2_=0.25624  FG: ω_0_=0.00955, ω_2_=1.00000, ω_2_=0.41149 |  |  |
|  | M2A_rel | 88 | -20264.328358 | P_0_=0.66331, P_1_=0.05486, p_2_=0.28183  ω_0_=0.00964, ω_1_=1.00000, ω_2_=0.26266 | 3.58 | NS |
| *Carnivora* order | clade | 89 | -20259.389833 | P_0_=0.66140, P_1_=0.05580, P_2_=0.28280  BG: ω_0_=0.00942, ω_1_=1.00000, ω_2_=0.27149  FG: ω_0_=0.00942, ω_2_=1.00000, ω_2_=0.14542 |  |  |
|  | M2A_rel | 88 | -20264.328358 | P_0_=0.66331, P_1_=0.05486, p_2_=0.28183  ω_0_=0.00964, ω_1_=1.00000, ω_2_=0.26266 | 9.87705 | <0.0025 |

np: number of parameters for each model, NS: not significant ( p-value > 0.05)
