# Supplementary material for: Molecular evolution of PCSK family: Analysis of natural selection rate and gene loss
Source: PLoS One. 2021 Oct 28;16(10):e0259085. doi: 10.1371/journal.pone.0259085 (PMC8553125; doi:10.1371/journal.pone.0259085)
Supplement: S10 Table — np: number of parameters for each model, NS: not significant (p-value > 0.05). (DOCX) [file pone.0259085.s047.docx]

**S10 Table. Parameter estimates for** **MBTPS1 Clade model C and the result of LRT tests**

| **Comparison** | **Model** | **np** | **lnL** | **Model parameters** | **2lnL** | ***P*.value** |
| --- | --- | --- | --- | --- | --- | --- |
| *Chiroptera* order (bats) | clade | 91 | -20753.533930 | P_0_=0.84261, P_1_=0.00209, P_2_=0.15530  BG: ω_0_=0.00641, ω_1_=1.00000, ω_2_=0.16841  FG: ω_0_=0.00641, ω_1_=1.00000, ω_2_=0.12293 |  |  |
|  | M2A_rel | 90 | -20754.044053 | P_0_=0.84328, P_1_=0.00188, P_2_=0.15484  ω_0_=0.00645, ω_1_=1.00000, ω_2_=0.16713 | 1.020246 | NS |
| *Rodentia* order (rodents) | clade | 91 | -20744.592954 | P_0_=0.84739, P_1_=0.00194, P_2_=0.15067  BG: ω_0_=0.00673, ω_1_=1.00000, ω_2_=0.14315  FG: ω_0_=0.00673, ω_1_=1.00000, ω_2_=0.25288 |  |  |
|  | M2A_rel | 90 | -20754.044053 | P_0_=0.84328, P_1_=0.00188, P_2_=0.15484  ω_0_=0.00645, ω_1_=1.00000, ω_2_=0.16713 | 18.902198 | <0.0005 |
| *Muridae* family | clade | 91 | -20752.284864 | P_0_=0.84535, P_1_=0.00176, P_2_=0.15289  BG: ω_0_=0.00658, ω_1_=1.00000, ω_2_=0.16426  FG: ω_0_=0.00658, ω_1_=1.00000, ω_2_=0.26199 |  |  |
|  | M2A_rel | 90 | -20754.044053 | P_0_=0.84328, P_1_=0.00188, P_2_=0.15484  ω_0_=0.00645, ω_1_=1.00000, ω_2_=0.16713 | 3.518378 | NS |
| *Artiodactyla* order | clade | 91 | -20753.847060 | P_0_=0.84349, P_1_=0.00192, P_2_=0.15459  BG: ω_0_=0.00646, ω_1_=1.00000, ω_2_=0.16482  FG: ω_0_=0.00646, ω_1_=1.00000, ω_2_=0.18485 |  |  |
|  | M2A_rel | 90 | -20754.044053 | P_0_=0.84328, P_1_=0.00188, P_2_=0.15484  ω_0_=0.00645, ω_1_=1.00000, ω_2_=0.16713 | 0.393986 | NS |
| *Balaenopteridae*, *Delphinidae*, *Monodontidae* and *Phocoenidae* families from *Artiodoctyla* order | clade | 91 | -20752.157929 | P_0_=0.84228, P_1_=0.00208, P_2_=0.15564  BG: ω_0_=0.00637, ω_1_=1.00000, ω_2_=0.16206  FG: ω_0_=0.00637, ω_1_=1.00000, ω_2_=0.29178 |  |  |
|  | M2A_rel | 90 | -20754.044053 | P_0_=0.84328, P_1_=0.00188, P_2_=0.15484  ω_0_=0.00645, ω_1_=1.00000, ω_2_=0.16713 | 3.772248 | NS |
| *Carnivora* order | clade | 91 | -20748.444017 | P_0_=0.84410, P_1_=0.00198, P_2_=0.15392  BG: ω_0_=0.00650, ω_1_=1.00000, ω_2_=0.17706  FG: ω_0_=0.00650, ω_1_=1.00000, ω_2_=0.06792 |  |  |
|  | M2A_rel | 90 | -20754.044053 | P_0_=0.84328, P_1_=0.00188, P_2_=0.15484  ω_0_=0.00645, ω_1_=1.00000, ω_2_=0.16713 | 11.200072 | <0.0005 |

np: number of parameters for each model, NS: not significant ( p-value > 0.05)
