# Supplementary material for: Molecular evolution of PCSK family: Analysis of natural selection rate and gene loss
Source: PLoS One. 2021 Oct 28;16(10):e0259085. doi: 10.1371/journal.pone.0259085 (PMC8553125; doi:10.1371/journal.pone.0259085)
Supplement: S11 Table — np: number of parameters for each model, NS: not significant (p-value > 0.05). (DOCX) [file pone.0259085.s048.docx]

**S11 Table. Parameter estimates for PCSK9 Clade model C and the result of LRT tests**

| **Comparison** | **Model** | **np** | **lnL** | **Model parameters** | **2lnL** | ***P*.value** |
| --- | --- | --- | --- | --- | --- | --- |
| *Homonidae* family | clade | 111 | -25525.214437 | P0=0.52893, P1=0.08773, p2=0.38335  BG: w0=0.03166, w1=1.00000, w2=0.27862  FG: w0=0.03166, w1=1.00000, w2=0.39698 |  |  |
|  | M2A_rel | 110 | -25525.738276 | P_0_=0.52853, P_1_=0.08811, p_2_=0.38336  w_0_=0.03161, w_1_=1.00000, w_2_=0.27935 | 1.047678 |  |
| *Cercopithecidae* family | clade | 111 | -25523.740735 | P0=0.52860, P1=0.08834, p2=0.38306  BG: w0=0.03167 , w1=1.00000, w2=0.28212  FG: w0=0.03167 , w1=1.00000, w2=0.14656 |  |  |
|  | M2A_rel | 110 | -25525.738276 | P_0_=0.52853, P_1_=0.08811, p_2_=0.38336  w_0_=0.03161, w_1_=1.00000, w_2_=0.27935 | 3.995082 | <0.05 |
| *Rodentia* order (rodents) | clade | 111 | -25525.735738 | P0=0.52856, P1=0.08809, p2=0.38334  BG: w0=0.03162, w1=1.00000, w2=0.27874  FG: w0=0.03162, w1=1.00000, w2=0.28031 |  |  |
|  | M2A_rel | 110 | -25525.738276 | P_0_=0.52853, P_1_=0.08811, p_2_=0.38336  w_0_=0.03161, w_1_=1.00000, w_2_=0.27935 | 0.005076 | NS |
| *Artiodactyla* order | clade | 111 | -25525.458572 | P0=0.52731, P1=0.08869, p2=0.38400  BG: w0=0.03147, w1=1.00000, w2=0.28084  FG: w0=0.03147, w1=1.00000, w2=0.24981 |  |  |
|  | M2A_rel | 110 | -25525.738276 | P_0_=0.52853, P_1_=0.08811, p_2_=0.38336  w_0_=0.03161, w_1_=1.00000, w_2_=0.27935 | 0.559408 | NS |
| *Balaenopteridae*, *Delphinidae*, *Monodontidae* and *Phocoenidae* families from *Artiodoctyla* order | clade | 111 | -25525.549287 | P0=0.52721, P1=0.08879, p2=0.38400  BG: w0=0.03145, w1=1.00000, w2=0.27994  FG: w0=0.03145, w1=1.00000, w2=0.24226 |  |  |
|  | M2A_rel | 110 | -25525.738276 | P_0_=0.52853, P_1_=0.08811, p_2_=0.38336  w_0_=0.03161, w_1_=1.00000, w_2_=0.27935 | 0.377978 | NS |

np: number of parameters for each model, NS: not significant ( p-value > 0.05)
