# Supplementary material for: Molecular evolution of PCSK family: Analysis of natural selection rate and gene loss
Source: PLoS One. 2021 Oct 28;16(10):e0259085. doi: 10.1371/journal.pone.0259085 (PMC8553125; doi:10.1371/journal.pone.0259085)
Supplement: S12 Table — np: number of parameters for each model, NS: not significant; Positive selection sites are numbered according to the PCSK1 reference sequence in H. sapiens (NP_000430.3), *probability >0.95, ** probability >0.99. (DOCX) [file pone.0259085.s049.docx]

**S12 Table. Parameter estimates for PCSK1 branch-site model**

| **Foreground**  **branches** | **Model** | **np** | **lnL** | **Model parameters** | **2ΔlnL** | ***P*.value** | **Corresponding sites of**  P**ositive selection in**  **H**.**sapiens** **pcsk1 (Probability**  **(BEB))** |
| --- | --- | --- | --- | --- | --- | --- | --- |
| *Chiroptera* order (bats) | null | 87 | -18455.795797 | P_0_=0.84780, P_1_=0.11033, P_2a_=0.03705, P_2b_=0.00482  BG: ω_0_=0.03751, ω_1_=1.00000, ω_2a_=0.03751, ω_2b_=1.00000  FG: ω_0_=0.03751, ω_1_=1.00000, ω_2a_=1.00000, ω_2b_=1.00000 | 0.034526 |  |  |
|  | Alternative | 88 | -18455.778534 | P_0_=0.85038, P_1_=0.11075, P_2a_=0.03439, P_2b_=0.00448  BG: ω_0_=0.03758, ω_1_=1.00000, ω_2a_=0.03758, ω_2b_=1.00000  FG: ω_0_=0.03758, ω_1_=1.00000, ω_2a_=1.08232, ω_2b_=1.08232 |  | NS | 502 K 0.975*  532 S 0.933* |
| *Rodentia* order (rodents) | null | 87 | -18468.175291 | P_0_=0.87904, P_1_=0.11543, P_2a_=0.00489, P_2b_=0.00064  BG: ω_0_=0.03964, ω_1_=1.00000, ω_2a_=0.03964, ω_2b_=1.00000  FG: ω_0_=0.03964, ω_1_=1.00000, ω_2a_=1.00000, ω_2b_=1.00000 | -1.390832 |  |  |
|  | Alternative | 88 | -18468.870707 | P_0_=0.88282, P_1_=0.11696, P_2a_=0.00019, P_2b_=0.00003  BG: ω_0_=0.04038, ω_1_=1.00000, ω_2a_=0.04038, ω_2b_=1.00000  FG: ω_0_=0.04038, ω_1_=1.00000, ω_2a_=5.31082, ω_2b_= 5.31082 |  | NS |  |
| *Muridae* family | null | 87 | -18464.956039 | P_0_= 0.85081, P_1_=0.11242, P_2a_=0.03248, P_2b_=0.00429  BG: ω_0_=0.03851, ω_1_=1.00000, ω_2a_=0.03851, ω_2b_=1.00000  FG: ω_0_=0.03851, ω_1_=1.00000, ω_2a_=1.00000, ω_2b_=1.00000 | -0.000196 |  |  |
|  | Alternative | 88 | -18464.956137 | P_0_=0.85084, P_1_=0.11242, P_2a_=0.03245, P_2b_=0.00429  BG: ω_0_=0.03852, ω_1_=1.00000, ω_2a_=0.03852, ω_2b_=1.00000  FG: ω_0_=0.03852, ω_1_=1.00000, ω_2a_=1.00000, ω_2b_=1.00000 |  | NS |  |
| *Artiodactyla* order | null | 87 | -18467.630510 | P_0_=0.87226, P_1_=0.11591, P_2a_=0.01044, P_2b_=0.00139  BG: ω_0_=0.03946, ω_1_=1.00000, ω_2a_=0.03946, ω_2b_=1.00000  FG: ω_0_=0.03946, ω_1_=1.00000, ω_2a_=1.00000, ω_2b_=1.00000 | -0.000688 |  |  |
|  | Alternative | 88 | -18467.630854 | P_0_=0.87216, P_1_=0.11587, P_2a_=0.01057, P_2b_=0.00140  BG: ω_0_=0.03945, ω_1_=1.00000, ω_2a_=0.03945, ω_2b_=1.00000  FG: ω_0_=0.03945, ω_1_=1.00000, ω_2a_=1.00000, ω_2b_=1.00000 |  | NS |  |
| *Balaenopteridae*, *Delphinidae*, *Monodontidae* and *Phocoenidae* families from *Artiodoctyla* order | null | 87 | -18468.855728 | P_0_=0.88065, P_1_=0.11661, P_2a_=0.00241, P_2b_=0.00032  BG: ω_0_=0.04033, ω_1_=1.00000, ω_2a_=0.04033, ω_2b_=1.00000  FG: ω_0_=0.04033, ω_1_=1.00000, ω_2a_=1.00000, ω_2b_=1.00000 | 0.004844 |  |  |
|  | Alternative | 88 | -18468.853306 | P_0_=0.88102, P_1_=0.11676, P_2a_=0.00196, P_2b_=0.00026  BG: ω_0_=0.04034, ω_1_=1.00000, ω_2a_=0.04034, ω_2b_=1.00000  FG: ω_0_=0.04034, ω_1_=1.00000, ω_2a_=1.00000, ω_2b_=1.00000 |  | NS |  |
| *Carnivora* order | null | 87 | -18463.810809 | P_0_=0.86199, P_1_=0.11307, P_2a_=0.02205, P_2b_=0.00289  BG: ω_0_=0.03923, ω_1_=1.00000, ω_2a_=0.03923, ω_2b_=1.00000  FG: ω_0_=0.03923, ω_1_=1.00000, ω_2a_=1.00000, ω_2b_=1.00000 | 5.396356 |  |  |
|  | Alternative | 88 | -18461.112631 | P_0_=0.87933, P_1_=0.11421, P_2a_=0.00571, P_2b_=0.00074  BG: ω_0_=0.03991, ω_1_=1.00000, ω_2a_=0.03991, ω_2b_=1.00000  FG: ω_0_=0.03991, ω_1_=1.00000, ω_2a_=8.63637, ω_2b_=8.63637 |  | <0.02 | 467 C 0.951*  471 D 0.998* |

np: number of parameters for each model, NS: not significant; Positive selection sites are numbered according to the PCSK1 reference sequence in H. sapiens (NP_000430.3), *probability >0.95, ** probability >0.99.
