# Supplementary material for: Molecular evolution of PCSK family: Analysis of natural selection rate and gene loss
Source: PLoS One. 2021 Oct 28;16(10):e0259085. doi: 10.1371/journal.pone.0259085 (PMC8553125; doi:10.1371/journal.pone.0259085)
Supplement: S13 Table — np: number of parameters for each model, NS: not significant; Positive selection sites are numbered according to the PCSK3 reference sequence in H. sapiens (NM_001289823.1), *probability >0.95, ** probability >0.99. (DOCX) [file pone.0259085.s050.docx]

**S13 Table. Parameter estimates for PCSK3 branch-site model**

| **Foreground**  **branches** |  | **Model** | **np** | **lnL** | **Model parameters** | **2lnL** | ***P*.value** | **Corresponding sites of**  P**ositive selection in**  **H**.**sapiens** **pcsk3 (Probability**  **(BEB))** |
| --- | --- | --- | --- | --- | --- | --- | --- | --- |
| *Chiroptera* order (bats) |  | null | 87 | -19546.602133 | P_0_=0.93320, P_1_=0.05362, P_2a_=0.01246, P_2b_=0.00072  BG: ω_0_=0.02944, ω_1_=1.00000, ω_2a_=0.02944, ω_2b_=1.00000  FG: ω_0_=0.02944, ω_1_=1.00000, ω_2a_=1.00000, ω_2b_=1.00000 |  |  |  |
|  |  | Alternative | 88 | -19546.602133 | P_0_=0.93320, P_1_=0.05362, P_2a_=0.01246, P_2b_=0.00072  BG: ω_0_=0.02944, ω_1_=1.00000, ω_2a_=0.02944, ω_2b_=1.00000  FG: ω_0_=0.02944, ω_1_=1.00000, ω_2a_=1.00000, ω_2b_=1.00000 | 0 | NS | 674 T 0.989* |
| *Rodentia* order (rodents) |  | null | 87 | -19549.926299 | P_0_=0.94025, P_1_=0.05198, P_2a_=0.00736, P_2b_=0.00041  BG: ω_0_=0.02984, ω_1_=1.00000, ω_2a_=0.02984, ω_2b_=1.00000  FG: ω_0_=0.02984, ω_1_=1.00000, ω_2a_=1.00000, ω_2b_=1.00000 |  |  |  |
|  |  | Alternative | 88 | -19552.495230 | P_0_=0.94613, P_1_=0.05387, P_2a_=0.00000, P_2b_= 0.00000  BG: ω_0_=0.03056, ω_1_=1.00000, ω_2a_=0.03056, ω_2b_=1.00000  FG: ω_0_=0.03056, ω_1_=1.00000, ω_2a_=35.27094, ω_2b_=35.27094 | 5.137862 | <0.02 |  |
| *Muridae* family |  | null | 87 | -19552.495226 | P_0_=0.94613, P_1_=0.05387, P_2a_=0.00000, P_2b_=0.00000  BG: ω_0_=0.03056, ω_1_=1.00000, ω_2a_=0.03056, ω_2b_=1.00000  FG: ω_0_=0.03056, ω_1_=1.00000, ω_2a_=1.00000, ω_2b_=1.00000 |  |  |  |
|  |  | Alternative | 88 | -19552.495226 | P_0_=0.94613, P_1_=0.05387, P_2a_=0.00000, P_2b_=0.00000  BG: ω_0_=0.03056, ω_1_=1.00000, ω_2a_=0.03056, ω_2b_=1.00000  FG: ω_0_=0.03056, ω_1_=1.00000, ω_2a_=1.00000, ω_2b_=1.00000 | 0 | NS |  |
| *Artiodactyla* order |  | null | 87 | -19548.523094 | P_0_=0.93815, P_1_=0.05090, P_2a_=0.01039, P_2b_=0.00056  BG: ω_0_=0.03024, ω_1_=1.00000, ω_2a_=0.03024, ω_2b_=1.00000  FG: ω_0_=0.03024, ω_1_=1.00000, ω_2a_=1.00000, ω_2b_=1.00000 |  |  |  |
|  |  | Alternative | 88 | -19548.410658 | P_0_=0.94058, P_1_=0.05100, P_2a_=0.00799, P_2b_=0.00043  BG: ω_0_=0.03031, ω_1_=1.00000, ω_2a_=0.03031, ω_2b_=1.00000  FG: ω_0_=0.03031, ω_1_=1.00000, ω_2a_=1.37457, ω_2b_=1.37457 | 0.224872 | NS | 37 R 0.989* |
| *Balaenopteridae*, *Delphinidae*, *Monodontidae* and *Phocoenidae* families from *Artiodoctyla* order |  | null | 87 | -19551.916774 | P_0_=0.93074, P_1_=0.05121, P_2a_=0.01710, P_2b_=0.00094  BG: ω_0_=0.03057, ω_1_=1.00000, ω_2a_=0.03057, ω_2b_=1.00000  FG: ω_0_=0.03057, ω_1_=1.00000, ω_2a_=1.00000, ω_2b_=1.00000 |  |  |  |
|  |  | Alternative | 88 | -19551.916774 | P_0_=0.93074, P_1_=0.05121, P_2a_=0.01710, P_2b_=0.00094  BG: ω_0_=0.03057, ω_1_=1.00000, ω_2a_=0.03057, ω_2b_=1.00000  FG: ω_0_=0.03057, ω_1_=1.00000, ω_2a_=1.00000, ω_2b_=1.00000 | 0 | NS |  |
| *Carnivora* order |  | null | 87 | -19552.495226 | P_0_=0.94613, P_1_=0.05387, P_2a_=0.00000, P_2b_=0.00000  BG: ω_0_=0.03056, ω_1_=1.00000, ω_2a_=0.03056, ω_2b_=1.00000  FG: ω_0_=0.03056, ω_1_=1.00000, ω_2a_=1.00000, ω_2b_=1.00000 |  |  |  |
|  |  | Alternative | 88 | -19552.495226 | P_0_=0.94613, P_1_=0.05387, P_2a_=0.00000, P_2b_=0.00000  BG: ω_0_=0.03056, ω_1_=1.00000, ω_2a_=0.03056, ω_2b_=1.00000  FG: ω_0_=0.03056, ω_1_=1.00000, ω_2a_=1.00000, ω_2b_=1.00000 | 0 | NS |  |

np: number of parameters for each model, NS: not significant; Positive selection sites are numbered according to the PCSK3 reference sequence in H. sapiens (NM_001289823.1), *probability >0.95, ** probability >0.99.
