# Supplementary material for: Molecular evolution of PCSK family: Analysis of natural selection rate and gene loss
Source: PLoS One. 2021 Oct 28;16(10):e0259085. doi: 10.1371/journal.pone.0259085 (PMC8553125; doi:10.1371/journal.pone.0259085)
Supplement: S14 Table — np: number of parameters for each model, NS: not significant; Positive selection sites are numbered according to the PCSK 5 reference sequence in H. sapiens (NP_001177411.1), *probability >0.95, ** probability >0.99. (DOCX) [file pone.0259085.s051.docx]

**S14 Table. Parameter estimates for PCSK5 branch-site model**

| **Foreground**  **branches** |  | **Model** | **np** | **lnL** | **Model parameters** | **2lnL** | ***P*.value** | **Corresponding sites of**  P**ositive selection in**  **H**.**sapiens** **pcsk5 (Probability**  **(BEB))** |
| --- | --- | --- | --- | --- | --- | --- | --- | --- |
| *Chiroptera* order (bats) |  | null | 70 | -46687.049535 | P_0_=0.74927, P_1_=0.24631, P_2a_=0.00333, P_2b_=0.00109  BG: ω_0_=0.05178, ω_1_=1.00000, ω_2a_=0.05178, ω_2b_=1.00000  FG: ω_0_=0.05178, ω_1_=1.00000, ω_2a_=4.70522, ω_2b_=4.70522 | -5.003952 |  |  |
|  |  | Alternative | 69 | -46689.551511 | P_0_=0.74241, P_1_=0.24501, P_2a_=0.00946, P_2b_=0.00312  BG: ω_0_=0.05143, ω_1_=1.00000, ω_2a_=0.05143, ω_2b_=1.00000  FG: ω_0_=0.05143, ω_1_=1.00000, ω_2a_=1.00000, ω_2b_=1.00000 |  | NS |  |
| *Rodentia* order (rodents) |  | null | 69 | -46649.709238 | P_0_=0.73198, P_1_=0.22208, P_2a_=0.03525, P_2b_=0.01069  BG: ω_0_=0.04784, ω_1_=1.00000, ω_2a_=0.04784, ω_2b_=1.00000  FG: ω_0_=0.04784, ω_1_=1.00000, ω_2a_=1.00000, ω_2b_=1.00000 |  |  |  |
|  |  | Alternative | 70 | -46649.709240 | P_0_=0.73198, P_1_=0.22208, P_2a_=0.03525, P_2b_=0.01069  BG: ω_0_=0.04784, ω_1_=1.00000, ω_2a_=0.04784, ω_2b_=1.00000  FG: ω_0_=0.04784, ω_1_=1.00000, ω_2a_=1.00000, ω_2b_=1.00000 | 0 | NS | 978 Y 0.953*  1078 E 0.982* |
| *Muridae* family |  | null | 69 | -46690.551195 | P_0_=0.74712, P_1_=0.24671, P_2a_=0.00463, P_2b_=0.00153  BG: ω_0_=0.05182, ω_1_=1.00000, ω_2a_=0.05182, ω_2b_=1.00000  FG: ω_0_=0.05182, ω_1_=1.00000, ω_2a_=1.00000, ω_2b_=1.00000 |  |  |  |
|  |  | Alternative | 70 | -46688.810080 | P_0_=0.75026, P_1_=0.24763, P_2a_=0.00159, P_2b_=0.00052  BG: ω_0_=0.05188, ω_1_=1.00000, ω_2a_=0.05188, ω_2b_=1.00000  FG: ω_0_=0.05188, ω_1_=1.00000, ω_2a_=4.67876, ω_2b_=4.67876 | 3.48223 | NS | 1647 G |
| *Artiodactyla* order |  | null | 69 | -46690.933085 | P_0_=0.74686, P_1_=0.24558, P_2a_=0.00569, P_2b_=0.00187  BG: ω_0_=0.05186, ω_1_=1.00000, ω_2a_=0.05186, ω_2b_=1.00000  FG: ω_0_=0.05186, ω_1_=1.00000, ω_2a_=1.00000, ω_2b_=1.00000 |  |  |  |
|  |  | Alternative | 70 | -46685.510459 | P_0_=0.75182, P_1_=0.24625, P_2a_=0.00146, P_2b_=0.00048  BG: ω_0_=0.05227, ω_1_=1.00000, ω_2a_=0.05227, ω_2b_=1.00000  FG: ω_0_=0.05227, ω_1_=1.00000, ω_2a_=7.92140, ω_2b_=7.92140 | 10.845252 | <0.001 | 1263Y 0.980* |
| *Balaenopteridae*, *Delphinidae*, *Monodontidae* and *Phocoenidae* families from *Artiodoctyla* order |  | null | 69 | -46689.010969 | P_0_=0.73163, P_1_=0.24087, P_2a_=0.02069, P_2b_=0.00681  BG: ω_0_=0.05150, ω_1_=1.00000, ω_2a_=0.05150, ω_2b_=1.00000  FG: ω_0_=0.05150, ω_1_=1.00000, ω_2a_=1.00000, ω_2b_=1.00000 |  |  |  |
|  |  | Alternative | 70 | -46683.957940 | P_0_=0.75052, P_1_=0.24422, P_2a_=0.00397, P_2b_=0.00129  BG: ω_0_=0.05240, ω_1_=1.00000, ω_2a_=0.05240, ω_2b_=1.00000  FG: ω_0_=0.05240, ω_1_=1.00000, ω_2a_=8.04053, ω_2b_=8.04053 | 10.106058 | <0.0025 |  |
| *Carnivora* order |  | null | 69 | -46685.798555 | P_0_=0.73411, P_1_=0.24179, P_2a_=0.01813, P_2b_=0.00597  BG: ω_0_=0.05091, ω_1_=1.00000, ω_2a_=0.05091, ω_2b_=1.00000  FG: ω_0_=0.05091, ω_1_=1.00000, ω_2a_=1.00000, ω_2b_=1.00000 |  |  |  |
|  |  | Alternative | 70 | -46685.594553 | P_0_=0.74350, P_1_=0.24482, P_2a_=0.00878, P_2b_=0.00289  BG: ω_0_=0.05125, ω_1_=1.00000, ω_2a_=0.05125, ω_2b_=1.00000  FG: ω_0_=0.05125, ω_1_=1.00000, ω_2a_=1.91943, ω_2b_=1.91943 | 0.408004 | NS | 1561N 0.989* |

np: number of parameters for each model, NS: not significant; Positive selection sites are numbered according to the PCSK 5 reference sequence in H. sapiens (NP_001177411.1), *probability >0.95, ** probability >0.99.
