# Supplementary material for: Molecular evolution of PCSK family: Analysis of natural selection rate and gene loss
Source: PLoS One. 2021 Oct 28;16(10):e0259085. doi: 10.1371/journal.pone.0259085 (PMC8553125; doi:10.1371/journal.pone.0259085)
Supplement: S15 Table — np: number of parameters for each model, NS: not significant; Positive selection sites are numbered according to the PCSK3 reference sequence in H. sapiens (NM_001289823.1), *probability >0.95, ** probability >0.99. (DOCX) [file pone.0259085.s052.docx]

**S15 Table. Parameter estimates for PCSK7 branch-site model**

| **Foreground**  **branches** | **Model** | **np** | **lnL** | **Model parameters** | **2ΔlnL** | ***P*.value** | **Corresponding sites of**  P**ositive selection in**  **H**.**sapiens** **pcsk7 (Probability**  **(BEB))** |
| --- | --- | --- | --- | --- | --- | --- | --- |
| *Chiroptera* order (bats) | null | 87 | -20474.531217 | P_0_=0.81637, P_1_=0.15247, P_2a_=0.02626, P_2b_=0.00490  BG: w_0_=0.04656, w_1_=1.00000, w_2a_=0.04656, w_2b_=1.00000  FG: w_0_=0.04656, w_1_=1.00000, w_2a_=1.00000, w_2b_=1.00000 | 0 |  |  |
|  | Alternative | 88 | -20474.531217 | P_0_=0.81637, P_1_=0.15247, P_2a_=0.02626, P_2b_=0.00490  BG: w_0_=0.04656, w_1_=1.00000, w_2a_=0.04656, w_2b_=1.00000  FG: w_0_=0.04656, w_1_=1.00000, w_2a_=1.00000, w_2b_=1.00000 |  | NS |  |
| *Rodentia* order (rodents) | null | 87 | -20450.658626 | P_0_=0.81628, P_1_=0.13892, P_2a_=0.03828, P_2b_=0.00652  BG: w_0_=0.04435, w_1_=1.00000, w_2a_=0.04435, w_2b_=1.00000  FG: w_0_=0.04435, w_1_=1.00000, w_2a_=1.00000, w_2b_=1.00000 | 0 |  | 71 E 0.998**  659 T 0.989*  693 V 0.988* |
|  | Alternative | 88 | -20450.658626 | P_0_=0.81628, P_1_=0.13892, P_2a_=0.03828, P_2b_=0.00652  BG: w_0_=0.04435, w_1_=1.00000, w_2a_=0.04435, w_2b_=1.00000  FG: w_0_=0.04435, w_1_=1.00000, w_2a_=1.00000, w_2b_=1.00000 |  | NS |  |
| *Muridae* family | null | 87 | -20455.310597 | P_0_=0.78358, P_1_=0.14097, P_2a_=0.06395, P_2b_= 0.01151  BG: w_0_=0.04548, w_1_=1.00000, w_2a_=0.04548, w_2b_=1.00000  FG: w_0_=0.04548, w_1_=1.00000, w_2a_=1.00000, w_2b_=1.00000 | 4.536734 |  |  |
|  | Alternative | 88 | -20453.042230 | P_0_=0.81322, P_1_=0.14610, P_2a_=0.03449, P_2b_=0.00620  BG: w_0_=0.04591, w_1_=1.00000, w_2a_=0.04591, w_2b_=1.00000  FG: w_0_=0.04591, w_1_=1.00000, w_2a_=2.24719, w_2b_=2.24719 |  | <0.05 | 598 E 0.982*  652 I 0.964*  659 T 0.999**  662 P 0.976*  781 E 0.965* |
| *Artiodactyla* order | null | 87 | -20474.497254 | P_0_=0.83175, P_1_=0.15033, P_2a_=0.01517, P_2b_=0.00274  BG: w_0_=0.03024, w_1_=1.00000, w_2a_=0.03024, w_2b_=1.00000  FG: w_0_=0.03024, w_1_=1.00000, w_2a_=1.00000, w_2b_=1.00000 | 0.079544 |  |  |
|  | Alternative | 88 | -20474.457482 | P_0_=0.83487, P_1_=0.15075, P_2a_=0.01218, P_2b_=0.00220  BG: w_0_=0.04784, w_1_=1.00000, w_2a_=0.04784, w_2b_=1.00000  FG: w_0_=0.04784, w_1_=1.00000, w_2a_=1.23599, w_2b_=1.23599 |  | NS | 683 M 0.967* |
| *Balaenopteridae*, *Delphinidae*, *Monodontidae* and *Phocoenidae* families from *Artiodoctyla* order | null | 87 | -20475.206304 | P_0_=0.81290, P_1_=0.14984, P_2a_=0.03146, P_2b_=0.00580  BG: w_0_=0.04774, w_1_=1.00000, w_2a_=0.04774, w_2b_=1.00000  FG: w_0_=0.04774, w_1_=1.00000, w_2a_=1.00000, w_2b_=1.00000 | 0 |  |  |
|  | Alternative | 88 | -20475.206304 | P_0_=0.81290, P_1_=0.14984, P_2a_=0.03146, P_2b_=0.00580  BG: w_0_=0.04774, w_1_=1.00000, w_2a_=0.04774, w_2b_=1.00000  FG: w_0_=0.04774, w_1_=1.00000, w_2a_=1.00000, w_2b_=1.00000 |  | NS | 128 R 0.956* |
| *Carnivora* order | null | 87 | -20477.491615 | P_0_=0.84325, P_1_=0.15675, P_2a_=0.00000, P_2b_=0.00000  BG: w_0_=0.04840, w_1_=1.00000, w_2a_=0.04840, w_2b_=1.00000  FG: w_0_=0.04840, w_1_=1.00000, w_2a_=1.00000, w_2b_=1.00000 | 0.000002 |  |  |
|  | Alternative | 88 | -20477.491614 | P_0_=0.84325, P_1_=0.15675, P_2a_=0.00000, P_2b_=0.00000  BG: w_0_=0.04840, w_1_=1.00000, w_2a_=0.04840, w_2b_=1.00000  FG: w_0_=0.04840, w_1_=1.00000, w_2a_=1.00000, w_2b_=1.00000 |  | NS |  |

np: number of parameters for each model, NS: not significant; Positive selection sites are numbered according to the PCSK3 reference sequence in H. sapiens (NM_001289823.1), *probability >0.95, ** probability >0.99.
