# Supplementary material for: Molecular evolution of PCSK family: Analysis of natural selection rate and gene loss
Source: PLoS One. 2021 Oct 28;16(10):e0259085. doi: 10.1371/journal.pone.0259085 (PMC8553125; doi:10.1371/journal.pone.0259085)
Supplement: S16 Table — np: number of parameters for each model, NS: not significant; Positive selection sites are numbered according to the MBTPS1 reference sequence in H. sapiens (NP_001177411.1), *probability >0.95, ** probability >0.99. (DOCX) [file pone.0259085.s053.docx]

**S16 Table. Parameter estimates for MBTPS 1 branch-site model**

| **Foreground**  **branches** | **Model** | **np** | **lnL** | **Model parameters** | **2ΔlnL** | ***P*.value** | **Corresponding sites of**  P**ositive selection in**  **H**.**sapiens** **LDLR (Probability**  **(BEB))** |
| --- | --- | --- | --- | --- | --- | --- | --- |
| *Chiroptera* order (bats) | null | 89 | -20917.214867 | P_0_=0.973332, P_1_=0.02668, P_2a_=0.00000, P_2b_=0.00000  BG: ω_0_=0.02383, ω_1_=1.00000, ω_2a_=0.02383, ω_2b_=1.00000  FG: ω_0_=0.02383, ω_1_=1.00000, ω_2a_=1.00000, ω_2b_=1.00000 |  |  |  |
|  | Alternative | 90 | -21111.537782 | P_0_=0.97341, P_1_=0.02659, P_2a_=0.00000, P_2b_=0.00000  BG: ω_0_=0.02727, ω_1_=1.00000, ω_2a_=0.02727, ω_2b_=1.00000  FG: ω_0_=0.02727, ω_1_=1.00000, ω_2a_=1.00000, ω_2b_=1.00000 | -388.64583 |  |  |
| *Rodentia* order (rodents) | null | 89 | -20875.846564 | P_0_=0.95415, P_1_=0.01827, P_2a_=0.02706, P_2b_=0.00052  BG: ω_0_=0.02167, ω_1_=1.00000, ω_2a_=0.02167, ω_2b_=1.00000  FG: ω_0_=0.02167, ω_1_=1.00000, ω_2a_=1.00000, ω_2b_=1.00000 |  |  |  |
|  | Alternative | 90 | -20875.846564 | P_0_=0.95415, P_1_=0.01827, P_2a_=0.02706, P_2b_=0.00052  BG: ω_0_=0.02167, ω_1_=1.00000, ω_2a_=0.02167, ω_2b_=1.00000  FG: ω_0_=0.02167, ω_1_=1.00000, ω_2a_=1.00000, ω_2b_=1.00000 | 0 | NS | 138 Y 0.980*  163 R 0.991**  722 I 0.972*  795 K 0.992** |
| *Muridae* family | null | 89 | -20909.977227 | P_0_=0.95969, P_1_=0.02506, P_2a_=0.01487, P_2b_=0.00039  BG: ω_0_=0.02334, ω_1_=1.00000, ω_2a_=0.02334, ω_2b_=1.00000  FG: ω_0_=0.02334, ω_1_=1.00000, ω_2a_=1.00000, ω_2b_=1.00000 |  |  |  |
|  | Alternative | 90 | -20909.547327 | P_0_=0.96618, P_1_=0.02528, P_2a_=0.00833, P_2b_=0.00022  BG: ω_0_=0.02342, ω_1_=1.00000, ω_2a_=0.02342, ω_2b_=1.00000  FG: ω_0_=0.02342, ω_1_=1.00000, ω_2a_=1.86892, ω_2b_=1.86892 | 0.8598 | NS | 596 K 0.997** |
| *Carnivora* order | null | 89 | -20917.214867 | P_0_=0.973332, P_1_=0.02668, P_2a_=0.00000, P_2b_=0.00000  BG: ω_0_=0.02383, ω_1_=1.00000, ω_2a_=0.02383, ω_2b_=1.00000  FG: ω_0_=0.02383, ω_1_=1.00000, ω_2a_=1.00000, ω_2b_=1.00000 |  |  |  |
|  | Alternative | 90 | -20917.214867 | P_0_=0.96585, P_1_=0.02527, P_2a_=0.00866, P_2b_=0.00023  BG: ω_0_=0.02328, ω_1_=1.00000, ω_2a_=0.02328, ω_2b_=1.00000  FG: ω_0_=0.02328, ω_1_=1.00000, ω_2a_=1.00000, ω_2b_=1.00000 | 0 | NS |  |
| *Artiodactyla* order | null | 89 | -20912.632102 | P_0_=0.96585, P_1_=0.02527, P_2a_=0.00866, P_2b_=0.00023  BG: ω_0_=0.02328, ω_1_=1.00000, ω_2a_=0.02328, ω_2b_=1.00000  FG: ω_0_=0.02328, ω_1_=1.00000, ω_2a_=1.00000, ω_2b_=1.00000 |  |  |  |
|  | Alternative | 90 | -20912.632102 | P_0_=0.96585, P_1_=0.02527, P_2a_=0.00866, P_2b_=0.00023  BG: ω_0_=0.02328, ω_1_=1.00000, ω_2a_=0.02328, ω_2b_=1.00000  FG: ω_0_=0.02328, ω_1_=1.00000, ω_2a_=1.00000, ω_2b_=1.00000 | 0 | NS | 819 T 0.992** |
| *Balaenopteridae*, *Delphinidae*, *Monodontidae* and *Phocoenidae* families from *Artiodoctyla* order | null | 89 | -20916.413090 | P_0_=0.95637, P_1_=0.02640, P_2a_=0.01677, P_2b_= 0.00046  BG: ω_0_=0.02340, ω_1_=1.00000, ω_2a_=0.02340, ω_2b_=1.00000  FG: ω_0_=0.02340, ω_1_=1.00000, ω_2a_=1.00000, ω_2b_=1.00000 |  |  |  |
|  | Alternative | 90 | -20916.413090 | P_0_=0.95637, P_1_=0.02640, P_2a_=0.01677, P_2b_= 0.00046  BG: ω_0_=0.02340, ω_1_=1.00000, ω_2a_=0.02340, ω_2b_=1.00000  FG: ω_0_=0.02340, ω_1_=1.00000, ω_2a_=1.00000, ω_2b_=1.00000 | 0 | NS |  |

np: number of parameters for each model, NS: not significant; Positive selection sites are numbered according to the MBTPS1 reference sequence in H. sapiens (NP_001177411.1), *probability >0.95, ** probability >0.99.
