# Supplementary material for: Molecular evolution of PCSK family: Analysis of natural selection rate and gene loss
Source: PLoS One. 2021 Oct 28;16(10):e0259085. doi: 10.1371/journal.pone.0259085 (PMC8553125; doi:10.1371/journal.pone.0259085)
Supplement: S17 Table — np: number of parameters for each model, NS: not significant; Positive selection sites are numbered according to the PCSK 9 reference sequence in H. sapiens (NM_174936.4), *probability >0.95, ** probability >0.99. NM_174936.4. (DOCX) [file pone.0259085.s054.docx]

| **Foreground**  **branches** | **Model** | **np** | **lnL** | **Model parameters** | **2ΔlnL** | ***P*.value** | **Corresponding sites of**  P**ositive selection in**  **H**.**sapiens** **LDLR (Probability**  **(BEB))** |
| --- | --- | --- | --- | --- | --- | --- | --- |
| *Homonidae* family | null | 109 | -25785.662958 | P_0_=0.76552, P_1_=0.19663, P_2a_=0.03011, P_2b_=0.00773  BG: w_0_=0.10332, w_1_=1.00000, w_2a_=0.10332, w_2b_=1.00000  FG: w_0_=0.10332, w_1_=1.00000, w_2a_=1.00000, w_2b_=1.00000 | 0.000004 |  |  |
|  | Alternative | 110 | -25785.662956 | P_0_= 0.76559, P_1_=0.19666, P_2a_=0.03003, P_2b_=0.00771  BG: w_0_=0.10332, w_1_=1.00000, w_2a_=0.10332, w_2b_=1.00000  FG: w_0_=0.10332, w_1_=1.00000, w_2a_=1.00000, w_2b_=1.00000 |  | NS |  |
| *Cercopithecidae* family | null | 109 | -25785.872280 | P_0_=0.79567, P_1_=0.20433, P2a=0.00000, P2b=0.00000  BG: w_0_=0.10361, w_1_=1.00000, w_2a_=0.10361, w_2b_=1.00000  FG: w_0_=0.10361, w_1_=1.00000, w_2a_=1.00000, w_2b_=1.00000 | 0.000002 |  |  |
|  | Alternative | 110 | -25785.872279 | P_0_=0.79567, P_1_=0.79567, P_2a_= 0.00000, P_2b_= 0.00000  BG: w_0_=0.10360, w_1_=1.00000, w_2a_=0.10360, w_2b_=1.00000  FG: w_0_=0.10360, w_1_=1.00000, w_2a_=1.00000, w_2b_=1.00001 |  | NS |  |
| *Rodentia* order (rodents) | null | 109 | -25758.733371 | P0=0.76616, P1=0.17148, P2a=0.05096, P2b=0.01141  BG: w0=0.09818, w1= 1.00000, w2a= 0.09818, w2b= 1.00000  FG: w0=0.09818, w1= 1.00000, w2a= 1.00000, w2b= 1.00000 | 0 |  |  |
|  | Alternative | 110 | -25758.733371 | P0=0.76615, P1=0.17148, P2a=0.05096, P2b=0.01141  BG: w0=0.09818, w1=1.00000, w2a=0.09818, w2b=1.00000  FG: w0=0.09818, w1=1.00000, w2a=1.00000, w2b=1.00000 |  | NS | 89 S 0.981*  94 T 0.999**  515 F 0.985* |
| *Artiodactyla* order | null | 109 | -25784.241834 | P0=0.78754, P1=0.19724, P2a=0.01217, P2b=0.00305  BG: w0=0.10360, w1=1.00000, w2a=0.10360, w2b=1.00000  FG:w0=0.10360,w1=1.00000,w2a=1.00000,w2b=1.00000 | 55.823852 |  |  |
|  | Alternative | 110 | -25756.329908 | P0=0.79521, P1=0.20138, P2a=0.00272, P2b= 0.00069  BG: w0=0.10411, w1=1.00000, w2a=0.10411, w2b=1.00000  FG:w0=0.10411,w1=1.00000,w2a=446.22957,w2b=446.2297 |  | <0.0005 | 2 G 1.000**  3 T 1.000**  555Q 0.996** |
| *Balaenopteridae*, *Delphinidae*, *Monodontidae* and *Phocoenidae* families from *Artiodoctyla* order | null | 109 | -25783.486697 | P0=0.78272, P1=0.19659, P2a=0.01654, P2b=0.00415  BG: w0=0.10361, w1=1.00000, w2a=0.10361, w2b=1.00000  FG: w0=0.10361, w1=1.00000, w2a=1.00000, w2b=1.00000 | 49.881076 |  |  |
|  | Alternative | 110 | -25758.546159 | P0=0.79405, P1=0.19722, P2a=0.00699, P2b=0.00174  BG: w0=0.10459, w1=1.00000, w2a=0.10459, w2b=1.00000  FG: w0=0.10459, w1=1.00000, w2a=45.95946, w2b=45.95946 |  | <0.0005 | 2 G 1.000**  3 T 0.996**  201 M 0.970*  555Q 1.000** |

**S17 Table.** **Parameter estimates for PCSK 9 branch-site model**

np: number of parameters for each model, NS: not significant; Positive selection sites are numbered according to the PCSK 9 reference sequence in H. sapiens (NM_174936.4), *probability >0.95, ** probability >0.99. NM_174936.4.
